# Antennal transcriptome analysis of olfactory genes and tissue expression profiling of odorant binding proteins in *Semanotus bifasciatus* (cerambycidae: coleoptera)

**DOI:** 10.1186/s12864-022-08655-w

**Published:** 2022-06-22

**Authors:** Han Li, Enhua Hao, Yini Li, Huan Yang, Piao Sun, Pengfei Lu, Haili Qiao

**Affiliations:** 1 The Key Laboratory for Silviculture and Conservation of the Ministry of Education, School of Forestry, Beijing Forestry University, 35 Qinghua Dong Road, Haidian District, Beijing, 100083 People’s Republic of China; 2 Institute of Medicinal Plant Development, Chinese Academy of Medical Sciences and Peking Union Medical College, 151 Malianwa North Road, Haidian District, Beijing, 100193 People’s Republic of China

**Keywords:** Antennal transcriptome, Odorant binding protein, Tissue expression profiles, *Semanotus bifasciatus*

## Abstract

**Background:**

Insect olfactory proteins can transmit chemical signals in the environment that serve as the basis for foraging, mate searching, predator avoidance and oviposition selection. *Semanotus bifasciatus is* an important destructive borer pest, but its olfactory mechanism is not clear. We identified the chemosensory genes of *S. bifasciatus* in China, then we conducted a phylogenetic analysis of the olfactory genes of *S. bifasciatus* and other species. And the expression profiles of odorant binding proteins (OBPs) genes in different tissues and different genders of *S. bifasciatus* were determined by quantitative real-time PCR for the first time.

**Results:**

A total of 32 OBPs, 8 chemosensory proteins (CSPs), 71 odorant receptors (ORs), 34 gustatory receptors (GRs), 18 ionotropic receptors (IRs), and 3 sensory neuron membrane proteins (SNMPs) were identified. In the tissue expression analysis of OBP genes, 7 OBPs were higher expressed in antennae, among them, *SbifOBP2*, *SbifOBP3*, *SbifOBP6*, *SbifOBP7* and *SbifOBP20* were female-biased expression, while *SbifOBP1* was male-biased expression and *SbifOBP22* was no-biased expression in antennae. In addition, the expressed levels of *SbifOBP4*, *SbifOBP12*, *SbifOBP15*, *SbifOBP27* and *SbifOBP29* were very poor in the antennae, and *SbifOBP4* and *SbifOBP29* was abundant in the head or legs, and both of them were male-biased expression. While *SbifOBP15* was highly expressed only at the end of the abdomen with its expression level in females three times than males. Other OBPs were expressed not only in antennae but also in various tissues.

**Conclusion:**

We identified 166 olfactory genes from *S. bifasciatus*, and classified these genes into groups and predicted their functions by phylogenetic analysis. The majority of OBPs were antenna-biased expressed, which are involved in odor recognition, sex pheromone detection, and/or host plant volatile detection. However, also some OBPs were detected biased expression in the head, legs or end of the abdomen, indicating that they may function in the different physiological processes in *S. bifasciatus*.

**Supplementary Information:**

The online version contains supplementary material available at 10.1186/s12864-022-08655-w.

## Background

Insects use their olfactory systems to recognize and discriminate chemical cues in the external environment to adjust behaviors such as oviposition, host location, and predator avoidance [1–3]. This task is performed by two major families of small soluble proteins, odorant-binding proteins (OBPs) and chemosensory proteins (CSPs) [4–8]. OBPs are abundant water-soluble acidic proteins with a pattern of six conserved cysteine residues, which are paired into three interlocked disulfide bridges that bind and protect small hydrophobic ligands [9–11]. CSPs have four cysteines that form two disulfide bridges. CSPs are present at high concentrations in chemosensory sensilla lymphs and are broadly expressed in non-sensory tissues [5, 6, 12, 13]. In addition, some membrane bound chemosensory receptors located on the chemical sensory dendrites of olfactory neurons also participate in olfactory signal transduction. These membrane proteins, including odorant receptors (ORs), ionotropic receptors (IRs), gustatory receptors (GRs) and sensory neurons membrane proteins (SNMPs), act as the bridge between extracellular odorant signals and intracellular nerve reactions [3, 14, 15]. ORs are G-protein coupled receptors (GPCRs) with seven transmembrane domains, including a variable odor-specific protein (ORx), and a highly conserved co-receptor protein (ORco) [16, 17]. IRs are related to ionotropic glutamate receptors (iGluRs) and are essential for odor-evoked neuronal responses and for detecting environmental volatile chemicals and tastes [18–21]. Insect GRs were first identified in *Drosophila melanogaster*, are mainly expressed in taste organs, and are associated with contact chemoreception [22, 23]. The GR family includes many related members, and the sequence and abundance of GR family members varies greatly among different species, with the exception of carbon dioxide receptors [24]. SNMPs are membrane-bound CD36 family members located in insect olfactory neurons, which are crucial for pheromone detection [25].

Compared with groups such as Lepidoptera and Hymenoptera, the study of olfactory-related proteins in Coleoptera began relatively late. However, the olfactory genes of more than 20 species of Coleoptera, including *Tribolium castaneum* [26], *Colaphellus bowringi* [27], and *Tenebrio molitor* [28], have been discovered and verified. In Cerambycidae, the olfactory genes of 9 species have been identified, including *Anoplophora chinensis* [29, 30], *Anoplophora glabripennis* [31, 32], *Anoplophora nobilis* [33], *Monochamus alternatus* [34], *Batocera horsfieldi* [35], *Xylotrechus quadripes* [36], *Saperda populnea* [37], *Apriona germari* [38] and *Semanotus bifasciatus* [39]. Identification of new olfactory genes will facilitate new studies of the olfactory behavior of Coleoptera insects.

*S. bifasciatus* (Motschulsky) (Coleoptera: Cerambycidae) is an important destructive borer pest of *Platycladus orientalis* in Japan, the Korean Peninsula, and China [40, 41]. In 1996, it was listed as the object of a forest plant quarantine in China because its seriously threatened to old cypress trees in northern China [42, 43]. Plant volatiles and pheromones are commonly used to develop attractants for forest insect pests. For example, *S. bifasciatus* has strong selectivity for scraps of *P. orientalis* and extracts with *α*-thujene, *α*-pinene, *β*-pinene, *β*-caryophyllene and nerolidol, which are the main compounds used in the “Y”-tube olfactometer and wind tunnel methods [44]. Field trapping experiments carried out with different concentrations of different monomer compounds revealed that the optimal core-trapping formula included a mixture of *α*-pinene, caryophyllene, *α*-terpinene and limonene (each at a concentration of 10%) [45]. In recent studies, a slow-release attractant for *S. bifasciatus*, mainly composed of 31 volatile substances, including 3-carene, cedrene, cedr-8(15)-ene and *α*-longipinene, had a good trapping effect [46]. With regard to pheromones, the antennae of *S. bifasciatus* were more sensitive to bark beetle pheromones in comparison with some monoterpenes and alcohols, especially the compounds 3-methy-l2-cyclohexen-1-ol and 3-methy-l2-cyclohexen-1-one [47]. In a GC–MS study of the cuticular hydrocarbons of *S. bifasciatus* across different 3 developmental stages, the content of methyl branched alkanes in adult cuticular hydro-carbons increased significantly compared with that of larvae, while the proportion of n-alkanes in cuticular hydrocarbons decreased, and it was speculated that 11Me-C26, 11Me-C27, and 3Me-C27 played important roles in the reproductive behaviors of adults [48].

In the process of prevention and treatment of *S. bifasciatus*, the identification of olfactory related genes and the study of olfactory mechanism are particularly important. An earlier study of olfactory-related genes based on antennal transcriptome analysis of *S. bifasciatus* in China sought to explain the reasons underlying differences in the trapping effects of a particular attractant at two locations: Beijing and Shandong province. Olfactory-related genes were identified, and differences in the expression levels and single nucleotide polymorphisms (SNPs) of these genes between the Beijing and Shandong populations were compared to illustrate their diversity [39]. The study demonstrated that genetic polymorphisms in olfactory-related genes and the variation in gene expression levels can explain differences in the attractiveness of traps in different locations for many insect species. However, in *S. bifasciatus,* some other important aspects, including genetic relationships of all chemosensory genes with other species and tissues or gender biased expression levels of some crucial genes, have not been reported. Such works can provide useful data to researcheres to comprehensively analyze phylogenetic relationship of these chemosensory genes and tentatively predict their function in chemcial cummunication in *S. bifasciatus* and the related species.

In this study, we identified the chemosensory genes of *S. bifasciatus*, after which we conducted a phylogenetic analysis of the olfactory genes of *S. bifasciatus* and other species. In addition, the expression profiles of OBP genes, an initial chemosensory protein for insects to specifically recognize external odorants, in different tissues and different genders of *S. bifasciatus* were determined by quantitative real-time PCR for the first time. Our findings may provide a foundation for future functional characterization of the chemosensory genes of *S. bifasciatus* and provide new information about the evolution of olfactory proteins in Cerambycidae.

## Results

### Transcriptome sequencing and Unigene assembly

The antennal cDNA libraries of male and female *S. bifasciatus* were sequenced using the Illumina HiSeq platform. A total of a total of 187,712,876 and 134,022,896 raw reads were obtained from male and female antennae. By removing low quality reads of length less than 20 nt, 185,896,722 and 132,600,212 clean reads were obtained for male and female, and the percentage of Q30 bases was more than 94.07%. Trinity was used to assemble the clean data of all samples from scratch, and the assembly results were optimized and evaluated. The results showed that the number of Unigenes assembled was 45,110, the transcript number was 75,880, and the average length of N50 was 1484 bp. The clean reads of each sample were compared with the reference sequence obtained by Trinity assembly to obtain the mapping results of each sample. The comparison rate in this analysis ranged from 78.93% to 86.20%. The total numbers of Unigenes and transcripts were 43,322 and 72,790, respectively. The longest transcript of each gene was selected as the Unigene.

### Homology analysis and Gene Ontology (GO) annotation

In total, 19,342 Unigenes (42.88% of all 45,110 Unigenes) were annotated to at least one of the databases using the BLASTx and BLASTn programs with an E-value cut-off of 10 e-5. A total of 18,345 (40.67%), 13,704 (30.38%), 3365 (7.46%), 13,137 (29.12%), 9367 (20.76%) and 10,424 (23.11%) Unigenes from *S. bifasciatus* were annotated using the Nr, Pfam, COG, Swiss-Prot, KEGG and GO databases, respectively.

**Fig. 1:**
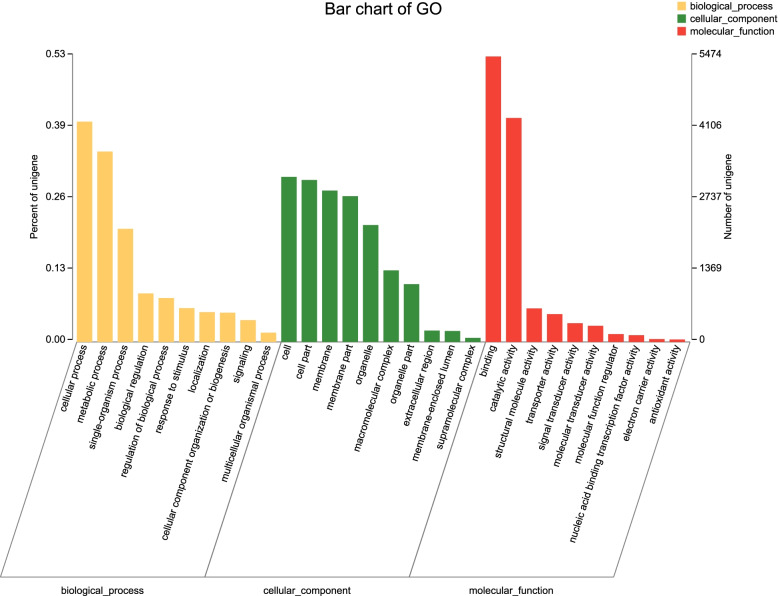
Functional annotation of all Unigenes based on gene ontology (GO) categorization. GO analysis was performed for cellular components, molecular functions, and biological processes

Homology searches against the Nr database showed that *S. bifasciatus* antennal transcriptomes shared the highest homology (59.10%) with sequences from *A. glabripennis*, followed by *Leptinotarsa decemlineata* (8.73%) and *T. castaneum* (5.08%). The GO annotations showed that the most highly annotated levels of *S. bifasciatus* in the biological process branch were cellular process, single organism process, and metabolic process; among the cell component branches, cell and cell part levels were the most representative. In the molecular function branch, hierarchy of binding and catalytic activity were the most enriched terms (Fig. 1).

### Identification and phylogenetic analysis of candidate OBPs

**Fig. 2:**
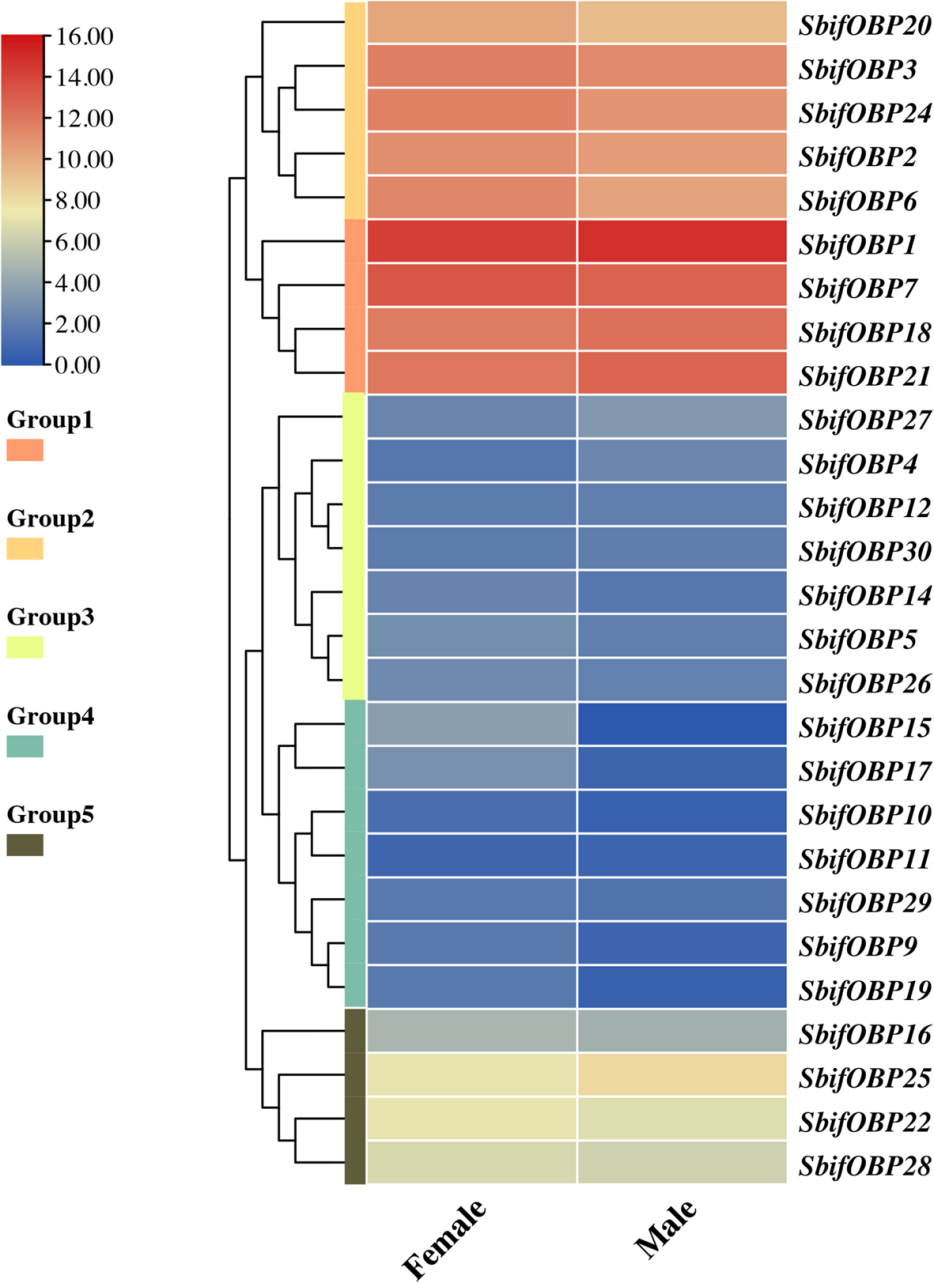
Heat-plot of FPKM values for SbifOBPs in female antennae (FAn) and male antennae (MAn). Relative expression levels are indicated by a 8-grade color scale. The genes were divided into group1-5 according to the expression level

A total of 32 OBPs were identified in the transcriptome of *S. bifasciatus*. All OBPs, with the exceptions of *SbifOBP8, SbifOBP13, SbifOBP23, SbifOBP31* and *SbifOBP32*, had a complete ORF of at least 300 bp and were considered to be full-length OBPs (Additional file 1: Table S1). According to the FPKM value, the expression levels of male and female *SbifOBPs* in antennal transcriptome were divided into five groups for comparison with the results of fluorescent quantitative real-time PCR (Fig. 2). According to the multiple sequence alignment of ClustalW, analyzing the number of conserved cysteine sites contained in OBPs will contribute to its 3D structure modelling. *SbifOBP9, SbifOBP10, SbifOBP12, SbifOBP13, SbifOBP14, SbifOBP15, SbifOBP17, SbifOBP18, SbifOBP20, SbifOBP21, SbifOBP22, SbifOBP24, SbifOBP27, SbifOBP28* and *SbifOBP29* lacked two cysteine residues (C2 and C5) and was determined to belong to the Minus-C OBP subfamily, whereas *SbifOBP19* is a member of the Plus-C OBP subfamily. Each of the remaing OBPs contains 6 conserved cysteine sites and belong to the Classic OBP subfamily.

**Fig. 3:**
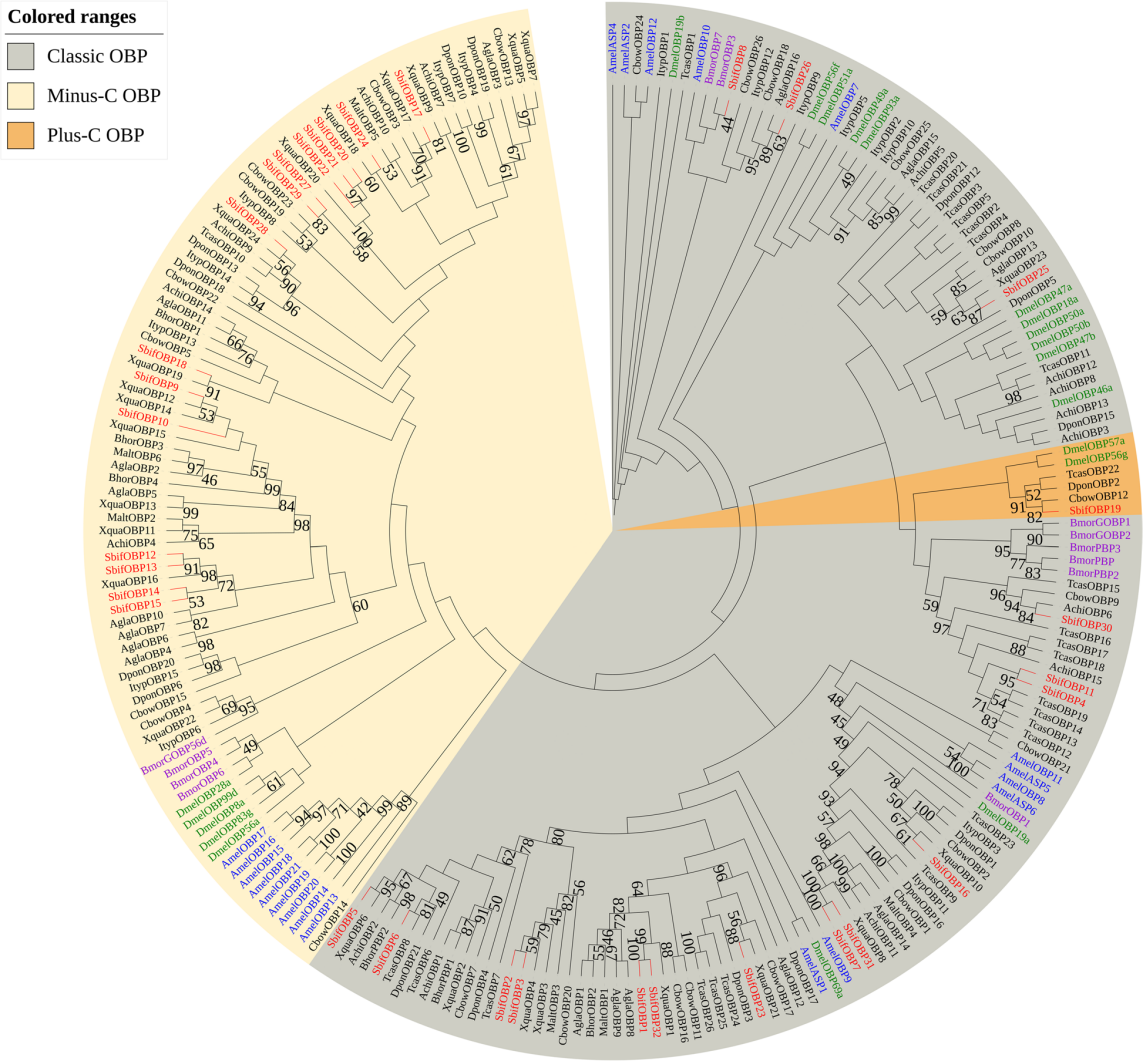
A maximum likelihood phylogenetic tree including candidate odor-binding proteins (OBPs) from Hymenoptera (blue), Diptera (green), Lepidoptera (purple), and Coleoptera (black). Target SbifOBPs are shown in red. Minus-C OBP, GOBP/PBP and plus-C OBP lineages are found in the beige, gray and orange regions, respectively

A total of 233 amino acid sequences of the OBPs of *S. bifasciatus*, *D. melanogastrodia* (Diptera), *Bombyx mori* (Lepidoptera), *Apis mellifera* (Hymenoptera) and other insect species (Coleoptera) were selected to construct the phylogenetic tree. According to multiple sequence alignment and phylogenetic tree analysis, 15 OBPs belonged to the Minus-C OBP subfamily, 16 belonged to the Classic OBP subfamily and 1 belonged to the Plus-C OBP subfamily. Interestingly, the number of Minus-C OBPs was slightly less than that of the Classic OBPs in *S. bifasciatus,* which is unique among studied Cerambycidae species. Most of the SbifOBP clades were clustered with *X. quadripes*, *A. chinensis*, and *A. glabripennis*, with a bootstrap support value of more than 80. SbifOBP19 has additional cysteines and is clustered together with Plus-C OBPs from other Coleopteran insects (Fig. 3).

### Identification and phylogenetic analysis of candidate CSPs

A total of 8 chemosensory proteins were identified in the antennal transcriptome of *S. bifasciatus*. The FPKM values of *SbifCSP1*, *SbifCSP3*, *SbifCSP4*, *SbifCSP6* and *SbifCSP7* were much higher than 100, indicating that they were highly expressed in the antenna of *S. bifasciatus*. In contrast to odorant binding proteins, chemosensory proteins are widely distributed in both olfactory and non-olfactory organs, and may play a role in the growth and development of insects in addition to their roles in the olfactory system (Additional file 1: Table S2).

**Fig. 4:**
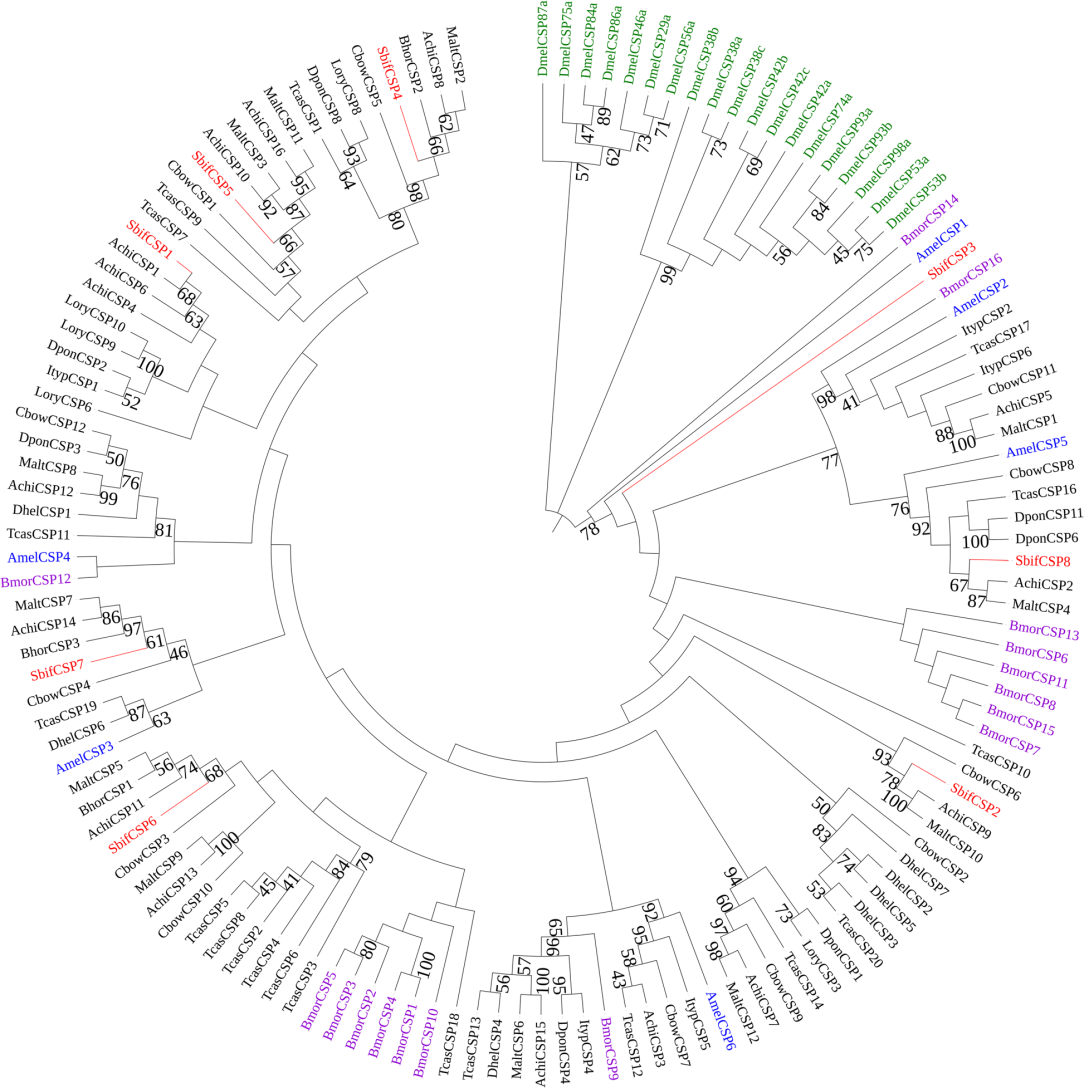
A maximum likelihood phylogenetic tree including CSP candidates for Hymenoptera (blue), Diptera (green), Lepidoptera (purple), and Coleoptera (black). Target SbifCSPs are shown in red

A phylogenetic tree was constructed to show the evolutionary relationships between the chemosensory proteins of various insects, including *D. melanoglycera* (Diptera), *B. mori* (Lepidoptera), *A. mellifera* (Hymenoptera) and Coleoptera species (Fig. 4). In the phylogenetic tree, most SbifCSPs were clustered with *A. chinensis*, *B. horsfieldi* and *C. bowringi* and the bootstrap support degree was greater than 85.

### Identification and phylogenetic analysis of candidate ORs

We identified 71 odorant receptors in the antennal transcriptome of *S. bifasciatus*, 51 of which had complete ORFs. Odorant receptor proteins are located on the neuronal membrane and are relatively low in abundance, so their overall expression levels and FPKM values are low. Of the 71 odorant receptors expressed by *S. bifasciatus*, only 15 had FPKM values more than 10. The FPKM values of 12 odorant receptors in males is more than that of females by two-fold, and those of *SbifOR59* and *SbifOR33* showed a nearly tenfold difference (Additional file 1: Table S3). The differences in the expression levels of odorant receptor proteins between males and females may indicate that they play a role in the recognition of gender-related odors.

**Fig. 5:**
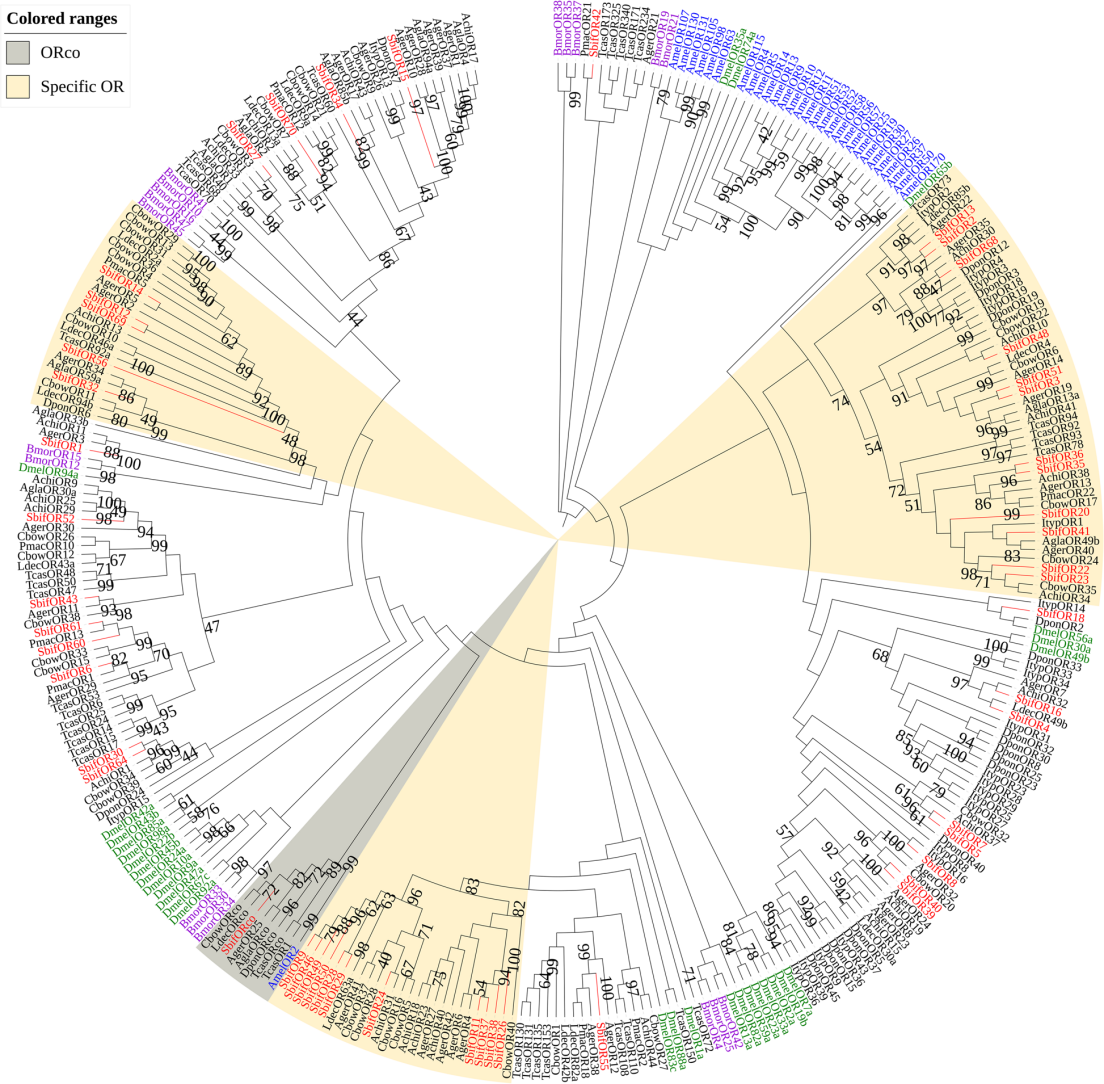
A maximum likelihood phylogenetic tree including candidate odorant receptors (OR) from Hymenoptera (blue), Diptera (green), Lepidoptera (purple), and Coleoptera (black). Target SbifORs are shown in red. Specific OR and ORco lineages are found in the beige and gray regions, respectively

The amino acid sequences of the odorant receptors of *S. bifasciatus* and other species (a total of 335 species of Hymenoptera, Lepidoptera, Diptera, and Coleoptera) were used to construct a phylogenetic tree displaying the evolutionary relationships of the odorant receptors of *S. bifasciatus* and hymenoptera, lepidoptera, diptera, and coleoptera insects. The evolutionary ORco lineage contains SbifORco and the ORco of other species, with a branch node that has 100 bootstrap support, confirming the identity of the odorant receptor ORco. Multiple Coleoptera odorant receptors were found to cluster into a single branch with bootstrap support greater than 70, suggesting that these receptors form a group of ORs with similar functions (Fig. 5).

### Identification and phylogenetic analysis of candidate GRs

A total of 34 gustatory receptors were identified in the antennal transcriptome of *S. bifasciatus*. Blastx sequence alignment showed that most of the taste receptors of *S. bifasciatus* had high homology with those of *A. chinensis*. FPKM values showed that the expression levels of gustatory receptors in the transcriptome of *S. bifasciatus* were relatively low, and only the FPKM value of *SbifGR16* exceeded 10 (Additional file 1: Table S4).

**Fig. 6:**
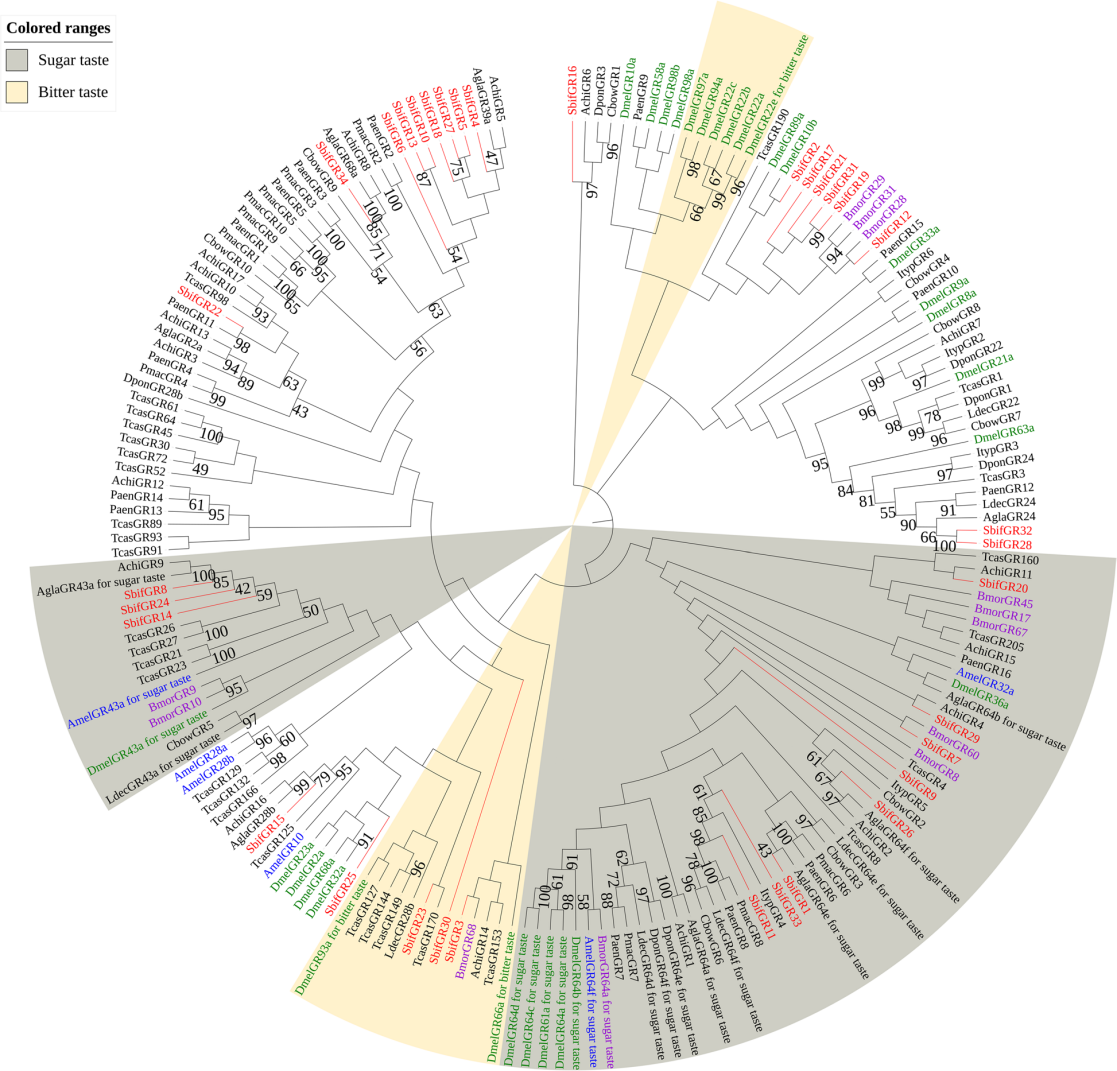
A maximum likelihood phylogenetic tree including candidate gustatory receptors (GRs) for Hymenoptera (blue), Diptera (green), Lepidoptera (purple), and Coleoptera (black). Target SbifGRs are shown in red. Sweet and bitter receptor lineages are found in the gray and beige regions, respectively

A phylogenetic tree was constructed using the gustatory receptors of 194 species, including *S. bifasciatus*, other Coleoptera species, and Diptera, Lepidoptera, and Hymenoptera. The phylogenetic tree (Fig. 6) has two primary branches for sugar taste receptors and bitter taste receptors. 11 gustatory receptors (SbifGR1, SbifGR7, SbifGR8, SbifGR9, SbifGR11, SbifGR14, SbifGR20, SbifGR24, SbifGR26, SbifGR29 and SbifGR33) were classified into the sweet taste receptor clade, while three gustatory receptors (SbifGR3, SbifGR23 and SbifGR30) were included in the branch of the bitter taste receptors. Most of the indentified gustatory receptors in *S. bifasciatus* are sweet taste receptors, which indicate that the primary function of most taste receptors in its olfactory system is carbohydrate detection.

### Identification and phylogenetic analysis of candidate IRs

A total of 18 ionotropic receptors were identified in the transcriptome of *S. bifasciatus*, and 4 of them (*SbifIR8*, *SbifIR12*, *SbifIR15* and *SbifIR17*) had complete ORFs encoding ≥ 600 amino acids. The overall expression levels of ionotropic receptors were relatively low, with only *SbifIR3*, *SbifIR12* and *SbifIR18* having FPKM values greater than 10 (Additional file 1: Table S5).

**Fig. 7:**
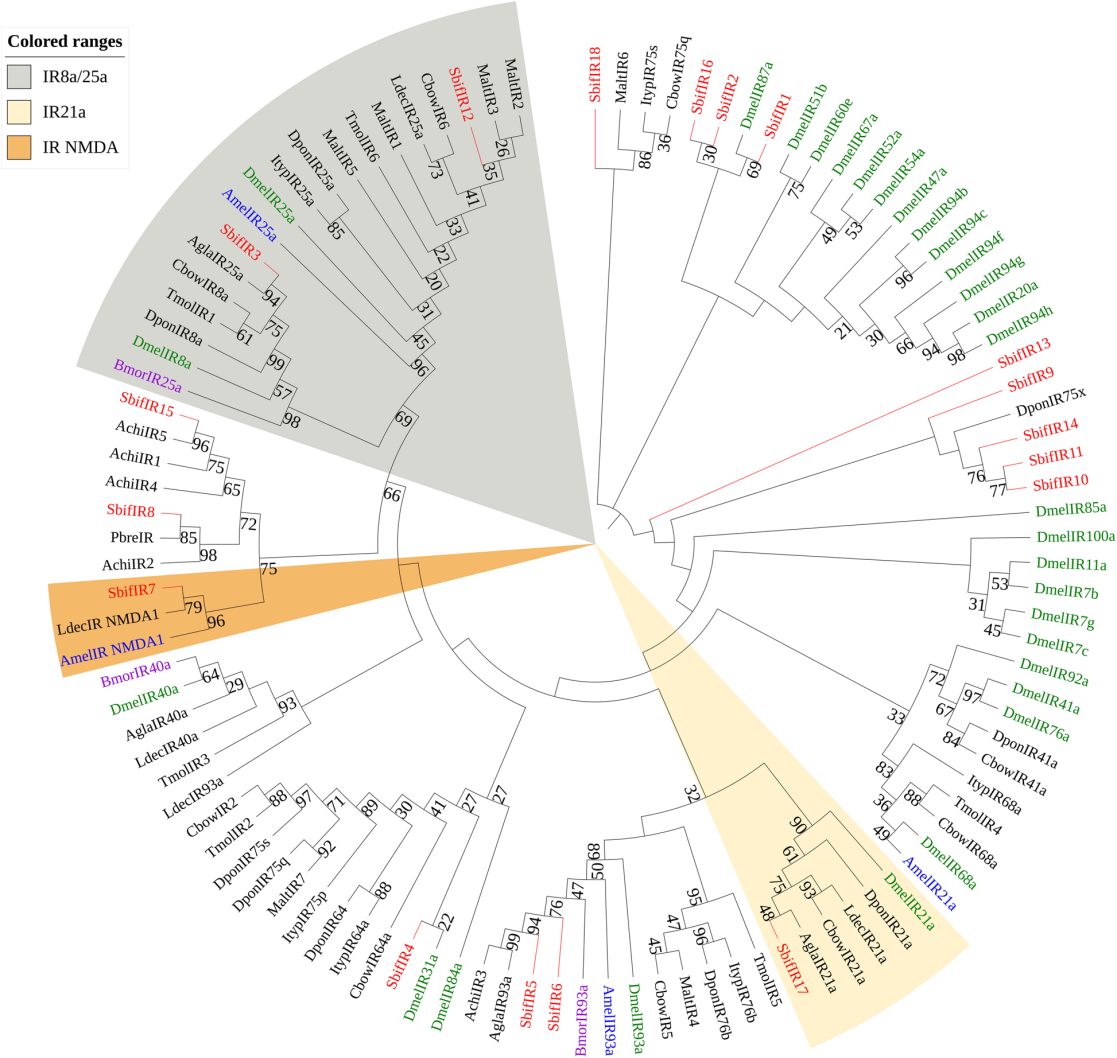
A maximum likelihood phylogenetic tree including candidate ionotropic receptor (IRs) for Hymenoptera (blue), Diptera (green), Lepidoptera (purple), and Coleoptera (black). Target SbifIRs are shown in red. The IR8A/25A, IR NMDA, and IR21A lineages are found in the gray, beige, and orange regions, respectively

Phylogenetic tree was constructed by using 107 amino acid sequences of *S. bifasciatus* and four model insects (*D. melanogastrodia*, *B. mori*, *A. mellifera*, *T. castaneum*) and other Coleoptera insects. It was found that SbifIR3 and SbifIR12 were in the IR8A/25A lineage and had a support degree of more than 90. However, SbifIR7 and the N-methyl-d-aspartate NMDA receptors of *L. decemlineata* and *A. mellifera* were clustered in one branch (Fig. 7).

### Identification and phylogenetic analysis of candidate SNMPs

**Fig. 8:**
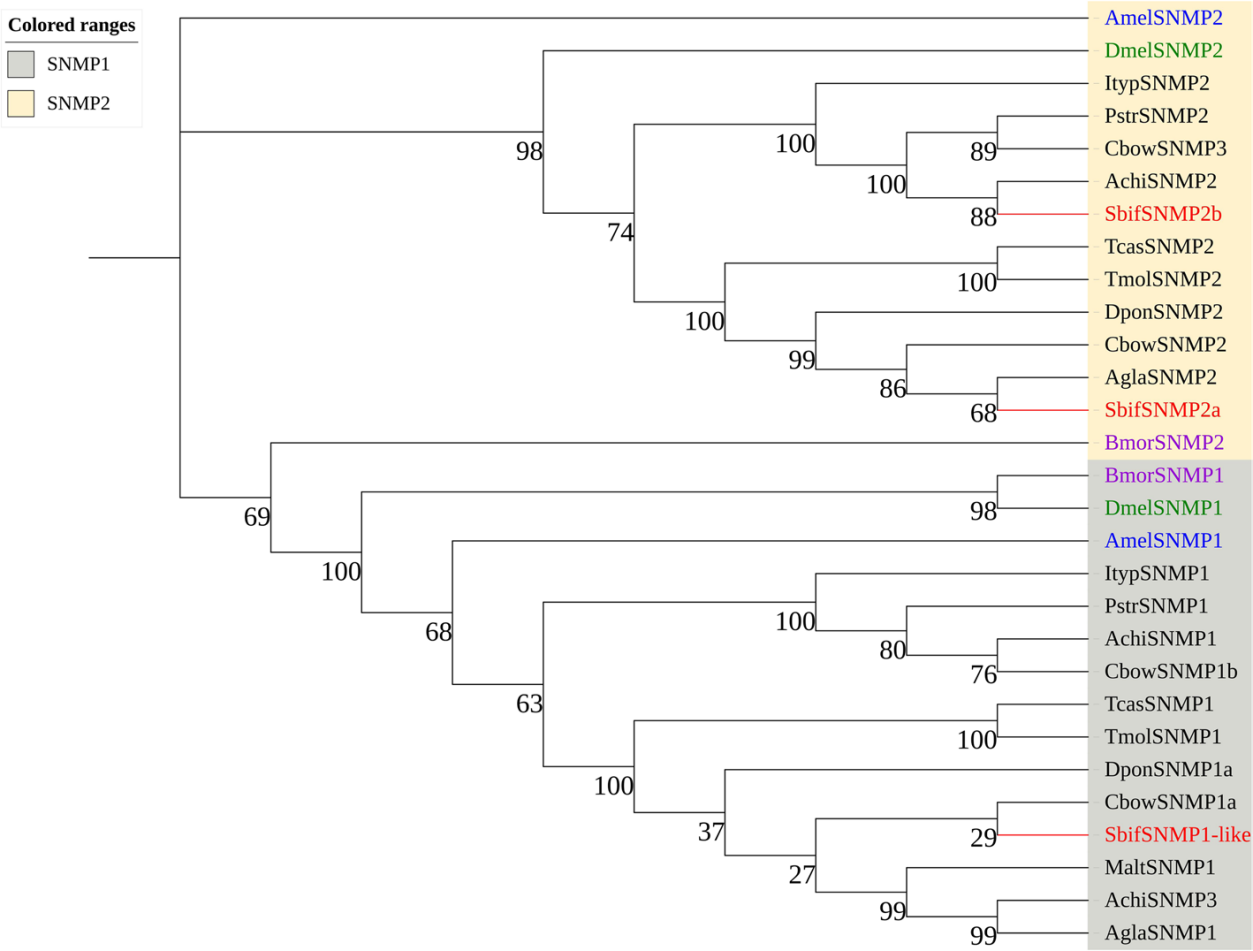
A maximum likelihood phylogenetic tree including candidate sensory neuron membrane proteins (SNMPs) of the orders Hymenoptera (blue), Diptera (green), Lepidoptera (purple), and Coleoptera (black). Target SbifSNMPs are shown in red. The SNMP1 and SNMP2 lineages are found in the gray and beige regions, respectively

Three transcripts encoding SNMPs were identified and were used to constructa phylogenetic tree with 30 sequences from *D. melanogaster* (Diptera), *A. mellifera* (Hymenoptera) and 9 Coleoptera species, including *A. glabripennis* and *A. chinensis*. Two genes belonging to the SNMP2a lineage were of full-length genes (more than 1000 bp in length) and were clustered into one branch together with the SNMP2a protein of A. glabripennis and A. chinensis with bootstrap support of more than 85 (Fig. 8). (Additional file 1: Table S6).

### Tissue expression analysis of OBPs

To verify OBP expression in the antennae and characterize the expression profiles of OBPs in 4 chemosensory tissues (antennae, legs, head and tail of the abdomen), all 32 OBPs of *S. bifasciatus* were selected for fluorescent quantitative real-time PCR. In the selection of internal reference genes, geNorm sorted the expression stability of seven candidate internal reference genes in different tissues of female and male *S. bifasciatus* by calculating the gene expression stability value *M*, with a smaller *M* value indicating a more stable internal reference gene. The optimal number of internal reference genes was determined by analyzing the variation of Vn/n + 1. In this study, the V2/3 and V3/4 values were both around 0.1 (less than the recommended value of 0.15 in the program [49, 50]). NormFinder selects the best reference genes by comprehensively calculating and comparing the expression stability values of the reference genes in a particular group; a smaller stability value indicates a more stable reference gene [51]. Based on the results of Genorm and NormFinder, the *UBC* gene was identified as the optimal internal reference gene (Additional file 2: Fig. S1, Fig. S2, Fig. S3).

**Fig. 9:**
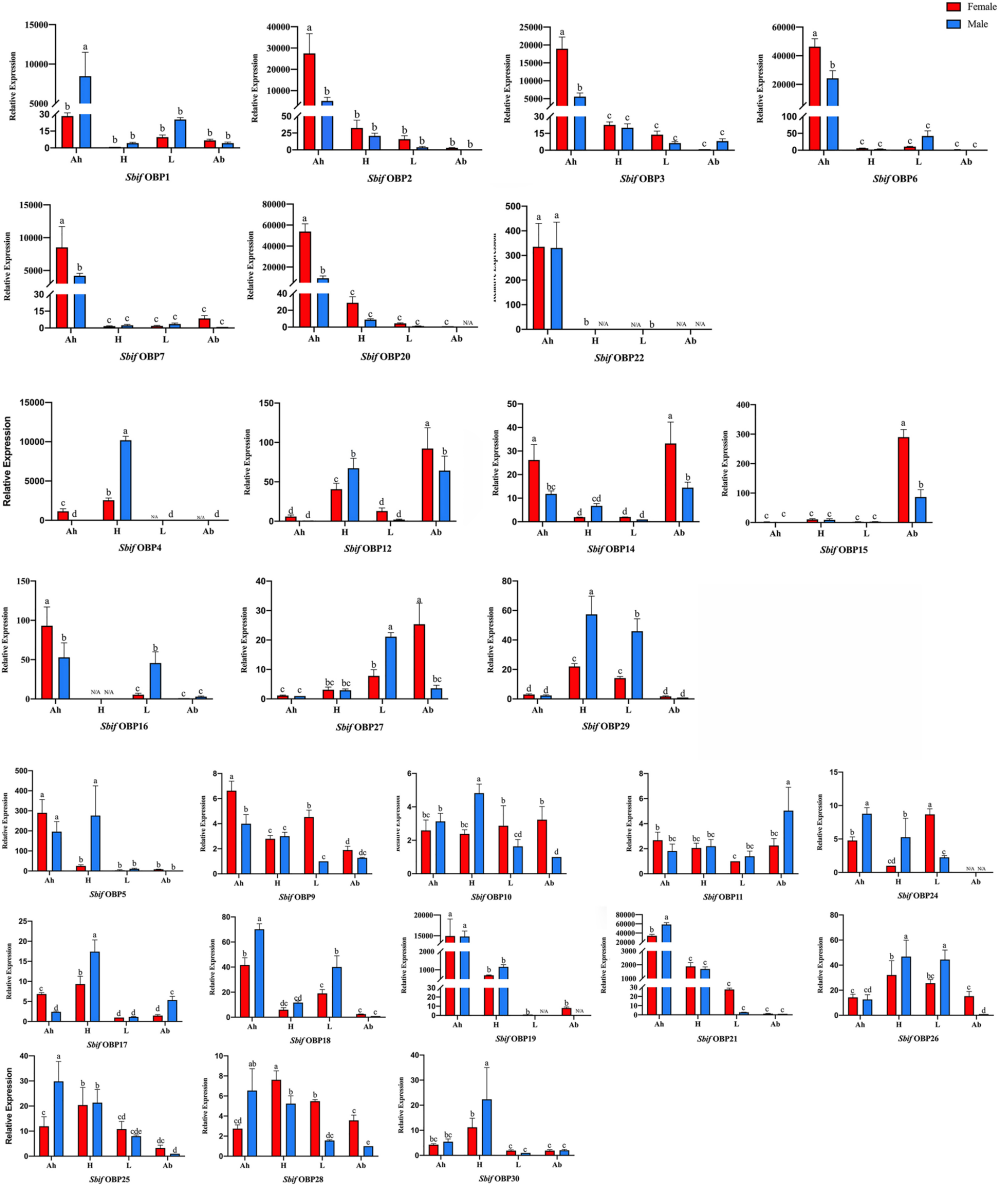
Expression patterns of candidate OBPs in *S. bifasciatus*. An: antennae; H: head; L: legs; AB: tail of the abdomen. The letters over the error bars (a-e) denote a significant difference (*P* < 0.05), and "N/a" indicates that the corresponding expression level was below the limit of detection. The genes were divided into group1-5 according to the expression level

The qPCR results showed that *SbifOBP1*, *SbifOBP2*, *SbifOBP3*, *SbifOBP6*, *SbifOBP7*, *SbifOBP20*, and *SbifOBP22* were highly expressed only in the antennae (Fig. 9). The expression levels of *SbifOBP2, SbifOBP3, SbifOBP6, SbifOBP7* and *SbifOBP20* in females were approximately twice or more than those in males. The expression levels of *SbifOBP1* was male-bias in antennae and *SbifOBP22* was no significantly different in both sexes antennae (*P* < 0.05).

In addition, the expression levels of *SbifOBP4*, *SbifOBP12*, *SbifOBP15*, *SbifOBP27* and *SbifOBP29* were very poor in the antennae. The expression level of *SbifOBP4* in the head and *SbifOBP29* in the head and legs were higher, and both of them were male-biased expression. *SbifOBP12* showed high biased expression in the male head and female abdomen. *SbifOBP15* was highly expressed only at the end of the abdomen of *S. bifasciatus*, where its expression level in females was three times that of males. *SbifOBP27* showed high biased expression in the male leg and female abdomen.

Other OBPs were expressed not only in antennae but also in other various tissues. *SbifOBP14* was higher in female abdomen. *SbifOBP16* was higher in male legs. *SbifOBP17* and *SbifOBP30* showed high male-biased expression in the head. Among the five groups divided according to the expression level, the male and female differences and expression amount shown by qPCR were the same as the FPKM value obtained by transcriptome sequencing (Additional file 3: Fig. S1).

## Discussion

The olfactory genes of more than 20 species of Coleoptera have been identified, but only those of 9 species of Cerambycidae have been reported, including *A. chinensis* [29, 30], *A. glabripennis* [31, 32], *A. nobilis* [33], *M. alternatus* [34], *B. horsfieldi* [35], *X. quadripes* [36], *S. populnea* [37], *A. germari* [38] and *S. bifasciatus* [39]. Although some olfactory gene of *S. bifasciatus* have been characterized, its olfactory proteins have not been comprehensively classified and analyzed. By screening annotation information and using BLASTx, we identified 32 OBPs, 8 CSPs, 71 ORs, 34 GRs, 18 IRs and 3 SNMPs, which were similar to the corresponding numbers of each group identified in the transcriptomes of *A. chinensis*, *A. glabripennis* and other Coleoptera. We named the six identified genes uniformly and submitted their sequences to the NCBI database, obtaining the GeneBank reference numbers OK182368–OK182533.

OBPs perform crucial functions in odorant recognition and transportation, and they typically contain between 120 and 150 amino acids and have a mass of approximately 14 kDa [6, 52]. Our phylogenetic tree classifies OBP as Classic OBPs, Minus-C OBPs and Plus-C OBPs based on a conserved six-cysteine-residue pattern. SbifOBP19 clusters with members of the Plus-C subfamily (TcasOBP22, DponOBP2 and CbowOBP12) with bootstrap support of 91, consistent with previous results [53–55]. Fifteen SbifOBPs belonged to the Minus-C OBPs subfamily. Some studies have shown that Minus-C OBPs originated from a Classic OBP that lost two cysteine residues during evolution, rather than having evolved from a Minus-C OBP of another species [56]. The Plus-C and Minus-C subfamilies play different roles in different biological processes [57–59]. PBPs are a branch of Classic OBPs known for high levels of expression in Lepidopteran male antennae, and it has been reported that many insect PBPs can bind pheromones [52, 60, 61].

OBP genes were highly expressed in adult antennae, suggesting that they may play a role in odor recognition, sex pheromone detection, and/or host plant volatile detection by *S. bifasciatus*. Among the identified 32 OBP sequences, 19 OBPs had the highest similarity with those of *X. quadripes* (all above 60%); *XquaOBP4, XquaOBP8, XquaOBP18 and XquaOBP21* are specifically expressed in the antennae of *X. quadripes* [36]. SbifOBP7, SbifOBP20 and SbifOBP22 are clustered with them in the phylogenetic tree, and corresponding genes were found to be specifically expressed only in the antennae of *S. bifasciatus*. In *A. glabripennis*, the OBP gene significantly expressed in adult antennae can bind to the male-produced aggregation-sex pheromones or the contact recognition pheromone produced by the female [60, 62]. *SbifOBP2, SbifOBP3, SbifOBP6, SbifOBP7* and *SbifOBP20* in female antennae are more than two times higher than those of male antennae, suggesting that they play an important role in identifying the volatiles of host plants or finding suitable sites for oviposition [63], and they may also help to capture and transport aggregation pheromone compounds to ORs [64]. The expression levels of *SbifOBP1*, *SbifOBP18*, *SbifOBP25* and *SbifOBP28* in male antennae were higher than those of females, suggesting that it may play a role in female sex pheromone recognition [65].

OBPs in other organs have functions distinct from chemosensation [66]. Zhang et al. (2020) reported the involvement of *TcasOBPC11* in sequestration of exogenous toxicants in the larvae of *T. castaneum* based on its high expression in the adipose body and epidermis, and this finding was verified by RNAi [67]. In the study, *SbifOBP15* was highly expressed only at the end of the abdomen of *S. bifasciatus*, where its expression level in females was three times that of males. Therefore, we can infer *SbifOBP15* plays an important role in finding suitable oviposition sites for females, but follow-up functional verification must be carried out. *BhorPBP2* has male-biased expression and can bind a broad range of host-plant-related odorants [68]. In the phylogenetic tree constructed in this study, SbifOBP5 and BhorPBP2 were clustered into one branch with bootstrap support of 67, but the qPCR results showed that the *SbifOBP5 was* expressed not only both sexes in the antennae of but also male head. Therefore, the specific functions of *SbifOBP5* need to be further verified. Some recent studies of OBPs suggest that this group of proteins are highly adaptive hydrophobic carriers that perform a variety of physiological functions beyond their classical role in chemoreception [8]. A recent report by Wang et al. (2020) regarding two *H. armigera* OBPs is consistent with this view, and their results suggest an additional physiological role in regulating insect flight in migratory Lepidopterans [69].

CSPs are more highly conserved than OBPs and are widely expressed in different parts of the insect body [30, 70]. In addition, CSPs have multiple functions. For example, CSP genes play important roles in insect host-searching behavior [2, 8]. It was confirmed by RNA interference combined with olfactory behavior experiment, CSPs and one takeout gene (LmigTO1) can modulate the switch between attraction and repulsion during behavioral phase changes in *Locusta migratoria* [71]. Moreover, blocking the embryonic expression of CSP5 in honeybee with double-stranded RNA causes abnormalities in all body parts where CSP5 is highly expressed [72]. This suggests that some CSPs play roles in the development of embryonic integument in *A. mellifera*. Competitive binding assays using tryptophan fluorescence spectroscopy and molecular docking demonstrated that some CSPs in *Bemisia tabaci* are crucial to facilitate the transport of fatty acids thus regulating some metabolic pathways of the insect immune response [73]. The seven identified SbifCSPs were clustered with the CSPs of *A. chinensis*, *B. horsfieldi* and *C. bowringi* in the phylogenetic tree, and only SbifCSP3 was clustered with the CSPs of *A. mellifera* (Hymenoptera) and *B. mori* (Lepidoptera), suggesting that it performs functions different from those of other SbifCSP proteins. We compared the FPKM values of male and female *S. bifasciatus* CSPs in the transcriptome. The FPKM values of *SbifCSP3* and *SbifCSP6* in male antennae were about twice as high as those in female antennae, and their expression levels were very high. Therefore, *SbifCSP3* and *SbifCSP6* might play important roles in the recognition of female sex pheromones by male *S. bifasciatus.*

A total of 71 OR genes were identified in the antennal transcriptome of *S. bifasciatus*, which is fewer than the 111 genes in that of *T. castaneum* [26], but more than the 37 in that of *A. glabripennis* [31], the 53 in that of *A. chinensis* [29], the 42 in that of *A. germari* [38], the 49 in that of *D. ponderosae* and the 43 in that of *I. typographus* [74]. Compared to traditional odorant receptors, ORco is highly conserved; amino acid sequence analysis shows that there is a highly conserved region at the end of the ORco sequence [75]. According to the FPKM values from our study, ORco was the most expressed odorant receptor in *S. bifasciatus*, and the expression level of females was much higher than that of males. In our phylogenetic tree, SbifORco is clustered with CbowORco, LdecORco, AgerORco and AglaORco, with bootstrap support as high as 99. The expression of OR genes in antennae of *S. bifasciatus* was preliminarily observed by FPKM value, and it was found that only *SbifOR59* and *SbifOR33* showed a nearly tenfold difference, both of which were highly expressed in female antennae. It is speculated that they play a role in sensing volatiles released by host to find oviposition sites and sensing male pheromones.

As a multimodal receptive entity, IRs detect volatile chemosignals and participate in taste sensation, hygrosensation, and perception of cool temperatures [76, 77]. Phylogenetic analysis showed that SbifIR3 and SbifIR12 belong to the common receptor branch of IR8a/25a, while the NMDA receptor SbifIR7 is clustered with those of *L. decemlineata* and *A. mellifera* in one branch.

Eleven gustatory receptors (SbifGR1, SbifGR7, SbifGR8, SbifGR9, SbifGR11, SbifGR14, SbifGR20, SbifGR24, SbifGR26, SbifGR29 and SbifGR33) were clustered in the sweet receptor branch, while only 3 gustatory receptors (SbifGR3, SbifGR23 and SbifGR30) were clustered in the bitter receptor branch. Most of the taste receptors of *S. bifasciatus* are sweet receptors, which may indicate that carbohydrate detection is the main function of most taste receptors in its olfactory system. Sugar recognition is thought to be involved in the host-plant selection and egg-laying behavior of some female Lepidopteran insects [78, 79].

Members of the insect SNMP1 subfamily are expressed in pheromone-sensitive ORNs, and SNMP2 proteins are expressed in supporting cells rather than in ORNs [80]. SbifSNMP2a and SbifSNMP2b clustered together with the SNMP2 proteins of *A. glabripennis* and *A. chinensis* on the phylogenetic tree, with bootstrap support of more than 65. Further research should be performed to assess the functions of ORs, IRs, GRs, CSPs and SNMPs in *S. bifasciatus.*

During the preparation of our manuscript, an independent and complementary work on *S. bifasciatus* antennal transcriptomes was published online by Zhang et al. (2019) that focused on the mechanisms underlying differences in the trapping effects of a particular attractant between the Beijing and Shandong populations of *S. bifasciatus* [39]. A total of 18 OBPs, 21 CSPs, 66 ORs, 24 GRs, 14 IRs, and 4 SNMPs in *S. bifasciatus* were identified by Zhang et al. (2019). In their study, sections of wood containing overwintering *S. bifasciatus* insects were collected from two sites, the Lingyan Forest district of Taian, Shandong province (116°59′ E, 36°21′ N), and the Mentougou district of Beijing (115°34′ E, 39°50′ N), in March 2018; sampling was performed after eclosion in the laboratory. By comparing the expression levels of olfactory-related genes between males and females in the two populations using FPKM values, it was found that knowledge of single nucleotide polymorphisms (SNPs) may facilitate interpretation of the diversity of chemosensory genes in *S. bifasciatus*. In our study, we identified 32 OBPs, 8 CSPs, 71 ORs, 3 SNMPs, 18 IRs, and 34 GRs; therefore, the numbers of genes annotated in each family differed from those reported by Zhang et al. (2019). We collected *S. bifasciatus* at the Qianfo Hill Forest Farm in Jinan, Shandong Province (117°01′ E, 36°38′ N) and at the Beijing Botanical Garden (116°28 ′ E, 40° N) in May 2019. In our study, four of the 18 OBPs (*OBP6*, *OBP9*, *OBP12* and *OBP16*) identified by Zhang et al. (2019) were significantly expressed in both male and female *S. bifasciatus* (FPKM value > 1000), and 11 of the 32 OBP genes had the same expression level in both studies.

The difference in sampling strategies may be one of the reasons for the different annotation results reported in our study and that of Zhang et al. (2019). In two previous reports on *A. chinensis*, *A. chinensis* collected from Anhui Province and Fujian Province were found to possess marked differences in olfactory-related genes [29, 30]. Although we and Zhang et al. collected *S. bifasciatus* in the same province, the distance between the collection sites was more than 40 km. Taking Shandong Province as an example, the main peak of Lingyan forest district is 668 m above sea level, whereas that of Qianfo hill forest farm was 285 m above sea level. Zhang et al. collected *S. bifasciatus* at the peak of eclosion, but we collected samples at the end of eclosion. It can be inferred that differences in distance, altitude and collection time will lead to differences in gene expression levels, even within the same province. Similar polymorphisms in olfactory recognition were found in a study of the important agricultural pest *Chilo suppressalis*. Sex pheromone recognition by male moths among six Chinese provinces and the expression levels of 12 genes involved in sex pheromone recognition showed significant linear correlations [81]. In addition, sequencing depth, living environment, sample status and other factors also contributed to the differences. For example, in a transcriptome analysis assessing different developmental stages of *B. horsfieldi*, there were large differences in the number of genes identified in larvae, pupae, females and males [82].

Our study focused on the annotation and classification of several olfactory proteins. Phylogenetic trees were used to analyze the genetic relationships of olfactory proteins, and qPCR was used to analyze the expression levels of OBP genes in different tissues of *S. bifasciatus*, allowing us to predict the functions of *SbifOBPs* specifically expressed in *S. bifasciatus* and thus explain the mechanisms by which *S. bifasciatus* search for host trees and other members of the species.

## Conclusions

We identified six olfactory-related genes expressed by *S. bifasciatus*, and we obtained the tissue expression profiles of *SbifOBPs* by RT-qPCR to allow us to predict their functions, providing insight into the mechanisms by which *S. bifasciatus* find host trees and other members of the species. Meanwhile, we compared an article on the transcriptome of the same species published in forests with our research, and analyzed the reasons for the differences at the molecular level. Our results provide a foundation for studies aimed at clarifying the mechanism by which the olfactory system responds to specific odorant molecules via experiments assessing protein expression, fluorescence binding competition, molecular docking, and behavior. Such studies will facilitate the development of attractants that will provide methods for more effective control of *S. bifasciatus*. Finally, our results demonstrate an ideal model for studying the olfactory behavior of other pests, and we provide new ideas and methods for controlling and monitoring other Coleoptera pests.

## Methods

### Insects and tissue collections

The samples of *S. bifasciatus* were collected from two sites, the Qianfo Hill Forest Farm of Jinan, Shandong Province (117°01′ E, 36°38′ N), and Beijing Botanical Garden (116°28 ′ E, 40° N) Beijing, in late May 2019. Damaged cypress trees with a diameter at breast height of 5–13 cm and a tree height of 2–8 m were selected. The target trees with debilitated wood were cut into 1 m long wood sections. Nine wood sections were grouped together, tied into a cube with iron wire, and then placed on an open and well-ventilated hillside. Specimens were caught when they flew out of the wood samples, and their status, sex and other information were recorded. After mating, female and male antennae were excised and then stored at − 80 °C until RNA extraction.

### Total RNA extraction, cDNA library construction, and Illumina sequencing

The antennal RNA was extracted using Trizol reagent (Invitrogen, USA) and the RNeasy plus Mini Kit (No.74134; Qiagen, Hilden, Germany) according to the manufacturer’s instructions. Adult antennae of both sexes (12 antennae from males and 12 antennae from females) were cut by ophthalmic surgical scissors to allow biological repeats and then used for transcriptome sequencing. Three biological repeats were assessed for both male and female antennae.

RNA samples were tested using a Nanodrop 8000 spectrophotometer (Thermo, Waltham, MA, USA) and a 2100 Bionalyzer RNA NanoChip (Agilent, Santa Clara, CA). The RNA samples were sequenced using the Illumina Hiseq 4000 platform (Shanghai Majorbio bio Pharm Technology Co., Ltd.)

The Illumina Hiseq platform was used for short sequence sequencing. The enriched mRNA was a complete RNA sequence with an average length of several bp, so it needed to be randomly interrupted. With the addition of fragmentation buffer, the mRNA was randomly broken into small fragments of about 300 bp. Under the action of reverse transcriptase, random primers were added to perform reverse synthesis of one strand of cDNA with mRNA as the template, after which two-chain synthesis was carried out to form a stable double chain structure.

The structure of the double-stranded cDNA had a sticky end, which was supplemented by end repair mix to form a flat end. An "A" base was added to the 3′ end to connect the Y-shaped junction. The library was enriched, 15 cycles were amplified by PCR, and then the target bands were recovered with 2% agar gum. TBS380 (PicoGreen) was used to quantify the bands. The cBot was amplified by bridge PCR to generate clusters. Finally, the sequence of Illumina Hiseq was carried out (PE library, reading length 2 × 150 bp).

### Assembly and functional annotation

De novo transcriptome was assembled from scratch by using Trinity (https://github.com/trinityrnaseq/trinityrnaseq/wiki) to assemble the short sequences of clean data [83], after which TransRate (http://hibberdlab.com/transrate/) and CD-HIT (http://weizhongli-lab.org/cd-hit/) were used to optimize the initial assembly sequence filter [84, 85], and BUSCO software (Benchmarking Universal Single-Copy Orthologs, http://busco.ezlab.org) was used for assessment [86]. Finally, transcripts and Unigenes were obtained. Generally, the longest sequence of each transcript was selected as the Unigene.

BLAST (Basic Local Alignment Search Tool) was used to search and compare transcript open reading frames (ORFs) with the Nr (non-redundant), String, Swiss-Prot, KEGG and other databases, to obtain the corresponding transcript annotation information [87–89]. The expected e-value was < 1e-5. As to the annotation results of BLAST, the Blast2GO program was used (http://www.blast2go.com/b2ghome) for Unigene processing to obtain the classification and description in the database based on the Unigene annotation results [90–92]. The expression level of each Unigene was represented by its FPKM value (Fragments Per Kilobase of exon model Per Million mapped reads). Higher fragment abundance indicated a higher gene expression level, and the value was calculated based on RSEM (http://www.biomedsearch.com/nih/RSEM-accurate-transcript-quantification-from/21816,040.html) [93].

### Identification of chemosensory genes

With tBLASTn, the available sequences of OBP, CSP, OR, GR, IR, and SNMP proteins from Insecta species were used as queries to identify candidate Unigenes involved in olfaction in *S. bifasciatus*. All candidate OBPs, CSPs, ORs, GRs, IRs, and SNMPs were manually checked by evaluating the NCBI BLASTx results. ORF Finder online software (http://www.ncbi.nlm.nih.gov/gorf/gorf.html) was used to predict the ORFs of genes.

### Phylogenetic analysis

The candidate OBPs and CSPs were searched for the presence of N-terminal signal peptides using SignalP5.0 (http://www.cbs.dtu.dk/services/SignalP/). TMHMM server v3.0 was used to predict the transmembrane domains of candidate ORs, IRs, and GRs (http://www.cbs.dtu.dk/services/TMHMM/). ClustalX software was used for multiple alignment of amino acid sequences [94] (Additional file 4). The maximal-likelihood (ML) method of MEGA X software was used to construct a phylogenetic evolutionary tree (with 1000 Bootstrap replicates) including *S. bifasciatus* and other proximal insects, whose amino acid sequences were downloaded from the protein database of NCBI [95] (Additional file 5). A JTT matrix-based approach was used to calculate the evolutionary distance [96]. Figtree 1.4.3 and ITOL (https://itol.embl.de/) software were used to edit the phylogenetic tree. Heat-plot of FPKM values plotted using TBtools v1.098652 [96, 97].The transcript levels were determined by calculating log2 (reads per kilobase per million mapped reads + 1) values.

### Tissue expression analysis of OBP genes

The relative expression levels of OBP genes in both male and female tissues (antennae, legs, head and tail of the abdomen) were analyzed by fluorescence quantitative real-time PCR. The antennae, legs, heads and abdomens were collected from 12 male adult *S. bifasciatus* and 12 female adult *S. bifasciatus* for each biological replicate. Three biological replicates and three technical replicates were used with each qPCR reaction for each tissue to examine reproducibility. The total RNA of each sample was extracted by the above method, the OD_260/280_ values of the samples were all in the range of 1.8–2.0 by Nanodrop 8000 spectrophotometer. cDNA from various tissues of *S. bifasciatus* was synthesized with the PrimeScript RT kit (No. RR047A; Takara, Shiga, Japan) including gDNA-Eraser. The internal reference gene was the UBC gene of *S. bifasciatus*. GAPDH, RPL13, UBC, RPS3, EF1-α, AK and α-tubulin genes were selected from the antenna transcriptome of *S. bifasciatus*. Using real-time fluorescence quantitative pre-experiments, the genes with the most stable expression were tested as internal reference genes. Primer3plus (http://www.primer3plus.com/cgibin/dev/Primer3Plus.cgi) was used to design specific primers for odorant binding protein genes and internal reference genes of *S. bifasciatus,* with a primer length of 20 bp, GC content of 40–60% and a product length of 150–200 bp.

All OBP genes were subjected to RT-qPCR using the CFX Connect Real-Time System (Bio-Rad, Hercules, CA, USA). The cDNA of each tissue of male and female adults was used as the template, and the total reaction system was 25 µL, including TB Green Premix Ex Taq II (12.5 µL), DDH2O (8.5 µL), forward and reverse primers (1 µL each), and cDNA (2 µL). The RT-qPCR conditions were as follows: 95 °C for 30 s; then 40 cycles of 95 °C for 5 s and 60 °C for 30 s; followed by 65 °C to 95 °C in increments of 0.5 °C for 5 s. The reference gene was UBC. All primers used in the experiment (including the reference gene) are listed in Additional file 6: Table S1. The RT-qPCR data were analyzed by the 2-∆∆CT method [98], the relative expression levels were calculated, and GraphPad Prism version 8.0.0 for Windows (GraphPad Software, San Diego, California USA, www.graphpad.com) was used to graph the results. SPSS 23.0 was used for one-way ANOVA, and the Tukey method was used to test the significance of the difference (*P* < 0.05) (The version information of the software packages used for transcriptome sequencing and bioinformatics analysis was listed in Additional file 7).

## Supplementary Information

**Additional file 1:** Best blastX hits for putative odorant binding proteins (OBPs), chemosensory proteins (CSPs), odorant receptors (ORs), gustatory receptors (GRs), ionotropic receptors (IRs), and sensory neuron membrane proteins (SNMPs) of S. bifasciatus. (Table S1, Table S2, Table S3, Table S4, Table S5 and Table S6). **Table S1.** Sequence information and best blasts match information of odorant binding proteins (OBPs). **Table S2.** Sequence information and best blasts match information of chemosensory proteins (CSPs). **Table S3.** Sequence information and best blasts match information of odorant receptors (ORs). **Table S4.** Sequence information and best blasts match information of gustatory receptors (GRs). **Table S5.** Sequence information and best blasts match information of ionotropic receptors (IRs). **Table S6.** Sequence information and best blasts match information of sensory neuron membrane proteins (SNMPs).

**Additional file 2:** **Fig. S1.** Expression stability values for seven reference genes calculated by geNorm software among different treatments. **Fig. S2.** Pairwise variation (Vn /n + 1) between normalization factors after stepwise inclusion of stable reference genes from the most stably expressed genes. **Fig. S3.** The ranking order of the expression stability value of candidate reference genes in different tissues of S. bifasciatus calculated by NormFinder.

**Additional file 3:** **Fig. S1.** Relative expression levels of SbifOBPs in different genders and tissues. (A) Heat-plot of FPKM values for SbifOBPs in female antennae (FAn) and male antennae (MAn). Relative expression levels are indicated by a 8-grade color scale. (B) Expression patterns of candidate OBPs in S. bifasciatus. An: antennae; H: head; L: legs; AB: tail of the abdomen. The letters over the error bars (a-e) denote a significant difference (P<0.05), and "N/a" indicates that the corresponding expression level was below the limit of detection. The genes were divided into group1-5 according to the expression level.

**Additional file 4.** Alignment of the S. bifasciatus OBPs.

**Additional file 5.** Protein sequences used in phylogenetic trees (downloaded from NCBI).

**Additional file 6:** **Table S1.** Primer s for fluorescence quantitative real time PCR.

**Additional file 7.** The version information or download links of the different software packages used for trimming/assembly/annotation.

## Data Availability

All supporting data is included within the article and its additional files. And the transcriptome data were submitted to NCBI, the accession number of *S. bifasciatus* are from SAMN19654708 to SAMN19654873. All of the olfactory protein gene sequences were submitted to Genbank, accession number are OK182368-OK182533.

## Abbreviations

OBPs: Odorant binding proteins
CSPs: Chemosensory proteins
ORs: Odorant receptors
ORco: Odorant receptor co-receptor
SNMPs: Sensory neuron membrane proteins
GRs: Gustatory receptors
IRs: Ionotropic receptors
ORFs: Open reading frame
FPKM: Fragments per kilobase per million reads
SNPs: Single nucleotide polymorphisms
GO: Gene ontology
ML: Maximal-likelihood
RT-qPCR: Fluorescent quantitative real-time PCR
