# Supplementary material for: Antennal transcriptome analysis of olfactory genes and tissue expression profiling of odorant binding proteins in Semanotus bifasciatus (cerambycidae: coleoptera)
Source: BMC Genomics. 2022 Jun 22;23:461. doi: 10.1186/s12864-022-08655-w (PMC9219211; doi:10.1186/s12864-022-08655-w)
Supplement: Supplementary file 1 — Additional file 1: Best blastX hits for putative odorant binding proteins (OBPs), chemosensory proteins (CSPs), odorant receptors (ORs), gustatory receptors (GRs), ionotropic receptors (IRs), and sensory neuron membrane proteins (SNMPs) of S. bifasciatus. (Table S1, Table S2, Table S3, Table S4, Table S5 and Table S6). Table S1. Sequence information and best blasts match information of odorant binding proteins (OBPs). Table S2. Sequence information and best blasts match information of chemosensory proteins (CSPs). Table S3. Sequence information and best blasts match information of odorant receptors (ORs). Table S4. Sequence information and best blasts match information of gustatory receptors (GRs). Table S5. Sequence information and best blasts match information of ionotropic receptors (IRs). Table S6. Sequence information and best blasts match information of sensory neuron membrane proteins (SNMPs). [file 12864_2022_8655_MOESM1_ESM.pdf]

Table S1. Sequence information and best blasts match information of odorant binding proteins (OBPs)

Number	Gene ID	Complete ORF	Signal peptide	FPKM		Blastx match					
				F	M	Name	score	E value	Ident	Accession	Species
SbifOBP1	TRINITY_DN18761_c6_g2	YES	1-21	20525.523	31569.7133	OBP1	187	3.00E-55	60.42%	AXO78379.1	*Xylotrechus quadripes*
SbifOBP2	TRINITY_DN12152_c3_g4	YES	1-22	2232	1532.73667	OBP4	133	3.00E-36	44.93%	AXO78382.1	*Xylotrechus quadripes*
SbifOBP3	TRINITY_DN17919_c1_g1	YES	1-19	3523.68	2551.54	OBP3	152	1.00E-43	54.20%	AXO78381.1	*Xylotrechus quadripes*
SbifOBP4	TRINITY_DN13088_c1_g3	YES	1-16	2.18	4.433333	OBP5	134	8.00E-37	41.10%	AWT23276.1	*Hycleus cichorii*
SbifOBP5	TRINITY_DN12812_c0_g1	YES	1-19	6.06	3.07	OBP6	179	7.00E-52	61.19%	AXO78384.1	*Xylotrechus quadripes*
SbifOBP6	TRINITY_DN11954_c0_g1	YES	1-18	2742.03	1265.64667	OBP15	170	6.00E-51	61.94%	AIX97030.1	*Monochamus alternatus*
SbifOBP7	TRINITY_DN14241_c0_g1	YES	1-19	10614.38	7376.70333	OBP8	234	9.00E-74	73.76%	AXO78386.1	*Xylotrechus quadripes*
SbifOBP8	TRINITY_DN14187_c0_g1	YES	NO	15256.593	10593.8267	minus-C OBP4	140	7.00E-41	76.74%	ADD82417.1	*Batocera horsfieldi*
SbifOBP9	TRINITY_DN13481_c0_g2	YES	NO	2.483333	0.62	OBP12	224	1.00E-68	77.61%	AXO78390.1	*Xylotrechus quadripes*
SbifOBP10	TRINITY_DN10230_c0_g2	YES	NO	1.24	0.406667	minus-C OBP3	161	5.00E-48	72.36%	ADD82416.1	*Batocera horsfieldi*
SbifOBP11	TRINITY_DN6797_c0_g1	YES	1-16	0.68	0.6	OBP14	124	6.00E-33	42.47%	APC94287.1	*Pyrrhalta aenescens*
SbifOBP12	TRINITY_DN15685_c2_g1	YES	1-19	2.766667	3.156667	OBP16	139	7.00E-38	47.69%	AXO78394.1	*Xylotrechus quadripes*
SbifOBP13	TRINITY_DN13911_c0_g1	NO	NO	18.203333	23.346667	OBP16	181	2.00E-53	62.79%	AXO78394.1	*Xylotrechus quadripes*
SbifOBP14	TRINITY_DN17387_c2_g1	YES	1-18	4.05	2.266667	OBP16	124	3.00E-33	46.03%	AXO78394.1	*Xylotrechus quadripes*
SbifOBP15	TRINITY_DN10340_c0_g1	YES	1-19	11.6	0.033333	OBP16	104	3.00E-25	40.46%	AXO78394.1	*Xylotrechus quadripes*
SbifOBP16	TRINITY_DN15078_c0_g1	YES	1-20	27.533333	21.956667	OBP16	193	7.00E-56	68.29%	APC94207.1	*Pyrrhalta maculicollis*
SbifOBP17	TRINITY_DN11727_c1_g1	YES	1-17	6.933333	0.653333	OBP17	182	4.00E-56	60.45%	AXO78395.1	*Xylotrechus quadripes*
SbifOBP18	TRINITY_DN14920_c1_g2	YES	1-16	3698.3133	5002.88667	OBP19	140	6.00E-37	60.47%	AXO78397.1	*Xylotrechus quadripes*
SbifOBP19	TRINITY_DN11079_c2_g1	YES	1-18	2.45	0.406667	OBP19	169	1.00E-47	37.31%	AIX97034.1	*Monochamus alternatus*
SbifOBP20	TRINITY_DN17630_c0_g3	YES	NO	1166.4167	649.56	OBP20	138	1.00E-38	56.48%	AXO78398.1	*Xylotrechus quadripes*
SbifOBP21	TRINITY_DN17630_c1_g1	YES	NO	4230.08	7053.30667	OBP20	132	3.00E-37	56.73%	AXO78398.1	*Xylotrechus quadripes*
SbifOBP22	TRINITY_DN18674_c0_g1	YES	1-19	146.08667	113.563333	OBP20	166	2.00E-47	56.35%	AXO78398.1	*Xylotrechus quadripes*
SbifOBP23	TRINITY_DN14306_c0_g1	NO	1-18	9886.1367	6214.8	OBP21	202	1.00E-63	71.32%	AXO78399.1	*Xylotrechus quadripes*
SbifOBP24	TRINITY_DN11180_c0_g1	YES	1-15	3272.3167	1949.79667	OBP21	122	2.00E-32	47.06%	AIX97067.1	*Dastarcus helophoroides*
SbifOBP25	TRINITY_DN10122_c0_g1	YES	1-16	144.66333	287.97	OBP23	92	9.00E-21	49.45%	AXO78401.1	*Xylotrechus quadripes*
SbifOBP26	TRINITY_DN12061_c0_g1	YES	1-19	5.093333	3.45	OBP	268	4.00E-85	76.54%	AUF72969.1	*Anoplophora chinensis*

SbifOBP27	TRINITY_DN10210_c0_g1	YES	1-20	4.22	8.986667	OBP	268	1.00E-36	41.73%	AUF72979.1	*Anoplophora chinensis*
SbifOBP28	TRINITY_DN10898_c0_g1	YES	1-20	94.226667	74.913333	OBP	170	2.00E-49	64.49%	AUF72954.1	*Anoplophora chinensis*
SbifOBP29	TRINITY_DN10898_c0_g2	YES	1-19	2.33	1.693333	OBP	275	7.00E-39	45.04%	AUF72979.1	*Anoplophora chinensis*
SbifOBP30	TRINITY_DN7346_c0_g1	YES	1-20	2.696667	2.966667	OBP	167	5.00E-49	64.35%	AUF72951.1	*Anoplophora chinensis*
SbifOBP31	TRINITY_DN12791_c2_g1	NO	NO	20.29	38.543333	OBP8	161	5.00E-49	74.47%	AXO78386.1	*Xylotrechus quadripes*
SbifOBP32	TRINITY_DN15874_c0_g3	NO	1-21	293.11333	5533.84667	OBP1	103	3.00E-26	62.34%	AXO78379.1	*Xylotrechus quadripes*

Table S2. Sequence information and best blasts match information of chemosensory proteins (CSPs)

Number	Gene ID	Complete ORF	Signal peptide	FPKM		Blastx match					
				F	M	Name	score	E value	Ident	Accession	Species
SbifCSP1	TRINITY_DN12712_c1_g1	YES	1-19	271.50333	283.99	CSP1	166	1.00E-50	59.02%	AUF72992.1	*Anoplophora chinensis*
SbifCSP2	TRINITY_DN13744_c0_g3	YES	1-19	43.326667	52.23	CSP10	169	2.00E-51	63.64%	AIX97085.1	*Monochamus alternatus*
SbifCSP3	TRINITY_DN10413_c2_g3	YES	1-21	970.82333	1446.63	CSP7	158	2.00E-47	58.68%	AIX97047.1	*Monochamus alternatus*
SbifCSP4	TRINITY_DN11041_c1_g5	YES	1-17	13499.387	16887.52	CSP8	192	3.00E-60	77.69%	AHE13803.1	*Lissorhoptrus oryzophilus*
SbifCSP5	TRINITY_DN12342_c0_g1	YES	1-18	3.013333	2.13	CSP11	86.7	4.00E-17	86.36%	AIX97086.1	*Monochamus alternatus*
SbifCSP6	TRINITY_DN16899_c0_g1	YES	1-18	8132.24	15807.49	CSP5	171	5.00E-51	67.77%	AIX97045.1	*Monochamus alternatus*
SbifCSP7	TRINITY_DN18407_c1_g1	YES	1-16	627.16	564.68667	CSP	164	2.00E-45	63.03%	AEC04844.1	*Batocera horsfieldi*
SbifCSP8	TRINITY_DN18666_c0_g1	YES	1-21	0.71	1.576667	CSP4	160	6.00E-48	80.43%	AIX97044.1	*Monochamus alternatus*

Table S3. Sequence information and best blasts match information of odorant receptors (ORs)

Number	Gene ID	Complete ORF	FPKM		Blastx match					
			F	M	Name	score	E value	Ident	Accession	Species
SbifOR1	TRINITY_DN10023_c0_g1	YES	18.8	12.77667	OR44	465	1.00E-157	56.39%	AVN97856.1	*Anoplophora chinensis*
SbifOR2	TRINITY_DN10182_c0_g1	YES	1.95	1.2	OR49b	466	6.00E-161	65.46%	XP_018570955.1	*Anoplophora glabripennis*
SbifOR3	TRINITY_DN10248_c0_g1	YES	24.333333	14.90667	OR13a	339	4.00E-109	45.24%	XP_018579026.2	*Anoplophora glabripennis*
SbifOR4	TRINITY_DN10639_c0_g1	YES	4.993333	2.763333	OR49b	495	9.00E-170	55.42%	XP_023026692.1	*Leptinotarsa decemlineata*
SbifOR5	TRINITY_DN10684_c0_g1	YES	2.423333	1.073333	OR	222	6.00E-63	33.15%	AUF73043.1	*Anoplophora chinensis*
SbifOR6	TRINITY_DN10751_c0_g1	YES	3.626667	2.166667	OR29	291	1.00E-91	55.60%	QNH68053.1	*Apriona germari*
SbifOR7	TRINITY_DN10776_c0_g1	YES	5.8	3.59	OR	266	2.00E-81	37.57%	AUF73043.1	*Anoplophora chinensis*
SbifOR8	TRINITY_DN10821_c0_g1	YES	4.2	2.496667	OR49b	303	4.00E-94	40.00%	XP_030759997.1	*Sitophilus oryzae*
SbifOR9	TRINITY_DN10893_c0_g1	YES	15.666667	8.043333	OR63a	147	2.00E-36	36.33%	XP_023016125.1	*Leptinotarsa decemlineata*
SbifOR10	TRINITY_DN11109_c0_g2	NO	0.343333	0.386667	OR92a	180	6.00E-49	36.84%	XP_023311417.1	*Anoplophora glabripennis*
SbifOR11	TRINITY_DN11230_c0_g1	YES	7.17	3.193333	OR40	171	2.00E-45	29.86%	ALR72583.1	*Colaphellus bowringi*
SbifOR12	TRINITY_DN11294_c0_g1	YES	52.18	24.25667	OR4	303	5.00E-95	41.87%	XP_018577142.1	*Anoplophora glabripennis*
SbifOR13	TRINITY_DN11529_c1_g2	YES	1.823333	1.57	OR49b	508	3.00E-177	66.21%	XP_018570955.1	*Anoplophora glabripennis*
SbifOR14	TRINITY_DN11580_c1_g2	YES	3.773333	2.163333	OR36	486	5.00E-166	61.36%	ALR72579.1	*Colaphellus bowringi*
SbifOR15	TRINITY_DN11629_c0_g1	YES	1.993333	0.863333	OR48	301	7.00E-94	41.16%	AVN97860.1	*Anoplophora chinensis*
SbifOR16	TRINITY_DN11688_c0_g1	YES	21.723333	14.14333	OR49b	532	0.00E+00	64.58%	XP_023026692.1	*Leptinotarsa decemlineata*
SbifOR17	TRINITY_DN12137_c0_g1	NO	14.306667	4.253333	OR15	157	5.00E-42	38.43%	ALR72560.1	*Colaphellus bowringi*
SbifOR18	TRINITY_DN12137_c0_g2	YES	2.506667	0.506667	OR15	167	2.00E-43	37.05%	ALR72560.1	*Colaphellus bowringi*
SbifOR19	TRINITY_DN12137_c0_g3	NO	13.106667	2.166667	OR15	158	3.00E-42	34.29%	ALR72560.1	*Colaphellus bowringi*
SbifOR20	TRINITY_DN12249_c0_g1	YES	17.046667	9.273333	OR24	330	4.00E-106	41.60%	ALR72568.1	*Colaphellus bowringi*
SbifOR21	TRINITY_DN12249_c0_g3	NO	2.893333	2.216667	OR40	152	2.00E-39	44.89%	QNH68064.1	*Apriona germari*
SbifOR22	TRINITY_DN12470_c0_g1	YES	1.73	1.39	OR24	418	4.00E-141	49.87%	ALR72568.1	*Colaphellus bowringi*
SbifOR23	TRINITY_DN12470_c0_g2	YES	1.56	0.743333	OR22	394	1.00E-130	48.42%	AVN97834.1	*Anoplophora chinensis*
SbifOR24	TRINITY_DN12654_c1_g1	YES	6.07	3.2	OR19	323	2.00E-100	41.32%	AVN97831.1	*Anoplophora chinensis*
SbifOR25	TRINITY_DN12759_c1_g1	NO	3.836667	2.423333	OR40	185	3.00E-50	35.15%	ALR72583.1	*Colaphellus bowringi*
SbifOR26	TRINITY_DN12759_c1_g2	YES	11.13	7.543333	OR40	179	6.00E-48	35.56%	ALR72583.1	*Colaphellus bowringi*
SbifOR27	TRINITY_DN12986_c0_g1	YES	6.476667	3.183333	OR1	429	1.00E-144	55.16%	XP_023310033.1	*Anoplophora glabripennis*
SbifOR28	TRINITY_DN13248_c0_g2	YES	0.346667	0.496667	OR36	129	4.00E-33	41.45%	AVN97848.1	*Anoplophora chinensis*

SbifOR29	TRINITY_DN13248_c0_g7	YES	2.65	0.683333	OR63a	214	2.00E-60	36.66%	XP_023016125.1	*Leptinotarsa decemlineata*
SbifOR30	TRINITY_DN13330_c0_g1	YES	5.376667	2.766667	OR92a	317	5.00E-97	40.86%	XP_023311417.1	*Anoplophora glabripennis*
SbifOR31	TRINITY_DN13442_c0_g1	NO	4.94	2.99	OR4	285	5.00E-91	54.40%	XP_018577142.1	*Anoplophora glabripennis*
SbifOR32	TRINITY_DN13865_c2_g8	YES	18.463333	11.25	OR94b	413	2.00E-138	48.32%	XP_023020469.1	*Leptinotarsa decemlineata*
SbifOR33	TRINITY_DN14029_c0_g1	YES	4.053333	0.416667	OR13	77	6.00E-12	56.92%	APC94240.1	*Pyrrhalta maculicollis*
SbifOR34	TRINITY_DN14553_c0_g1	YES	1.673333	0.446667	OR85b	409	1.00E-137	54.13%	XP_018564120.1	*Anoplophora glabripennis*
SbifOR35	TRINITY_DN14968_c0_g1	YES	3.05	1.13	OR2	320	3.00E-103	42.26%	XP_018567969.1	*Anoplophora glabripennis*
SbifOR36	TRINITY_DN14968_c0_g3	YES	1.153333	1.59	OR2	101	2.00E-21	46.32%	XP_018567969.1	*Anoplophora glabripennis*
SbifOR37	TRINITY_DN15011_c0_g1	YES	4.926667	1.32	OR40	166	2.00E-43	32.58%	ALR72583.1	*Colaphellus bowringi*
SbifOR38	TRINITY_DN15011_c0_g2	YES	6.443333	2.633333	OR40	163	5.00E-42	30.88%	ALR72583.1	*Colaphellus bowringi*
SbifOR39	TRINITY_DN15067_c0_g2	YES	2.3	1.326667	OR20	142	4.00E-35	32.62%	ALR72565.1	*Colaphellus bowringi*
SbifOR40	TRINITY_DN15067_c0_g5	YES	1.383333	0.776667	OR20	151	4.00E-38	34.91%	ALR72565.1	*Colaphellus bowringi*
SbifOR41	TRINITY_DN15114_c2_g1	YES	1.993333	2.223333	OR24	358	6.00E-115	45.07%	ALR72568.1	*Colaphellus bowringi*
SbifOR42	TRINITY_DN15319_c2_g2	YES	0.276667	1.023333	OR171	108	8.00E-22	27.30%	EFA01342.2	*Tribolium castaneum*
SbifOR43	TRINITY_DN15321_c0_g2	YES	2.396667	1.52	OR1	497	3.00E-169	63.32%	XP_023310030.1	*Anoplophora glabripennis*
SbifOR44	TRINITY_DN15321_c0_g3	NO	2.36	0.79	OR1	84	9.00E-17	51.32%	XP_023310030.1	*Anoplophora glabripennis*
SbifOR45	TRINITY_DN15703_c0_g1	NO	5.45	2.89	OR63a	103	4.00E-22	32.57%	XP_023016125.1	*Leptinotarsa decemlineata*
SbifOR46	TRINITY_DN15703_c0_g3	YES	3.966667	2.393333	OR19	193	2.00E-53	33.87%	AVN97831.1	*Anoplophora chinensis*
SbifOR47	TRINITY_DN15703_c0_g4	YES	10.95	6.253333	OR63a	61.2	3.00E-08	46.15%	XP_023016125.1	*Leptinotarsa decemlineata*
SbifOR48	TRINITY_DN15844_c0_g4	YES	3.223333	1.42	OR6	290	2.00E-90	38.08%	ALR72551.1	*Colaphellus bowringi*
SbifOR49	TRINITY_DN15895_c0_g2	YES	0.026667	0.83	OR63a	150	1.00E-38	32.14%	XP_023016125.1	*Leptinotarsa decemlineata*
SbifOR50	TRINITY_DN16354_c0_g1	YES	8.37	6.113333	OR63a	187	2.00E-51	33.61%	XP_023016125.1	*Leptinotarsa decemlineata*
SbifOR51	TRINITY_DN16426_c1_g2	YES	3.526667	1.596667	OR13a	273	3.00E-83	42.51%	XP_018579026.2	*Anoplophora glabripennis*
SbifOR52	TRINITY_DN16429_c1_g1	YES	4.173333	1.853333	OR49b	477	5.00E-161	57.11%	XP_018560835.2	*Anoplophora glabripennis*
SbifOR53	TRINITY_DN16455_c0_g1	YES	0.553333	0.603333	OR8	73.2	1.00E-10	64.00%	AVN97820.1	*Anoplophora chinensis*
SbifOR54	TRINITY_DN16455_c0_g2	NO	2.893333	1.866667	OR46a	248	5.00E-76	46.72%	XP_023012678.1	*Leptinotarsa decemlineata*
SbifOR55	TRINITY_DN17010_c0_g1	YES	17.183333	18.84667	OR18	426	3.00E-143	51.79%	APC94230.1	*Pyrrhalta maculicollis*
SbifOR56	TRINITY_DN17611_c2_g4	YES	1.066667	0.343333	OR92a	418	3.00E-139	49.88%	XP_008192407.1	*Tribolium castaneum*
SbifOR57	TRINITY_DN17800_c1_g4	NO	0.586667	0.753333	OR6	128	7.00E-32	44.38%	ALR72551.1	*Colaphellus bowringi*
SbifOR58	TRINITY_DN17800_c1_g6	YES	0.796667	0.156667	OR14	77.8	3.00E-15	69.81%	QNH68040.1	*Apriona germari*
SbifOR59	TRINITY_DN17845_c1_g1	NO	11.78	1.086667	OR4	141	1.00E-39	53.72%	XP_018575345.1	*Anoplophora glabripennis*
SbifOR60	TRINITY_DN17845_c1_g2	YES	32.576667	16.41	OR15	279	3.00E-86	38.01%	ALR72560.1	*Colaphellus bowringi*
SbifOR61	TRINITY_DN17845_c1_g3	YES	10.826667	3.683333	OR4	157	4.00E-44	49.32%	XP_018575345.1	*Anoplophora glabripennis*

SbifOR62	TRINITY_DN19158_c0_g1	NO	1.843333	0	OR51	80.5	3.00E-15	38.83%	AVN97863.1	*Anoplophora chinensis*
SbifOR63	TRINITY_DN20306_c0_g1	NO	0.8	0	OR19	110	2.00E-26	52.69%	AVN97831.1	*Anoplophora chinensis*
SbifOR64	TRINITY_DN3146_c0_g1	YES	0	0.863333	OR34	152	1.00E-40	52.63%	ALR72577.1	*Colaphellus bowringi*
SbifOR65	TRINITY_DN6436_c0_g1	NO	0.753333	0.12	OR28	76.3	8.00E-14	40.62%	ALR72571.1	*Colaphellus bowringi*
SbifOR66	TRINITY_DN6436_c0_g2	NO	1.116667	0.936667	OR28	91.3	4.00E-19	44.14%	ALR72571.1	*Colaphellus bowringi*
SbifOR67	TRINITY_DN6968_c0_g1	NO	0.54	0.54	OR20	132	2.00E-32	36.76%	ALR72565.1	*Colaphellus bowringi*
SbifOR68	TRINITY_DN9200_c0_g1	YES	1.503333	0.65	OR73	221	2.00E-60	36.48%	EFA05710.2	*Tribolium castaneum*
SbifOR69	TRINITY_DN9655_c0_g1	YES	1.646667	0.943333	OR4	351	5.00E-114	45.54%	XP_018577142.1	*Anoplophora glabripennis*
SbifOR70	TRINITY_DN9808_c0_g1	YES	1.143333	0.71	OR9a	238	7.00E-70	35.47%	XP_023027006.1	*Leptinotarsa decemlineata*
SbifORco	TRINITY_DN17522_c0_g2	YES	206.87	83.61667	ORco	842	0.00E+00	91.77%	ALR72547.1	*Colaphellus bowringi*

Table S4. Sequence information and best blasts match information of gustatory receptors (GRs)

Number	Gene ID	Complete ORF	FPKM		Blastx match					
			F	M	Name	score	E value	Ident	Accession	Species
SbifGR1	TRINITY_DN4443_c0_g1	NO	0.406667	1.176667	GR	57.4	2.00E-07	37.04%	AUF73052.1	*Anoplophora chinensis*
SbifGR2	TRINITY_DN4443_c0_g2	YES	0	0.586667	GR7	90.5	2.00E-20	41.96%	APC94254.1	*Pyrrhalta maculicollis*
SbifGR3	TRINITY_DN19652_c0_g1	YES	0	0.366667	GR	176	6.00E-51	88.54%	AUF73061.1	*Anoplophora chinensis*
SbifGR4	TRINITY_DN12702_c1_g4	YES	0.613333	1.336667	GR5	103	2.00E-25	57.50%	AVN97870.1	*Anoplophora chinensis*
SbifGR5	TRINITY_DN12702_c1_g6	YES	1.096667	0.77	GR3	100	4.00E-21	36.61%	APC94248.1	*Pyrrhalta maculicollis*
SbifGR6	TRINITY_DN12702_c1_g7	YES	0.376667	0.65	GR5	110	7.00E-29	59.26%	AVN97870.1	*Anoplophora chinensis*
SbifGR7	TRINITY_DN11648_c0_g1	YES	0.203333	0.4	GR	732	0	55.10%	AUF73068.1	*Anoplophora chinensis*
SbifGR8	TRINITY_DN18392_c0_g9	YES	0.786667	0.886667	GR9	384	1.00E-126	50.82%	AVN97874.1	*Anoplophora chinensis*
SbifGR9	TRINITY_DN4012_c0_g1	NO	0	1.093333	GR	93.6	2.00E-22	88.00%	AUF73054.1	*Anoplophora chinensis*
SbifGR10	TRINITY_DN9768_c0_g1	YES	1.14	1.04	GR5	93.2	1.00E-19	55.70%	AVN97870.1	*Anoplophora chinensis*
SbifGR11	TRINITY_DN8121_c0_g2	NO	0.543333	0.486667	GR8	184	2.00E-55	53.85%	APC94346.1	*Pyrrhalta aenescens*
SbifGR12	TRINITY_DN8121_c0_g1	NO	0.613333	1.313333	GR16	74.3	5.00E-14	56.25%	APC94344.1	*Pyrrhalta aenescens*
SbifGR13	TRINITY_DN10559_c0_g1	YES	1.34	1.253333	GR5	92.4	3.00E-19	51.25%	AVN97870.1	*Anoplophora chinensis*
SbifGR14	TRINITY_DN23022_c0_g1	NO	0	0	GR9	59.7	2.00E-08	42.37%	AVN97874.1	*Anoplophora chinensis*
SbifGR15	TRINITY_DN16190_c1_g4	YES	1.506667	0.933333	GR16	255	6.00E-82	66.28%	AVN97881.1	*Anoplophora chinensis*
SbifGR16	TRINITY_DN14183_c0_g1	YES	8.546667	34.726667	GR6	520	0	64.63%	AVN97871.1	*Anoplophora chinensis*
SbifGR17	TRINITY_DN10945_c0_g1	YES	0.723333	2.056667	GR5	69.7	5.00E-11	39.24%	AVN97870.1	*Anoplophora chinensis*
SbifGR18	TRINITY_DN10969_c0_g1	YES	1.103333	1.173333	GR5	111	5.00E-26	61.73%	AVN97870.1	*Anoplophora chinensis*
SbifGR19	TRINITY_DN19747_c0_g1	YES	4.25	0	GR2	121	4.00E-31	72.97%	NP_001161916.1	*Tribolium castaneum*
SbifGR20	TRINITY_DN14237_c1_g1	YES	0	0.693333	GR64b	59.7	2.00E-08	72.97%	XP_023312177.1	*Anoplophora glabripennis*
SbifGR21	TRINITY_DN14237_c1_g2	YES	0	1.246667	GR17	38.1	2.50E+00	50.00%	AVN97882.1	*Anoplophora chinensis*
SbifGR22	TRINITY_DN9156_c0_g2	YES	0.473333	0.343333	GR11	85.5	6.00E-16	43.53%	APC94336.1	*Pyrrhalta aenescens*
SbifGR23	TRINITY_DN9079_c0_g1	NO	0.706667	1.24	GR3	85.1	2.00E-14	34.42%	APC94333.1	*Pyrrhalta aenescens*
SbifGR24	TRINITY_DN14519_c1_g1	YES	1.59	2.133333	GR9	284	3.00E-86	42.02%	AVN97874.1	*Anoplophora chinensis*
SbifGR25	TRINITY_DN5360_c0_g1	NO	0	0	GR43a	59.3	2.00E-08	51.11%	XP_023021809.1	*Leptinotarsa decemlineata*
SbifGR26	TRINITY_DN19563_c0_g1	NO	0	1.673333	GR	90.9	1.00E-19	54.93%	AUF73061.1	*Anoplophora chinensis*
SbifGR27	TRINITY_DN8587_c0_g1	YES	0.576667	0.703333	GR5	107	3.00E-24	60.49%	AVN97870.1	*Anoplophora chinensis*
SbifGR28	TRINITY_DN9635_c0_g1	NO	1.606667	0.836667	GR24	582	0	71.23%	XP_023022354.1	*Leptinotarsa decemlineata*

SbifGR29	TRINITY_DN5139_c0_g1	YES	1.526667	1.146667	GR68a	55.1	2.00E-06	53.19%	XP_018567270.1	*Anoplophora glabripennis*
SbifGR30	TRINITY_DN20110_c0_g1	YES	0.726667	0	GR50	44.3	5.00E-04	58.33%	KAE8748081.1	*Frankliniella occidentalis*
SbifGR31	TRINITY_DN19747_c0_g1	YES	4.25	0	GR2	121	4.00E-31	72.97%	NP_001161916.1	*Tribolium castaneum*
SbifGR32	TRINITY_DN9635_c0_g1	NO	1.606667	0.836667	GR3	476	3.00E-160	63.13%	EFA04709.2	*Tribolium castaneum*
SbifGR33	TRINITY_DN11515_c0_g1	YES	1.283333	1.963333	GR2	328	6.00E-78	35.11%	CAL23135.2	*Tribolium castaneum*
SbifGR34	TRINITY_DN1421_c0_g1	NO	1.643333	2.52	GR68a	152	6.00E-41	43.22%	XP_018567270.1	*Anoplophora glabripennis*

Table S5. Sequence information and best blasts match information of ionotropic receptors (IRs)

Number	Gene ID	Complete ORF	FPKM		Blastx match					
			F	M	Name	score	E value	Ident	Accession	Species
SbifIR1	TRINITY_DN7048_c0_g1	YES	0.556667	0.353333	IR	432	1.00E-147	0.8361	AUF73080.1	*Anoplophora chinensis*
SbifIR2	TRINITY_DN7048_c0_g2	YES	0.25	1.01	IR	134	3.00E-34	0.776	AUF73080.1	*Anoplophora chinensis*
SbifIR3	TRINITY_DN14761_c2_g1	YES	55.143333	26.313333	IR25a	410	3E-130	0.7843	XP_023311227.1	*Anoplophora glabripennis*
SbifIR4	TRINITY_DN14351_c1_g1	YES	0.77	0.866667	IR93a	243	2E-72	0.707	XP_018576792.1	*Anoplophora glabripennis*
SbifIR5	TRINITY_DN14351_c1_g2	NO	0.62	0.473333	IR93a	695	0	0.8109	XP_018576792.1	*Anoplophora glabripennis*
SbifIR6	TRINITY_DN14351_c1_g3	NO	0.593333	0.28	IR93a	306	4E-96	0.7892	XP_018576793.1	*Anoplophora glabripennis*
SbifIR7	TRINITY_DN6042_c0_g2	NO	0.093333	0.413333	IR	279	8E-93	0.9643	QBB73025.1	*Protaetia brevitarsis*
SbifIR8	TRINITY_DN16165_c0_g1	YES	6.48	5.803333	IR	1302	0	0.6878	AUF73087.1	*Anoplophora chinensis*
SbifIR9	TRINITY_DN10973_c0_g2	NO	2.903333	1.67	IR1	53.9	0.000006	0.7333	AVN97883.1	*Anoplophora chinensis*
SbifIR10	TRINITY_DN17609_c0_g1	YES	3.26	1.36	IR	328	6E-100	0.4541	AUF73087.1	*Anoplophora chinensis*
SbifIR11	TRINITY_DN17609_c1_g1	YES	2.88	2.266667	IR	416	2E-130	0.4022	AUF73087.1	*Anoplophora chinensis*
SbifIR12	TRINITY_DN17589_c0_g2	YES	26.743333	16.49	IR25a	1000	0	0.8732	XP_018574744.1	*Anoplophora glabripennis*
SbifIR13	TRINITY_DN17589_c0_g3	YES	0	4.266667	IR25a	125	1E-31	0.803	XP_018574744.1	*Anoplophora glabripennis*
SbifIR14	TRINITY_DN16098_c0_g1	YES	3.3	2.13	IR25a	898	0	66.77%	XP_023311227.1	*Anoplophora glabripennis*
SbifIR15	TRINITY_DN15458_c0_g1	YES	6.26	5.756667	IR	1242	0	0.6563	AUF73077.1	*Anoplophora chinensis*
SbifIR16	TRINITY_DN6569_c0_g1	YES	0.87	0.533333	IR40a	275	4E-86	0.7735	XP_023310509.1	*Anoplophora glabripennis*
SbifIR17	TRINITY_DN8575_c0_g1	YES	1.543333	0.746667	IR21a	962	0	0.6446	XP_023313060.1	*Anoplophora glabripennis*
SbifIR18	TRINITY_DN17849_c0_g2	YES	10.403333	13.45	IR	107	3E-24	0.4762	AUF73085.1	*Anoplophora chinensis*

Table S6. Sequence information and best blasts match information of sensory neuron membrane proteins (SNMPs)

Number	Gene ID	Complete ORF	FPKM		Blastx match					
			F	M	Name	sco re	E value	Ident	Accession	Species
SbifSNMP1-like	TRINITY_DN16438_c0_g1	YES	1.2	0.436667	SNMP	404	1.00E-134	74.21%	AUF73093.1	*Anoplophora chinensis*
SbifSNMP2a	TRINITY_DN15938_c0_g2	YES	4.24333	1.09	SNMP2	629	0	62.04%	XP_018566911.1	*Anoplophora glabripennis*
SbifSNMP2b	TRINITY_DN16673_c0_g1	YES	1.35	0.64	SNMP3	648	0.00E+00	59.12%	ALR72545.1	*Colaphellus bowringi*
