# Supplementary material for: Antennal transcriptome analysis of olfactory genes and tissue expression profiling of odorant binding proteins in Semanotus bifasciatus (cerambycidae: coleoptera)
Source: BMC Genomics. 2022 Jun 22;23:461. doi: 10.1186/s12864-022-08655-w (PMC9219211; doi:10.1186/s12864-022-08655-w)

Fig. S1. Expression stability values for seven reference genes calculated by geNorm software among different treatments

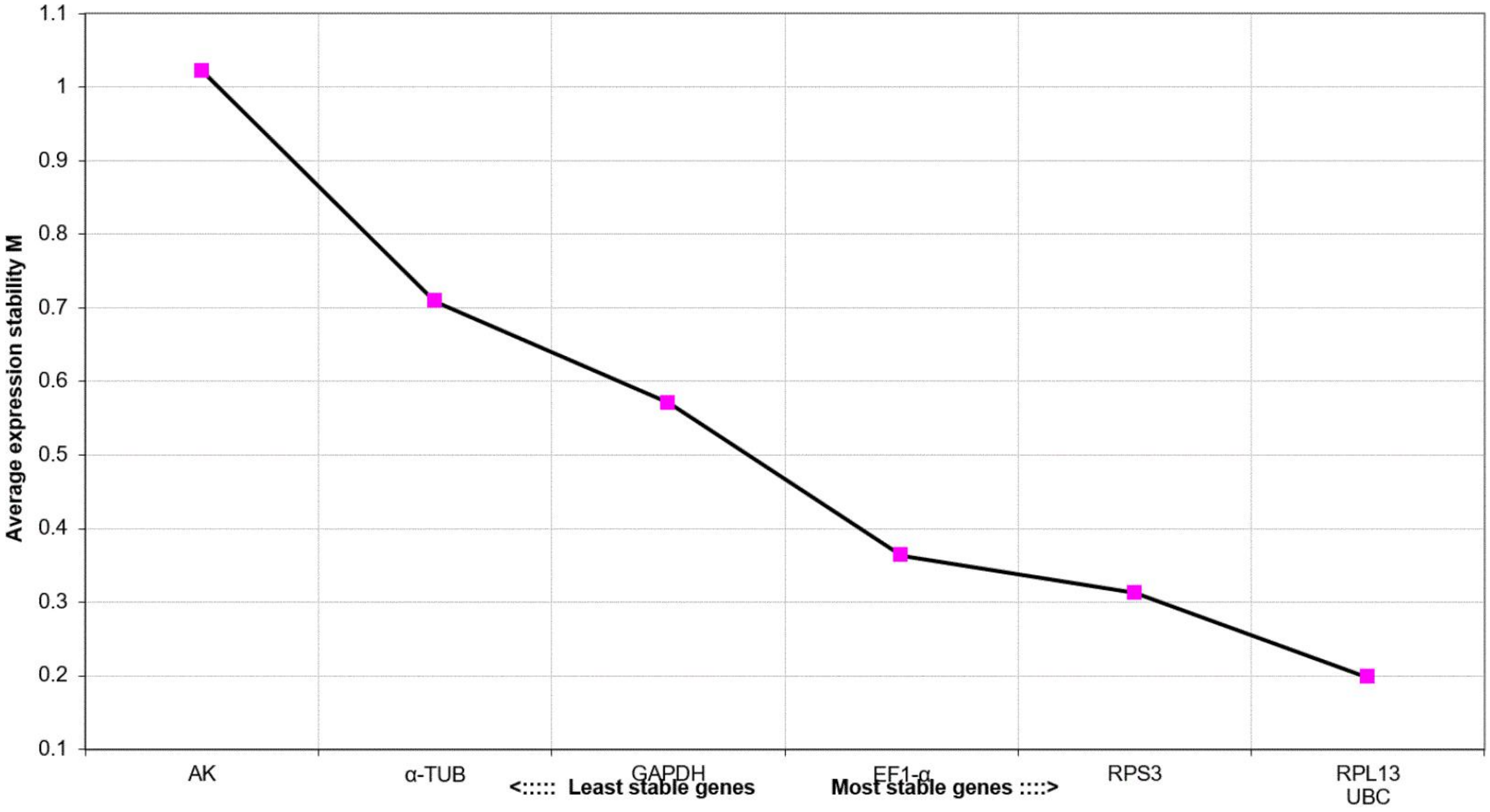

Fig. S2. Pairwise variation (Vn n + 1) between normalization factors after stepwise inclusion of stable reference genes from the most stably expressed genes

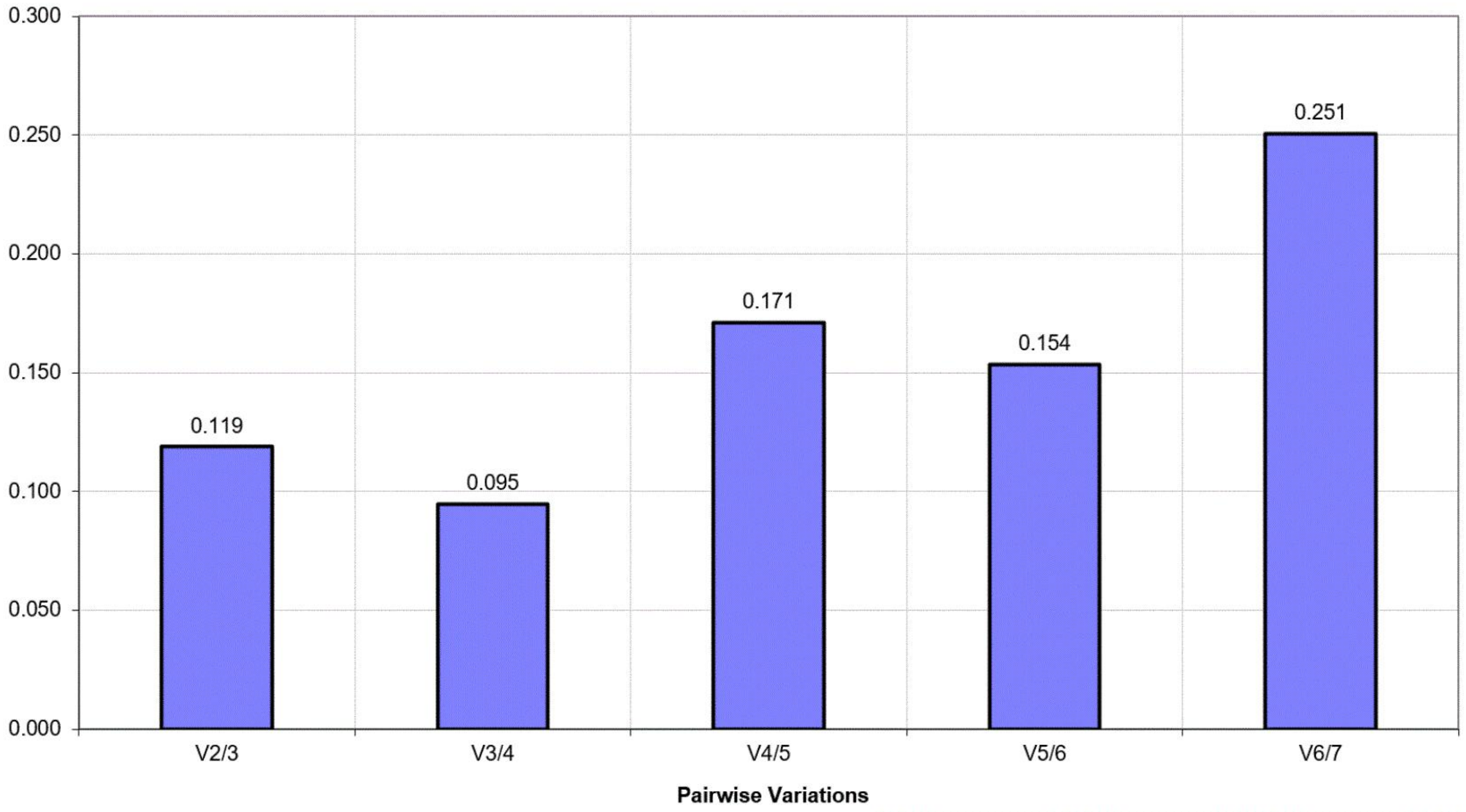

Fig. S3. The ranking order of the expression stability value of candidate reference genes in dif-ferent tissues of S. bifasciatus calculated by NormFinder

Gene name	Stability value
α-TUB	0.713
GAPDH	0.416
RPL13	0.192
RPS3	0.311
UBC	0.069
EF1-α	0.241
AK	1.206

Best gene	UBC

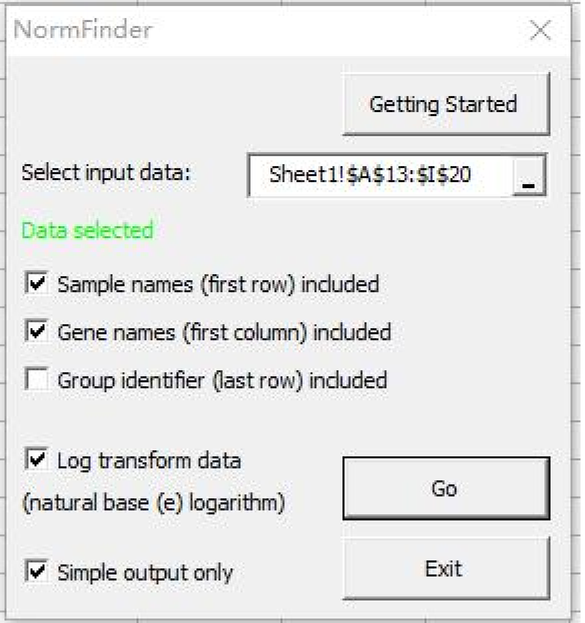

Supplement: Supplementary file 2 — Additional file 2: Fig. S1. Expression stability values for seven reference genes calculated by geNorm software among different treatments. Fig. S2. Pairwise variation (Vn /n + 1) between normalization factors after stepwise inclusion of stable reference genes from the most stably expressed genes. Fig. S3. The ranking order of the expression stability value of candidate reference genes in different tissues of S. bifasciatus calculated by NormFinder. [file 12864_2022_8655_MOESM2_ESM.pdf]
