# Supplementary figures and images for: Antennal transcriptome analysis of olfactory genes and tissue expression profiling of odorant binding proteins in Semanotus bifasciatus (cerambycidae: coleoptera)
Source: BMC Genomics. 2022 Jun 22;23:461. doi: 10.1186/s12864-022-08655-w (PMC9219211; doi:10.1186/s12864-022-08655-w)

A

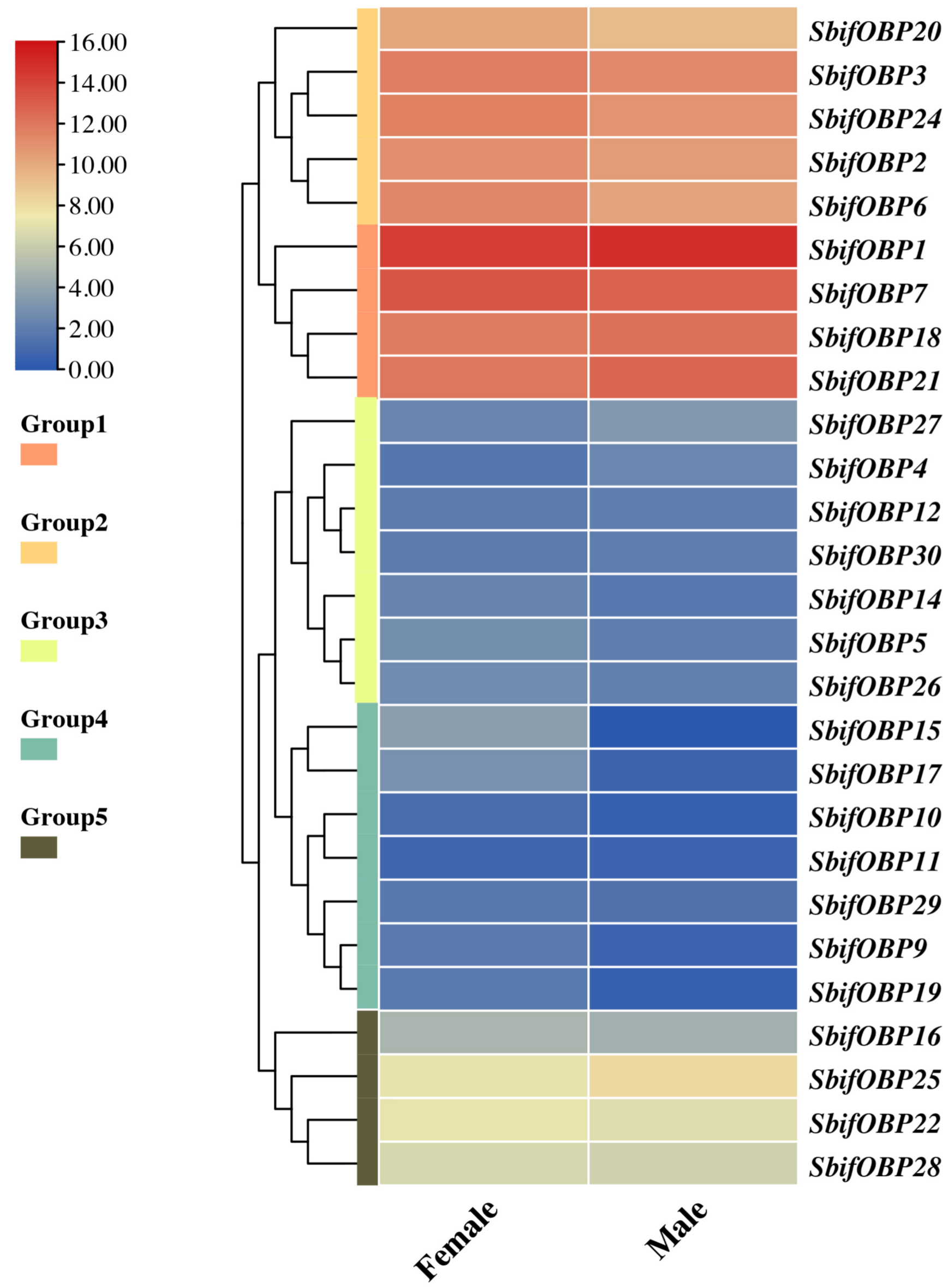

B

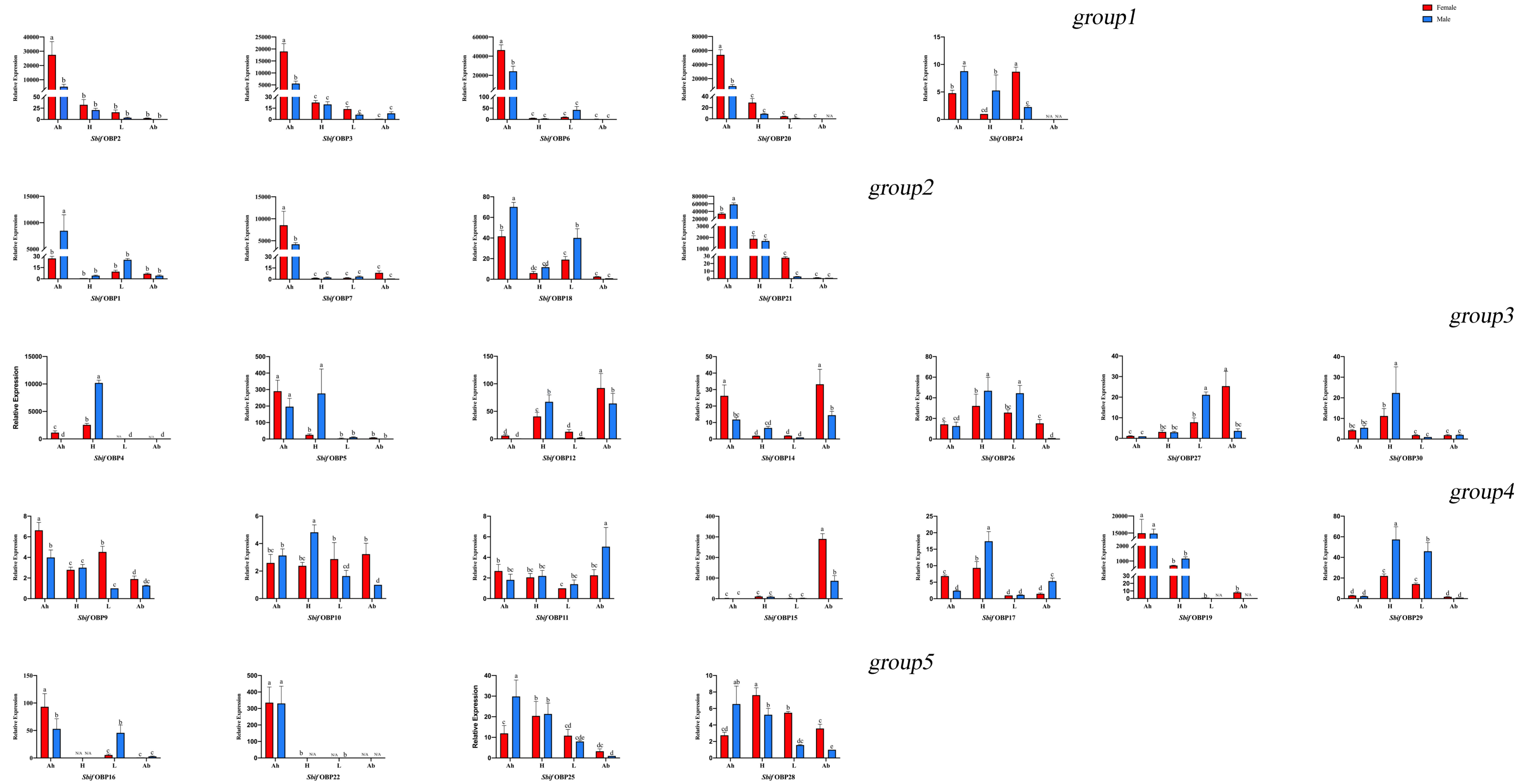

Supplement: Supplementary file 3 — Additional file 3: Fig. S1. Relative expression levels of SbifOBPs in different genders and tissues. (A) Heat-plot of FPKM values for SbifOBPs in female antennae (FAn) and male antennae (MAn). Relative expression levels are indicated by a 8-grade color scale. (B) Expression patterns of candidate OBPs in S. bifasciatus. An: antennae; H: head; L: legs; AB: tail of the abdomen. The letters over the error bars (a-e) denote a significant difference (P<0.05), and "N/a" indicates that the corresponding expression level was below the limit of detection. The genes were divided into group1-5 according to the expression level. [file 12864_2022_8655_MOESM3_ESM.pdf]
