# Supplementary material for: Antennal transcriptome analysis of olfactory genes and tissue expression profiling of odorant binding proteins in Semanotus bifasciatus (cerambycidae: coleoptera)
Source: BMC Genomics. 2022 Jun 22;23:461. doi: 10.1186/s12864-022-08655-w (PMC9219211; doi:10.1186/s12864-022-08655-w)
Supplement: Supplementary file 4 — Additional file 4. Alignment of the S. bifasciatus OBPs. [file 12864_2022_8655_MOESM4_ESM.pdf]

Classic OBP

```
SbifOBP4     .MKFLLLVSLIF..AVSNALDK.......EFVEKMKMKMQ     30
SbifOBP11    .MKVLLLLAYVF..VATHAMDK.......EFAAKFVQEVQ     30
SbifOBP2     MLKINVFIVVAICFALSRLVVA.......VVPDDMKQKMQ     33
SbifOBP3     MLKIRASVFVIVCIALSR...........ANLDELKPLFE     29
SbifOBP5     ..MCKKFVLLLVGMYLPLN.MG.......LTKEELEVLGE     30
SbifOBP6     ...MKYCVLVFLCMAIPSV.IC.......VS.DEIKKLIE     28
SbifOBP7     .MKCVFILYLFF.VIHDVAYSA.......MTEKQLNATKK     31
SbifOBP16    .MKCFILVVIFSNLLLTEVKSA.......MTEKQLIAAVK     32
SbifOBP28    .MRGIATTAVLVLALSAVTLGQ.......NYSDEEKKRII     32
SbifOBP30    .MTFFLVLVYVLSIWTPTAPAA.......STTTISKGPFK     32
SbifOBP26    MVNFIYFFAFCAVLVSTSAEIKENDTNRCDIPPAAPKRVE     40
SbifOBP25    .MVPLSFFLTVTVLVFGVNVHG........FPDMEKFMGM     31
Consensus

SbifOBP4     EVGAECVEKEKPN...........EDDIGLLIAH.QMPTT     58
SbifOBP11    ELGIKCAGEEKPD...........PDDIARMMAH.KLPET     58
SbifOBP2     LLHGTCVPATGIS...........EDVVARAQVG.DFDDD     61
SbifOBP3     SLHKTCVSATGIS...........EDVVARAQKG.DFVNE     57
SbifOBP5     QLHSTCVSKTGVS...........EDLILAVNEDKTFADD     59
SbifOBP6     TLHTTCVAETGVD...........EALIKKTNSEKAIPDD     57
SbifOBP7     LVRNTCMNKAKPT...........SEQVDGLQKG.QFIDD     59
SbifOBP16    MVRNVCLPKTKAS...........VEDVDKMHKG.DWDVD     60
SbifOBP28    KNREECIAETKVN...........PELIDRADSG.DFVDD     60
SbifOBP30    VFIKNCQEESGAT...........KADMEIIKMK.KLPET     60
SbifOBP26    EVINQCQDEIKLAILSEALQALNIHEDKHTRTKRASFNDD     80
SbifOBP25    PGVRECISSSGVD...........QEELKKRPGP.DMSPE     59
Consensus         c

SbifOBP4     HE..GKCVIYCVYKYFNTINEDATINVEGGIEALQPLKEN     96
SbifOBP11    HE..GKCVIYCIYKHYNTVNGDGTINVDAGIKALQPLKEN     96
SbifOBP2     EK..LGCYMNCVMREAGVLDNDEKFNLELVMDFFSE....     95
SbifOBP3     EE..VGCYMRCVIVEMGLF.NEGKLDIERAINSVPE....     90
SbifOBP5     EN..LKCYIKCVMQEGGVMDDEGLIDPAAAREIIPE....     93
SbifOBP6     EK..LKCYLKCIMVQSGCMSDDGIIDEEATIAAVPD....     91
SbifOBP7     RN..LQCYIYCILNTYKLIRKDNSFDWEGGIKALEAN..A     95
SbifOBP16    HT..AMCYMHCALNMYKLIDKDNTLNYDSVIIQIKQ...L     95
SbifOBP28    DK..LKCFTKCFYQKAGFVTEAGALLLDTIKAKIPAN...     95
SbifOBP30    KT..GRCFLQCLFNKAKIM.DDGKFNKQGMVMAFVPSTKG     97
SbifOBP26    ERRIAGCLLQCVYRKMDAVNDKGFPTVEGLVSLYTEG.IT    119
SbifOBP25    YL....CFLKCTAEAVGTLSEDGEIDSENTQGLPMLMN..     93
Consensus          c   c

SbifOBP4     DEELYEKVAAILKKCTSSL...........TIDGDPCNTG    125
SbifOBP11    DEALYNLIVADFKKCAGSI...........SINSDPCETS    125
SbifOBP2     ..SIRDNAYPVMKKCG..I...........LVGANLCETA    120
SbifOBP3     ..EIKTELAPVIRKCG..V...........LVGANECQTA    115
SbifOBP5     ..EYDSDHELLLRSCS..S...........KKGSTTCETA    118
SbifOBP6     ..EHRSKSEPIIRACG..T...........KVGANPCENA    116
SbifOBP7     PPNIAESGSKSIVNCKDAV...........KTTDDKCIAA    124
SbifOBP16    PDRYKESTEKCLDPCKNSA...........VTLDDKCAAA    124
SbifOBP28    ..IDKEKALQVIEKCQ..............QQGKDACETV    119
SbifOBP30    DASKIKQLKELGDVCEKEIG..........GKRPDNCEGV    127
SbifOBP26    KKDYILATVQAVNVCLNNAQKKILVTPQGLEEHGKTCDIA    159
SbifOBP25    ...MPEDTKNEVMECMANVG............KIETCDDM    118
Consensus                  c                     c

SbifOBP4     AKLIECTVLEAKAMGLSEEMLDM.................    148
SbifOBP11    FNLVNCCLVEAKAKGLPKELFDFDI...............    150
SbifOBP2     FLICKCVFEEDPSVYHLP......................    138
SbifOBP3     FLMCKCVFEENPSLYMLA......................    133
SbifOBP5     WLAHKCYAQHP..QYRLL......................    134
SbifOBP6     WLTNKCYMEKAPEDYILI......................    134
SbifOBP7     YEITKCVYDDNPAGYFLP......................    142
SbifOBP16    YEISKCLYFCNPGDYYLP......................    142
SbifOBP28    YLVHKCYFLHTHLPEPKVEESKADGLAPAASDVPSQKESK    159
SbifOBP30    RLVVECVARHGKAYGITFSNSKNTV...............    152
SbifOBP26    YDVFDCVSGEIGKYCGQTP.....................    178
SbifOBP25    IKMLECAPKPPKP...........................    131
Consensus         c

SbifOBP4     .................                           148
SbifOBP11    .................                           150
SbifOBP2     .................                           138
SbifOBP3     .................                           133
SbifOBP5     .................                           134
SbifOBP6     .................                           134
SbifOBP7     .................                           142
SbifOBP16    .................                           142
SbifOBP28    NETKTETKSESQTEAKK                           176
SbifOBP30    .................                           152
SbifOBP26    .................                           178
SbifOBP25    .................                           131
Consensus
```

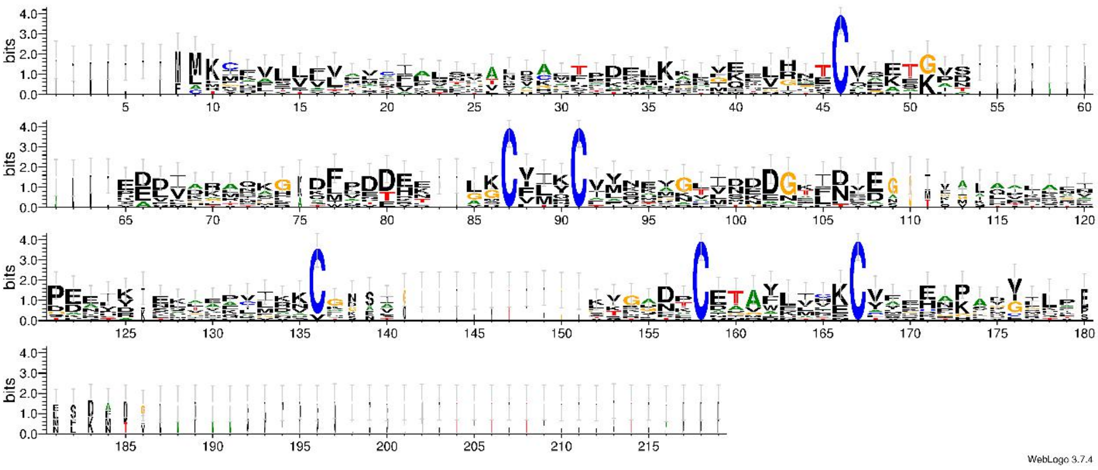
bits
4.0
3.0
2.0
1.0
0.0
5 10 15 20 25 30 35 40 45 50 55 60
65 70 75 80 85 90 95 100 105 110 115 120
125 130 135 140 145 150 155 160 165 170 175 180
185 190 195 200 205 210 215
WebLogo 3.7.4

Minus-C OBP

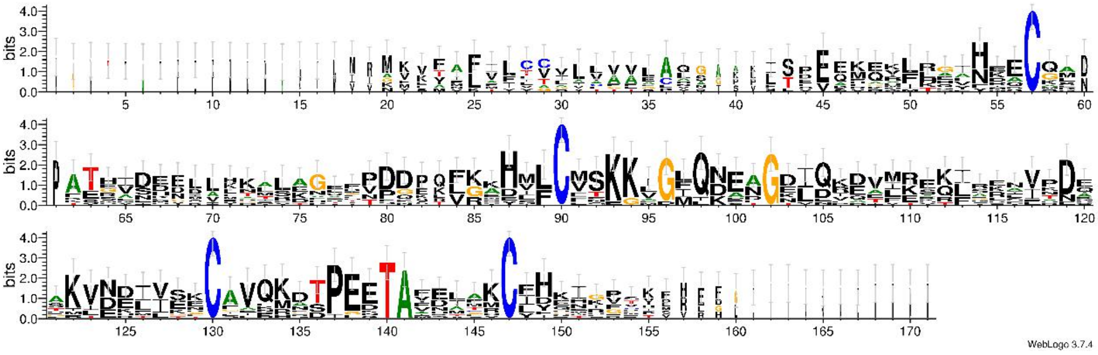
bits
4.0
3.0
2.0
1.0
0.0
5 10 15 20 25 30 35 40 45 50 55 60
65 70 75 80 85 90 95 100 105 110 115 120
125 130 135 140 145 150 155 160 165 170
WebLogo 3.7.4

```
SbifOBP20   ........................................     0
SbifOBP21   ........................................     0
SbifOBP22   ..................MRAFLAIFALYGILVTAECL..    20
SbifOBP27   .................MVVNVEYIFILTCLLVVVLAQ..    21
SbifOBP29   ..................MIGVKYFLMLTTLLVVVEPQSI    22
SbifOBP17   .......................MNFFIICVICFFGAVQA    17
SbifOBP24   ....................MYVTIFVYFTVLTVAQCLS.    19
SbifOBP18   ........................MKTFVAVLFVVALAAA    16
SbifOBP9    MGNTVAVFIFNKRAQVLIRKMKTALVFLCAA.AGVFAQN.    38
SbifOBP10   ........................................     0
SbifOBP14   ....................MKVFVLILCVS.LAALANG.    18
SbifOBP15   ....................MKVFAFILCVGYLALLAHG.    19
SbifOBP12   ....................MKSFALVICVIVLATLAYGG    20
Consensus

SbifOBP20   .......MEYFRGVHRECGM..AEENSQ....TMLDGEVP    27
SbifOBP21   .......MEYFKGLHMECGM..AEQYSE....KMFAGQLP    27
SbifOBP22   ...SPEELKYFREVNRECGM..AEEVID....RALAGDFP    51
SbifOBP27   ..FSSEEVQNLLQYHEECKL..KTNIDDYRVLNILTGTFP    57
SbifOBP29   PKISPEEMQALLQYHKDCKL..ESHASDNLAYGTLAGQFP    60
SbifOBP17   AVITEEQKGKLLEFNKQCME..ETHVDMDIVMKASKGVYT    55
SbifOBP24   ....PEQHAKLTAIFIECSQ..TTGINEDQARAAVEAETT    53
SbifOBP18   KPLSDEAKEKMKAINEECAK..DSGIEEGDLLKVLEEAG.    53
SbifOBP9    ..LLLVEEQQLHHIHDACQANPATRADHNLLHDLSANLDN    76
SbifOBP10   .........MLHHIHDACQADPATSVNHELLHNLSENLDN    31
SbifOBP14   ..LTEEQKERIRGAYKSCQSDPATSIDLELLKTTRDPSKV    56
SbifOBP15   ..ISDEDKQHFRSLNEECQSNPATRLDKEMIEKLRKHEEV    57
SbifOBP12   SLSTVVSRRRITNAHNQCQADPATHVDEEDLKKSLKGIQV    60
Consensus                    c

SbifOBP20   DGEQFRKDLLCVSKKVGMQDEAGNIQVQEMEDQFRRNIPD    67
SbifOBP21   DDEQFKKELLCVSKKMGMLDEAGNLQVQVMEEQFRRNIPD    67
SbifOBP22   DDQQFKNDIFCMFKKIGMQDDEGTLQIEAITEQIRRNVPE    91
SbifOBP27   DDPLLRRHVFCLSKKFGIQNEAGEIQRREVREKLEKLVNN    97
SbifOBP29   EDPRLKKHVFCMSKKIGFQDETGKIQRDVFEEQLQKLVTD   100
SbifOBP17   DDPTFKKHIFCVNKKAGFQNAAGDLQIDTMKAKINSIVKD    95
SbifOBP24   DDPKVKEHVLCFSKKIGFQNEAGDIQLDVMRQKIGEAVPD    93
SbifOBP18   DDSKVKEHMFCFQEKLGIINADGEIQKDVLKEKLVDFFDD    93
SbifOBP9    PQ..VGVHMLCESKGVGLQNENGKLEKEVIRSKISLSIAD   114
SbifOBP10   PQ..VGAHMLCESKGVGLQKPDGELDPEIIRSKISLSVKD    69
SbifOBP14   PN..FGAHSLCVTKKLDLQNENGDINKDTLKSRLAEVLTD    94
SbifOBP15   DKKQLRAYSLCMTKKLGIQKENGDVDKEALKKTLAKAIND    97
SbifOBP12   SG..LGPHMLCMSKKIGLQKSNGDIDRAVFRVRFSEIVRN    98
Consensus             c           g

SbifOBP20   QSKVNDILSNCFVQMNSPEETAYEMTKCIHKIRFS.....   102
SbifOBP21   QSKVNDIVSNCFVQMSSPEETAYEATKCIFKIRFP.....   102
SbifOBP22   ETKVNDILSKCLVQMGTPEETAYEACKCIHGIKHS.....   126
SbifOBP27   SEKIDEIIKTCIVQKDDPEQTAVDIARCYIETIVVKS...   134
SbifOBP29   NDRVEELIELCSVQHDDPEETAMDMAKCYYKILRNTV...   137
SbifOBP17   EKKTNEFISKCALNKDTPENTAFEVAKCFHNISPEKDVLG   135
SbifOBP24   AAMVEEMISKCAVQKATPEDTAFDTSVCLYKMKPV.....   128
SbifOBP18   DAKVEDIITKCAEEKDSPLETAFSLGSCIHQEKGDK....   129
SbifOBP9    PGIVDHLVEECAVPKNTPEKTAINLFMCFDRNGVTYFHEF   154
SbifOBP10   AAKVDRLVKECAIKKRTPEETAVNLFMCLDENGVTYFHEF   109
SbifOBP14   EAKINKTVEDCAVQRATPEETAARLLKCLHDQG..ILRRD   132
SbifOBP15   ETKLNKLVGECGARRDTIGETAEEIMRCFHEHIGRHMREH   137
SbifOBP12   QSKLDDIVEGCAVQKETPEDTAEHIMKCFHQHHPCP....   134
Consensus             c         ta     c

SbifOBP20   ...........                                102
SbifOBP21   ...........                                102
SbifOBP22   ...........                                126
SbifOBP27   ...........                                134
SbifOBP29   ...........                                137
SbifOBP17   L..........                                136
SbifOBP24   ...........                                128
SbifOBP18   ...........                                129
SbifOBP9    ...........                                154
SbifOBP10   ...........                                109
SbifOBP14   ...........                                132
SbifOBP15   GEHHDSDEHHD                                148
SbifOBP12   ...........                                134
Consensus
```
