# Supplementary material for: Antennal transcriptome analysis of olfactory genes and tissue expression profiling of odorant binding proteins in Semanotus bifasciatus (cerambycidae: coleoptera)
Source: BMC Genomics. 2022 Jun 22;23:461. doi: 10.1186/s12864-022-08655-w (PMC9219211; doi:10.1186/s12864-022-08655-w)
Supplement: Supplementary file 5 — Additional file 5. Protein sequences used in phylogenetic trees (downloaded from NCBI). [file 12864_2022_8655_MOESM5_ESM.pdf]

Additional file 5 : Protein sequences used in phylogenetic trees (downloaded from NCBI)

>AchiOBP1
MLIRLGAACGCVLLVLPLVLSISEELQELVDMLHNTCVGETGTSEEAIENAKKGDFADDEK
FKCYLMCIM
VQMACIDEDGIVDVEATIAVIPEEFQDLAAPIIRKCDTQKGSTPCESAWLTHKCYYNENPDA
YFLV

>AchiOBP2
MNLSVTLLYVLLCITSIKGLSEDKFIEAKAEARAACLASTGVSEDLVMDINRDGKFADDEN
LKCYVKCVH
EYLGLMAEDGTMDYEKLIENIPEEFRIKYASRIRACGTIYGSDVCDTAWLTIKCYGENIPKL
PHP

>AchiOBP3
MLTSSESSLFTEALSYSYSCVLRIILLDGTFVYSFVQLGLI

>AchiOBP4
MKTLFVFLCVAILGAVAHHLPETEMRKLKEVHDSCQANPTTHVDEKLLKKLKENVEDKK
VGTHLLCMAVK
VGLLTQDGDLIKNVIRDKIVIATHDLSKVDEVLKKCAVKRETPEKTAVQMLVCFIDNGIHY
HHDL

>AchiOBP5
MNNVVAFCALLVATVSAYNFEDPDFNILLSDDLEELSSGVASFSHPRSRRDDEAVNDKDKC
HHRKRWGEL
CCAEDVVAKMRDVEKDLKRECFKEVVGKDKHDKFDPFNCETMDQRKKQIVCVIECVGK
KKDLLDTEGNPK
EEEFRSFLKESFSSESWLAALQDKVISTCLDEGKNATANRDASDSTSCNPAGIKIAHCLHRE
IQLNCPAD
QIKDEKSCARLQERLKRRDFFHPPPPPGGFDEPDN

>AchiOBP6
MIRTIVIVAFVWIFFSWIPYPLAADSVINKFADTLQSSVKPSLRTCQSQTGATKADLESLKM
RKVPRTKT
GRCFLQCLFNSVKLMDDGKFSKQGMIIAFSPAMKGDLSKIGKLRKLSEVCEKEIGGKKYE
NCEGVQKIVE
CVAKNGRAYGIEFKHDKM

>AchiOBP7
MKTSVAIIVTCVIFSTAKATLDITSEQKAKLDEHKDECKKQSGVNVEILKKLPEGEFPEDPK
LKEFLLCV
SKKTGFQNDAGEIQQAVIIEKLGKALKDPAKAKELTEKCTKQEGSPSEITYKFVICFYNNSS
KHVVLV

>AchiOBP8
MKKRRDSRHCIMNAKVISMRRPQENFTKENYWINP

>AchiOBP9
MKVFATVAVVVVVLVVCCTGQTYTEEERERIRKNREDCITETKVDPNLITRADAGDFVDD
DKLKCFTKCF
YQKAGFVTENGELLLDVIKAKIPASVDKERAVKVVENCKQTGKDACETVYLIHKCYFEYT
QRNNLNKTPE
NAETATEAQAEAKTEAPKESTTKSKA

>AchiOBP10
MKTAAVLCLCVIFFSAVQGFSKEQREATHKECLAQTGADEETVLKAMDGEFADDPKFKSY
LLCFGKKEGF
HNDAGELQKDFMRAKLLELFGDDATVDEMMKCAVEKATPEETAFEGCKCMYAFKNKFV
DYF

>AchiOBP11
MKFCFFLLCSCLVAPYVYSAMTEKQLNATKKLMRNTCQNKAKPTSEQIDAMQKGDFNV
DRNAQCYLLCIL
STYKLLTKENTFDWENGIKALAANAPASVAGPGSATLKNCKDAVKTPSDRCVASIEIAKCI
YDDNPSNYF
LP

>AchiOBP12
MNEVLVNKKLMKGESSSFKCFLHCLFAKYGWMDEEGGFLLHDIKATLEQAEVEIASLEYI
LYKCTALESV
DRCERSYMFTECFWEKMSEQQPSEDQLFYSIE

>AchiOBP13
MQIIYVILNKTEPEKIGSPLGVTIDNSTLLDQTINFGLVSCNKGNLLSDNSDVQVAVGLLET
GLSGHAQH
VGAYYVVIDVLVELFQTAFIGESFRVGLAGIMDLFKVYPFAIGELSQGYCKDST

>AchiOBP14
MKTVFVISMVVVMAISHAEMEKEHQECLDETELTDAELSEFLLGDDAENDAKATKFLMC
IFKKNEALNDE
GHLDVTKAQEAVKHYMTEVVGSEDQQAVDCVQEKDTTEETVLALGKCVEKRKVELSSS
K

>AchiOBP15
MKFLVVLACFIVLSSSLDQDFKEKFMQQMEEYGTKCIDEVHATDADIAELVAHKVPPTSH
EAKCLIFCIH
KSFKMMTEDGNPNTEGVLQLMAPLKETDSDIYEKFLKIAEKCSNSLEKDDDHCVTASNW
AACGINEAKAM
GMPDDLFQM

>AglaOBP2
MKPVIVFACVILGAWAANVLVVEEQNLHHLHDACQSNPATYADHELLHHLAENIDNPQV
GAHMLCESTKV
GLQKENGELDLETIKSKVALSVTDSDKVDRLVQECAIKKKSPEKTAIHLFMCLDKNGVTY
FHEF

>AglaOBP3
MKFAVAIVCLVVVAVARASLTEEQIQKLKGYHKDCAAETGVDTELVTKARKGEFSEDPKF
KDHLFCVAKK
IGFMTADGEIHRDVLKEKLGSAINDDAAAQKLIDECAVKKDTPQATAFDTIQCYYVKTPTH
ISIV

>AglaOBP1
MDNLLFVVVMFTLLTLSMVQAVLDESEFTPKLLEQVKALHDTCASQSGADDGLIGKIKK
GDFVEDPKIKS
YMKCGLTELGVMDDRGEISVDMVPELVPAKYVSESIANTKTCTGKTKDIANLEDRVFAFF
KCYHDLNPGI
FIFF

>AglaOBP16
MFPSVVCAVGSYLIFLTIIQAKEISSGRCDIPPSAPKKVEEIINQCQDEIKLAILSEALQAFNV
NEHSRS
RAKRATFSEDEKRIAGCLLQCVYRKMDAVNEKGFPTVQGLVSLYTEGISQKDYILATVQA
VNTCLIKSQK
KYLITPQSIDENGKTCDIAYDVFDCVSDEIGKYCGQRP

>AglaOBP15
MNNLVAFCALLVAAVSAYNFEDPDFNILLSDDLEELSSGVASFSHPRSRRDDEAVNDKDKC
HHRKRWGEL
CCAEDVMAKMRDVEKDLKRECFKEVVGKDKHEKFDPFNCETMDQRKKQIVCVIQCVG
QKKDLLDTEGNPK
EEEFRSFLKESFSSESWLAALQDKVISTCLDEGKNATANRDASDSTSCNPAGIKIAHCLHRE
IQLNCPAD
QIKDEKSCARLQERLKRRDFFHPPPPPGAFDEPDN

>AglaOBP14

MKFCFFLLCSCLAAPYVYSAMTEKQLNAAKKLMRNTCQNKAKPTSEQIDAMQKGDFNG
DRNAQCYLLCIL
STYKLLTKENTFDWENGIKALAANAPASVAGPGSATLKNCKDAVKTPSDRCVASTEIAKCI
YDDNPSNYF
LP

>AglaOBP13
MIVPTIVILVAVILVMDVDADLTDKLRQKLDLPEVQKCITSTGYIPKGPPEGPSQEFTPEQLC
FFKCIME
EKGLLDSTGNIIQDELNNVPLPIPDDKKNEIKKCAAGAGKIESCEDIQKLLSCLPM

>AglaOBP12
MSSRSIIFFFCLITLAYSKLQLPPDLQEYADELHDLCIKRTGITEDDHIAYDIANNPHDEKLQ
CYIKCLL
MEANWMDKDGVIQYDWIEENIHEGVKDIVLAALRKCKNINEGANLCEKSSHFNACMYD
ADKENWFLV

>AglaOBP11
MKTVFVISMVVIMAISHAEMDKEHQECLDETELTEAEVSEFLLGDDAENDAKATKFLMCI
FKKKEAVNDE
GHFDVTKAQEVVKHYMTEVVGSEDQQALDCVQEKDTTEQTVLALGKCVEKRKVELSSS
K

>AglaOBP10
MRTFVVIISISALVLGRPDENLATINMAHNECQSNPRTYVDEDILDRISGGEKIDNPSVRAHI
LCVTTKL
GVLNEYGEVNRTNLRTVLSRVILNEEKLEENLEKCAVEETDAEEVALVLDKCFWNNLDHD
HNSHIHYHHQ
KT

>AglaOBP9
MVSLNFFVTVSSLFAASVVQAALPQSEYGPQLEALQKNVRAACISTSGVDETAISNVGNG
VFTDEPKIKH
YLTCVLKEGKLVNEKGVFSEKNIAQLFPDKYKEESLTNIKSCIVKVNDITNLEDKIFALFKC
YYHQNPDL
FVFF

>AglaOBP8
MSVKMGTAIFSVVVTAILAISSVQAILKQSDFTPELKKLAANLHVTCVSKTGIDEALIDKVL
NGEFVEEP
KMKAYMTCLLLEGTLIDEKGTPNLEFGATLIPENIREESVKNIKHCYAVNNDVTDLEEKIFR
VFKCYYYI
NSDIFIFF

>AglaOBP7
MRTCAIVVCIATLVVSIHCASEEQHERVKKIHSECQADPKTHADDELLKKYHKGEEVDKSI
VGAHMLCMS
TKFGVIQEDGKINKSALKTSLSRLISDETKLNEAIEKCAVEKDDPKDTALALGKCFREQGG
LRGHEHIHN
RL

>AglaOBP6
MKVLALIVCVSVFCGLVIGTAIDRDLHKECQADKATHLDENIMKALDEGEITDRAKVGAH
LLCISTKAGV
INQDGSINKNMVKEKFSRYYSDAAELERITNKCSQQGTTPVETALKLAECINEYSLEQ

>AglaOBP5
MKSFLLVAFVVGVVTAASLPASEKKFINQVHANCQSNPKTFVDESLLKNLPANKDNAQVG
VHMLCMSKGA
GFQGPNGDINKETIKSKIALVIRDGSKVNELTNKCAVKTGSLEKTSINIFLCLNSNQVPYTP
DLN

>AglaOBP4
MKIFASFLCVFITCALVYGAPSIRDIHGECQSDPATRLDHDEFKAVRTGESFDRTKVGAHML
CMNKKFGT
QNADGTVNRNAVKEVLAQDITDETKLEEITNKCVEEGSTPSETALKLSKCVSENTKGGRH
GHGHEHHHGH
HHEHHHDH

>AmelOBP7
MKKFLVIFVYILSVAVIIRANGINEILKIMAVSMKDIRYCIIHMGLTFKDFIKMQELLQEEDIS
EGNIKK
YLTNYSCFITCALEKSHIIQNDEIQLDKLVEMANRKNISIDVKMLSECINANKSTDKCENGL
NFIICFSK
LLSDMYEDTFEDTLKHKSYV

>AmelOBP9
MFKNYHFFFILVITLIFLYFGEADIKKDCRKESKVSWAALKKMKAGDMEQDDQNLKCYL
KCFMTKHGILD
KNAEVDVQKALRHLPRSMQDSTKKLFNKCKSIQNEDPCEKAYQLVKCYVEFHPEVLQTV
PFL

>AmelOBP10
MKYSILLSLLITCLICSPSVHCGTRPSFVSDEMIATAASVVNACQTQTGVATVDIEAVRNGQ
WPETRQLK
CYMYCLWEQFGLVDDKRELSLNGMLTFFQRIPAYRAEVQKAISECKGIAKGDNCEYAYRF
NKCYAELSPR
TYYLF

>AmelOBP11
MKAAEIWLVSLYWYLILQIALVYGEISDIDEFREMTSKYRKKCIGETKTTIEDVEATEYGEF
PEDEKLKC
YFNCVLEKFNVMDKKNGKIRYNLLKKVIPEAFKEIGVEMIDSCSNVDSSDKCEKSFMFMK
CMYEVNPIAF
IAP

>AmelOBP12
MLYNNLTIVIILIMCGVQNLRARSVNIFQDIADCVDRSNMTFHELKKLRDSSEARIKLINEE
ENFRNYGC
FLACIWQQTGVMNGSELSTYNIAGIIEGQYHDDEDLKTFFHKIALTCEDDVHRKFLHVND
ECDVALSFKL
CMLKAMRNYP

>AmelOBP13
MKTIIFIFAFCLVGILAVSEESINKLRKIESVCAEENGIDLKKADDVKKGIFDKNDEKLACYV
DCMLKKV
GFVNADTTFNEEKFRERTTKLDSEQVNRLVNNCKDITESNSCKKSSKLLQCFIDNNLMKIF
E

>AmelOBP14
MKTIVLIFGFCVCVGALTIEELKTRLHTEQSVCKTETGIDQQKANDVIEGNIDVEDKKVQL
YCECILKNF
NILDKNNVFKPQGIKAVMELLIDENSVKQLVSDCSTISEENPHLKASKLVQCVSKYKTMKS
VDFL

>AmelOBP15
MKTILIISAICICVGALSIKDFQNAIRMGQSICMAKTGINKQIINDVNDGKINIEDENVQLYIE
CAMKKF
SFVDKDGNFNEHVSREIAKIFLNENEINQLITECSAISDTNVHLKITKIFQCITKFKTINDILN
S

>AmelOBP17
MKTIVIISAICVCVSAMTLDELKSGLHTVQSVCMKEIGTAQQIIDDINEGKINMDDENVLLF
IECTMKKF
NVVDENANFNEKISSDIVRAVLNDNEADQLLAECSPISDPNALIKISKILECFFKYKTINQIL
NS

>AmelOBP16
MKTFVIIFAICVCVGAMTHEELKTGIQTLQPICVGETGTSQKIIDEVYNGNVNVEDENVQS
YVECMMKKF
NVVDENGNFNEKNTRDIVQAVLDDNETDQLIVECSPISDANVHIKISKIFQCFMKYKTITDI
LNS

>AmelOBP18
MKTFVIISAICVCVGALTLEEFQIGLRAVVPICRIETSIDQQKEDDFRDGNIDVEDEKVQLFS
ECLIKKF
NGYDDGGNFNEVVIREIAEIFLDENGVNKLITECSAISDADLAVKSAKLLKCIGKYKTLKE
MLSG

>AmelOBP19
MKTIVVIFAFCICVNAMTIEELKIQLRDVQEICKAESGIDQQTVDDINEVNFDVEDEKPQRY
NECILKQF
NIVDESGNFKENIVQELTSIYLDENVIKKLVAECSVISDANIYIRFNKLVKCFGKYKTMKEV
LNL

>AmelOBP20
MKTIVVIFAFCICVNAMTIEELKIQLHDVQEICKTESGIDQQTVDDINEVNFDVEDEKPQRY
NECILKQF
NIVDESGNFKENIVQELTSIYLDENVIKKLVAECSVISDANIYIRFNKLVKCFGKYKTMKEV
LNL

>AmelOBP21
MKTIVIISAICVCVGALTLEELQIGLRAVIPVCRIDSGIDEKKEDDFRNGIIDVENEKVQLFSE
CLIKKF
NAYDDGGNFNEVVVREIAEIYLDENEVNKLITECSAISDADIHLKSSKLIKCFAKYKTLKEI
MNE

>AmelASP4
MKITIVSLLCVIYCALVHADTVAILCSQKAGFDLSDLKSMYEANSEEQMKKFGCFEACVF
QKLHFMDGNT
LNVEKLESGTRELTPDDFTEDVHEIIEQCVSKAADEDECMVARKYIDCALEKMKFLDDEL
EKIAGN

>AmelASP1
MASNTKQAFIYSLALLCLHAIFVNAAPDWVPPEVFDLVAEDKARCMSEHGTTQAQIDDV
DKGNLVNEPSI
TCYMYCLLEAFSLVDDEANVDEDIMLGLLPDQLQERAQSVMGKCLPTSGSDNCNKIYNL
AKCVQESAPDV
WFVI

>AmelASP6
MKGLGVSLLVALLLVLLAIEDTMSKKMTIEEAKKTIKNLRKVCSKKNDTPKELLDGQFRG
EFPQDERLMC
YMKCIMIATKAMKNDVILWDFFVKNARMILLEEYIPRVESVVETCKKEVTSTEGCEVAWQ
FGKCIYENDK
ELYLAP

>AmelASP5
MHVKSVLLLITIVTFVALKPVKSMSADQVEKLAKNMRKSCLQKIAITEELVDGMRRGEFP
DDHDLQCYTT
CIMKLLRTFKNGNFDFDMIVKQLEITMPPEEVVIGKEIVAVCRNEEYTGDDCQKTYQYVQ
CHYKQNPEKF
FFP

>AmelASP2
MNTLVTVTCLLAALTVVRGIDQDTVVAKYMEYLMPDIMPCADELHISEDIATNIQAAKNG
ADMSQLGCLK
ACVMKRIEMLKGTELYVEPVYKMIEVVHAGNADDIQLVKGIANECIENAKGETDECNIGN
KYTDCYIEKL
FS

>AmelOBP8
MTIEELKKTIKNLRKVCSKKNDTPKELLDGQFRGEFPQDERLMCYMKCIMIATKAMKND
VILWDFFVKNA
RMILLEEYIPRVESVVETCKKEVTSTEGCEVAWQFGKCIYENDKELYLAP

>BhorOBP3
MKIFLVLLCTIVGIWAQENKKLIAEEQMLEHIHDECQADPATNADHELLHNLAANIDNPQV
GAHMLCEST
KVGLQKPNGELDIPTIKEKIGLSVPDANRVEFLVKECAIKKNTPEKTAINLFMCLDKNGVT
YFHEF

>BhorOBP2
MDSLIFLVVVSSLLAMSTVQAALERSEYSPKLLELVDSLHSICIGKSGTDEDSINKVINGEFT
DEPKIKK
YMKCGITEVGVMNEEGVIDYEMTAELLPVKLVDKSIAIIKKCEADGKDIPNLDDRVFALFK
CYHDQDPET
FIFF

>BhorOBP4
MKTAFVFACVVVAALAASLSEEEKKLQEIHDKCQADPATYVDHELLHNLSANIDNPKVGA
HMLCESKAVG
LQKPNGELDLNVIKQKISLTVSDKAKVERLVKECAVKKQTPEKTAVNLFMCLDKDGVTYF
HEF

>BhorOBP1
MKTVFVVSLLFALAASDTDMEKKFHECDEETGLTLSEVTEYLLGDDAENDEKATKYMM
CMFKQQGAIDGE
GHLDMEKVRLSVNNYMKTTDAADDKEALECVEEKDTAEETALAVGKCVEKRRAELTSS
K

>BhorPBP2
MHGLVVPIFFYFCVALSMGLTPQEIEESINEIRSFCKESNGVSQDIIDAAYREGIYSDDMNFK
CYVKCIK
QKIGVIDDEGTADIEKIIELMPEKKKAMFEQKIRDCGTIYGSNICETAWLTMKCYGEGSTTF
PHGN

>BhorPBP1
MVSRLRVACVGLLLALPLVFGLSEEIQELVNMPHTTCVDETGASEEYIEKAKKGEFIDDDK
FKCYSMCVM
IQMACIDEDGIADVDATIAVIPEEYQKEAAPIIRKCDTQKGSTPCENAWLTHKCYYDQNPE
VYFLV

>BmorGOBP2
MFSFLILVFVASVADSVIGTAEVMSHVTAHFGKTLEECREESGLSVDILDEFKHFWSDDFD
VVHRELGCA
IICMSNKFSLMDDDVRMHHVNMDEYIKGFPNGQVLAEKMVKLIHNCEKQFDTETDDCTR
VVKVAACFKKD
SRKEGIAPEVAMIEAVIEKY

>BmorGOBP1
MWKLVVVLTVNLLQGALTDVYVMKDVTLGFGQALEQCREESQLTEEKMEEFFHFWNDD
FKFEHRELGCAI
QCMSRHFNLLTDSSRMHHENTDKFIKSFPNGEILSQKMIDMIHTCEKTFDSEPDHCWRILR
VAECFKDAC
NKSGLAPSMELILAEFIMESEADK

>BmorPBP3
MARYNIVVAVLVLGVVGARGSSEAMRHIATGFIRVLDECKQELGLTDHILTDMYHFWKLD
YSMMTRETGC
AIICMSKKLDLIDGDGKLHHGNAQAYALKHGAATEVAAKLVEVIHGCEKLHESIDDQCSR
VLEVAKCFRT
GVHELHWAPKLDVIVGEVMTEI

>BmorPBP2
VEMVCGSRDVMTNLSIQFAKPLEACKKEMGLTETVLKDFYNFWIEDYEFTDRNTGCAILC
MSKKLELMDG
DYNLHHGKAHEFARKHGADETMAKQLVDLIHGCSQSVATMPDECERTLKVAKCFIAEIHK
LKWAPDVELL
MAEVLNEVSWKS

>BmorPBP
MSIQGQIALALMVNMAVGSVDASQEVMKNLSLNFGKALDECKKEMTLTDAINEDFYNF
WKEGYEIKNRET

GCAIMCLSTKLNMLDPEGNLHHGNAMEFAKKHGADETMAQQLIDIVHGCEKSTPANDD
KCIWTLGVATCF
KAEIHKLNWAPSMDVAVGEILAEV

>BmorOBP5
MYTNFILIFYFGISIYDVRASSLDDLKMVYKNVIKECVGDYPITAADLKLIKARQIPNDDIK
CVFACAYK
KTGMMTEEGMLSVEGIKDMSQKYLSDNPEQLRKSKEFAEACSSVNDQQVSDGTKGCER
AALIFKCSTEKI
TNVMTAMRTPRRTVAADASSHPPGVGVVSTPSGRPHHREETPGFGFEL

>BmorOBP7
MANPVLLLTFVLMTXSMARLKSTEAPKSKTALFNDQDNMGYEELDMEEIMSACNESFRIE
YAYLESLNDS
GSFPDETDKTPKCYIRCVLEKTEILSENGVLNPATAALVFAGERNGKPMSDLEEMAVACAD
RHEKCKCEK
AYNFVKCLMYMEIDKYEKKN

>BmorOBP3
MFYPFRFTLLFYGLFVIYLVRAEPEKENHFTLALKKTLFSTARSCMSHVNANETDLEYLRK
DPPFPDKAA
CIIKCLLEKIGVVKNNKYSKMGFLTAVSPLVFTNKKKLDHYKSVSENCEKEINHDQTTECE
LGNEVVSCI
FKYAPELHFKT

>BmorGOBP56d
MIIILSYSQNVHLAETQKEKAKQYTSECVRESGVSTEAINAAKIGKYSKDKAFKNFVLCFF
KKSAIFNSD
GTLNMDVALAKLPPGVNKSEAQSVLKQCKNKTGQGAADKAFEIFRCYYKGTKTHILF

>BmorOBP1
MSRQQLKNSGKMLKKQCMGKNDVTEEEIGDIEKGKFIEQKNVMCYIACIYQMTQIIKNN
KISYEASIKQI
DLMYPPELKESAKASAGRCKDVSKKYKDICEASYWTAKCMYEDNPKDFIFA

>BmorOBP4
MTSAKTDVEIKAWFLGQAVECSKDHPVTTEELRMHKHELPDSKNAKCLMKCVFRKCNW
LDSKGMYDINAA
YASSTKDFSDDKTKQENANKLFDTCKSVNEENVGDGEEGCDRSLLLAKCLTKAAPQFGF
QL

>BmorOBP6
MSIKWRHIERVGSFCYLGSIVDDRGGTEADIAARINKARAAFSQLRPVWSSSTLTRRTKAL
TEEQKAEIT

KSSLPLIAECSKEFSVNQGDIDAAKKLGDPSGLNSCFVGCFMKKAGIINASGLFDVAATIEK
SKKYLTSE
EDLKAFEKLTETCAPENDKPVSDSDKGCERAKLLLDCFVANKGSNTRIVALSKDLDEEAE
DRAQWLAIGE
AYVQQ

>CbowOBP26
MKAFVVLTFCLAVANAKVDPKVIEEIIEEFTETVAKCSDEINPNADDIAALTEMKHIPDSHE
GKCMIYCI
YRSFDAVEEDGHVKFEGGMAFLSKIKESDPDMFDKMSAIYKKCTETDYFDKDPCISSANF
VSCNIKAGKE
ANISSDISSW

>CbowOBP25
MYYALLPRSAVLAVIIVVVAAYNFEDTEFNQILANDLEDVYSFTYSHPRSRRDDKAVEEDK
CHPPRRGRP
LCCAEETMRKLHDDKKEIKRACFKEITGKEKPERPDRHHGPPFDPFSCEKIEQHRRDMMC
IQQCVGEKLD
YLDADGKPKPEQFEKYVEGIFEKEDYLLPLKDKIVSVCLDEAKNATEKVSSDPCKSTGLVL
EHCIFINTQ
LNCPEDQIKDKKMCSKFQDRLRQGFDKRSSPSPEAEDE

>CbowOBP24
MNVHAGLQKPNGDIDKDDLRRALSEGIRDVITVDDIVDDCGQRVGSTAEEASVNLFRCIF
GHSNAYVHEW
KPSMLRQTSGAEGFFTSSLVLSVILATLSVRLI

>CbowOBP23
MKFLVLGFFAFGFIAGTKCLSEENMKEIEEFQKSCVAEVHTSPDVLSQIMSGDVSDDPKIK
AHLLCFAKK
AGVMTESGETIMDKLKQKLNQYLGSKADGFFEKCNIEITTPEDTAFNVYKCLSEMLQEGK

>CbowOBP22
MRVFIVFCCVIGHVLTQMVSPSQLNTILQYHTECREKTKLPNSLVTGLIAGQFPNDPVLKS
HLLCVHQKL
GVQDADGNLRKEFISETLGAVLPASVDSKELLNKCAVQKSSPEDTALDLDRCLYQTVQPM
RG

>CbowOBP21
MLPYLVLGLILYMFTAPVQGWMSDDKFGDKLEKMYRLWHDDCMKKTGAPENTVELIRS
GIFPDDDPRMKAY
NRCLYTDVMDKNARLLPEKLDYYIYPAFGKTGLKMYLDCEEKVKDEANYDDRVYKMQ
QCIYEANPDVSFN
FF

>CbowOBP20
MFSLLVLGFLCGVSALTEEQQQIMESLHAECISQTGATEDMIVNARNGDFSEDNKLKCYM
KCVFEELGVL
DDDGKVDIDGILAMLPDEYKDVATTVFNKCGTQAGTDVCDAIFQTHKCYYAANSEHYFL
P

>CbowOBP19
MKFYTFVLVACAFIATTKCLLATDREDKMKIHNVCVAETGVSQEFIDKMIAGEFCDDTNFK
NYLVCFLKN
DGVFLDNGELKADGANRQIREFADDEDTVSGFMANCAVQMATVEESAFHYSKCMYNTL
YG

>CbowOBP18
MRCSGGYLYTWCLVMMIVAVNAGKNQTNNCDIPASAPKRIEETINSCQEEIKLAILTEALE
ALNVNEHIH
SRAKRSAFSKDEKRIAGCLLQCVYRKMNAVNERGFPTAEGLISLYTEGIRHKDYVLATMQ
AVNHCLYHVQ
KNHLTTPQSIDEHGKTCDIAYDVFDCVSEEIGKYCGQTP

>CbowOBP17
MFSKGFVYVLACVALCQSIELPSELQEYVEDLHKICVTKSGISEDDHAAYDVKTNPHDPK
LQCYMKCLML
EAKWMNPQGDIQYDFIIDTSHPQIKDLLVAAINKCRAIDNGANLCEKASNFNFCMYDADP
VNWFLI

>CbowOBP16
MFKLILFCAVLLIPFVSAYLSEEDYGPKLSAVANKVHNACIKKHAVNEDTIMQVRKGNFVE
DELIKKYIS
CIWLLSTVLDESGNLNIKIINDLCPPKGKDTLPKIYHDCHAENAGVSQLDEKVYNIMKCW
YEKDPELFFV
L

>CbowOBP15
MKSIIVCFVAVAFLALAFAEETEKEKMKRIHEECQSDPATKVEESVLKAAEEGDVDVTKIG
PHTLCMNVK
VGLQKENGDIVKDELRAGLRRVPGVDESKIESIVEECGQREGGTAEEAAIKLFQCLQKRSK
ITHHHHHHE

>CbowOBP14
MSTTMKLAVFLFVVFSSVMEGYSAPSDSSNSTIKDYCIKEVEISEEKVNKFEKNPDDTPDE
DIMCYTHCI
LVTLGVVDDDGKIIIEKFTKIFDKYDMECVKKIPKILECTDLINLNKCAATDE

>CbowOBP13
MKFLVAFILTVAVCFVQAGVTEEQKKKIESYHKECSKQTGIDEDLVNKARNGQYTDTQILK
DYLFCTSKL
AGFINDNNELQKDVILQKTSVSTKDSAAAQKMFEACAVPQKNGPETSYHVLKCYYEKSG
LSLV

>CbowOBP12
MKSVVLLIISCCLSYSMALECGIAKANRNEIRQALSMCVKNNDTLEDILEMSSLSSSTTSSP
TEDSDDED
SQEDTIKSTSSTTKSPRRIKSSRIKRARSFSNTKQYASKATERNREESNNSNNTNNKIDNLK
DAQKDDSE
DDNEVSQETPKKRQDMSENCIVHCVLEHLNLTDETGLPDHSKLSEELLKTASGRELRNFL
QESTDECFQE
VNEENDLDSCSYTTKLITCLADKGKSNCADWPAGALPF

>CbowOBP11
MKTILKYVILIGVSSTYAYITEEGWGEPLIALANSLHNKCVPITGVTQASIDQVKEGNFIED
EKMKRYVL
CLWLVSEVISEKFELNTEIFKLLPKKLQDGHNIIGCTKKINGTDVSELYEKTYSVTKCIQKA
NPDEFIMF

>CbowOBP10
MTGLKNLKNVFSACFLLLLANVLLISAEKKLPIEAEECLKITNTDLKDMMAHPKEMSESH
YCFFKCIFEK
RGIINKVDGTVVPDVLDDIKDVAVLQVASEEKLAELKKCMADVEKIEKCTDMENFRVCFD
KLMS

>CbowOBP9
MKFLAYTVYLLLLFSVSIAVRNTAGKITQNESAKKTLGNCKTETGATMADIESLKEKKIPK
TKTGRCFME
CLFSKAKIMDNGRFNKKGMVVAFTPALKGDLTKMGKLRELSEVCEKEIGLNKLENCEGG
KKIVECVAKHG
KSYGMSFSTTK

>CbowOBP8
MNFVLEISCFFILILAVDISPANAVGKFPDGRPYPDGFEDCLKSSNAKLEEVLNKPKANISEE
VYCFFKC
LSERVGFIDQQGNVHIDKMDVTQIFQGAVEEVPDELKSCLGGVNKVESCQDMSKICECFL
KMAP

>CbowOBP7
MLRKFDVFVLGLLLTSRGVSGLSEEMQELADMLHATCVHETGARQDDIENARKGIFAEDE
KFKCYIKCIM
AQMACIDEDGIIDEDATIAVLPEEYRNQAEPVIKKCGTKKGSNPCENAWLTHKCYQNEAPE

DYFLV

>CbowOBP5
MEKVVFLLVLVLVAVVISKPLSEEGREKAQKINEECAKESGIEEDNLEKILADEFPEDDKM
KEHSFCFLT
TLGVMDKDGKIDKDVMTDTLKLFAPEGKEVEIMEKCAVETDDAKETAFAIGKCVHEQVK
SV

>CbowOBP4
MKHREEIGLECLRQVNIQRDTIENAKATLNFPEDRKYKDFLACSYKKQGFQSQDGVILYN
SIKDFLSRYY
KRNDLKVMDNCKENIREDHGEMALNALRCIMDNLKNMEEKSRR

>CbowOBP3
MRAAKGDYQDDMKLKKQILCFNKKVGLQDENGDIVLDVAKSKLFDIVKDEKKTMDILK
KCAVKKDTPENT
AFESAKCLHKLAPEEKLVI

>CbowOBP2
MCQLKFTVLILVSFMLLNVTGLNEKQMKAAVKMVRNVCQPKFKATDVDIDKMHKGDW
NIDHTAMCYMHCA
MNMYKLMNTDNSFNYQSALAQLNQLPDSYKKATEICMEQCKDSAVTLSDKCISAYELAK
CMYFCNPEKYF
LP

>CbowOBP1
MKYFLLLLCLSIMAQCAISELTEKQMKATKKLIRNTCQNKAKATTEELDAMVKGNFNQG
KNAQCYQLCIL
NTYKLLKSDNTFDWQAGVNALKANAPERIAGPGSASIKNCKDALKTKDDKCKGATEIAQ
CIYEDNPENYF
LP

>DmelOBP56a
MNSYFVIALSALFVTLAVGSSLNLSDEQKDLAKQHREQCAEEVKLTEEEKAKVNAKDFN
NPTENIKCFAN
CFFEKVGTLKDGELQESVVLEKLGALIGEEKTKAALEKCRTIKGENKCDTASKLYDCFESF
KPAPEAKA

>DmelOBP19b
MMQCSRMTTTLKMTNLLLAVACAAVLMGSATADEEEGSMTVDEVVELIEPFGDACTPKP
SRENIVEMVLN
KEDAKHETKCFRHCMLEQFELMPEDQLQYNEDKTVDMINMMFPDREDDGRRIVKTCNE
ELKAEQDKCEAA
HGIAMCMLREMRSSGFKIPEIKE

>DmelOBP83g
MQSQSLLLIVAAVATFLVAQTTAKFLLKDHADAEKAFEECREDYYVPDDIYEKYLNYEFPA
HRRTSCFVK
CFLEKLELFSEKKGFDERAMIAQFTSKSSKDLSTVQHGLEKCIDHNEAESDVCTWANRVFS
CWLPINRHV
VRKVFA

>DmelOBP93a
MYVYNLLFVVIVFSYCAKSFNYTSCDHAKQPKFLSSCCDVQKNDKAINSCRKSLLGNNST
NSNGEVRNLK
SDKVALHACIAECSFRTNGFLLSNGTVNTQALQKSYQQRYKNDPNMSQLMLKSLNSCTD
YARKRVQEFQW
MPKKGDCDFYPATLLACVMEKVYINCPTSKWKNTSDCTAMWKYLVACDDVASNKKK

>DmelOBP99d
MNHLRLEIICWSCLLIAMAVSTEAASVWKLPTAQMVYEDLEKCRQESQEEDAATLRCLVK
KLGLWTDESG
YNARRIAKIFAGHNQMEELMLVVEHCNRMEQDTSHLDDWAFLAYRCATSGQFGHWVKD
FMSQKEVER

>DmelOBP46a
MCSQLFAFLLLLLTAFVTGRSTPPALDEDCELNSVDTMHDFCCDLHDESPQFSDCQMEWH
EKIPYETDEE
EQTYMFCTAECSFNSTNFLGRDRRSLNLNEVKEHLESDLVNDADIKLLYDTYVKCDKHAL
SLMPHKGVKQ
LSKRLSRLGCHPYPGLVLECVANEMILHCPTKRFRQTAQCEETRNHLKQCMQYLKYKS

>DmelOBP47b
MSPSQLLVIFASLALNTRLVFGQATIDCQRPPQLVDPALCCKDGGRDQVAEQCAQRILGTA
NGQKAGGPP
SLDTAACLAECILTSSKYIDEPQKLNLANIRSDLSAKFSNDTLYVETMTMAFSKCEPQSQR
RLAMIMQQQ
QQVQQQKTQQQQPRCSPFSAIVLGCTYMEYFKNCPDHRWTPNAQCTLAKAYVTQCGLG
A

>DmelOBP49a
MLSKSQLLLLVVGFCLNAAVSADVDCSKRPSFVNPKTCCPMPDFVTAELKQKCIKFDMTP
PPPPDGEASG
SFESKRRHHHPHPPPCFFSCIFNETGIYQNRKLDEAKLNAYLQEVFEDSSDLQTTATQAFTT
CATKVADF
EANLPPRPAPSPPPGFPMCPHDAGHLMGCVFRNMMKNCPDSIRNDSQQCTDMKEFFTKC
KPPRGPPPSAE
DM

>DmelOBP50b
MSSVLHLLGFLWLPLLVYSVSNDMGGLQKCTELLNTHKLVYCCGKSFLDKFPFVGSNCTP
FWDDYGPCRY
ECLYRHWDLLDQDNKIKKPELYLMITSLYSPLNGYDKYGAAFKAAHETCEALGSRHADF
LLLYSNQVADK
MGMASSTCLPYAMLHAQCTMVYLTANCPRENWIDDPKCNSLQKLLSSCTKKLDEKTNAL
KGKDEELTDNG
CGHIDSEGSNLLMACFLTLMIAKFISDH

>DmelOBP51a
MKVFIGLVLLLAVTTLSSALFESEANECAKKLGITPDYFENFPHSSRVKCFYHCQMEKLEII
ANGVVTPF
DLKVLNISPESYDKYGVKVKPCLKLSHRDKCELGYLVFQCLKREFNL

>DmelOBP56f
MKVFLLFIFISAIWLQAFCMKSSEKIKACLKRQLGYTITENTKFDAKEDSLQSKCFYHCLL
EVKGVIAND
AISSEQPRKVLEKKYGITDTDELEKAEEKCHSIKASGKCELGYEILKCYQSITKH

>DmelOBP56g
MRATFALTLLLGCLSGILAQANIDSSVSKELVTDCLKENGVTPQDLADLQSGKVKAEDAK
DNVKCSSQCI
LVKSGFMDSTGKLLTDKIKSYYANSNFKDVIEKDLDRCSAVKGANACDTAFKILSCFQAA
N

>DmelOBP47a
MNRVLVLLLVLKMFALSEININLGLTVADESPKTITEEMIRLCGDQTDISLRELNKLQREDF
SDPSESVQ
CFTHCLYEQMGLMHDGVFVERDLFGLLSDVSNTDYWPERQCHAIRGNNKCETAYRIHQC
QQQLKQQQQNL
LATKEVEVTTTPAGSDETKP

>DmelOBP50a
MRTGRILVALIFLGLIIPFRAAKCRAAPKSVQNVHVCCSAPLPNWGVFNRECHKSAIQASV
SINRISKSK
VNLANFLIKCRLDCDFNASSVLQGNRLIQAKVRPMLERAFSNEPTIDAYESNFAKCSTVVR
SKYQELSPL
SRQSDACDRHALFYSLCAYARLIFTCPDKMWQRNNRMCQEAKAYAKKCPWPALKMFMR
NT

>DmelOBP18a
MKVVCSIAVLWICLITMWQSAGRVNAEGCLKHHNLTSAQVQAVAPSTPVADVPVAVKCYS
RCLIQDYFGD

DGKIDLQKVGKRGSQEDHVILSQCKQQFDGVTNLDTCDYPYLILQCYFKGKQSGTIAS

>DmelOBP8a
MMRRSQIGLLSRLLLLLLVVELTPPAIPVPMRSSPQSLALLRARDQCGRELTAAQRLQLDR
MQFEDAAHV
RHYLHCFWSRLQLWLDETGFQAQRIVQSFGGERRLNVEQALPAINGCNAKTSSRGSGAQ
TVVDWCFRAFV
CVLATPVGEWYKRHMSDVINGNA

>DmelOBP69a
MVARHFSFFLALLILYDLIPSNQGVEINPTIIKQVRKLRMRCLNQTGASVDVIDKSVKNRIL
PTDPEIKC
FLYCMFDMFGLIDSQNIMHLEALLEVLPEEIHKTINGLVSSCGTQKGKDGCDTAYETVKCY
IAVNGKFIW
EEIIVLLG

>DmelOBP57a
MFNTRLAIFLLLIVVSLSQAKESQPFDFFEGTYDDFIDCLRINNITIEEYEKFDDTDNLDNVL
KENVELK
HKCNIKCQLEREPTKWLNARGEVDLKSMKATSETAVSISKCMEKAPQETCAYVYKLVICA
FKSGHSVIKF
DSYEQIQEETAGLIAEQQADLFDYDTIDL

>DmelOBP19a
MKFHLLLVCVAISLGPIPQSEAGVTEEQMWSAGKLMRDVCLPKYPKVSVEVADNIRNGDI
PNSKDTNCYI
NCILEMMQAIKKGKFQLESTLKQMDIMLPDSYKDEYRKGINLCKDSTVGLKNAPNCDPA
HALLSCLKNNI
KVFVFP

>DmelOBP28a
MQSTPIILVAIVLLGAALVRAFDEKEALAKLMESAESCMPEVGATDADLQEMVKKQPAST
YAGKCLRACV
MKNIGILDANGKLDTEAGHEKAKQYTGNDPAKLKIALEIGDTCAAITVPDDHCEAAEAYG
TCFRGEAKKH
GLL

>DponOBP3
MHCSRACLIVFFSICGLSSSLKITLPPELQEYVDDLHKLCLEKGGLTENDHQTYDINHKNE
KMMCYMKCL
MLESKWMKSGGEIDYDFIETQAYPEVKDLLLNALNKCRTIEEGADLCEKSYNFNKCLYDA
DPVNWFFV

>DponOBP21

MALTTWVLSIMLILPAIRALSDEMKELAQMLHNTCVAETGVNEDFIQKVNAEKIFADDEN
LKCYIKCLMA
QMACIDDDGIIDEEATIAVLPEEYQALAAPVIRACGTKHGANPCENAWLSHRCYAEMEPSA
YMLI

>DponOBP20
MKVFVVLCIVLFAFTLIVSAKKNKSNDEEKAKSYKKVFKECQKKDETRVDASIIRKLKKH
KQVDLPANFG
EHKLCVFTGIGLLKADNTVDEDKLKKKIASAKPQKDIVDNIVMDCTSSKSTLQETALNLD
KCLTTYSIEF

>DponOBP19
MKAMFVTLTVATVVVFASADLTEEQKQKIVANGKACVAETGADPELIKAARQGKFADDA
KLKAFALCMSK
KSGFQNEAGEIQSDVVKQKLGLAIGDEAAAKKLVEKCLVSKGSGEETAIETFKCYYENTP
THIAVF

>DponOBP18
MNGFSVFFLLLLAAVVKSDFDFSNYKEFENLAGDQREKAIKLFKECMAETGATHEMMEK
SVEGDIPDDIV
FKNHLVCIGKKSGFIDENGMHIKEKLKEKLTLLLGNEELVDKILDKCFMEKGSPQDTAFEL
AKCCHREYH
N

>DponOBP17
MQVTMNQGWFLLLVSVVSVFAELDQTSLPPEAKELMAALHKNCIEQVGVSEADVDKLR
AANFEEDANLKC
YTRCLMAESGVMDENGAIDIEAFGEILPEAIRGNIQAIFRSCSLTKNDIVDQCVKAYEMVK
CWHKENPES
YFMI

>DponOBP16
MKLMWILVLGAALKSTEGAMTEAQMKAALKLIRNVCQPKNKATDAQIAAMHNGDWNQ
DKNGMCYMNCVLN
YYKLQLPDNSFDWETGLKVVESQAPPSMAGFIMETIKSCKDAVKTGDDKCKAALEITKCL
YDQNPEKYFL
P

>DponOBP15
MGTTIFLLVGLFMMTNAYVPNVNDKIRDFCIDDSGVSIEMVENLLANPEKELIDVESCYVH
CIFTEMGLL
SENGNVEIENFKSLKASEAPYIDLNCLEEIKSIDHCNEMMILRACHV

>DponOBP13

MSNLLKLSIAFAVVSVISCQDFTEEQRKKIIENRQQCIEETKVNPDLIEKADLGDFAEDQAL
KCFTKCFY
QKAGFVNDKGEVQKDVVEAKLPPQADKKRALEIVDKCALKGKDACETVYLIHKCYFEH
THPEADEKTAKD
GKSEEKKA

>DponOBP12
MHFQWLTNVSVFLCILGVAQLVAAGKPNDLFTRITPGDVEVCGKDTGVDRKDFEEAREK
GALNHSMLCFL
KCAMEKAGFLKDGHLEIDQAKEASPDKMTEPVVECFKAVGPISTCDDIQKVENCLPGS

>DponOBP10
MQLLFVAVLVIALVQVNSLTDKQKELLTQHYNQCVAISKVDQAVLQKARAGDFANDPNLK
THIKCISEKI
GFQGTDGKFRRDVIEKKLKETLPGDNAKNAKLIETCVVANKDPQLQAFNAFKCLYTNAKI
NLL

>DponOBP6
MKSIVALFVCALTATALADAEINETAFKAGRDRIMAMSRTCDENPATAVDQKALKKYLQS
NGPAPANGAA
HALCITKNLGWQNEDGSVNKPVITEKVKAIFGSVDAKVQQYIEECTEAKATPEDTAEQLL
KCYRKHSPKI
E

>DponOBP5
MSEKTHFALVALLLTCLVNIIDADQREKAVEFQRDCMEAHGLLEDELHEIMDGKPIQNEAF
YFHFFCVVK
KAKLISDNGIVNTDHFEENLKGVIDEENMAHVAALTRRCLIQRDDIFTTIKMAIDCFYSSEH
KL

>DponOBP4
MNTALKVFLVALAIPTIMGLSDEMQELANQLHTTCIGETGAAEDAITNARNGDFSEADSFK
CYIKCLLSQ
MAIIDDNDGTIDVDAMVAVLPEEIQEATEPIIRKCGSIIGANPCDSAWLTHKCYYKEGPEHY
FLI

>DponOBP2
MKQLVMVVLTALCVVHCKGLECGLSKISSEHFRKIASECVKDNETLNRIWELTSETSMEE
DSVSSDEEVP
VTKGREAPNFHDLGSSAHRNMKMSGASRTKRSRKGFNNESPMSNVQKKSSPASTTTEHT
TTMQSEENEEN
AAANNVEESGEVCILQCIFEKLEMTDTNGLPDHKKVASALVKSASGRETQDFLQDSVDEC
FQETEEGDFE
NSCEYSTKLVTCLAGKGKSNCADWPVGDLPF

>DponOBP1
MLTKTILIWAAILLTVFIPKGNCRLTEKQLAAAIKLVRNMCMGKSKANPEDIEKMHQGNW
DVDYQAQCYM
WCGFNMYKMLDKENHFDKKAALQQMDQLPIDLQEYVVKCMDQCENAVTNFDDKCVV
AFEYSKCLYFCDPE
KYFLP

>ItypOBP10
MNAFICMFLVFGVVKAYDFSDSIFNDHLNQIYYTLDNWQHERIRRNAEDVELKCRKPPPP
MPKPCCAQDS
FRDLMDKEREVLRDCFKEVVGEEHHPGRSNHPNKFDMFSCEAVEKRKNDIICIKQCLGSK
LGLVNKDGKL
DQAQIGNYVKSTFKNEAWLSPLADQIIGKCLVEAESVAPPKFHIEKLKPCKPSVITFKHCLD
REIQLNCP
ADQIHNQESCERFRNHLNHKNDFDEDQPMMGPPDDD

>ItypOBP9
MTKLQIVLLLTTLMGSFNIITGEKKCNSSNCMYDRMLETVGKEFIEQCFKETGVTPEDIRS
VMEQNGYGE
KQIVFPKMLDKENWYFGKRWSNQYRLY

>ItypOBP8
MKYLFLVIVSLLLAVQGEVSKEELEKLKEIHDTCLTESGVDQSMPEKAFKGEFTDDPKFKE
HLLCFHKK

>ItypOBP7
MKVLFAFVCLVVLIQVNSQTDKQKELLAQHYKECLAKSKVNEATLQKARIGQFADDDKL
KEHILCVAQKI
GFQNSAGQFQNQVIETKLREALKGDAAKTKKLISDCAITNPDPKLQAFNAFKCVYQKASI
NLL

>ItypOBP6
MVLYLCDLVQLAPTNQTEEVTTSTKRQLTREKKKKIGKTCMLETGVRIETILRAIKEDIPK
NDEKYKSYL
VCSYKKQGYLSEDGGTMLYDNLYSFLQESAGYAKEDLHYIDDCKTITAETPGDLCLKKLV
GILDGLHKVE
KNREIDTNTIES

>ItypOBP5
MRQPGGNNKNTQQDYEMWTPSTGYQPSGSNNDFNVRTRYDGNTRFNRPSSEECRDQGN
GNIPRSPFGSSN
LPRRQRSSYFNREDDDNDNDCISQCVLGYMQLLDTDRSPSETLIIKWLQEHVTRNEMDRI
KALRDTRKCF

GKLVTTDIEDGCEYAKELSKCLELDLE

>ItypOBP4
MISAVIFFALVGTIFCADADLTQQQKDKLLADGKACVAETGVSTDLIQAARQGKFTEDDKL
KAFSFCMSK
RLGFQNDAGDIQTEVVKQKLGGALGDLGVAAQLVTKCLVPKATPQETAFESFRCYYQNTP
THLTVF

>ItypOBP3
MATGKVFVYFFVVLFLSEQSVSRMTEKQLAAAVKLVRNMCLSKEKAKLEEVDKMHEGN
WDIDHKTQCYMW
CVLSQYKLIGKPNHFDRESANIQVDTLLPESMHDYVVGCLDKCENAATNFDDKCVAAYE
YAKCLYFCNPK
EYFLP

>ItypOBP2
MNSVAVFAVLALGAVCVIDAYNFQDEDFXSAVVVRDGRIVDSIDSGPVHPRVRRDQEAATV
AEEKCPKRH
RRPKLCCAEETLDALHAKKKEITKACFKEVTGLEKQDRHDHGPHFKRFDLFNCKEVEKR
KSDMICIDQCV
GQKKGLLDDSGAPIRDQLIQHLKQHFSNESWFDQTVVEKITSNCLAAAKNATETPIKFSTE
GLKACNPSG
ITLKHCLFREIQLSCPADQIKDKTACDRFQDRIQKEIEIDDLRLAPDDQQ

>ItypOBP1
HGPPVELLSCVAKELSLIDADGNVSEAGIKQHVEHVESDAGKAEEIVKACAVNQASADET
VRQLWKCLHD
NNIVQTPKHNHGSNESSSSESEEHSHRHRH

>ItypOBP15
MIQHHRTNSDLTAVKMKILAVLFVICVLFQFTIARNGGNLHYSKISMKKVQKRCQKNEESR
IDPDVLKKL
RKGEEVVQLPDNFPDHVTCLMKGMEYLNDDNTVNEEKVRNMVQRRVTDDQDVDAIVG
ECKAVKTALKETA
LNLINCLRKHELLWNHNFHD

>ItypOBP14
MYGSVLKVSLVFAVISVISCQDFTEEQRKKIIQNRQDCIQETKVNPELIEKADQGEFIDDQA
LKCFTKCF
YLKAGFVNDEGEVQKDVVEAKLPPQADKKKALEIVDKCAVKGKDACETVYLIHKCYFE
HTHPDLPAKAEE
EKKA

>ItypOBP13

KVEMLTDKNEIIKCAMECMQAEIDRLTEERNEHLKKLFESHNAQISETKKKQWCYNCEQ
DAIYHCCWNTA
YCSQTCQQQHWQAEHKKVCRRKRQT

>ItypOBP12
MLTVGKLVLVLVLVLIETSALQKTNNKCEIPTAAPKKIEDVINTCQDEIKIAILSEALEALNI
NEHKVSR
KRRSTFNDDEKKIAGCLLQCVYRKMNAVNQYGFPTVDGLVSLYTEGITQKEYVLATLQSV
TKCLGKAQKT
YDIPAQNGTASTACDVAYGVFDCVSEEVAKYCGQTP

>ItypOBP11
STGDSTMKFILLVIVVGQMGCVFGAMTESQMKAAFKLIRNVCQPKNKATDAQIEAMHKG
DWNQNKNGMCY
MNCVLNYYKLQLPDNSFDW

>MaltOBP6
MKTVFVFLCVIVGILAQDKKLVAEELMLEHIHDECQANPATYANHDLLHNLAANIDNPQV
GAHMLCESTK
VGLQKESGELDIETIKSKIGLSVTDPERVEFLVKECAIKKNTPEKTAINLFMCLDKNGVTYF
HEF

>MaltOBP2
MKAVLVLCVVVAGALAQLPDSERIHLKQVHDSCQADRATYADESRLKQLNKYIDDAVVG
NHMLCMSKKAG
LQKGNGDLDIGVIKQKIALVTADKSKVDGLVKKCAVVNGNPQKTANLLWLCFVQNNIDY
LHRL

>MaltOBP1
MSSSIFLVVVFFLLAVSKVEPTMKRSEFPPKVLELADALHSTCLPRSGTDEESINKVIDGEF
TDEPKIKA
YMQCLMDESELVDENGELIMDLIIPLTPPKIFDEALKNTKFCDGERKEVKERTDKAFVFFK
CIYGKNPDT
FIFF

>MaltOBP5
MNKLTVLFYITFFAAVHGLTEEEKMAIHEDCFSQSGVSEEMASKVMDGVFVDDPKLKLYI
LCFAKKVGIM
NDSGDIQVDVFRAKLGTKVPDEVKLNEIIAKCAIKKGTPEETIFALSRCTHGNKS

>MaltOBP4
MKFSVFLLCLCLVVPYVYSALSEKQMNATKKLMRNTCQNKAKPTSEQIDAMHKGDFSG
DRNAQCYLLCLM
NTYKLLTKENTFDWEGGIKALEANAPASVAGPGTISLKNCKDAVKTQSDRCVASMEIAKCI

YDDNPSNYF
FP

>MaltOBP3
MRATTEIFLIVGVCAALVSGMSDEMKDLLDSLHAQCLSDTGANEDLISKAQKGEFTEDEK
LKCYMKCIFD
ETGLFGDDGQIDIDGMIAMLPDEIREPFSPTVRKCGGLLSGSPCQQSFMLYKCCFDEAPQY
YFLP

>TcasOBP11
MSFLILLICVIPAIFCRSFSHDELDTDLSFIKTCNRTSPISMRTMNEVLINKKLGHGESSAFKC
FLHCLF
MKYGWMDSDGGFLLHDIKQTLEESDVEIASLEFILYKCTATESNNRCERAFVFTQCFWDK
MAEQQPSEDQ
FFYNIEDKK

>TcasOBP24
MSRMLPAALFVVLATLTFATAEIVVPDDLKDYINELHDHCLKEMGLTEGDHKNYNIHVKD
PKMMCYMKCL
MTTSKWMNMDESIQYDFILSSVHPAVKNILLPALDKCRDIPKGTMECEKAYNFNMCLFNA
DPENWFFI

>TcasOBP16
MQLLVVVLAVCVLGANAGLDPKFLEKLTQEVQAVGTSCGEKEHATADDMIEIMEEKFPPT
SHEAKCVVAC
FYKHYKMMKEDGTFDKDAAVKAFDEIKAQDAEIHAKILKVIDACDAKKQMSDDHCVSA
ASMAGCVKTEAI
ANGLTKEAFMAS

>TcasOBP26
MMHLKNFVVLVVCPLFVFAKVEIPPDLEAEIDEYFEQCFEPNGVTMDDIKAYKMGDKDP
KIMCFMRCLFV
SGKWMDENENMQYDYIKETIHHAIRHITIPELENCGKEAQTGDKCEKSFNFFMCMNRAEP
EDWILDYKS

>TcasOBP25
MPLKNLIILIVCPLFVFAKVDIPPDLQAEIDGYYDICYKQIGLTKDDLKAYKIGDRDPKIMCF
MKCVFVE
AKWMDENENLQYDYIKNTIHHSIRHITLPELENCGKKAEGDKCEKSFSFFNCMNKAEPED
WVLIQ

>TcasOBP8
MIRYYIVLLLYFFAPPVLGISEEMQELVNQLHSTCVAETGVSEDLINKVNSDKVMIDDEKL
KCYIKCLLT

ETGCISDDGVVDVEATIALLPEDMKAKTTPVIRSCGAKMGANPCESAWLTHKCYLETSPA
DYVLI

>TcasOBP20
MATRFCFGLLILFVGTVLVFAENEHEILEVRALCMNETGVSEETARNYKPAEDPASEEILC
MVKCIFEKI
GCLKDDGSFCVDTMKKKNYIMDVINEENEEKIYECLRGVGKITNCRDMAAVEECFVKND
SK

>TcasOBP2
MNPITSVILTFLFVFSFGEKESEEALQQIFTELDGPAAELRDQCLEKNSMKVTDLKTYNTSN
DIPEKELC
FYKCFYEGVEFIDANGNLNVNNMKEIPAISELGDEVLNEITACVEKIGKIRCCGDLRKIEQC
YQNITM

>TcasOBP21
MLRLVSLLCLFLLVQGENLDMFDPAGLQACMKKLSVGETELAKALEDKSKDPPEKIMCLF
KCALEDSGFL
QDGVVDKSKWPMPECVQDVVKITNCNDMVALKHCFD

>TcasOBP22
MKPIFAIITLTLCTTVHALDCGIHINKNDALKATINKCLISNKTLEDLWDMAPMSSESDSSSE
EVPPVDG
KMLQNFRIKRASVRLTNTETNETTPEPKAVSSEAQATENCIIQCIFDNLQMTDSTGYPVHT
KILDGLLKN
TTNRELRDFLQDTTDECFQVMDKEDTMDPCSYSNKLVTCLAEKGRSNCADWPVGELPFK
P

>TcasOBP15
MNCFVIFALSLSATVFGQSLSEDEMRENARKLMTSCKDKVGASDADVEALKMHQMPESR
EGFCMLECVFD
SAKIMQDGKFSKSGMIEGFKPLIGDDKAKLESLEKLSATCESELGDGEDKCETAKRLVECV
IKNGKTHGF
EVPPPRE

>TcasOBP23
MKYFPHLCLCLIFFELSEAAMSEAQLKAAVKLVRNMCQPKSKATNEDIEKMHHGDWNID
RTAMCYMHCAL
NSNKLITKENVFNRDYAITLAEKNLPTALKTASIEAANLCKDSAKTLDDKCVAAYEISKCL
YESNPEKYF
LP

>TcasOBP9
MKAILLLLVATLSFYHVYCAMSEAQMKAALKLVRNVCQPKTKATNEQIEAMHTGNWDL

DKNGKCYMWCIL
NMYKLIGKDNSFDWEAGIATLKAQAPESVRDPAIASVNNCKDAVKTTSDKCEAAYEIAHC
MYLDNPEKYF
LP

>TcasOBP12
MKLLITLATLVVATYAIDKEFVQELRQKLRSHVEACAKEVNAGPDDVSAIFAHKLPATHEG
KCIFFCMHK
LYNAQNEDGSLNMAGALANLELIKDMDPDVYTKVSTSFKNCESAPFDSDPCLYAANLVT
CIVKEGRAVGL
DEVLVE

>TcasOBP13
MKFLLVFLSVAILCTFAMDESFLQQTRDRVKAIVKECVTEEKATDSDFDDIMALKIPTSHE
GKCVFFCSH
KKFNMQHPDGSINKEGALDTFEVVKDVDAEFHDKVITVYNHCLSTPVDPDPCVYSVNLF
QCFMKEAKAAG
IHELIIK

>TcasOBP18
MKLFILAGILFTGVCAVDQEFVEKFLQKMEKIGEECAEETHATSDDIADLIEQRDPKTHEG
KCLIFCYHK
KFNTMKEDGSLDKVGSVLALEEVRDADFELYKNILTIFVTCGDKAKIYDDPCETATALTM
CGRDEAKALG
LQDAIFG

>TcasOBP17
MKSTWFFLLLACSLTCALDQEFVDEFLEKMQEFGAQCAEETDATSDDIAELIARKLPPSTH
EGKCMIFCM
QKKFNMMKENGGIDRAGAIAALKPLQKADPELHQKVLKIFVTCGMRVKPSPDPCDTATE
LALCGKKEAEA
IGLEDALLT

>TcasOBP14
MNSVLFLLVCALVACSGELDKEFLMQFLQKIKKVSEDCIAETQATKNDIKTLLEHKIPDSH
EGKCMIFCF
HKHFQIQNEDGSLNKVAAISLLEPIKDHSQDIYDKVVKIFNTCFDSAERDDDSCIYASNLAE
CAIRESKS
LGLDDLLVIE

>TcasOBP19
MKYFVVFASLFLATNALSQDFIDKFVAKVKSIGETCVPETNASKDDISSLLAHKMPDSHEG
KCLIFCFHK
QFQIQNDDGSINREGAIKALEPLKADDAELYEKVISIFKKCESTPVDGDSCLYAASLAECAV

KEGRAMGL
DNLIVLEIE

>TcasOBP7
MYKTRVIYVLFALCLVEIFAIEMDDDMKELINNLHNTCTGETGATDDQIENARKGNFAED
DSFKCYFKCV
FDQMGCMTDDGKVDSEAVIAVMPPELADKIASTVRGCTEVGANPCETAWLANKCYQKS
NPDMYFVP

>TcasOBP6
MSPLLLIFISCLFPRVFGISEEMQELANTLHATCVDETGVSEDAIESARKGNFAPDDKLKCY
MKCIMEQM
ACIDDEGIIDVEATIAVLPEEYQAKAEPIVRKCGTKIGANACDNAFLTNKCWYEEDPEDYF
LV

>TcasOBP3
MWSFVTLLFSFLVLASAQKKGKYWTTISECLTEHSMGVEDMKKFDLPAEKMSEEMLCFN
KCFYDKLLITD
ENGEINTDNLMSIPLVNAIDASKHDDLVTCLKKVGKIEECDGVKKIEQCFVEFI

>TcasOBP5
MAKKQLVLFFLAFIFLQSSWAYFFMSQKFAEVREECLSENSMTMDELHEGWKMENLPES
HLCFLKCLLEK
REVIDENGVPQKEKIDEILTVKQLSDEKREEISTCITNVEKIENCETMSEIMRCFPKKRRD

>TcasOBP1
MILKASIFLILAVATFGAILEDSELMKVVENCVKKTNANESEFSSPNFLETTPSQPALCTAKC
LLESLEI
VNSEGNINMETLKEYAQPFESPAREAVATCGEEIKSVTTCDDMEKYRKCVEPLIKNS

>TcasOBP4
MCRLFVVLSLFVASQALDVEKIRNELMADKNFVELRNKCLDKLGLKEEDLRDLKFDGDV
SEDLMCFGKCI
QEEDGLLDSEGNLNEEKLEKKIETMPFLSRVSDDTKNNIMECLKEIGKIETCQDFGKQRDC
IHKYV

>TcasOBP10
MKTVAVLLFLALAACTKQEDDDRQETIRQYRDDCIAETKVDPALIDRADNGDFTDDAKL
QCFSKCFYQKA
GFVSETGDLLFDVIKDKIPKEANREKALAIIDKCKELKGADSCETVYLVHKCYFLHSYGTD
KKTE

>XquaOBP19
MKTVVLCLCVLALGATKPLSDEAKEMLKAIHEECAADSGVEKEDIMKVLEEDGDDQKIK

DHVFCFQDKIG
IITSEGTIDRALLKGKLSDFLEDEDKAEEIVNKCADEAEGKESPVERAFTLGKCIHREKPTE

>XquaOBP13
MKSFLLVAFIVGAVTAGTLPASEQQFVNQVHAHCQSNPKTFVDENLLKNLSANKDNAQV
GVHMLCMSKGA
GLQGQNGEINKGAVQSKVALVIRDSSKVNEIVNKCAVKTGSWESTAVSMWLCLNKNQVP
YTPILN

>XquaOBP24
MKVVATVAVIVVFSVATSGQNYSDEERKRILKNREDCVAETKVDPELIDRADKGDFVDDD
KLKCFTKCFY
QKAGFVTETGDLLIETIKAKIPSNIDKEKALEIIEKCKQKGKDACETVYLVHKCYFEHTHIP
EPEKQPEI
KSEPAAPEGAATNVVNEKENVKEDKNANKTENQGEAKKL

>XquaOBP23
MVSLTTLIGATCLLLGMQVGADPPMESQIVRDCIKSSGVPAEEIGKGPDSGASLEKMCFLK
CLAEGMGML
SEEGDLNFDNIDNVPFRDDVPEDEKKEFIECASKVGKIEKCEDMAKVMACRQ

>XquaOBP22
MYFFVSLLMCSVFILTQGVPVRDEYVDDMVQRKAMALDCINQVQIDIKIIQAAINDYANL
PNDPKYKQYL
ACSFKKQKFQTEDGKLSYKEIYKFLNRFYDLKDLSVLETCKDVTGVDDGDRAYNALKCIF
P

>XquaOBP21
MLFGSIVVFGCLVLVYSIQLPEELQEYADDLHNICIQKSGITEADYAAYDIKNNPHDVKIQC
YMSCLMLE
SKWMTPIGAIQYDYILENAHPEIKDLLEAAINKCRKVEDDSDLCKKASNFNSCMYEADPE
NWFLV

>XquaOBP20
MRVFLGVLSLLGIIITSQCMTQEEIEYFTGLHRECGITDDVTDRAMAGNFPEDQQFKERLF
CVSKKVGMQ
DEDGLLLVKEVEDQMRRNVPDEEKVNQILSTCMVQLDTPEETAYRAMKCIHKIKTS

>XquaOBP18
MKSIVIVFVSFAIISTQSMTENQREILMAAHAECSAETGVDEAKVRMALTGEAAVDQSVK
DHMFCINKKI
GVQNDAGELQVDAIRAEVETAVADKSIIDGMMSTCVVQKSTPQDTAFETNKCFYNMEHK
P

>XquaOBP17
MNFLGLTVLCIFGAVQAGIITDEQKAKIIEFGKQCLEETHADEALVRKAANGEYVDDPSLK
KHLLCFTKK
AGFQNEAGDIQIETVRMKLNAITKNEKMTDDLITKCAVKKDTPEDTAFETLKCFHELSPEK
QFLTA

>XquaOBP16
MKGLVFIVCVTISVTLASGLTEEQKEKVKIVHKECQDDPATHADENKLSDSYKGVEVTGL
GAHFLCMSKK
LGMQKDNGNVDREAIKRKLSDVITVPEKLQEAVDKCAVQKATPEDTAEHVLKCFHDYAG
HVLHAHDHHH

>XquaOBP15
MKPVLVFLCAIVVGITGQNRRIVEEQNLHHIHDACQADPATNADHELLHNLAANIDNPQV
GAHMLCESKG
VGLQKPNGELDPETIRSKLTVFIDDPAKVDKLIKECAIQKNTPEKTAIYLFMCMDKEGVTY
FHEF

>XquaOBP14
MKVAFVFATAIVGILAADNILIVEEQKLHHIHDACQANPATYADHDLLHDLSANLDNPQVG
AHMLCESQG
VGLQGPDGRLDLPVIKSKIALSVKDPAKVDRLVRECAIAKTTPEKTAINLFMCLDKNGVTY
FHEF

>XquaOBP12
MKSVIVFLCAIVGILAQNRLIVEEQMLHHIHDACQANPATYADHDLLHDLSANLDNPQVG
AHMLCESKGV
GLQKENGQLDPDTIRDKISLTIGDAAQVDRLVKECAIPKNTPEKTAIHLFMCLDRNGVTYF
HEF

>XquaOBP11
MKVVLFFLCVAIFESMAHHLPEDEKLKLKQIHESCQADPATYADENKLKRLSEFIDDEQVG
THMLCMSIK
EGLQDEDGDLDEDLIRSKVALVTHDQSKVDDYVKKCAIEADNPEKTAILLVLCFVNNNIH
YYHQL

>XquaOBP10
MKYLFIAALLGFGVVVINGMTEKQLVAAVKVVRNVCQPKSKATDEDITKMHEGNWDIAH
SAMCYMYCSLN
MYKLMSKDNSLNYEGAVAQLKLLPDRYRASSEKCINQCKESAITVEDKCIAGYEIAKCIY
YCSPDDYYFP

>XquaOBP9
MKNSVDVMLLLISIGIFTCFVQAEINLTEEQKQKLAVHHKACTVPARDEEFIKKLIDGDLID

DEQFKDYL
FCISKRIGFQDASGVVQRAVIIEKLRKSIEDPSKAEEYTDKCLANTSSSPTETVFKVVACINSI
NPKSSI
FSK

>XquaOBP8
MNRIVVFLYLVFITQSVVFSALTEKQMNATKKLIRNTCLNKAKPSSDQVDGLQKGNFVDD
KNLQCYTYCV
LNTYKLIRKDNSFDWEGGVAALEANAPTNIAGPGAKTIVNCKDAVKTATDKCMAAYEIAR
CIYDDNPSNY
FLP

>XquaOBP7
MKFFVALVSLISVVLVAEAGLTDEQKQKLIAHHKECGAQSGVDNELVTKARKGEFIDDPK
LKEHLFCVAK
RIGFLNEAGVIQEQLLKSKLSAALGDEALAQKLVSECAIKKDTPQDTAYETVKCYFEKSPT
HISIV

>XquaOBP6
MFNKILVFLVGCYLPHIMALSKEEMKDLAQQLHNTCVTQTGVSEDLIKAVNNDGTFSDDE
NFKCYTKCIM
QESGIMDDEGLVDVEAAVAMLPEEYKDFEAIIRDCGTKKGSTACENAWLTQKCYSQHPRY
HLV

>XquaOBP5
MKFFVALVASISVVLVAEAGLTDEQKQKLIAYHKECGAQSGVDKELIAKARKGDFSDDPK
LKEHLFCVAK
KIGSFNEAGEIQQQVLKPKITAALGDEALAQKLLDECAVKKDTPQDTVYAAVKCYYEKSP
THISVV

>XquaOBP4
MMKYYVSVVLIAILAETMAISDEFKNMLQTLHDTCVPAVGVSEDVLIRARKGDFDDDDK
LKCYMRCLFQQ
LEGLDSDGKVKIDHLITHLPDEFRDALTPIIRKCGVLVGSNECETAFMFCKCSFDQDPSLFF
LP

>XquaOBP3
MLKFVVFMSVCVALSRGMSDEMKELMDSLHKTCTASTGATEDVVAKAQKGDFTEDENL
KCYMKCTLDEVG
LVEDDGKIDIEGMIAMLPDEVKDTLDPILRKCGATSVGANACETVFLLYKCTFNENPSMYF
LP

>XquaOBP2
MRNKYICVLTLINLFCPIAFGLSEEMQELANMLHSTCIGETGVSEENIQKAKTGDFVDDEK

LKCYIMCTM
AQMACINENGIIDVEATIAVLPEEFQEMAAPIIRKCDTQKGKSPCENAWLTHKCYYNENPQ
GYFLV

>XquaOBP1
MLSSTYFMFIFLLLETAMVQAVLEESEYSEQLENLSKTLHASCAALTGTDDSMIEMVKKG
DIPDNPTLKK
YVKCLLISSTAMDEDGELNKELMVDLMPEKHRSEISKTIDTCTPKVKAVATLEDKALTFLR
CLMSESPDI
IPIF

>AmelCSP5
MKIKILLFFTILALINVKAQDDISKFLKDRPYVQKQLHCILDRGHCDVIGKKIKELLPEVLN
NHCNRCTS
RQIGIANTLIPFMQQNYPYEWQLILRRYKIMKYY

>AmelCSP6
MKIYILLFVLVTITCVIAEDYTTKYDDMDIDRILQNGRILTNYIKCMLDEGPCTNEGRELKK
ILPDALST
GCNKCNEKQKHTANKVVNYLKTKRPKDWERLSAKYDSTGEYKKRYEHGLQFAKNN

>AmelCSP4
MKTILIALVPVCFLLGEVFSEDKYTTKYDNVDIDVVLNTERLLNAYVNCLLDQGPCTPDA
AELKRNLPDA
LENECSPCSEKQKKIADKVVQFLIDNKPEIWVLLEAKYDPTGAYKQHYLQNRVKEESY

>AmelCSP3
MKVSIICLVLMAAIVLVAARPDESYTSKFDNINVDEILHSDRLLNNYFKCLMDEGRCTAEG
NELKRVLPD
ALATDCKKCTDKQREVIKKVIKFLVENKPELWDSLANKYDPDKKYRVKFEEEAKKLGINV

>AmelCSP2
MASAIKALLIVCALFIYTVTAETEEGQSGRSRVSDEQLNMALSDQRYLRRQLKCALGEAP
CDPVGRRLKS
LAPLVLRGACPQCSPEETRQIKKVLSHIQRTYPKEWSKIVQQYAGVS

>AmelCSP1
MRHNYIVILILSLLTWTYAEELYSDKYDYVNIDEILANDRLRNQYYDCFIDAGSCLTPDSVF
FKSHITEA
FQTQCKKCTEIQKQNLDKLAEWFTTNEPEKWNHFVEIMIKKKDEGA

>BmorCSP4
MKVLIVLSCVLVAVLADDKYTDKYDKINLQEILENKRLLESYMDCVLGKGKCTPEGKELK
DHLQEALETG

CEKCTEAQEKGAETSIDYLIKNELEIWKELTAHFDPDGKWRKKYEDRAKAKGIVIPE

>BmorCSP13
MKLLLVFLGLFLAVLAQDKYEPIDDSFDASEVLSNERLLKSYTKCLLNQGPCTAELKKIKD
KIPEALETH
CAKCTDKQKQMAKQLAQGIKKTHPELWDEFITFYDPQGKYQTSFKDFLES

>BmorCSP10
MACVAVTWARPESTYTDKWDNINVDEILESNRLLKGYVDCLLGKGRCTPDGKALKETLP
DALEHECVKCT
GKQKSGADKVIRHLVNKRPDLWKELAVKYDPDNIYQARYKDKIDAVKGSA

>BmorCSP12
MFMLFIISFIIVPVLKCCGTETSTYTTQYDEVDIKEIMGNERLLVAYIGCLLDKNPCTPEGKE
LKRNIPD
ALQSDCSKCSDKQRENADAWIEFMIDNRPEDWTKLEER

>BmorCSP14
MKSSLFCVLVLTVVVSSSRQQSYPRNDNININAILQNDRILLGYFKCVMDRGPCTKDGKTF
KRALPEALP
TACARCSNKQKAAFRTLLLAIRARSEPSFLELLDKYDPSRSNRELLYTFLATGL

>BmorCSP6
MKSLIVLSCLLAACLAADLSKYENFDVEPIVTSDRLLKAYINCFLDKGRCTPEASDFKKAL
PDTIATNCG
KCTEKQKANVRKVIKVIQQKHSTEWEKLVKKHDPSGKHRADFDKFLLGS

>BmorCSP3
MNSLIAFCLFAVLAVALARPDDKYTDRYDNVNLDEVLSNSRLLQPYIKCILDKDRCAPDA
KELKEHIREA
LETECAKCTEAQKKGTRRVIGHLINNESKSWNELTAKYDPENKFTAKYEKELREIKA

>BmorCSP5
MKTVIVCLLALTAVALARPEQYTDKYDTVDLDQLISNRRLLIPYVHCILEKGQCTAEGKEL
KSHIKEALE
TNCAKCTKAQKGGTEKMIGHLINHEAEFWEELKAKYDPTNEFTKKYETELKRVTA

>BmorCSP7
MKGFYVLCFALFAAVYCKETYSSENDDLDIEALVGNIDSLKAFIGCFLETSPCDAVSGDFK
KDIPEAVAE
ACGKCTPAQKHLFKRFLEVVKDKLPQEYEAFKTKYDPQGKHFDALLSAVANS

>BmorCSP9
MRAVIFLYTCVFVVVGQDINAMMSMPKYDERYDYLDVDDIFRNKRLVRNYVDCLINAQR

CTPEGKALKRI
LPEALRTKCIRCTERQKRTSVKVIRRLKNEYPEEWAKLASRWDPTGDFTRYFEDYLAKEH
FNTIPGSGPT
VNVLSLQTTPPPPPPPPSRPASVFTNPPPPVMSTSPRPVVLNRFRR

>BmorCSP2
MKSVILICFLGVATVVIARPKTPFDNINIEEIFENRRLLLGYINCILERGNCTRAGKDLKSSL
KNVLEEN
CDKCSEDQRKSIIKVINYLVSSEPESWNQLKSKYDPEGKYLIKYEAKMESN

>BmorCSP8
MKTILILCALVSVVVCRPEEYYSSQYDNFDVEQLVGNLRLLKNYAKCFLDQGPCTAEGTE
FKKRIPEALR
TKCAKCNPKQRHLIRTVVKAFQTKLPDLWEELAIKEDPKGQYKHEFTAFINAMD

>BmorCSP1
MKVLIVLSCVLVAVLADDKYTDKYDKINLQEILENKRLLESYMDCVLGKGKCTPEGKELK
DHLQEALETG
CEKCTEAQEKGAETSIDYLIKNELEIWKELTAHFDPDGKWRKKYEDRAKAKGIVIPE

>BmorCSP11
MKLTSFLLVGMAMVSAEFYSSRYDDFDVKPLVENDRILQSYTNCFLDKGPCTPDAKEFKK
VIPEALETTC
GKCSPKQKQLIKTVIKAVIERHPEAWEELVNKYDKDRKFRPSFDKFINEDD

>BmorCSP16
MIEWKRFKILHFLSYLGLLVLVVVCAAQQNRPQVTDTALDEALNDKRFIQRQLKCALGEA
PCDPIGKRLK
TLAPLVLRGACPQCSPQETKQIQKTLSYVQRNFPQHWAKLVRQYAG

>BmorCSP15
MIENFYSKCTISKSVLFLCLIFLPYALNQKYYDSRYDYYDIDHLVQNPRLLKKYLDCFLGK
GPCTPIGRL
FKQVMPEVITTACAKCTPTQKRFARKTFNAFRRYFPETLMELRRKFDPESKYYDAFEKVIT
NA

>TcasCSP17
MHCLLQFCLLAAIFTCVKPQLTRISDEAIESTLNDRRYLLRQLKCATGEAPCDPVGRRLKS
LAPLVLRGS
CPQCTPQEMKQIQKVLAFVQKNYPKEWNKILHQYAG

>TcasCSP20
MKIIILAVLIATAVAATYDVYPTKYDNVDIDAILHNKRLFDNYLQCLLKKGKCNEEAAILRD
VIPDALIT

GCRKCNDHQKVSVEKVIRFLIKERNSDWQQLISVYDPKGEYQTQYAHYLEKI

>TcasCSP3
MLFTVFLVLTCAHVVFLEEYVIPDNIDIDDILSNERLLKNYVNCLLDKGRCTPEGKKLKSTI
PEALSTDC
AKCNEKVKANVRKVLHHLIDNKPDMWKQLEAKYDPSGEYRSKYKDELEKNGIHV

>TcasCSP4
MFKVLFVVFACVQAYVYAEEYTVPQNIDIDEILKNDRLTKNYLDCILEKGKCTPEGEELKK
DIPDALQNE
CAKCNEKHKEGVRKVIRHLIKNKPSWWQELQEKYDPKGEYKSRYNHFLEEEGLN

>TcasCSP5
MTAIVFLLALACLKTYVSSQEYLVPQNIDVDEILKNDRLTRNYLDCVLGKGKCTPEGEEL
KKDIPEALQN
GCAKCNEKHKEGVRKVIHHLIENKPNWWQELESKFDPQGEYKKKYDELLKKEGLAN

>TcasCSP2
MFATSALFAFICIQGLVSAEEYLVPQNIDLDEILKNDRLTRNYIDCILGKGKCTPEGEELKRD
IPEALQN
ECAKCNEKHKEGVRKVLHHLIKNKPNWWQELEAKFDPKGEYKQKYNKLLEKEGLQA

>TcasCSP6
MFLAIVLVVCACTNVLSEEYTNQYNDELDAALKSERLMKSYFECLLGTGKCTPSGEELKK
DIPDALKNEC
AKCNDKHKEGIRKVIHYLVKQKPEWWEQLQKKFDPQGIYKKRYQNYLDKEGLKA

>TcasCSP9
MKTLVLVLFVAVLSVVFAADKYTTKYDNIDLNQILKSDRLLKNYVNCLLDRGKCSPDGQE
LKNNLADALQ
TSCSKCSQRQKDGSRTIIRYLIKNKRDWWNELEAKYDPTGIYKNKYADELKAEGIVL

>TcasCSP1
MKTLVPLLFFVIAIASSLAENSKYTTKYDNVDLDEIIKSDRLLKNYVNCLLEKGKCTPDGA
ELKRHLPDA
LHTECSKCSETQKNGSKKIMRHLIDHKRDWWNELEEKYDKEGEYRKKYEAEIKGKKD

>TcasCSP7
MKTFVLVAFAAVLGLALARPQEKYTTKYDNIDLEEILKSDRLLKNYFNCLMERGTCSPDG
EELKKALPDA
LHSGCSKCTEKQKEGSRKIIHYLIDNKRDWWNELEAKYDKDGVYRQKYKDVIEKEGIKL

>TcasCSP10
MKLFVINFILMSLVYMSFGASVPYETVDIDKLLADDKMVTEYMACLRGEGPCNPAEKDL

EEHIPLVLGNY
CADCNDKQKNFVIKLATFVIKNRFDEWRQVQKRFDPDLSHADDFNKFILGS

>TcasCSP11
MYSYLIPLYLFLFVHYGWSEDTTHKYTTKYDNIDLENVVKNERLLKSYVDCLLEKGRCSP
DGLELKKNMP
DAIETDCSKCSEKQKEGSDFIMRYLIDNKPDYWKALEAKYDPDGTYKKRYFESQKDEVS
KVEA

>TcasCSP12
MKLISAVILCAFLVAVSAAENKYTNKYDNVDVDKILNNDRVLTNYIKCLMDEGPCTSEGR
ELKKTLPDAL
SSGCTKCNQKQKETAEKVIRHLTQKRARDWERLSKKYDPQGQYKKRYEEHVATSRAA

>TcasCSP16
MPLVKSLVVVVLLIGVVYQVQGQLGLAGNNYIEKQLLCALDKAPCDALGNQIKGALPEII
GKNCERCDSR
QVANARRIARYVQTKHPDVWNALVKKYSV

>TcasCSP18
MRFFVIFFVACVSVALARPEDQYTIKYDNVNLKEILQSDRLTENYVNCLLEKKPCTPDGEE
LKRVLPDAL
KTSCAKCTDKQKQGAKTVIQHLYKNKQDWWKQLEAKYDPEHTYVKAHEDELKAL

>TcasCSP19
MKFFIAFLMLLGAVWCEQYTTKYDNINVDEILASERLLKNYFNCIMDRGACTPDADELKR
VLPDALKSDC
AKCSEKQKEMTKKVIHFLSHNKQQMWKELTAKYDPDGIYFEKYKDKFDS

>TcasCSP8
MIFKIHFLVFGALLTYVSSVEYLILREIDTILKNDQMTRNYLDCVLDKGKCTKEAEKLKKG
ITETMKNGC
VKCEQKQKEDVHKVFQHLMIHRPNWWHELETKFNPHHEIKLQHLHQSKFNPHEEVKLQ
HLHQFPHHDFLE
REGFIR

>TcasCSP14
MKTFVILFFGVFFIIFSDFVNGKTLHRSTRDDKYTTRYDNVDVDRILHSKRLLLNYINCLLE
KGPCSPEG
RELKKILPDALVTNCSKCSEVQKKQAGKILTFVLLNYRNEWNQLVAKYDPDGIYRKQYEI
DDDYDYSELD
SAKK

>TcasCSP13

MIPLIAIAGILAVSAAPAEFYESRYDHLDVESILNNRRMVNYYAACLLSKGPCPPQGVDLK
RVLPEALQT
NCAKCTEKQRTAAYRSIKRLKKEYPKIWEQLRAVWDPDDVFIRKFETSFESGKPSGVISTN
TSPPSPILS
NRFGENEEADAASNVISSTPLPPTTSTTTRTTLTTKFTTKPSTKPTNKPVVVTKPPQAPPFAT
VGANLQA
TVSFGTNLVGGIVRSLGTLGSRVVESGTKLANMVISAAIRP

>CbowCSP1
MKYFVIILSVLIIAAAVGEKYTTKYDNVDIDSILNSERLIKNYMDCLMERGPCTPEGKELR
DNLPDALKT
ECHKCSDKQKEVSKKVLRHLVKNKRKEFDELTGKYDPEGVYKNKYKEDLAKEGIIV

>CbowCSP2
MSRLLFSCLLLGVVVFVLADEKKSRANVPKYTTKYDNVDLDAIINNDRIFRNYIECCLGK
RKCTPDGLEL
RNHIRDAMDNECDKCSETQKKAMKKVGRKLYKEKPEWWKELCDHFDPDMKYRTKYQK
FIDEALAEEDK

>CbowCSP3
MKFSLMLCVLVALLVFADARPEDKYTTKYDKVDLDAILQNERLLRSYIDCLLDKKKCSKD
GEELKKILPE
ALKSKCAKCNENQKKGAKKVIRYLLKEKRAWWDELEAVYDPEGIYRKTYEKELKEEGIQ
I

>CbowCSP4
MNIPLGICFLMMVIFVRAGEKYTAKFDNIDYEEILRSERLLKNYIFCLLDKGPCSPDGLGIK
NILADALE
TECSKCSDRQKEGSTKVIRFLIENHAGWWKELTEKYDPDGIFMQKYRDQWNSNN

>CbowCSP5
MKASALFFVAFFAVVATKPAEKYTTKYDNVDLDTILKSDRLLKNYVNCLLDKGNCTPDGA
ELKKVLPDAL
QTDCSKCSDVQKRGSKKIIRYLIDNKAEWYKELEAKYDKNGVYKKKYDKELEDAKV

>CbowCSP6
MGLSKVLFCFFVIVTVAFCQTYNTKYDHVDIDSILANKRVLSSYIKCIMDEGPCTSEGREFR
KHIPEAIT
NNCAKCSDAQKRIIRKTSRFIRRERPQDWDRVSRKYDPQQKYTANFNKFLSEN

>CbowCSP7
MKIVAALLFISLLAFVTAQNSYSRKYDNVDVDKILKNERVLSNYIKCLLEEGPCTAEGREL
KKTLPDALA
NECEKCNPNQKNTAEKVMKHLMSKRARDWERLSKKYDPQGNYKKRYQHLVEKVAN

>CbowCSP8
MMNMRTFLVFTFVCVATKAQILSRNNYIQKQLLCTLDRAPCDHLGSQIRDALPEIIGNNCK
SCDQRQTAN
AKRIAVFVQSKYPDVWNALVKKYSRLE

>CbowCSP9
MEVSYHLIVCIVVFSYVSEDRFVSTTSLNRVERAVEKYSNKYDKFDVAGVLASARLVKRY
GDCLMDRGPC
PPEGRFLKDIVPDAIATECSKCNNIQKKQAGLILQHLLLHYRPLFLELCDKYDPTGKARKQ
YGIDTNEAD
EYEDYDEA

>CbowCSP10
MRTIILLAVLGVCVSAYNTAFDRVNVEDVLKNKRLLKRYVDCLLGVPKTCTKDGQLLKD
TLPNALKTKCE
DCSEPQRKGAKRVANYLIDCKPKWWSDLAKIYDSDGIYTKQYHDELLAEGINIDGSSKDT
EHKTQCYN

>CbowCSP11
MQIKYQDAILVTVVLCAIVTIVNALPQSQAISDEALESTLKDKRYLLRQLKCALGEAPCDP
VGRRLKSLA
PLVIQGSCSQCTPQEQRQVRKVLSYMQVNFPKEWNKVLKQYSG

>CbowCSP12
MKEGKARKLSQCYSFHGHEQCKYISLKMFLPFVVLSCLITLSISAVPEKSRYTTKYDNINLE
EIIHNDRL
LKNYVDCLLDKGRCTPDGLELKKNMPDAIETDCSKCSDKQKEGSEIMMRYLIDNKPEYW
NPLQEKYDPSG
SYKKRYLDAKKTEVSVEPIVKS

>LoryCSP10
MQIVVLFTFLSGILFSFCLAKPAKSYTNKYDNVNIDEILNSDRLLKNYMNCLLDKGRCTPD
GAELKKNLP
EALEDDCSKCSEYQRNRGAKALKYLIEHKRSYYDQLERKYDPERKYRKRYEASFKREGI
NL

>LoryCSP9
MNSATLTCILVILILNICYGADEKYTNKYDNINIDEILQSDRLLNNYMNCLLDKGRCTPDG
AELKKNLPD
ALEDECSKCSEYQKDRGRKALKFLIEKRRGYFDQLEAKYDPDGKYRKRYEENMKKEGIT
L

>LoryCSP8

MKFRLLFFVAFIGIVVAANSKYTTKYDNVDLDEIIKSDRLMKNYVNCLLEKGKCTPDGAE
LKKVLPDALH
TECSKCSDTQKRGSRKIMRHLIDNKPEWWKDLEDKYDKEGAYKKQYREELKKEGIKL

>LoryCSP6
MKYLVLVLIATAVVVANARPDQYTSKYDNVDIDEILKSDRLLRNYVNCLLGTGKCTPDGA
ELKKNLPDAL
ETGCTKCTEKQREGSKKVIHYLIEKKRPWWDELAVKYDPTGKYTKKYEEEAKQQNN

>LoryCSP3
MFWIKAIFYIVVVFEIFYDKRSFCALLSRSKRAEMYTTKYDNIDVDSILASTRLLKNYVNC
LLDQGPCSP
EGKELKKYLPDAIATECTKCSEAQKKIAGKVFSHMLLKHRDDFEKITAKYDPERKIYNKY
LLDDKDYADL
EEA

>DhelCSP6
MRSYVLLCAIIFLNILHCFCNEKYTTAFDNVNVEEILSSERLLNNYFKCMMDRGSCPPDAA
ELKRNLPDA
LQTDCSKCSEKQKEVSKQIVKFLIEKKPNMWKELQEKYDPEQVYIKKFAQHLDIKV

>DhelCSP5
MKTLLLFGLCLVGVFAIDKYPTKYDNIDVDNVLKNKRLFNNYMQCLLKKGKCTEDGTLL
RDLIPDALLTE
CAKCSEVQKTKAEKVIKHIKKEHPQDWEDLLKVYDPEKKYVKSYQKYLDE

>DhelCSP4
MKFDIPLILIVTVASVKCVPVEYYATKYDHLDIERILNNRRMVNYYASCLLNKGPCPPQGT
EFKRILPEA
LETNCARCTEKQKFVTYHTIKRLKKEYPKIWVQLEAQWDPNHTFIPLFEASFIASSTSPSTT
STTVVPDL
GNRFGENKNETETFPYPAEAVASGATLLPNLQQTTAFSSSIQSTHSTNTQHTTPPYLSYSTT
KRYNVKQQ
QNTVSYGFNIVGDVIKNIGIFSVRVAETGKQLANVLISSLHI

>DhelCSP3
MKAVFALVTLLFAATLALEKYSTKYDNVDVDNILHNQRLYNNYIQCILKKGRCTEEGKFL
QELIPDAIIT
ECSKCSDKQKEQGEKVIRFIRKEHPKDWDALIAVYDPEGKYQEAYKKYLDKV

>DhelCSP2
MKTIIFGLCLVVAVCAIDKYPTKYDNIDVDNILNNKRLLNNYMQCLLKKGKCTEDGSLLR
DLIPDALLTD
CAKCSEVQKTKSEKVIRFIKKNRGDDWDELLKVYDPDKKFVKVYQKYIDE

>DhelCSP1
MRRVVAFVFLVTFIYANEQPKYTTKYDNIDLEEIVTNDRLLKNYVYCLLDKGPCTSDGQE
LKRNMPDAIK
TDCAKCSEKQRTGSEYIMKYLIDHKPEYWMPLEEKYDPEGSYKKKYLESKKKNEKETF

>DhelCSP7
MKTVLLSLICSLVLVLVASEDKYTTKYDNIDVDGILTNHRLFDNYVKCLLGRGKCSEDGAT
LKKYIPDAL
LTECAKCSALQKKHGEKVLRFMYKNRRDAWDSLLKVYDPESKYQSSYRKYIDELEAPKD

>MaltCSP12
MRVLCQMVLTAMALLVLGSIYERSVSAAMFRTKREEKYTTKYDNFDVAGVLASQRLVRV
YLNCLLETGPC
TPEGKELKKYIPDAIATECKKCSPVQKKQAGIVLYHILLNYREDFHKLTDKYDPEGKARKI
YGIDQDDDD
DDNLDQ

>MaltCSP11
MNQYLLVLFVALIGVVASQKYTTKYDNVDLDQIIKSDRLLKNYIDCVLDRGNCTPDGQEL
KKNLPDALLT
DCSKCSETQKNGSTKILRHLVKNKRPWFDELAAKFDPDSSYRKRHEEEFAKEGIV

>MaltCSP10
MLYLSSLICLVCISSVIGQKYSNKYDHIDIDRILSSKRVLNNYIKCILDQGPCTPDGREFRDHI
PEAITT
NCVKCTDAQVKIIRKTSAHIMNNRPEDWEKIKNKFDPEEKYKDSFMKFIDG

>MaltCSP9
MKVVLVFAVLCAFAYAEYTTKYDNIDLDEILRNDRLLKSYFQCLEGTKSCTKDGQELKNIL
PDALKTKCS
QCNETQKQGAEKVIRYLIDNKPEWWKKLEAIYDPSG

>MaltCSP7
MNELVYLAIFLSAAVVGEEMYSSKFDNIDYEEIIKSDRLLKNYANCLLDKGGCTAEATELK
RILPDALKT
DCAKCSERQKKGAKKIIQFLVNNKPDLWEQLMAKYDPDGEFKEKYEGDWLNED

>MaltCSP6
MVCLLPVLVTTGTLLGLIDGANAQYYASKYDHIDVDAILNNRRIVNYYAACLLTKGPCPP
EGIEFKRILP
EALRTNCQRCTEKQKTVTLRAIRRLKKEYPKVWAQLEKQWDPDGSYISKFESTFGAKSTE
SIPESSVQIV
NRYASPEDNKTIENALSQIFTTPLPTSTSTSTSTTSTTTATTSTTSTTAKTTTTSKATTVSTKA

APVTKT
TTHRVSTINIIKNINTISSSTKPTKRPRPSIGQSIQATVSIGTNIVGDLVRGLEAIGNRVVETGA
EIAGV
VLKNIARPL

>MaltCSP5
MKLVVFLFFVALCGITYGRPDDKYTTKYDNIDLDEILKNDRLLRAYVDCLLGTKKCTNDG
EELKKVLPEA
IDNECAKCSEKQKDGAKKVVHYLIDNKSDWWKELEVVYDPTGKYRKKYEEQAKKEGL
KI

>MaltCSP4
MTVRYAVVTLVLSLVIAVEVQGQRGLTGNNYVERQLLCALDKAPCDNLGRQIKDALPEIIG
NNCKSCDQK
QMANAKRIARFVQNKYPDVWNALITKYGAE

>MaltCSP3
MRAYVLAVFVVLVGVVASQKYTTKYDNIDLDEIIKSDRLLKNYLDCVMERGNCTPDGQE
LKKNIPDALVT
DCSKCSDRQRDGTRKILRHLVMNKREWFDEVAGKYDPEGVYRKKNREEFAKEGVEI

>MaltCSP2
MLLIAVTMKVSLLFVVVLGLVVSIRADEKYTTKYDNVDLDEIIKSDRLLKNYVNCLLDKG
NCSPDGTELK
KVLPDALLTDCSKCSETQKNGSKKIIRHLIDNKADWYKELEAKYDKEGVYKKKYEEELG
LKKEKREEAKE
EKKEKEEKKEKEEKKEKEVKDEKKD

>MaltCSP1
MHFKCQIALLLCIAVMTIVTEAFPQADTERSEVSDEALESTLKDKRYLMRQLKCALGEAP
CDPVGRRLKS
LAPLVLQGSCSQCSPKELNQIRKVLSYMQINFPKEWNRVLKQYSR

>MaltCSP8
MLKMIIYFLVAIGLITYATSTVTERTKYTTKYDNIDLDEIIHNERLLKNYVDCLLEKGRCTP
DGLELKKN
MPDAIETDCSKCSEKQKEGSEIIIRYLIDNKPEYWNPLQDKYDPTGSYKKRYLDAKKTEVN
VEPIVKS

>AchiCSP16
MNQYLLVLFVALIGIVATQKYTTKYDNIDLDQIIKSDRLMKNYIDCVLERGNCTPDGLELK
KNIPDALLT
DCSKCSDTQKNGSRRILKHLVQNKRAWFDELAAKYDPDNAYRKRNEQEFAKEGIVF

>AchiCSP15
MVCLLPVLVAAGTLLGLIDGANAQYYASKYDHIDVDAILNNRRIVNYYAACLLSEGPCPP
EGIEFKRILP
EALRTNCHRCTEKQKTVTLRAIRRLKKEYPKVWVLLEKQWDPDSSYISKFESTFGGKPAE
SSPTPSVQIV
NRFASTENDNKTVENTISQLFTTPLPTSTSTRTSTSSTTTATTRTTSTATSTTTRTTTRTTTRTT
TPTTK
TTTTKPTTVSTKATPVTKATIDDFGTISTIKLNINTISASAKPKKRPRPNIGQSIQATVSLGTNI
VGDLV
RGLGAIGNRVVETGAEIAGVVLKNIARPL

>AchiCSP14
MITLNELVCLIIFFSGFAVPEEKYSSKFDNIDYEEVLRSDRLLKHYANCLLDKGSCPPEGAEL
KRILPDA
LETDCAKCSEGQKRGAKRVIQFLVINKPDTWEQLMAKYDPNGEFKEKYEREWLNED

>AchiCSP13
MKVVLLLAALCAFAYARPEYTTKYDNIDLDEILRNERLLKSYFQCLEGTKSCTKDGQELK
NILPDALKTK
CSQCNENQRRGAEKVIRHLIDNKADWWKKLEVIYDPEGSYRKAYQEEAEKRGIQLPK

>AchiCSP12
MLKMIIYFLVAVGLTAYTTSTVTERTKYTTKYDNIDLDEIIHNERLLKNYVDCLLEKGRCTP
DGLELKKN
MPDAIETDCSKCSEKQKEGSEIIIRYLIDNKPEYWSPLQEKYDPTGSYKKRYLDAKKTEVN
VEPIVKS

>AchiCSP11
MKLVVLLLFVALCGFAYARPDDKYTTKYDNIDLDEILKNDRLLRAYVDCLLGTKKCTNDG
EELKKVLPDA
IDNECAKCSEKQKDGARKVIHYVIKNKRDWWTELEAIYDPEGKYRKKYEEQAKKEGIDL

>AchiCSP10
MRAYVLAVFVVLVGAVASQKYTTKYDNVDLDQIIKSDRLLKNYLDCILEKGNCTPDGQEL
KKNIPDALLT
DCSKCSDKQKDGTKKILKHLVKNKREWFDEVAAKYDPEGVFREKNREEYAKEGITF

>AchiCSP9
MYFFSGLVCLICISSVIGQKYSNKYDHIDIDRILSSKRVLNNYIKCILDQGPCTPDGREFRDH
VPEAITT
NCAKCTEAQINIIRKTSVFIMKNRPEDWEKIKNKFDPQEKYKDSFMKFINGHN

>AchiCSP8
MKVSLLFVVVVALVVCARADDKYTTKYDNVDLDEIIKSDRLLKNYVNCLLDKGNCTPDG

AELKKVLPDAL
LTDCSKCSETQKKGSKKIIRHLIDNKPDWYKELEAKYDKEGVYKKKYEEELELKKETKEE
EKEKKEEEKE
KKVEKEVKIEKKE

>AchiCSP7
MKVICQTVLSAMALLVLVSNEERSVSAAAVSRAKREEKYTTRYDNFDVAGVLASKRLVR
VYLNCLLETGP
CTPEGRELKKYIPDAIATECIKCSPLQRKQAGIVLSHILLNYRDDFNKLSQKYDPEGKARK
MYNIDQDGD
DDYQDLEEA

>AchiCSP6
MKVVLFLTVIFVIGCLCQKYTTKFDNVDLDNILKNERLLRNYMNCLLDKGKCSSDAVELK
KVIPEALENE
CEKCSEKHRDGVKKVIKYLVENKRDYWNELLAKYDPEGNYRKKYEELSKKEEVQI

>AchiCSP5
MHFKCQVALLLCIALTAIVTEAFPQADTDRPAVSDEALESTLKDKRYLMRQLKCALGEAP
CDPVGRRLKS
LAPLVLQGSCAQCSPKELNQIRKVLSYMQINFPKEWNKVLKQYSR

>AchiCSP4
MKILCLSVVIVLLSGYANTQKFTDKYDNVNLDQILRNDRLLINYVNCLLDKGRCTADGLE
LKKAVPNALQ
NGCNMCSEKQRNGATKVIRYLIDNKRAWWNELEQKYDPQGNYRRQYEEEGKRYGVHL

>AchiCSP3
MQPFSAVIFCALVGLVLADNKYTSKYDNVDVEKILTNDRVLTNYIKCLMDEGPCTPEGREL
KKTLPDALS
SNCSKCNAKQKDTAEKVMKHLMSKRAKDWERLTKKYDPQGLYKQRYEEHLSKT

>AchiCSP2
MTVRYTVVSLVLSFIIAVEVQGQRGLTGNNYVEKQLLCALDKAPCDNLGRQIKDALPEIIG
NNCKSCDQK
QVANAKRIARFVQTKYPDVWNALITKYGAN

>AchiCSP1
MHLTLIFVFTLVAGALSEEYTSKYDNIDLDAIMKNERLLKNYIDCLLDRGKCSNDANELK
KHIPEALETE
CAKCSERHKGGVRRVIKFLAENRKEWWNELVQKYDPDGTYRKKYQDLSKKEHVSM

>BhorCSP2
MKAYLSFVALLVAVACARADDDKYTTKYDNVDLDEIVKSDRLLKNYVNCLLEKGNCTPD

GTELKKVLPDA
LLTDCTKCSDTQKKGSKKIIRHLIDNKADWYKELEAKYDKDGVYKKKYEEELELKKE

>BhorCSP3
MDALYELVYLALLFSSVVAEETYTTKFDNIDYEEILRSDRLLRNYINCLLDRGGCTAEGKE
LRRILPDAL
ETDCSKCSETQRKAAKKVIQHLVNNKADMWEELMVMYDPDGEFKKKYEGEWLNED

>BhorCSP1
MKLVVLLLFVALCGMAYGRPDDGKYTTKYDNIDLDEILKNDRLLRAYVDCLKGTKKCTN
DGEELKKVLPE
AIDNDCAKCNDTQKNGARKVIRYLIKNKRDWWNELEVIYDPTGKYKKKYEEEAKKEGL
EV

>DmelCSP56a
MSFWWMIQSLLILWTVSVGSKKSNYEVRFESIDAVKGSTETLFLYQLRLLGRNRMINGTLI
FLEDLDETF
DVLFESHAFKNGYWVKGIVNAAASKPCEFFNRYYISFFLVKSTESNLPTTGAEMCPFRKG
TYFVKNGVVS
TEDWPPIVFKGLNRFTISYLKNGECVGGVQLTISIAEIIT

>DmelCSP46a
MSLELHKVLLVLSIAHSALVRAGLECRIESISKVFGDNETLFEFNFRVIGRQRLLNGTLNFH
VDLDDDYE
MSNEVLALKDGEWESTSVSARFKTCKYMAVIYDKYFAVSFKDSNIPKGTEACPIKKGEYY
ARNVEVIADN
WAHYAKLGLVRSNMLVRKNNVVYGGFDIVLVLSQKIV

>DmelCSP86a
MLKYTVLVLLIGIPKLLLQAQMSYEAIFVSVTSEENSKPFDLSNLRLIGRERILNGTFEILED
LDDEHFQ
ISVEIYTNPARDGNYKLLPMSVPRQGVCTFFKKYGFYFRDCIKNGINTDLFLNTTSCLFPK
GHYYLKNVT
INVQNWPKIMQRGLCRHIAFFYKNNVPMGSYNLTSSIEDRAPNFNLRPL

>DmelCSP84a
MKQFLFCVLIMLIGKTCALIPRTYETRFISITSNGTNLFDFSQIRFLGRERMANGTFELKEDL
DNESFSV
VGETFIDSVGDGEYKQLPFTAPKQSVCTALKAYWSYFEPSIKYGVKTDFPAHTHPCPLPKG
IYYIKDVVL
KNDNWPVIMPRGYLKAVANLFKNDEYGGSLEIVSQISDLS

>DmelCSP29a
MWHCELKVFLILWTVSQVYIPCWGKKFISRFESINGIEGEKETLFTCSVRLVGRERMLNGS
IMHQVDLDD
SFDVWMDILHFKNGEWAQGNIKVRTKPCDWFTNYFGKYFLPLVKDSNLPPIQEMCVFPK
GEYYLRITKIE
PQNWPPILYRGLNQFNINYVRDGKSTGGIQFVIDLEDSTL

>DmelCSP98a
MDAKIIILALVSLFYKANGAFYELSVADEEIFSSCPNPEPGTLDIHGLFDFSEFSTSLEADGLT
VSGNQT
LVWDIQRGDRVQLFIKLFYFDRGTWTSTAFSILSQDFCKTMYDKSNVLYEPWTGHVMND
VKDQCINAPGT
KLILDTYFLSLSASVTVPLREGRYKTTIKFRAFDSKGTERPTSICCEVIGDVFKIRN

>DmelCSP42c
MRTLMLFVFGFASSWAADYELLLEDPDIFSPCTEPPPGSIGFHDAFDIGDLVVDQDMDIIHL
SESVTSIW
DVEPTDRISARFAIMHYNRGSWEPTVFSMATPDFCASMFDENQSWFKYWTKHISNRDEV
MEKCFKTRGTV
IMHNPFDLQLRLTDIRGATLRGRYKAVVTFEAVDEKDVPRRNSICFEIRGEAEKIN

>DmelCSP93b
MDRQQVTLLNILISLTLQIISPSFALNHFFIPEREELFSECLDKPGFSYVNELADLSRFNRKK
DADGAMN
ISGNITMLWDVEPSNRVAVEVNIEKFEGGTWNPTLFKGGDKDFCKNFYDKNTIYYPFSTKH
VINKQQVKD
KCITTPGTVLVLEPFILKILINFAVPLSPGIHKAVIIFSAFDKSGVKRPRDTCIEIVGEIVNI

>DmelCSP93a
MGCETSVLCTLLIFFQIIGRSFEMSHDFVPIKDDLLSKCEDKPEMGYLDAFVDLSNFSRKR
GPGGVNISG
NITTIWDVDPSDVVEIDVSILKFEGDKWIPTIIKGNVKDFCKSFYDKNTLYYSYSTKHVVN
KKEAKEKCI
TTPGAILLWEPYLLKISFSYAVPLNVGRHKAVLIFTAIDKAGVKRDRDICMEIVGDIVNA

>DmelCSP75a
MKWLVIIVLLQLLEKIKCEQSYEVTNERLEPFEGDSQTLVLFDGLKTIGRERALNGSFKFLG
EMNNDDFK
VSVELYSSPNGDGEFKRMVMDVPQTSICECFKKFYVQFVQPSLKTGETTNFPVVDDDFCP
VPEGEFYVKN
VILNTQDWPSQVPRGIVKAIITFFSGGKNVGGLIVEVKIEDRQS

>DmelCSP38c
MFATLLILILGSTDILATDYILLVEDPDIYTPCTDGPPGSVGLNEAFDVSEMQVEMDEEGIH

VSGNITTR
WSLPPTYRISARMSVLHFNRGNWEPTVFNTLTPDFCDAMFNPNLFWYKYWFKNFENREE
IQEKCLATQGT
VLVYNPFVVVPRLNNVLGPTLKGRYKVVFLFEAFNEQDERQPSSVCFEITGDAEKIKN

>DmelCSP74a
MDFQFSVLCQILIFLTAAQPSIGGYFEYVLDDESVFSECFNTPPGYANVSGLFDVSTVNFEM
GPEGVHID
GHVTSTWDIQPTDRIEGRLNVVHMDRGTWQPTVLNMVCKDFCKTFLDPNQYWYNVFPK
HIINKDEARQKC
LNYKGTVYFVEPYTLQMHFGLGLTLPSGRNRMVINLVAIDENNVTRPNGICFEVKGDFFKI
E

>DmelCSP53b
MSQQLFRIWQLLVLMAFIPHSIEVSYEFTMEDERITSDCQNEPPKTLNIDGLFDMSNIDFEM
AEDGVQLS
GYKTVVWDIQPTDRVELQGSVQYFDRGTWQPTTLNMLVKDFCHVLFDKKQVWYDAYS
KHITNSAQINNTC
FRVKGSVIEFETYTIGLEFGSGIPLHRGRYAIRLKFRAFDKNGKVRPNEICFEIKGQFSKKSL
G

>DmelCSP53a
MLELILILNVVHLSLQISYEFEIEDESIYSDCSDVPPGTLNISGLFDLTNYTTTITADGLSVSG
NMTSVF
NAQPTDRIELTGNLLFFDRGAWQPTTLNMMVRDFCAVMYDKKQLWYTDWSSHVVNRDE
IKDNCIKVPGTL
FLIESYNMKLVFGSGIPLGTGRYSIRVQVFAYDQKGKKRPNNVCYEVKGNFYKESNICISK
PILITINVL
AESLKSQMSSTNHANI

>DmelCSP42a
MKATFTILVLQVVICLAGATEYQLTLDKDGLLAPCENQPGNPSGFEAMVDTSSLKVHNLG
SKVRIEGEQK
VVWKDVQPGDTLKVFGQVYRLDKGTWQKTMFTASSNNFCKNMFDKNQYWYNFWTKY
ISNSDEIKEKCLTT
PGAVLKYKDYELDLKTSLNVPNLDGRYKLVVQIEAFDKRNVRRPVPICIEFRGTAGQV

>DmelCSP42b
MIFVLLLLLGVTSSWATDYELLLEDPDIFSTCTDGPPGSINIRQALNLDDIVIDQKGDILHVS
GNATVVW
DVQPTDRITARLDVFHFNRGTWEPTVFSMATQNFCSIMYDKNQYWYKYWTRFITNRHEV
EKKCFRGPDTV
LVHEPFDLILKFENFRGPLLRGRHKLVILFNALDERNIPRPNPICLEIIGEPLKLQ

>DmelCSP38a
MISISTILALVVSSVWATDYTLEFEDSDLYSECSEKLPGAIGLREAFDMRNIVTELDIDGLHL
SGNCTTI
WDVPSTDRISLRMTVMHFDRGTWQPTVFNTYARDFCAVMFDKELSWYKYWLKYFANRE
EISEKCIGTKGT
VLVYKPFIVKPLIQNVIGPIYRGRVKAIFNFESFDKNNVKGATDVCFEVRGQVEKIK

>DmelCSP38b
MIRGLVILVLALANTWATDYNALIDDEGIYVKCSEAPAGTLGPRDVFNIDNMVMHMEPEG
IYVSGNMTVK
LNFLPSDRISARFSVMHYERGSWQPTMFNLHSPNFCEVMFDEDQYWFKYWFRYIRNKEEI
REKCLKVKDT
VLVYDEFLMVLHLENVNTSNLQGRYKAVITLEAFDEHNVRRPSSLCVEIRGDLERVT

>DmelCSP87a
MGSSLCPVSYLAVLAIIVLTSNITVQAKRTFRIQKLEKVTEDTSYLRSRLRIAESEENELKVS
GYLDLNQ
RLDNDWTVVLKVSRSPDSDGDYEKVLTFEMQLCDFMKSYYKDIFYERIKEYSNAPHPSSC
PLPKERYVLE
DYPFNVKLLKKLMSPGFYRIKYTLKNEETKILSYVLDLELEEN

>DponCSP1
MVPLAGSFVLIAVLTLLIGEENGANARSVKRSAQTYTTKYDNIDIDQILASNRLLKNYVNC
LLDKGACTQ
DGKELKKYLPDAIATECSKCSQAQKKIAGRVLQALLLNHRDDWELLTNKYDPDGNFQKK
YLQEDEDYSDL
EEA

>DponCSP2
MKVVLLLVVVVGVAYGEEYTSKFDNVDLDQILSSDRLLGNYMNCLLDKGKCTPDGTELK
KNLPDALENDC
SKCSAKQRDGAKKVIRYLIDNKRDYWDQVAAKYDPEGKYYKKYQEEAKKENIKL

>DponCSP3
MWKLVLLGSLLICIGQTLAEVTEKSQYTTKYDNVDINEVVHNERLLKNYVNCLLDRGPCS
PDGLELKKNM
PDAIETDCSKCSDKQREGLEAMMRFLIDNKPEYWNPLQEKYDPTGSYKKRYLDAKRAEV
AIQPAEKTP

>DponCSP4
MLLIISVLIGMALDLTDAKPAAKNYASKYDHIDVGAILNNRRMVNYYSACLLSQGACPPE
GVELKRILPE
ALQTNCARCSEKQATIALMAIKRLKKEYPKIWSELSAKWDPSDSFVKKFETTFESLHGPG
RRVESTTSAG
NKLDPSEADGNTIDANITQTSPEGSDRVNQPDTTTTPQIITTNPSFSTSTKPAFSSTKRPSPIP
GLVPFN
TFFTNPPIPIRPIVNLNLGGNIGATVKAIKQVEKMVADIALEKIGIIRSILRPWRKAKKTRYA

>DponCSP6
MALFPQYWLQFGALLLLLALADGQVLNGNVYVEKQLLCALDRAPCDNLGRQIKDALPEI
IGKNCKACDNK
QLSNAKRIARFVQNKYPDVWNDLVRKYGNPTN

>DponCSP8
MKIFIVVCCAFIGLVLADTPKYTTKYDNVDLEEIIKSDRLMKNYVNCLLEKGKCTPDGAE
LKRVLPDALH
TECSKCSDSQKKGSRKIMRHLIDNKPEWWTELENKYDKEGAYKKQYREELKKDGIKL

>DponCSP11
MAPFPQSWLQFGALLLLLALVQGQILNGNVYVEKQLLCALDRAPCDNLGRQIKDALPEII
GKNCKACDNK
QLSNAKRIARFVQNKYPNVWNDLVRKYGNPTN

>ItypCSP1
MKLIISFLLIAVAALSYADKYTSKYDDVDIDQILQSERLLRNYLNCLLDKGRCTPDGAELK
KNLPDALEN
ECSKCNESQXKGASKVIRYLIDNKRQYWDELAAKYDPEGVFFKKYEAEAKKDLLDQIGR
A

>ItypCSP2
MGGHRKSYLVLALVLVNLVSLNRAAESTTRAPISDDALEKTLSDKRYLTRQLKCALGEAP
CDPVGRRLKS

>ItypCSP4
MALLIFFVVILTVGLASAKPAVKHYASKYDHIDVETILNNPRMVKYYSACLLSQGPCPPEG
VEFKRILPE
ALHTNCHRCTEKQATVTLRAIKRLKKEYPKIWSQLSQMWDPDDVYVRKFESTFGNRNKI
PSVVVNNGWDL
GSSTTSNADEPRPDTTTHQIITSPNIMSFTTSKTSSTPITTSSTANPSTKTSTTTVGTTTKPPSR
PAPIP
GLLP

>ItypCSP5
MQCLGLFVVLVLGCSLVAAQSPYTSKYDNVDVDKILKNERVLTNYIKCLMEEGPCTPEGR
ELRKTLPDAL
ASGCSKCNEKQKDTTEKVIRHLMDKRTKDWDRLSKKYDPQGVYKQRFEKELSARKLA

>ItypCSP6
LIGLAPLVLRGSCPQCTEQEKKQIKKVLAYVQVNFPKEWNKMLQTYASG

>AchiGR17
MRKNPLVYFIGGCLLYFVSIGCQLFHIGSTVLIYYITPILLHYAKYVLIDIIYNITLSIEIKYKD
LNNML
LEINSVTTVTSKSATKLIKKVRILYLKINSVIDTFNSFFGWPLLLLVAHSFAQILTCLSLLATN
SYPDVL
SARIVISYYLIMTMGFLLRVISGCDNVLKASHNFVQNCFELRESFPLTSKPAEEIEKLQATFK
VQSNISA
AGFFEINRSLLLSIFSTATTYLIVVIQFRSVYSVQ

>AchiGR16
MGKKILVRDVKLIKEVTDRLYHLCRMHYKLCHLMRELNKTFAFQLLCSLAVSMGDVLFQ
SYYLYHLLFNN
VPNVTTTMILCPIAWLVDEMVEIFLLVRSCASSCESANTTPILLHEFRNELDNIEIESHIQMY
SLQLLHQ
KIRVSALGFFDIDYSLIYSIVGAVTTYLVIFIQFDQQTSDIKLYPVGNCSGP

>AchiGR15
MKLSDVDIILQQYKIATYFGFPLISEVTAPKLKRVCSKWYPFVIFFTYFVFTLNCLTKRISYY
IYLFPPM
QILNDVLEILSEILFLSVCLLWPTKRFQQWKTFWNNLKTLDKNLKKLNFEEKRNPFIDGTIT
SVCFVVIF
CGHIWEILDWYKIGKIGIIYYYMNFRIVMYYHLFATILICQITQMLKVRFDFLNQYLYKVG
KKKHILWDV
SQTEAYFTIRQLQEISKLYAMIHGLIKIFNNILGWHICFIIKSTILVILSSITFMVAILSQDIFKRG
QIS
TGLIIINVGYSSIYLVSAY

>AchiGR13
MFSCDWAIKEAGKLASTCYNYQAYFPTFSVEKQELLNLANQIVNNKPAFTAAGFFEVNCK
TLFALFGTTT
TYFIVILQFDQM

>AchiGR12
MQGKAAGGKFSKTKILLVVLAAAGDYVSSEANKTSAICYKVINKIPISSKYDVIKEELKLI
AQQTLCRNP
RLSAFGFFTVNFTMMGFIVTSITSYIIISIQFLI

>AchiGR11
MCPHTLDVVSCLYPAYIYSLLMGFAPFKIERKGPFVEIKHCKIYHVLSTLFYTLSICFLVWM
GYTKMGYY
HLQSPMQRFINFLYLLLVAGLMSVNVLFNKLHTTSLVTLWRKMCHIDTLIRNRGIKLNYRR
LMYSTSTFC
IVAATLHTCYIIFLVSIQNYKDPLFSGSVYFALNFAVTGFACVSCSFLAFFLLIGHMFEKILEY
MKHRVL
RSFLLDSDSKSKSIIDTADLHQELCELVRHANDVMSVQMLASFAVTFSVVTLQIYSMVNAF
NIKKVDYSF
VLSSLCCVIILLQEKVILSLVSRRCMGMIPKTKELLTELLNVHIDDKRICREVNAFNLQILHN
QFDITAG
NFFVIDPPIFLTAGSAITTYILLFIQADPFVDLYQSMKDSVGAI

>AchiGR9
MDLQDINISQENFEILQPIILVARFLGILPVRYDKLGDHFKLKRSLVYSIYSYVLTIFLTVATV
LGIVND
LEKDTNHSVRMVDQKARYVTSCDISIVIIIVFFSAVTIPQKMRKLWKLLHYLNQTDSIIPLTR
QDQFRQS
SLFFMATTFVVAVLLFTFDIVVWTNSTTKRMKDATSFFRNYTTFYVLYLIVMIHEIFYWHL
VLFIKIRIA
ALNRYLHSIGKEDRCKKIPVQAGGRQVVVGGALENIEVCKEGYKKKNFENMSLVERITVL
ATFQERISNA
VQVLNNDGAFGIHLITLSCLLHLIVTPYFLLVEIIKPDGNGMFTYLQAAWLLAHIGRLLIIVE
PCQLCLD
EHRRTSMLLCELLTKDFDENVRNSLIIFSMQLNYCKIKFSPCGFFKIDRSLITSVTAAVTTYL
VILFQFN
TN

>AchiGR8
MSCDGVEQSGKQIGKICFLHQEPLDKSSLKEELVLFTKITKELAPTFSAAGFFTINQSVLPT
LFSTVSTY
LIIIIQFNMTL

>AchiGR7
MMTNITIGIYGFTSEVLDHGITFSFKEMGLLVDSIYCMVLLYVFCDCSHQTSANIAEGVQLS
LMNIKLNS
VDVATTREVELFLKAIHLNPPKVSLQGYSVVNRELISSSVGTIAIYLIVLLQFKISLVNLRG

>AchiGR5
MSCDALEKSGKKIIKTCYMLHESVGNEHIKEELFLLATYAEQWKPALSAAGFYDLNQTTL
STLFEAIITY
LVIIIQFNLALV

>AchiGR3
MFSCDLAVKEAEKLVATCYKYQAYFPTFSEEKQELLNFAGQIINNRPVFTAAGFFEVNCRT
LFALLGTTT
TYFIVILQFNQT

>AchiGR2
MVYVIPQENVFNVNIKPKYVKESRENIVNSLKFFVICSQFLGILPVENITVPNKLRFRWKS
WKVLHTLFF
IGMTTVASTLCLLDWFYVGYVFNSLGITIFYSSALVTLILYVNLARSWPKLMTLWCRIDKI
MNNSYGYPK
SLNRRFRIVSATYSILALGNFLINTVYKSISIKESTRNEYNINKYYFKSFPQMFRFIPFSAVSA
TFCCIV
HIHTLLTWLINDLFIILISIALALRFKQISERLVRNQ

>AchiGR1
MVIRRVYFIYSFGFLIIRTICVCLFAAEIDCESRNPLDVLTSLPSKIYNIEIERFIVHIAKDPAHL
SGGG
FFIISRSILLRIAGAIVTYELVLIQMYTIK

>AchiGR14
MLNKIKVLVRVYDMLLQASNMTNRNFDIQLVPSLCNQLFLAAYILFYIYWMISEKPYSAL
CWAIWGILKI
LEVINFSAACHFASRELHVVGNSIHKVLIHNNNLAIREEVREFFYFIKSNAINLNFSYKSTTS
LAYVLFD
FS

>AchiGR10
MYLEVDDFIDALNNLCGWPVMLLSFNLVVDILTCLIEAVTWQGQDDVLSSDIALSIWFLV
QSVIWLTAII
FCCDSVVRETDELLTNCYRLEQTLPLLSKELEELDSLKKLIKNKRPKLTAAGFFEIRRSTLL
SLLSTTTT
YFIVALQFNSL

>AchiGR6
MSKQEEKLEAKSLFSWLPVPHIRSNALRRTITGLTLIAYFHIMVYYGLPVILLHALAIEIKCH
DEIINIA
INAKKIPNVLYFRSLNWYFVIIANYFFFGETFAEYLELFIDKYYVVKILFSYHRFFSFCFYFL
GMIWFLA
RLRRKFIRQQFSLLAWTHFLLIIIGFQSYMIVQNIFEGLIWLIIPVCVVILNDVFAYTFGLLLG
KTPLIS
LSPNKTLEGFVFGGISTLILGTFLSFIFCHIPFLICPVRFLETDNGIIMNTNCTPSYLFQPIYYDI
GNTG
ISVKVFPFLLHSLGFSLFASVIAPFGGFCASGFKRAFKVKDFSDLIPGHGGLTDRFDCQFLM
ATFVNVYI

WTFIRIPQVEKIYQKVFQLSEENQLKFYLLLNESLHTRGLMKD

>AchiGR4
MGIKSALSSVWNKMDYLDNFKLFDNVYGILDTSYMIYIAFGMTPYLRIKSPEGNFIYYNM
SWQKYGISAW
YSFLLLLAIAMTVIDELESHFNSIVNIYLIAQMIFSILTLNIFPARANILLKAYGNCHEAEKTL
KRLGFK
LDYTSIHKITLYGSLLMIFFASMNLTLYILWEKTIFDFIKFCFTYSFPCAVSVIPVYHFNYLGIL
VLQRL
RLVNSYLAEDFSSLTRKQVEYNLWAIAKVHSELCNAVNNVLYASAFSMVTFYFFTYLQFS
ALLLGAMNIA
HLTFDVETYIWNTIFIATTVGILVLATVLKYEATKTSYTLCEQTSTFFDDCIGTRRNTLLMQL
LHQKVDL
RIFGHFEVHLSRVLSDLQALVNLLMFATQFRN

>AglaGR64b for sugar taste
MALYQDKVVFRFNSVYDSVNMKTFYWYFRPVAIILKMFGVFPIQNVTTLDTSHLQFRFFS
CSFLYSLIIF
CLYASIICFISGFILYNPETEKYLTYVVCSITGRSIISYLFCFRKYRELPRLIRLLDTFDQKKNK
ILITY
STGKYTMLYQTVISKSAAIVGLALSSYHSSELIRSILSPTIKEMYISSLVSVFFSFLTTWQLYP
SFLYIY
FAVKIKCNFQEINKTLELRNITHDYFNTVIKYDSDTHQTLADVRTLHNMLSECVHELGKCY
GTYIAIDHL
CMIVFLVLSISVFLYESNHDIHLLFLTLGHVVILMNTIFTSESLKETGSEVINLLHGIPSTAISE
KCQLE
INVFLTQLVARPIQVSAAGYFILDKNQTLAIMSAVATYIIVVVQFVQTSPPCNLTNVTSIK

>AglaGR64e for sugar taste
MSKKFPTFLSKIDALDKSFVKKYKSSTNVRRNVVVFAIIANIIVTVEHSLQALTFMLTKDCD
NNSTGLEY
YFKKHFHYIFDFFPYNTAFAFILLLANWIGAYIWNYGDVFIIAMSMILTARFHEIREKICIYTS
AMPTSD
KKFSKPLSNDLQKTNFMFWKEIRRDYIMMSELCRKLNDLISDVILFDFASNLSFILFQLFAG
LSHKEKFA
KILYFYFSFGYVVLRICLLSIFGGWLYEAGRASLPILNAVPTEIYNIEITRLIHQMHYNTPCFT
GNNFFA
VTKGLTLSVAAAVITYELVLIQFSPDIHADDHSTCL

>AglaGR64a for sugar taste
YFTTTLLIQIFFIDLARQWPCLMKKWAEVDSSMRSYAYSSNLYKKLKITSIFVMLAAFGEH
GLFVANKIA
VIETNVTKAEMIEIYFRRNYNQIFKIIPYNLWFGIFVEIFEVYSVFTWNYNDLFIMLISITLSE
RYSQIG

NKIKLMSKYKIIDRSHWQSVREDYIRLNALCKLTNHLMTNIIVLSFMHNLFVVLIQFMYTL
HYETEIDCE
SRNPLDVLTSLPSKIYNIEIERFIVHIAKDPAHLSGGGFFIISRSLLLRIAGAIVSYELVLIQMY
TIK

>AglaGR43a for sugar taste
MDLQDINISQENFELLQPIILVARFLGILPVRYDKHGGHFKLKMSLVYSIYSYVLTIFLTVAT
VLGIVND
LEKDTNHSVRMVDQKARYVTSCDISIVIIIVFFSAVTIPQKMRKLWKLLHYLNQTDSIIPLTK
QSQFRQS
SLFFMATTFVVAVLLFTFDIVVWTNSTTKRMKDATSFFRNYTTFYVLYLIVVIHEIFYWHLV
LFIKIRIS
ALNRYLHSIGKEDRRKKIPVQTGGRQVVVGGALENIEVCKESYKKKNFENMSLVERITVL
ATFQERISIA
VQVLNNDGAFGIHLITLSCLLHLIVTPYFLLAEIIKPNGNVMFTYLQAAWLLAHIGRLLIIV
EPCQLCLD
EHRRTSMLLCELLTKDFDENVRNSLIIFSMQLNYCKIKFSPCGFFKIDRSLITSVTAAVTTYL
VILFQFN
TN

>AglaGR68a
MWGLKHNPKLDIFILLKQFKLGNILGITPVFFDTNCKKRIMISKVYPVVLTTVFMTSCVVT
VTERREHLY
RKQKITETIIDIGQCASQTIFVTVCLLGALTKSNNWKLLFIRIREIESKLNHTDFEVKKSLLL
NNINMII
YHAMFLLLHIYESYDWITTGRPSAIYTYIICRIGLYYQLFELIFICKITRMLRRRYDYLLGILN
ETIIGN
TLSCLTRTRLNWNLNDIFGLYKILYSIVQQFNAIFGWQIFFILQCTVLEVLNSINSVLSHAQT
GELDISA
LVTNLVCSMVYIVSTVVIVMSCDGVEQSGKQIGKICFLHQEPLDKSSLKEELVLFTKITKEL
APTFSAAG
FFTINQSVLPTLFSTVSTYLIIIIQFNMTL

>AglaGR24
MSYREDGAYGYNIKKMYNRNLNHLSIIGDSVTEPARKRSVYLEGAQPFYHTSLSKITKVAP
APGDYANGA
PNAFQKSALDSALFDTLKPVLTLMRVMGIFPITNQGQIFQVTPQWMIYSIVICFLILGYIGYI
RWDRVQM
VRSAEGRFEEAVIDYLFTVYLIPIVMNPIAWYEARKQAAVLTNLVAFEKMYRKISKKKIYV
FLGNRPLIT
AIGLPVLSVATMIVTHITMVHFRFLQVVPYCYINTITYLIGGAWYIYCDLIGKVATLIASDFQ
QALRNIG
PSARVADYRSLWMLLSKIIRDVGNAFGYTVTFLCLYLFLVITLTIYGLMSQIQEGLGVKDIG
LAITAGYA

IALLFFICDEAHYASNCVRVQFQKKLLLVELNWMNDDAQQELIATMVTYLVVLLQFQISIP
EDSSGGETT
ASGVTEKTTTSN

>AglaGR2a
MFSCDWAIKEAGKLASTCYKYQAYFPTFSVEKQELLNLANQIVNNKPAFTAAGFFEVNCK
TLFALFGTTT
TYFIVIVQFEQM

>AglaGR28b
MTLMYVRLRFKKVNEIITIRIRKRSILKCTSGHDVKLIKEVTDRLYHLCRMHYKLCHLMRE
LNKTFAFQL
LCSLAVSMGDVLFQSYYLYHLLFNSVPNVTTTMVLCPIAWLVDEMVEIFLLVRSCASTCES
ANTTPILLH
EFRNELDNIEIESHIQMYSLQLLHQKIRVSALGFFYIDYSLIYSVVGAVTTYLVIFIQFDQQTS
DIKLYP
VGNCSGP

>AglaGR64f for sugar taste
MVYVIPQENVFNVNIKPKYVKESRENILNSLKFFVICSQFLGILPVENITVPNKLRFRWKSW
KVLYTLLF
IGMTSFASTLCLLDWFYAGYVFNSLGITIFYSSALVTLILYVDLARSWPKLMTLWCRIDKIM
NNSYGYPK
SLNRRFYIVSITYSILALGHFLINTVYKSLSIKESTGNEYNFNKYYFKSFPQMFRFIPFSAVSA
TFCFIV
HIHGFLTWLTNDLFIILISIALALRFKQISERLVRNQRTFQNKSLHFWKEIREDYDRLSLLCK
ELDKHLS
YMILLSFSMNIFFLLVKLYNSLEGFADVSGQVYFVYSFMYLIIKIVSVSLYAAWINDESVGP
ASILNSVP
ASSYNIEIRRLLTQISFDNVALTGCRMFKVTRGIILSIAGAVVTYELVLIQFNAATGLNTTE

>AglaGR39a
MALYFVDAPLEKNASKNKFNMYKRAFLRTDQYDVKVIIGLLRCYKFFFVTPFNFEDESNR
KLGKYLSIIL
SLLCVVYMYFSITDSYGLLKDSPITRFLLADLWCIMNMLYLMSCFLNANAFKEETWKTAL
RSVDQAEYML
RKMSFQSPKKDLILLLQVVLIFVPYVVLTSFQVFLWISGGNMIALYSFVGSYVAYLYMCLF
LFFVLRLTG
ILKARYEFVEDNLKNIAGSKMDDQEKVRKLTEIVRLYKTFMFLMDSINGIFGPHLFFFISVT
ISYMLCAI
SYNMDIDAENETAKDSSILSITVTVVYTLSLIVLIISCDGLEASGKKVIKTCYIMHESTESRL
VKDHLLQ
MAQYAEQWRPTLSAAGFYNVNQSTLSAIFEAIITYLVIIIQFNLALA

>AmelGR10
MVLVGDTNLIFNAGLILGCTALCASKDNVKARRLRDIIFIVFSMTLYLVSLVVIYIYVFSYE
DSDLKSTL
IIIIRVFLIYLCLFTDASVTTLWNWKIRSVLSQLRNFDRATKFRDFSKGNKLRIICHVTMFVS
FSYWAIV
GYFTYRIEAKVPIFHGIIYFIMDASMNTQILIFVCILFLIEERFRHLCSMIELSKADKIIEAHRS
IRHST
LQQIWWLHCSLANATEIINSVYAIQLLFWISSMSFNLMSRIYSLKVFKLSDYGKIRESMLVT
DCAWNLVL
ITTVCHMTAHQANRVGELIFSPYSSVSLKRVHLQENIEAAAYFQLRKVHLFTVAGLIRVDLP
LLLSVSIF
SALTTYLVILT

>AmelGR64f for sugar taste
MHSEDQIQLMMLKTKDGLGEIPKGKGRGSNLKIWSSVMYHKDDNNIEDISANQENDLST
KRPRAERNYFR
NSEALENFHCAIGPVLKAAQIFGMFPVSGIGSSSLSKLQFKIFSLLTMYSGFIALMISFMTIV
SMIHMLK
TFNASTFQIRGGLGAATVGAVFYGNSLVGSILFFSLSSRWVSLQYEWRAMERYIDSNSTEP
TRLRWKFFI
ISTMVLVLSLIEHVLSIFNNIDGYEWNESNSTFHNFLEIYTLRSHSFIFDTLNYNFVYGLYVF
VVSKLAT
FTWNFTDLFIMLVATGLAERYKSLNKKLAVTMTKCQAAFNWRELREDYAILSCIVKKVDD
HISPIILLSF
ANNVYFICLQLLNGLSISDKNSVLSEAYFFGSFAFLICRTCAVTLLTARIHDQSKQALPYLYN
CSTSSYS
VEVQRLQCQLATDDIALTGLRFFSITRNFMLAVAGAIITYEVVLLQFNGK

>AmelGR43a for sugar taste
MEVKRVEEKRKILFNNELCQAIFPIYYLGKFCGLVPVRFFVHTSEGCQARLNIIDLIYSLCV
LVLLLSAE
IWGLWRDLKDGWEYSTRLKSRTAVIATCSDVLGVMSLTVVCIVGSPFRWKYLQLVINKLIE
VDEKIGVSS
AKVARRFTIVLTICSLSYLWFNSIIDFYTWNRKTKVDNKAMTGKGPINYAPLYFMYTVIIST
EIQYTVST
YNIGQRFIRLNTSLKDLFNANSNNNDNAIDYFRKCPETAHDMDDKKIWNLKPKRQIVLGS
YRLSRKLDEN
KMYVNNISELIMVHSSLCDAVSLINSTFGVVILAVTVTCLLHLVITPYFLILQAGERHEWIFL
IVQGGWC
IFHITRMLIIVQPSYSAIAEAKKTAVLVSQLLSCTFEANIRRELEIFSLQLLHRPLEFSACGLFS
LDRNL
ITSIAGVVTTYLVILIQFQNADDTKDDFDIIRNATQILKNASPLQNFTGLKTIV

>AmelGR28a

MVDKLYWSRNMHLAIILFLFFFKFFGLATFSLNNRRNSKIWSSKNVALFVNSKLGILYNLF
VSFLIITLN
FSLMPLIFYAEYAFRTNITIILETFQALLGSSVIILTLLSYCIFQSVIKEIGNYLIRIELILHRLQQ
PIN
QKYIFNLLFFVCLFKFIIFVALLFTEIIYFKPEPITLLGNLIPTIFAGLLFVQYFFVITLINEMFIK
LNC
IMQNFYQNRLDDFNSNILYQNRRIFLNCSRIHLLLQIRNIHDHLCNISREISQFYAFPTLTGLC
FIFFTS
LYIIYFFLAIFLKNINVDLILVINGILWIILLLCPFGLLTSKITKIVNEIEKTGCIVHILLNCVIDQ
KVK
KELKQFSFQLLHQKIIFSTNGYFTLDNKFFQSI

>AmelGR28b
MRIYIFISSYIIFFGHKKMVDKSYWSRNMDRLTLLFFFFFKFAGLATFSLNNRANLNKKNSE
NTMFFISS
KLGILYNLFGSCLIIALSFYSIPITMYADYVHKTIVTITIEIFLIILGCFVMTSTLLFYCIFESVII
RIG
NYLINIENVLRHLQQPLNRKHIFNVLFFICLFMLILLIILLITEIKNFNPNPLVLMASIIPLIFVG
LLFI
QYFFVLNLIYAIFVKLNCIIQSFCRTRYDDINFKILNQTRCVFMSYSKIQLFIQIRDIYDHLCD
ISREVS
DFYSFSILTALSFIFLVILYNFYYLFLHFVNDKLNFLLITNAIIWIMLPLSLLALLTSKVTNVIN
EIEKT
GCIIHVLLNCTIDRETKIELEQFSLQLLHQKVKFTANGYFTLDNTLFQSMINTVTTYMVILF
QFQMEISN
ENDKFCNCTQCR

>AmelGR32a
MYEPKTLKETIKPLMVVNFVFGMGLTGIEAKKPSKKLEYVYTLCNLVLFYFINKLTLPYY
DKYYVISTFE
LSRFIFQWMFHANIWTITLLIIITRIKAQQLRTIVSLVELSDQKMENIGLSPKHRCLMMYQIK
RYIFLGI
YTLIFVVLIYHCHYESTTPALIKLCLSILPNIPFIVFGVSTISFCFWVTCLKLKFRQLNELLRS
MRIMES
PIHKRVLEMTNNFENNRFALYRNEHVRRNTNTIRAVKQIHLEIIKIVSFLNQTFGIQILIQMT
VSVVFTT
NLLHLLYRVIWLNFTLPELLQELISVIFWILIYGSQILYVNHVCASTNSEAVNIGNIICEFYEP
FATKEF
QAEIRDFTLQLIQNPVVFTAYGFFNLDHSFIQGVIGTITTYLVVMIQVGDLSNSDKSILS

>BmorGR9
MPPSPDLRADEPKTPCLVGGAHAFILKISSFCGLAPLRFEPRSQEYAVTISKGKCFYSYILVT
FLVICTI
YGLVAEIGVGVEKSVRMSSRMSQVVSACDILVVAVTAGVGVYGAPARMRTMLSYMENIV

AVDRELGRHHS
AATERKLCALLLLILLSFTILLVDDFCFYAMQAGKTGRQWEIVTNYAGFYFLWYIVMVLEL
QFAFTALSL
RARLKLFNEALNVTASQVCKPVKKPKNSQLSVYATSVRPVSCKRENVIVETIRVRDKDDA
FVMMKTADGV
PCLQVPPCEAVGRLSRMRCTLCEVTRHIADGYGLPLVIILMSTLLHLIVTPYFLIMEIIVSTH
RLHFLVL
QFLWCTTHLIRMLVVVEPCHYTIREGKRTEDILCRLMTLAPHGGVLSSRLEVLSRLLMLQN
ISYSPLGMC
TLDRPLMVTVLGAVTTYLVILIQFQRYDS

>BmorGR67
MRERKKKFNKLLNTRNYNNIVEALLPSDSIRKISGVSVVYLAVNSENRIVTKFSFIGTIFFLF
WYILYFY
CTYKAHSEDQTILRTIYNTKLKRYGDDFERIASIIYVTYSMWKVPFRMSGNQVFIQRIVDID
SAIENMGE
AVDYNKNAKTALVISIAQLGDFLVRMFCIWLSLENLSVIVPTEKLYQVVYTDALSFVITSH
YCFSLIVLR
GRYKYINKVLSEIKTRSAWEYKVFVRNKVAPDLEKVQRLQDRIVCEKIKACARIYSMLYK
ATEAINRMYG
TALVLTMLLYLVFIILYMFYFMEATASGLLYDIKKYVDFLICVFWQMSHALSIIYANVYFSES
ITREVCK
F

>BmorGR60
MLTPRSDLCNEKLSPSFPSGKTTAADKDDTEARCQVDSSLERLLLPFNLVQHVSFIPMYSIR
RGLVSPDG
PLAYLYSLLGFCLFTSVSVYRNAIMHGTRLSSLHLFTLYSDLVSFVINYSLSLICNVVNSKS
NVEFVCRL
QRLQTVLRRNQREQEQFARSNWAHLAVVTALYLAVVGLLNVVVLKQSLPDTLYLLLLFCI
DVNVLYATRM
LALLRCYLQLWTRKINEKAFNPVHHNMFTAYLDILQEYEVYTTLFKKIITYYVLETFLHGL
LYVQVAIQI
CKSIRRSGRFSEQLMMIVSIFTWTIKNMIIMTLHNVECEKFYLAVEQAVAACQTQRASTTR
CREEKRLYK
NVCRVSRAAFSRERGWGLLAAGAALTLRFMDLATTYVTVLLQFAFVSRT

>BmorGR8
MAPRSVRSMVGTSKKDMLKGGFYETVRIPLYIYRLIGILPISGLWHRSSKYNRFSLKSFYTII
YAPTIVM
QTFLLLVHIYDLFAFFFGHQRLGRLIYHMNFYTITILIFMGSRKWKNVIKEIETIELTLPRLR
NSKKALA
LTKSFVFAFFVFSLAEVVLILQFTLRLTKQRHVLPGDSGLYLRSYFVYIFPYLYDHFPFSYV
MGFIVQII

KVQGIITLNMVNCSVVILSIYLTNRLKHYNRIVFAKGSKTNNTRLKWVELNLLYTRISNLV
KIIDKNLNP
FVFISFTANLSYICAQLFYILNKLTSSRTVKITSFLEDKRSDWETVLYISISFALVVLKVLLVSI
TAAEV
HTTSREPLRLLYTLPTAEYTIETQRLMTQVYYSNLSLSGLNFFHITRGMLLGMVATLLTYEI
VLLQI

>BmorGR45
MKSPEYLSKDILDEDFVRVFSFPFLVQMALGSCRVHLKARFITVPTLGQKLYTVMCIIICSL
MYFNMTKL
YLPLYYEHSIVYYIFVTVTGLDQLSFFANLIHLRFLNGETNTAFYIMMQRIDRNMKIDHNNI
FNKTVTLA
NILTITLIILHYVGLVISTIILKEYSLLSLFGLLYGQLMLMVEMALCSNLIIFFFMRVRFVNAII
KNHVH
PENQNQPPKLVRYFITNRITRYLAAQTHDFIVNDTDVYLKQIFEGFSMFIDIYRFQVCPLCIK
LVVLTLL
NFEFCLVAIQRNVLGPNHIGNYYIIVNSVMGFFTALYVSGRCELFFREIRETKRLSVAVLLQ
YQEGPLRE
KATRMLKIIEESTPQFSIYDMWQMDGYTFVKICSLVTNLIVTLLQFAYL

>BmorGR31
MYLRSKKSRFKLFSFERMIKILLMICGHYVQTDSSNVVSSIHRIFSIVITICLCPYFQFNPFFF
HVIESV
LYSILSQFTQYGFFFRYCSTIKTFDLLSGFKQIPLYTKRVCFFLLITLLVRLIIVLIHFSAHQTK
LKTFC
AFLIILSANTGHILMTIMFSILNTRMTLIQKLFANNPIPVNIVGKNQNASHIKRVRKGLICYN
NLLDTLK
VAEKEIQFTLTVTYLCHVPKIICYVYFVITVIYKSKFSGYNLIPMLDMILACMAVTAPALFAE
LTKNTVD
KIKKILGSQLLRCSDESLRYELEITLEYVIQRPFSFSIWRAVSLDASLPVAMTSLCITYVIVIL
QLTQLR
P

>BmorGR64a for sugar taste
MGLPVGDEDRDESGVEDWEEEGEEDVASLSSPSDLEEGPSRGRQHDRKPQPTTVHHINDE
YAMNIDRDSL
VLPIGMPDFKLRCETVYLDGLKILMYKREFLPTMSRIFSMTRYFGVSTCKPSIAFGWTVILL
LMLLAIEV
GAIWKIVRLLGGWAVHSTDSRGFTARLSGCIFYGNALLSLILSIKFVSSWEQLSERWSRTET
DPGLRLPS
DSRIKRRTVLVSAFVMTCACVEHMLSMMSATGFDCPPEEYTERYILSSHGFLVQNDEYNL
WLAIPIFIMS
KLATALWNFQDLIIILISMGFTSRYNRLNTYVHRVVMLERNLKEGAQVSSENYMRFQIWR

RIRQAYVRQA
ALVRLVDDQLGALVLLSNVNNLYFICLQLFLGINSKDRGSFINRLYYFISLGWLMFRACGV
VLAAADVYI
HSKKALISLYLCPELAYNLEIKRLKYQLKNDEVALTGMGLFSLNRELLLEVAGTIVTYELVL
LQFSNED

>BmorGR10
MTMSIKPRLQCMVPPSLALALRVSRLAGIAPLKFVAKQSNIMIRLSTSLCVYSYLLVTALN
VCTLIAVMI
DFSVPVKLSIRMQTETKRFVWIADVVIVGILSGVGVYTAPIQMRRLIAYLHRIHKINSDLGT
YSSSLTDK
MLHRLTIGMLLITSVIIVTDFTFVMYLADLNHRQLLIAIMYWCYYCSYFIAHLLEMQFVLI
AALALSSLK
LVNNGLRTLLHQSGIESLTEIPNSNEQHTANAILPQPPKKSVNNSIDTLAFVVTKRSVRFPTA
GWMDQRT
IRRLALSYGSICEVVRQIDNNNGIIVLLLLASFLLHLVVTPYYLIISFVTESPHTGFEKVLNPI
LQTVWC
LYHTFGLVMIIEPCHRTHEEMETTRELVSRVMCSADPRDPISIELEMFFRQLVLNKASYAPL
KVCTLTRS
LVATILGSITTYLIVIVQLEIKNMQ

>BmorGR68
MRFGLKAGAAVVTILRPYNLCLKNIFKPFYVMLSLLGLFPYSIRFLGGKQFLIKPKSIYTNA
VCALSLML
SMTLFLIFHIDHIIYKSTEDNSLTEGFMTQVNYIIEMLNLEIFCVVYYFSSFLNRNKFVKVLN
TVAVWSD
RISISGIKTLSFLRLKIHFSIGILMFLLISQVCVNFTRVDSLWKKVLVMFTFNIPQMIQFTAILF
YYILV
NMVITLLVIIQENISISTRDTKTSSFIRVEHRMPLSLKQLELIYIKAFELKRDINKAFEAPILLT
TMQCF
HSIVSESHIIYHGAVMEPHMVLHSIMNCSVWILYQLFKLYILASTGHLLQEKIQHFSNLIHF
HGKGLTVY
GLFPLDGTLMFKVVASAAMYLIILVQFDKRN

>BmorGR17
MGFSLGTTALSMFFFEKPVVFTIIQITMIIVKPAKYKLSDPFRPKDTSKLSESIIMYFKLFHIF
LGIDLG
GFRYQNRQVKYAVRLISLIQPLAIYGLCIYALLKIIANTEFLWYTISFTEYVAMSVAITLFSNE
MTYCNF
MINLKFIDTKLKIGDESFRIGVKLISSTILIGVTRCFTTTTYCLLGFCAKPTAAQILFQIPWLTI
DLMLL
QYMFIFYACYCRLVKILRILKKRNTDIEEMRRIYKTLVDVLDRARAPFDLAYLLGLLFSIPD

VLYSIYES
IIKVGEINTAKALSMSIIYITNIQSLALMFAPALTAGFLPSLTMKMRIILHDKLLEEQDKKTYR
HIVLFI
KYIETCPLKLKACQIIPLDFSFPIIILNIVVTYLIVAIQLTHFL

>BmorGR29
MYLRSKKSRFKLFSFERMIKILLMICGHYVQTDSSNVVSSIHRLFSIAITICLCPNFEFNPFYF
HVIESV
LYSILSQFTQYGFFFRFCSTIKTFDLLSGFKQIPLYTKRVCFFLLITLFMRLFTVLIHFLAYQS
KFVTFC
AFIIMLSANTGHILMTIMFSTLHTRMKSIQKLFANNPIPVNIVGKNENASHIKRVRKGLICY
NNLLDTLK
DAEKEIQFTLTVTCLCHVPKIICYVYFVITVIYKSKFSGYNLVPLFDMILACMAVTAPAVFAE
LTKNTVD
KIKKILGSQLLRCSDESLRYELEITLEYVIQRPFSFSIWRAVSLDASLPVAMTSLCITYVIVIL
QLTQLR
P

>BmorGR28
MAYVMGLIEYLLFVLLSLFTGDEYFYKFHNSIKSIDVLMGYKRGKIIDSNAIIFLLSVITIMR
IVIIYCR
STVLAFRFTIIGVYLAIFSLRISYMLITVIFFAMYHRMKFLRKKFEIITIPVTIIGKQKVASKIR
LIRKY
LINYHHLLDCLRDINGGLQYFLAIMIACNLPKYIFFAYSAIKIQVLEHITIHSAVQNVEMFEG
FLFVVVP
AIFAELTTAEVERIIDVINRQLLRCTDEHMELELKVALEFIRRRPFDYVIWRTVPLNASLPIAI
ISLCIT
YVVIVIQLTQFHDNF

>CbowGR1
MFAGMMAFCTIFVLFADFTEGAPALVKARQFLSDIPGFIPVYIRAGETPLEDINPDLAEAFN
YYAQKHGR
LAFGRSIDEKSDNTDFHSGFPEGDKLSDIDTVSLDDESIQEGDNNVTVTVNPKESQHIQKIP
KA

>CbowGR4
MIMGLVQLLGVILSVSLISCLGKRLINFISLFGSGICCLIVGSYAYINDINYLDSPIPIVSNTSN
ATMEN
TATDSLQWISVTFLVISAFLTYFGIKVLPWILIGEIYHNKIRATASGLSAGTGYIIGFLANKFF
LDMVNN
FTLPGVFWFYGSVGVFGTVLLYFVLPETEGKSLFEITEHFAGGDKLRRSVRRKKLSGNINN
AFEPEDMNG
NGMNES

>CbowGR8
MINMNCALWYINCRAIGNASHALAETFQNDIDHYCTAYIIAHYRVLWLELSELLQKIGNAY
SRTYATYSL
FMITNITVAVYGFTSEIVDHGITFSFKEMGLLVDAIYCSVLLYVFCDCS

>CbowGR2
MTSWCRMDKIMNESYGYPESLDKRLQLFPAIFLVLALTEYFLSVWSRYIRLTLTLGEKYDY
EKYYSDTFP
QIFKFIPMNVVTAAYCSIITVHATLMWGLMDVFIIIMSIALALRFKQVSRRIAKHVKRATSE
TFWAEIRE
DYHRLSILCKELDDHISYIILLSYTLNIFFILKQLYESLEIRSGTVGKVYYLFSFLYLLVKVGS
VSLYGA
WINDESKEPADMLNSVSSACFNVEIKRLLAQINFDNVALTGCRMFKLTRGIILSIAGAVVTY
ELVLIQFN
SATIDNY

>CbowGR3
MNILFVVTQLYKGLTTRRTTVDQCYFVFSFGMIILRLVGVTLCGSTVHTQSKKPLAHLVSL
KSEFYNVEV
KRLILQIHCHDDSLSGNQFFTITRPLIFEMATVIITYVLFMLQASITIYS

>CbowGR5
GSLSDTANSSKKIICNILQNSLNSSIKQELWIYLSLATTNKIQCSACSFFKINKTLITSAVSVG
TTYLVI
LAQFNDK

>CbowGR6
MLIGSALTFRLKQVSNRLENISRMKVNDPTQWRSVRKDYNRLTELCQTVNKRLSAIVIVC
FLTNLYFLLI
QLFLSLGKMDNFVEKIYFYMSFLLIIMRIVGVCILGGEVYEEWKNLSFFLNCVVTSAYNEE
VERMTCHVV
SWELSLSGKNYFKISRGLILKIAGAIVTYELVLIQFYKNVIEQ

>CbowGR7
MTLLFIICNEAHSASHKMGSEFRERLLSVNLGAVDNRTRQEVNMFLTAIDKNPPIMNLNGY
ANINRKLIS
STVTSIATYLVMLMQFRLSLMRNAAIAARKAAASATTNATLST

>CbowGR9
MSCDGVERCADRIVTTCYMNLDILEKSPIREEILSFTEYVEQLTPVFSAVGFFQVNQKVLSS
LFSAVISY
FIIIIQFNSGL

>CbowGR10

MSCDRVEKSAQKLLATSHCLQASVQDDNIRTELFHLWEFIEELLPKFSASGFFRINQHIIPAF
LSAMTSY
IIIIIQFEM

>DmelGR23a
MFPPTRVQASSRVVLKIFHFILVAFSLRSRRLSRLVLWLQFLGWLTWFISMWTQSVIYAQTI
DCTLDCSL
RHILTFFQTVSHAFIVVTSFLDGFRIKQDQLDEPIAFEDSDPWLAFTVLAMLVPTLGVEYLV
CSNAPEYA
FRIRIYHLKTLPSFLALQVQIISFILEVMKVNIRVRQTKLQLLILARELSCRWPQRKQKPQFS
DQQAHRV
KDLKRRYNDLHYLFVRINGYFGGSLLTIIIVHFAIFVSNSYWLFVDIRTRPWRIYAILLNLGFI
FNVALQ
MAAACWHCQQSYNLGRQIGCLISKLVKPQGSKLYNDLVSEFSLQTLHQRFVVTAKDFFSL
NLHLLSSMFA
AVVTYLVILIQFMFAERSSTRGSG

>DmelGR68a
MKIYQDIYPISKPSQIFAILPFYSGDVDDGFRFGGLGRWYGRLVALIILIGSLTLGEDVLFASK
EYRLVA
SAQGDTEEINRTIETLLCIISYTMVVLSSVQNASRHFRTLHDIAKIDEYLLANGFRETYSCR
NLTILVTS
AAGGVLAVAFYYIHYRSGIGAKRQIILLLIYFLQLLYSTLLALYLRTLMMNLAQRIGFLNQK
LDTFNLQD
CGHMENWRELSNLIEVLCKFRYITENINCVAGVSLLFYFGFSFYTVTNQSYLAFATLTAGSL
SSKTEVAD
TIGLSCIWVLAETITMIVICSACDGLASEVNGTAQILARIYGKSKQFQNLIDKFLTKSIKQDL
QFTAYGF
FSIDNSTLFKIFSAVTTYLVILIQFKQLEDSKVEDISQA

>DmelGR8a
MSGHLGRVLQFHLRLYQVLGFHGLPLPGDGNPARTRRRLMAWSLFLLISLSALVLACLFS
GEEFLYRGDM
FGCANDALKYVFAELGVLAIYLETLSSQRHLANFWWLHFKLGGQKTGLVSLRSEFQQFC
RYLIFLYAMMA
AEVAIHLGLWQFQALTQHMLLFWSTYEPLVWLTYLRNLQFVLHLELLREQLTGLEREMGL
LAEYSRFASE
TGRSFPGFESFLRRRLVQKQRIYSHVYDMLKCFQGAFNFSILAVLLTINIRIAVDCYFMYYSI
YNNVINN
DYYLIVPALLEIPAFIYASQSCMVVVPRIAHQLHNIVTDSGCCSCPDLSLQIQNFSLQLLHQP
IRIDCLG
LTILDCSLLTRMACSVGTYMIYSIQFIPKFSNTYM

>DmelGR10a

MTSPDERKSFWERHEFKFYRYGHVYALIYGQVVIDYVPQRALKRGVKVLLIAYGHLFSM
LLIVVLPGYFC
YHFRTLTDTLDRRLQLLFYVSFTNTAIKYATVIVTYVANTVHFEAINQRCTMQRTHLEFEFK
NAPQEPKR
PFEFFMYFKFCLINLMMMIQVCGIFAQYGEVGKGSVSQVRVHFAIYAFVLWNYTENMAD
YCYFINGSVLK
YYRQFNLQLGSLRDEMDGLRPGGMLLHHCCELSDRLEELRRRCREIHDLQRESFRMHQF
QLIGLMLSTLI
NNLTNFYTLFHMLAKQSLEEVSYPVVVGSVYATGFYIDTYIVALINEHIKLELEAVALTMRR
FAEPREMD
ERLTREIEHLSLELLNYQPPMLCGLLHLDRRLVYLIAVTAFSYFITLVQFDLYLRKKS

>DmelGR93a for bitter taste
MFSSSSAMTGKRAESWSRLLLLWLYRCARGLLVLSSSLDRDKLQLKATKQGSRNRFLHIL
WRCIVVMIYA
GLWPMLTSAVIGKRLESYADVLALAQSMSVSILAVISFVIQARGENQFREVLNRYLALYQRI
CLTTRLRH
LFPTKFVVFFLLKLFFTLCGCFHEIIPLFENSHFDDISQMVGTGFGIYMWLGTLCVLDACFL
GFLVSGIL
YEHMANNIIAMLKRMEPIESQDERYRMTKYRRMQLLCDFADELDECAAIYSELYHVTNSF
RRILQWQILF
YIYLNFINICLMLYQYILHFLNDDEVVFVSIVMAFVKLANLVLLMMCADYTVRQSEVPKK
LPLDIVCSDM
DERWDKSVETFLGQLQTQRLEIKVLGFFHLNNEFILLILSAIISYLFILIQFGITGGFEASEDI
KNRFD

>DmelGR64d for sugar taste
MERSVQENTLHYTIGHVLIIARIFGVLPLAGINPNGKPENVRFRWFSPYILFFVVAFTFVIAD
FMLSTKI
VLNDGLQLYTMGSLSFSVICIFCFGSFIKLSRRWPHIIRETALCERIFLKPCYANQEGLNFTRF
LRRWAL
ILLVAALCEHLTYVGSAAWSNYVQIRDCNLKVGFVENYFLRERQELFSVFEYRAWMVFFI
EWNTMAMTFV
WNFGDIFLFLMCRGLKIRFQQLHWRIRQNLGKPMAKEFWQEIRSDFLDLDSLLKLYDKEL
SGLILVCCAH
NMYFICVQVYHSFQVKGAFMDELYFWFCLLYVISRLMNMMLAASSIPQEIKDISNTLYEV
RSSPWCDELG
RLSEMLRNETFALSGMGYFYVTRRLIFAMAGALMGYELVLFRQMQGAVVQKSICSRGPG
SSMSIFFS

>DmelGR64b for sugar taste
MPQGETFHRAVSNVLFISQIYGLLPVSNVRALDVADIRFRWCSPRILYSLLIGILNLSEFGAVI
NYVIKV
TINFHTSSTLSLYIVCLLEHLFFWRLAIQWPRIMRTWHGVEQLFLRVPYRFYGEYRIKRRIYI

VFTIVMS
SALVEHCLLLGNSFHLSNMERTQCKINVTYFESIYKWERPHLYMILPYHFWMLPILEWVN
QTIAYPRSFT
DCFIMCIGIGLAARFHQLYRRIAAVHRKVMPAVFWTEVREHYLALKRLVHLLDAAIAPLVL
LAFGNNMSF
ICFQLFNSFKNIGVDFLVMLAFWYSLGFAVVRTLLTIFVASSINDYERKIVTALRDVPSRAW
SIEVQRFS
EQLGNDTTALSGSGFFYLTRSLVLAMGTTIITYELMISDVINQGSIRQKTQYCREY

>DmelGR64c for sugar taste
MQQSGQKGTRNTLQHAIGPVLVIAQFFGVLPVAGVWPSCRPERVRFRWISLSLLAALILFV
FSIVDCALS
SKVVFDHGLKIYTIGSLSFSVICIFCFGVFLLLSRRWPYIIRRTAECEQIFLEPEYDCSYGRGY
SSRLRL
WGVCMLVAALCEHSTYVGSALYNNHLAIVECKLDANFWQNYFQRERQQLFLIMHFTAW
WIPFIEWTTLSM
TFVWNFVDIFLILICRGMQMRFQQMHWRIRQHVRQQMPNEFWQRIRCDLLDLSDLLGIY
DKELSGLIVLS
CAHNMYFVCVQIYHSFQSKGNYADELYFWFCLSYVIIRVLNMMFAASSIPQEAKEISYTLY
EIPTEFWCV
ELRRLNEIFLSDHFALSGKGYFLLTRRLIFAMAATLMVYELVLINQMAGSEVQKSFCEGGV
GSSKSIFS

>DmelGR63a
MRPSGEKVVKGHGQGNSGHSLSGMANYYRRKKGDAVFLNAKPLNSANAQAYLYGVRK
YSIGLAERLDADY
EAPPLDRKKSSDSTASNNPEFKPSVFYRNIDPINWFLRIIGVLPIVRHGPARAKFEMNSASFI
YSVVFFV
LLACYVGYVANNRIHIVRSLSGPFEEAVIAYLFLVNILPIMIIPILWYEARKIAKLFNDWDDF
EVLYYQI
SGHSLPLKLRQKAVYIAIVLPILSVLSVVITHVTMSDLNINQVVPYCILDNLTAMLGAWWF
LICEAMSIT
AHLLAERFQKALKHIGPAAMVADYRVLWLRLSKLTRDTGNALCYTFVFMSLYLFFIITLSI
YGLMSQLSE
GFGIKDIGLTITALWNIGLLFYICDEAHYASVNVRTNFQKKLLMVELNWMNSDAQTEINM
FLRATEMNPS
TINCGGFFDVNRTLFKGLLTTMVTYLVVLLQFQISIPTDKGDSEGANNITVVDFVMDSLDN
DMSLMGAST
LSTTTVGTTLPPPIMKLKGRKG

>DmelGR22e for bitter taste
MFRPSGSGYRQKWTGLTLKGALYGSWILGVFPFAYDSWTRTLRRSKWLIAYGFVLNAAFI
LLVVTNDTES
ETPLRMEVFHRNALAEQINGIHDIQSLSMVSIMLLRSFWKSGDIERTLNELEDLQHRYFRN

YSLEECISF
DRFVLYKGFSVVLELVSMLVLELGMSPNYSAQFFIGLGSLCLMLLAVLLGASHFHLAVVF
VYRYVWIVNR
ELLKLVNKMAIGETVESERMDLLLYLYHRLLDLGQRLASIYDYQMVMVMVSFLIANVLGI
YFFIIYSISL
NKSLDFKILVFVQALVINMLDFWLNVEICELAERTGRQTSTILKLFNDIENIDEKLERSITDF
ALFCSHR
RLRFHHCGLFYVNYEMGFRMAITSFLYLLFLIQFDYWNL

>DmelGR61a for sugar taste
MSRTSDDIRKHLKVRRQKQRAILAMRWRCAQGGLEFEQLDTFYGAIRPYLCVAQFFGIMP
LSNIRSRDPQ
DVKFKVRSIGLAVTGLFLLLGGMKTLVGANILFTEGLNAKNIVGLVFLIVGMVNWLNFVG
FARSWSHIML
PWSSVDILMLFPPYKRGKRSLRSKVNVLALSVVVLAVGDHMLYYASGYCSYSMHILQCH
TNHSRITFGLY
LEKEFSDIMFIMPFNIFSMCYGFWLNGAFTFLWNFMDIFIVMTSIGLAQRFQQFAARVGAL
EGRHVPEAL
WYDIRRDHIRLCELASLVEASMSNIVFVSCANNVYVICNQALAIFTKLRHPINYVYFWYSL
IFLLARTSL
VFMTASKIHDASLLPLRSLYLVPSDGWTQEVQRFADQLTSEFVGLSGYRLFCLTRKSLFGM
LATLVTYEL
MLLQIDAKSHKGLRCA

>DmelGR32a
MSPNTWVIEMPTQKTRSHPYPRRISPYRPPVLNRDAFSRDAPPMPARNHDHPVFEDIRTILS
VLKASGLM
PIYEQVSDYEVGPPTKTNEFYSFFVRGVVHALTIFNVYSLFTPISAQLFFSYRETDNVNQWI
ELLLCILT
YTLTVFVCAHNTTSMLRIMNEILQLDEEVRRQFGANLSQNFGFLVKFLVGITACQAYIIVLK
IYAVQGEI
TPTSYILLAFYGIQNGLTATYIVFASALLRIVYIRFHFINQLLNGYTYGQQHRRKEGGARAR
RQRGDVNP
NVNPALMEHFPEDSLFIYRMHNKLLRIYKGINDCCNLILVSFLGYSFYTVTTNCYNLFVQIT
GKGMVSPN
ILQWCFAWLCLHVSLLALLSRSCGLTTTEANATSQILARVYAKSKEYQNIIDKFLTKSIKQE
VQFTAYGF
FAIDNSTLFKIFSAVTTYLVILIQFKQLEDSKVEDPVPEQT

>DmelGR21a
MTFLDRTMSFWAVSRGLTPPSKVVPMLNPNQRQFLEDEVRYREKLKLMARGDAMEEVY
VRKQETVDDPLE
LDKHDSFYQTTKSLLVLFQIMGVMPIHRNPPEKNLPRTGYSWGSKQVMWAIFIYSCQTTIV
VLVLRERVK

KFVTSPDKRFDEAIYNVIFISLLFTNFLLPVASWRHGPQVAIFKNMWTNYQYKFFKTTGSPI
VFPNLYPL
TWSLCVFSWLLSIAINLSQYFLQPDFRLWYTFAYYPIIAMLNCFCSLWYINCNAFGTASRAL
SDALQTTI
RGEKPAQKLTEYRHLWVDLSHMMQQLGRAYSNMYGMYCLVIFFTTIIATYGSISEIIDHGA
TYKEVGLFV
IVFYCMGLLYIICNEAHYASRKVGLDFQTKLLNINLTAVDAATQKEVEMLLVAINKNPPIMN
LDGYANIN
RELITTNISFMATYLVVLLQFKITEQRRIGQQQA

>DmelGR64a for sugar taste
MKGPNLNFRKTPSKDNGVKQVESLARPETPPPKFVEDSNLEFNVLASEKLPNYTNLDLFH
RAVFPFMFLA
QCVAIMPLVGIRESNPRRVRFAYKSIPMFVTLIFMIATSILFLSMFTHLLKIGITAKNFVGLVF
FGCVLS
AYVVFIRLAKKWPAVVRIWTRTEIPFTKPPYEIPKRNLSRRVQLAALAIIGLSLGEHALYQV
SAILSYTR
RIQMCANITTVPSFNNYMQTNYDYVFQLLPYSPIIAVLILLINGACTFVWNYMDLFIMMIS
KGLSYRFEQ
ITTRIRKLEHEEVCESVFIQIREHYVKMCELLEFVDSAMSSLILLSCVNNLYFVCYQLLNVF
NKLRWPIN
YIYFWYSLLYLIGRTAFVFLTAADINEESKRGLGVLRRVSSRSWCVEVERLIFQMTTQTVAL
SGKKFYFL
TRRLLFGMAGTIVTYELVLLQFDEPNRRKGLQPLCA

>DmelGR43a for sugar taste
MEISQPSIGIFYISKVLALAPYATVRNSKGRVEIGRSWLFTVYSATLTVVMVFLTYRGLLFD
ANSEIPVR
MKSATSKVVTALDVSVVVMAIVSGVYCGLFSLNDTLELNDRLNKIDNTLNAYNNFRRDR
WRALGMAAVSL
LAISILVGLDVGTWMRIAQDMNIAQSDTELNVHWYIPFYSLYFILTGLQVNIANTAYGLGR
RFGRLNRML
SSSFLAENNATSAIKPQKVSTVKNVSVNRPAMPSALHASLTKLNGETLPSEAAAKNKGLLL
KSLADSHES
LGKCVHLLSNSFGIAVLFILVSCLLHLVATAYFLFLELLSKRDNGYLWVQMLWICFHFLRLL
MVVEPCHL
AARESRKTIQIVCEIERKVHEPILAEAVKKFWQQLLVVDADFSACGLCRVNRTILTSFASAIA
TYLVILI
QFQRTNG

>DmelGR66a for bitter taste
MAQAEDAVQPLLQQFQQLFFISKIAGILPQDLEKFRSRNLLEKSRNGMIYMLSTLILYVVLY
NILIYSFG
EEDRSLKASQSTLTFVIGLFLTYIGLIMMVSDQLTALRNQGRIGELYERIRLVDERLYKEGC

VMDNSTIG
RRIRIMLIMTVIFELSILVSTYVKLVDYSQWMSLLWIVSAIPTFINTLDKIWFAVSLYALKERF
EAINAT
LEELVDTHEKHKLWLRGNQEVPPPLDSSQPPQYDSNLEYLYKELGGMDIGSIGKSSVSGSG
KNKVAPVAH
SMNSFGEAIDAASRKPPPPPLATNMVHESELGNAAKVEEKLNNLCQVHDEICEIGKALNEL
WSYPILSLM
AYGFLIFTAQLYFLYCATQYQSIPSLFRSAKNPFITVIVLSYTSGKCVYLIYLSWKTSQASKR
TGISLHK
CGVVADDNLLYEIVNHLSLKLLNHSVDFSACGFFTLDMETLYGVSGGITSYLIILIQFNLAA
QQAKEAIQ
TFNSLNDTAGLVGAATDMDNISSTLRDFVTTTMTPAV

>DmelGR33a
MIQIMNWFSMVIGLIPLNRQQSETNFILDYAMMCIVPIFYVACYLLINLSHIIGLCLLDSCNS
VCKLSSH
LFMHLGAFLYLTITLLSLYRRKEFFQQFDARLNDIDAVIQKCQRVAEMDKVKVTAVKHSVA
YHFTWLFLF
CVFTFALYYDVRSLYLTFGNLAFIPFMVSSFPYLAGSIIQGEFIYHVSVISQRFEQINMLLEKI
NQEARH
RHAPLTVFDIESEGKKERKTVTPITVMDGRTTTGFGNENKFAGEMKRQEGQQKNDDDDL
DTSNDEDEDDF
DYDNATIAENTGNTSEANLPDLFKLHDKILALSVITNGEFGPQCVPYMAACFVVSIFGIFLE
TKVNFIVG
GKSRLLDYMTYLYVIWSFTTMMVAYIVLRLCCNANNHSKQSAMIVHEIMQKKPAFMLSN
DLFYNKMKSFT
LQFLHWEGFFQFNGVGLFALDYTFIFSTVSAATSYLIVLLQFDMTAILRNEGLMS

>DmelGR10b
MRVGKLCRLALRFWMGLILVLGFSSHYYNPTRRRLVYSRILQTYDWLLMVINLGAFYLY
YRYAMTYFLEG
MFRRQGFVNQVSTCNVFQQLLMAVTGTWLHFLFERHVCQTYNELSRILKHDLKLKEHSR
FYCLAFLAKVY
NFFHNFNFALSAIMHWGLRPFNVWDLLANLYFVYNSLARDAILVAYVLLLLNLSEALRLN
GQQEHDTYSD
LMKQLRRRERLLRIGRRVHRMFAWLVAIALIYLVFFNTATIYLGYTMFIQKHDALGLRGRG
LKMLLTVVS
FLVILWDVVLLQVICEKLLAEENKICDCPEDVASSRTTYRQWEMSALRRAITRSSPENNVL
GMFRMDMRC
AFALISCSLSYGIIIIQIGYIPG

>DmelGR94a
MDFTSDYAHRRMVKFLTIILIGFMTVFGLLANRYRAGRRERFRFSKANLAFASLWAIAFSL
VYGRQIYKE

YQEGQINLKDATTLYSYMNITVAVINYVSQMIISDHVAKVLSKVPFFDTLKEFRLDSRSLYIS
IVLALVK
TVAFPLTIEVAFILQQRRQHPEMSLIWTLYRLFPLIISNFLNNCYFGAMVVVKEILYALNRRL
EAQLQEV
NLLQRKDQLKLYTKYYRMQRFCALADELDQLAYRYRLIYVHSGKYLTPMSLSMILSLICH
LLGITVGFYS
LYYAIADTLIMGKPYDGLGSLINLVFLSISLAEITLLTHLCNHLLVATRRSAVILQEMNLQHA
DSRYRQA
VHGFTLLVTVTKYQIKPLGLYELDMRLISNVFSAVASFLLILVQADLSQRFKMQ

>DmelGR97a
MRFLRRQTRRLRSIWQRSLPVRFRRGKLHTQLVTICLYATVFLNILYGVYLGRFSFRRKKF
VFSKGLTIY
SLFVATFFALFYIWNIYNEISTGQINLRDTIGIYCYMNVCVCLFNYVTQWEKTLQIIRFQNS
VPLFKVLD
SLDISAMIVWRAFIYGLLKIVFCPLITYITLILYHRRSISESQWTSVTTTKTMLPLIVSNQINN
CFFGGL
VLANLIFAAVNRKLHGIVKEANMLQSPVQMNLHKPYYRMRRFCELADLLDELARKYGFT
ASRSKNYLRFT
DWSMVLSMLMNLLGITMGCYNQYLAIADHYINEEPFDLFLAIVLVVFLAVPFLELVMVAR
ISNQTLTRRT
GELLQRFDLQHADARFKQVVNAFWLQVVTINYKLMPLGLLELNTSLVNKVFSSAIGSLLI
LIQSDLTLRF
SLK

>DmelGR9a
MSLWLEHFLTGYFQLCGLVCGWSGSRLGRLLSSTFLVLILIELVGEIETYFTEENPDNESVPA
YFAKVIM
GVNMAYKMIHAWIALSALFECRRFRYLLEELPPVKATSFIYRHLILEIILFACNAFLVLSEYT
IRGIYLE
NLRYAYSLQAVRARYLQMMVLVDRLDGKLEQLHHRVISGSSDYKTLRLDYAHLAKVTRS
LSHLFGLSLLL
LNVLCLGDWIIVCNVYFMVAYLQVLPATLFLFGQVMFVVCPTLIKIWSICAASHRCVSKSK
HLQQQLKDL
PGQTPVERSQIEGFALQIMQDPIQIDVCGIYHLNLQTLAGMFFFILEALVIFLQFVSLVRT

>DmelGR98a
MEQMSGELHAASLLYMRRLMKCLGMLPFGQNLFSKGFCYVLLFVSLGFSSYWRFSFDYE
FDYDFLNDRFS
STIDLSNFVALVLGHAIIVLELLWGNCSKDVDRQLQAIHSQIKLQLGTSNSTDRVRRYCNWI
YGSLIIRW
LIFIVVTIYSNRALTINATYSELVFLARFSEFTLYCAVILFIYQELIVGGSNVLDELYRTRYEM
WSIRRL
SLQKLAKLQAIHNSLWQAIRCLECYFQLSLITLLMKFFIDTSALPYWLYLSRVEHTRVAVQ

HYVATVECI
KLLEIVVPCYLCTRCDAMQRKFLSMFYTVTTDRRSSQLNAALRSLNLQLSQEKYKFSAGG
MVDINTEMLG
KFFFGMISYIVICIQFSINFRAKKMSNEQMSQNITSTSAPI

>DmelGR98b
MVAQKSRLLARAFPYLDIFSVFALTPPPQSFGHTPHRRLRWYLMTGYVFYATAILATVFIVS
YFNIIAID
EEVLEYNVSDFTRVMGNIQKSLYSIMAIANHLNMLINYRRLGGIYKDIADLEMDMDEASQ
CFGGQRQRFS
FRFRMALCVGVWMILMVGSMPRLTMTAMGPFVSTLLKILTEFVMIMQQLKSLEYCVFVLI
IYELVLRLRR
TLSQLQEEFQDCEQQDMLQALCVALKRNQLLLGRIWRLEGDVGSYFTPTMLLLFLYNGLT
ILHMVNWAYI
NKFLYDSCCQYERFLVCSTLLVNLLLPCLLSQRCINAYNCFPRILHKIRCTSADPNFAMLTR
GLREYSLQ
MEHLKLRFTCGGLFDINLKYFGGLLVTIFGYIIILIQFKVQAIAANRYKKVVN

>DmelGR89a
MLRFPHVCGLCLLLKYWQILALAPFRTSEPMVARCQRWMTLIAVFRWLLLTSMAPFVLW
KSAAMYEATNV
RHSMVFKTIALATMTGDVCISLALLGNHLWNRRELANLVNDLARLHRRRRLSWWSTLFL
WLKLLLSLYDL
LCSVPFLKGAGGRLPWSQLVAYGVQLYFQHVASVYGNGIFGGILLMLECYNQLEREEPTN
LARLLQKEYS
WLRLIQRFVKLFQLGIFLLVLGSFVNIMVNIYAFMSYYVSLHGVPLTISNNCLVLAIQLYAVI
LAAHLCQ
VRSAKLRKKCLQLEYVPEGLTQEQAMASTPFPVLTPTGNVKFRILGVFILDNSFWLFLVSY
AMNFIVVIL
QTSFEHINHGEI

>DmelGR36a
MFDWVGLLLKVLYYYGQIIGLINFEIDWQRGRVVAAQRGILFAIAINVLICMVLLLQISKKF
NLDVYFGR
ANQLHQYVIIVMVSLRMASGISAILNRWRQRAQLMRLVECVLRLFLKKPHVKQMSRWAIL
VKFSVGVVSN
FLQMAISMESLDRLGFNEFVGMASDFWMSAIINMAISQHYLVILFVRAYYHLLKTEVRQAI
HESQMLSEI
YPRRAAFMTKCCYLADRIDNIAKLQNQLQSIVTQLNQVFGIQGIMVYGGYYIFSVATTYIT
YSLAINGIE
ELHLSVRAAALVFSWFLFYYTSAILNLFVMLKLFDDHKEMERILEERTLFTSALDVRLEQS
FESIQLQLI
RNPLKIEVLDIFTITRSSSAAMIGSIITNSIFLIQYDMEYF

>DmelGR58a
MLLKFMYIYGIGCGLMPAPLKKGQFLLGYKQRWYLIYTACLHGGLLTVLPFTFPHYMYD
DSYMSSNPVLK
WTFNLTNITRIMAMFSGVLLMWFRRKRILNLGENLILHCLKCKTLDNRSKKYSKLRKRVR
NVLFQMLLVA
NLSILLGALILFRIHSVQRISKTAMIVAHITQFIYVVFMMTGICVILLVLHWQSERLQIALKD
LCSFLNH
EERNSLTLSENKANRSLGKLAKLFKLFAENQRLVREVFRTFDLPIALLLLKMFVTNVNLVY
HGVQFGNDT
IETSSYTRIVGQWVVISHYWSAVLLMNVVDDVTRRSDLKMGDLLREFSHLELVKRDFHL
QLELFSDHLRC
HPSTYKVCGLFIFNKQTSLAYFFYVLVQVLVLVQFDLKNKVEKRN

>DmelGR22a
MSQPKRIHRICKGLARFTIRATLYGSWVLGLFPFTFDSRKRRLNRSKWLLAYGLVLNLTLL
VLSMLPSTD
DHNSVKVEVFQRNPLVKQVEELVEVISLITTLVTHLRTFSRSSELVEILNELLVLDKNHFSKL
MLSECHT
FNRYVIEKGLVIILEIGSSLVLYFGIPNSKIVVYEAVCIYIVQLEVLMVVMHFHLAVIYIYRYL
WIINGQ
LLDMASRLRRGDSVDPDRIQLLLWLYSRLLDLNHRLTAIYDIQVTLFMATLFSVNIIVGHVL
VICWINIT
RFSLLVIFLLFPQALIINFWDLWQGIAFCDLAESTGKKTSMILKLFNDMENMDQETERRVT
EFTLFCSHR
RLKVCHLGLLDINYEMGFRMIITNILYVVFLVQFDYMNLKFKTD

>DmelGR2a
MDTLRALEPLHRACQVCNLWPWRLAPPPDSEGILLRRSRWLELYGWTVLIAATSFTVYGL
FQESSVEEKQ
DSESTISSIGHTVDFIQLVGMRVAHLAALLEALWQRQAQRGFFAELGEIDRLLSKALRVDV
EAMRINMRR
QTSRRAVWILWGYAVSQLLILGAKLLSRGDRFPIYWISYLLPLLVCGLRYFQIFNATQLVRQ
RLDVLLVA
LQQLQLHQKGPAVDTVLEEQEDLEEAAMDRLIAVRLVYQRVWALVALLNRCYGLSMLMQ
VGNDFLAITSN
CYWMFLNFRQSAASPFDILQIVASGVWSAPHLGNVLVLSLLCDRTAQCASRLALCLHQVS
VDLRNESHNA
LVGTLVRYCAPLIILVPLQITQFSLQLLHQRLHFSAAGFFNVDCTLLYTIVGATTTYLIILIQF
HMSEST
IGSDSNGQ

>DmelGR22b
MFGSSREIRPYLARQMLKTTLYGSWLLGIFPFTLDSGKRIRQLRRSRCLTLYGLVLNYFLIF
TLIRLAFE

YRKHKLEAFKRNPVLEMINVVIGIINVLSALIVHFMNFWGSRKVGEICNELLILEYQDFEG
LNGRNCPNF
NCFVIQKCLTILGQLLSFFTLNFALPGLEFHICLVLLSCLMEFSLNLNIMHYHVGVLLIYRYV
WLINEQL
KDLVSQLKLNPETDFSRIHQFLSLYKRLLELNRKLVIAYEYQMTLFIIAQLSGNIVVIYFLIV
YGLSMRT
YSIFLVAFPNSLLINIWDFWLCIAACDLTEKAGDETAIILKIFSDLEHRDDKLEMSVNEFAWL
CSHRKFR
FQLCGLFSMNCRMGFKMIITTFLYLVYLVQFDYMNL

>DmelGR22c
MFASRSDLQSRLCWIILKATLYSSWFLGVFPYRFDSRNGQLKRSRFLLFYGLILNFFLLLKM
VCSGGQKL
GIPEAFARNSVLENTHYTTGMLAVFSCVVIHFLNFWGSTRVQDLANELLVLEYQQFASLNE
TKCPKFNSF
VIQKWLSVIGLLLSYLSIAYGLPGNNFSVEMVLINSLVQFSFNCNIMHYYIGVLLIYRYLWL
INGQLLEM
VTNLKLDCSVDSSRIRKYLSLYRRLLELKGYMVATYEYHMTLVLTTGLASNFLAIYSWIVL
DISMNINFI
YLLIFPLFLLVNVWNLWLSIAASDLAENAGKSTQTVLKLFADLEVKDIELERSVNEFALLC
GHCQFNFHV
CGLFTINYKMGFQMIITSFLYLIYMIQFDFMNL

>DponGR3
MITGRILDLVIGALIFASLTESLPAQLKERNLLYSDPGVVAVYIRPGDTPLQDINPDLAEAFN
FNDVKYG
RRAFGRDINKILNEKKENSVLFSSGELAEGESYAPKKKSLKERGESHIQKIPKH

>DponGR1
MSKPPMAAQMDGQFLSPYPPEGPDVSRRKSDNIRIVTPETVRPEHVPDNELLEKLHTYDN
FYQTTKSLLI
LFQIMGVMPIQRLRGKTIFRWFSANTCWAYFVYTVETIFVSIVFKERLVLILKPGKRFDEYI
YGVIFLSI
LIPHFLLPLGAWRNGQEVARFKNMWTKFQLKYFKMTGTVIKFHRLTLTTYSLCVLSWLVG
ILIMLAQYYL
QPDMLLWHTFGYYHILAMLNCLCTLWYINCTAKGRVAGWIAEKLQEALQTKGSAKKLLT
IGKLWVDLSHM
MQQLGTAYSGMYALYCLLVLLTTI

>DponGR64f for sugar taste
EHILFILTNLNHSFHCEHYNKYPVEIYFGCAFPQWFTLITYSHWAGALVELTNFISTFTWNF
TDLFIIMI
SISLREKFNQISNRIKQSKNPPHKFWKEIREDYYRVSNLTKVVDIQIAGLVLISFLNNIFFLCI
QLYNSI

KEREAVIDSIYFFYSFGYIVFRVVAVSLYSATLNEAARKPLKYLYSLPTENYTIDVSRLITQIN
YLPNGI
TGHGFFLITKNFLLQVSCQLYSC

>DponGR24
MYNNPGTPLPHGMMDNSYQIPNQNIYFDDGMQFYRTKNQNTITKVAPVPPPKYSNAENM
EMMESIDDKSV
KKSVIFDNVKPIFVTMRLLGILPVVRRGSIFIISTKWIIYSFLLLLAVAGFIGYLKYYNINVTR
TAEGRF
EEAVIDYLFTVYLVPIFVNILALYEAKKQANVLTQMVSFERIYTRMFKKRIGFDLGSKPLV
MTVVLLILG
CGVMVITHFSMANFIIYQVVPYCYINVITFIIGGAWYIYCDIIGNIATCLAEEFQFALRNIEYS
NRVCDY
RSLWMILSKIIRNVGNSFGYTLTFLCLYLFFVITLTVYGLLSQIQEGMGVKDIGLTITGVSAT
LMLYFIC
DEAHYASTCVRTYFQKKILLVELSFMNEDAQQEISMFLRATEMNPTDMCLCGFFDVNRNL
FKSLLATMVT
YLVVLLQFQISIPSSGDDVTNNTTNTTPSAPK

>DponGR64e for sugar taste
MVKSVFYNFSRTKMRVLRKINSDTTLTIEENNVNNIQMYNRPHIFYENIRFALILSQCFGIM
PLHNISKN
AQEVKFSFMSFRFLYALAHFGCVFATGIASIVKLALNGFMIDETSIVSFYIFNSFASIHFMYL
ATRWQNI
LKEYSYVERAMKNYASNRNVKRINICTATFFMFFGLTEHILFILTNLNHSFHCEHYNKYPV
EIYFGCAFP
QWFTLITYSHWAGALVELTNFISTFTWNFTDLFIIMISISLREKFNQISNRIKQSKNPPHKFW
KEIREDY
YRVSNLTKVVDIQIAGLVLISFLNNIFFLCIQLYNSIKEREAVIDSIYFFYSFGYIVFRVVAVSL
YSATL
NEAARKPLKYLYSLPTENYTIDVSRLITQINYLPNGITGHGFFLITKNFLLQAAATVVTFEL
MIFQFSPV
KTTSNQTSLSSGIICIK

>DponGR22
MNGKHSFRQLDIKDLEDLYGEQLDIKSMANMRGSPRARDIAKRFKLDENDGRNIDDHDQ
FYRDHKLLLIL
FKWMGVMPVERGEIGKITFSWTSKPMLYAYGFYVITTIVVLLVGYERVDILLNKSRKFDEY
IYAIIFIVF
LVPHFVVPFVGWSVAHQVCDYKNSWGRFQLNYYKITGRDLEFPYLSTLIGTISLGCLFLAV
SFLLSLSTL
LDGFALYHTIGYLHIITMINMNCALWYINCRAIGNASTGVAESFKKDIHSYCVAYIIKHYRV
LWLELSEI
LQKLGNAYTRTYSSYCLLMMTNIIISVYGFTSEVVDHGVKFTFKEMGLLVDAIYCFTLLYIF

CDCSHQAS
ANIAERVQWALMEINLNQVDHATIKEIQMFLKAIHLNPPKVSLRGYTVVSRELVTAMISTI
AIYLIVLLQ
FKISLVNMRG

>DponGR28b
MTNAVAASEYTNEQDCKKMLSDTRKLYTEMHKVIETFNTLFGYVLLLMAVQCSLQILDF
GVFLIEMVLPN
FKFEYDAFFIYMGFILLDLIWLSVAQSRCEMAKDESMKLRSACYNLLDNSSGSGDLHQEL
KALIKQIKNR
PVRFTAADFFEIKRSTTFTIVGATATYFIVYVELRSNDNTTWNNGTL

>ItypGR6
MSYNGDVIKYPREITVFAAVCAVVFCVLGIVGNSITILALFRCPKLKSHATTAFVISLCVSDL
LFCGFNL
PLTAARYIAEEWIFGDTLCQLFPVFFYGNVALSLLNMVAITLNRYVLITLYDYYTKFYSKLS
ICLQLFCT
WLIAFLIMMPPLTGIWGQLGLDPSTFSCTILEKDGRSPKKTLFLFGIGFPCLVIILAYSCIYWT
VRNSKL
RLRSHEPLPNQRTSKRDRDDRRLTRLMALIFICFVLCFLPLMLVNVFDDKVSYPTLHVLASI
MAWASSVI
NPFIYAATNKQYRSAYKRLLALVRSSIGPESMHSNSLRSKQEKFQGVSYKAK

>ItypGR4
MQFVNIVELNALEFHLEGVLLNLIFLYMAKIWAAFVREWSLIEKSMKYYEGPKNAKIKIVV
AMVSIMGIA
LFEHALVTSQIAWTSIRNNPLSFFNASREYFVHHEFVEVFHFVPYSIWTGLFFKLLIIQKTFI
WSFIDVF
ISTVSICFHCKMVQISRKVARLSAYEEKNCMIWRSTREDYTKLSKLCQIVNSKLRWLIILSF
FNNVYHIL
SQLFNSLKPNDDAMHKIYFCISFTLLILRMTTVCVYAGSICDEQDRLITVLTTAPSGVYNIE
VERFIMHL
DTFEMALSGSNFFKITKGLLLKISSAIVTLRISVNSV

>ItypGR3
MVETVHPITKVAPAPPPRYSNGDLMEAFDDSGELNSRIFSCLKPAYATLRLFGLMPVTQSGP
VFHVTAKW
IIYSFMLLCSLAGFLGYLKYFNIAITRNAEGRFEEAVIDYLFTVYLLPVALNVIAMYEASKQ
AGVLTQIV
AFERIYTRTFRKRLTLDMGSKQLILISVLLILGCVVMVVTHFTMANFIVYQVVPYCYVNIV
TYIIGGSWY
IYCDVIGKVATSIAEEFQFALKNAEHSSRVADYRSLWMMLSKIIRNVGNSFGYQLTFLCLYL
FFVITLTV
YGLLSQIQEGMGIKDIGLTITGVSAVTMLYLICDEAHYASSCVRTYFQKKILLMELSLLNEE

AQQEINMF
LRATEMNPTDMCLCGFFDVNRNLFKSLLATMVTYLVVLLQFQISIPAGNDNLTNSTLNS

>ItypGR2
LMMTNIIISVYGFTSEVVDHGVKFTFKEMGLLVDAVYCLVLLYIFCDRSHKASENXAEXV
QWTLMEINLR
QVDEATVREVQFF

>ItypGR5
MSVLFWKGIREDYDRLASFXXELDSHISVLVLLSYFLNIFFLLIQLYHGLE

>LdecGR64d for sugar taste
MTSKFEQIFKRLEINKVTHEKFWKEIRHDYQKLYNLSKSLEKHLSFLVLVSYFHNIFFLCIQ
LYNSLSER
TGAIETAYFFISFGFLVLRILTVSMYGAWLNE

>LdecGR64f for sugar taste
MDTTGMNVNPVNLALEEVFIFHLSGLIQFVLFFKLTYYWPRFVEEWEKVEVNMRKYQVF
TNLRLKLNLMT
SCFMFYAIVEHSLVSAYKLKKWIDSSQNTSEALQNYFVETYDHVFTYVNYSLWIGLALQY
FNLQRTFNWS
FADVTIMLIGTALTFRLKQMSRRIERISTLKVNNINQWKVVRKDYIRLTELCYEVNTRLSTII
MISFLTN
MYFVLLQIFLSMNPMENLVQKIYFYLSFGLILIRIVGLCVFGGAVYEEWRNLGFLLNCVVT
TTYNQEVER
MACHVISWELSLSGKNFFHISRSLILKIAGAVVTYELVLIQFYRNAQYNSM

>LdecGR22
MHGFDSFNRKNPHQLRCIPHNLLPPQKILVTGTPAFKTVNQIDPEVLHQYDNFYHTTKSLL
VLFQIMGVM
PIEREVGRTTYRWLSPTNLWAYFIFTIETILVSIVFKERLKLVLLPGKRFDEYIYAIIFLSILIPH
FLLP
IGAWTNGSEVAKFKNMWTKFQYKYFTVTGTPVVFRNLSAITISLCIVSWIVGIVIMLAQYY
LQPDMLLWH
TFGYYHILAMLNCLCCLWFINCTAKGRVAGWLAENLHNALQSTDPASKLAEYRDLWVDL
SHMMQQLGKAY
SGMYGMYCILILLTTIVASYGCLTEILDQGLSFKEAGLFLISFYCMSLLFIICNEAHSASHKM
GAEFRER
LLNVNLAAVDNRTRQEVNMFLTAIDKNPPIMNLNGYANINRKLISSTVTSMATYLVMLMQ
FRLSLMRNVS
IALKRATQGAAVNSTPFENTTIS

>LdecGR64e for sugar taste
MATKNSENCFHRDIKYILRVCQIFGLFPVSELTSEDHRNIKFEWKSTLTVYSFLLTLSTLICT

VLTFIQL
VTHEFNLHSMDSFMFCFFAFAISLSFYKLAMDWSELLKSFREIEIAFKNYSYYQNLRKQFTI
MFVISTTG
TLIEHSLARVATYNQVDPQAKSNRTQLELYLKKEYSFVFDHMEYSNPVASFSFYISLYFWT
KVREDYSLI
SELCQKVNDKIGHMILISFASNMYFVLSQLYKGLTIKRTIIDGIYFIFSFGIVILRLSGVILYGS
EVNLQ
SQKPLRYLISVKSDYYNEEIERFILEIHCHETSLSGNQFFTLKRGLIFGMATAIITYELFMLQT
KIN

>LdecGR24
MIMNSDNFNISLIEGIPRNNSRRRSVYLEGTQPFYQKSGKNPNPVIKVAPYDPSGPGGPVRA
FHDSRELD
SVFFNSIKPMVYLLKAVGIFPIGNTGCVFQTTPQLLIYSIAVFVVIFAYICYIRWDKVEMVRS
AEGRFEE
AVIDYLFTVYLVPIVINPIAWYEAKKQARVLTNLVAFEKLYQKVTKNKFPLLLGNKSLIIAIG
LPVLATV
TMVVTHLTMVHFKFLQVVPYCYINMITYLVGGAWYLYCDLLGNIALTVAKDFKQTLKNI
GPSSRIADYRS
LWMMLSRLIRDVGNAFGYTVTFLCLYLFLIITLTIYGLMSQMQEGLGIKDVGLTITAVFATA
TLFFICDE
AHYGSNCVKVQFQKRLLLVELNWMNDDAQQEINMFLRATEMNPTDMSLGGFFDVNRNL
FKSLIATMVTYL
VVLLQFQISIPEDGSASGSASSNDNNSTKILR

>LdecGR43a for sugar taste
ILLVVSWPYQIYSILIVTTDNWMAFLMFYLNLYNNFSIIACEFQFVSLCWMIQTRFRAMNR
GIEELYSKV
LTADEELMKHKLIRLKKCYKLLIQSCSQLNKLYGFKLLIILSGLSLNVLFGLYFSIFGGFAKN
STKTSLN
FQRISNVANEIIWSFYYMVRFVFICVVADLLINAGYQSRKTISNIFCNSLDENVKQELWIFLT
LISTNKI
RLSACGFFTINKTLVTSAVSMGTTYLVILAQFQKK

>LdecGR28b
MSSDSLSIGKMELHHSLRPVYIFTKIFAHCQYKFDYQRRVYIPDLWAIVYSILFSLTTCLPFC
FEHSFID
DFGNVMAYTYSFSFVLYFELIIVNVIIFMHLLNVKKTAGVFTKLEEIDEELAEIVGERIDSRP
IFKFSIF
VITFFVVDSIFRVIYEFKVFYNDALYYCVSTFWNAILCDIFKVEFCVIMYVPLRRFRLLNSLF
QGLKMRG
NGRVIVEKHVESIETIHAIHNKLCSVCLDINDIFGIPTLLLFTHAVSSAVIQFYYTFLMRVHFS
WRVAAQ
VFAILHWTIFRLGSLVIIALLYHCILKQVANMKRTIHNLLHRIQDQSVKAALEIFALHEMNR

NLEFTVCG
LFSVDFRMIQMAFATIATYVVLLIQLKVVK

>PaenGR8
GVEHALVNAYKLKNKFDREDNFSEAVKYFFIDSYKHVFNMVDYSVWLGLLIQFLNLQRT
FYWNYTDVFIM
VIGSVLTYKLKQISKKIQDTATSRVNDVVSWKIIRKDYVRIAELCHVVNETISGVIITCFLMD
LYFVLLQ
LYKSLRPIESVIEKVYFYISFGLLLLRIFCLCIFGGAVFEEWKDIRFYLNTIATSAYNLEIERLT
NHVAT
WELSLSGKNFFSISRGLILQMAGAIVTYE

>PaenGR7
FRVVYSALNASGAFISFGFWLIKFCVDGLVVDKTAHMAFYISTFLCSIHLIKLAKHWNGIL
QEWSYIEMS
MRGYGNDVNLKKRYLWMTVTSFTLGTIEHALFIINGIYAADACASYVISPLRATMEVLFN
NFFSFITFNV
WLGILVKLINLLATFTWIFTDIFISLVSVALTAKFRQLVVRLKKNKVMHHQFWREIRQDYH
KLYILCKKM
DKHISFLVLISYMHNIFFLCIQLYNSLRERKGVVESTYFVFSFSFLVSRIIAVSMYGAWLHDE
ARKPMEY
LYNVPTEYYCNEISR

>PaenGR16
MNLLKILNGNGLLLKDRIASNYFQEVMKFPLKLAQTFSYFPIFIGDREEPLQFKWLYWRIG
YSLITFILF
IIELCFVVLETFKNPMNPLEIKVVVFHLGAVIQFILFFKLTYHWPNFVKEWTKIEYHMKTYE
IVENIRLK
LILIAGIIMFIAGGE

>PaenGR15
FSQSKKLAKWLNEICIFDKEIQKSNFKIDFKSEFFITFLMALSGSIISLAGIVFIVTVDVLCFY
PTSFKA
ANSNISYCAPIMALSLLFLSVCSILLVLKRRFKWINCGIQTLKEQLDEKYSTDTFTIPFRKSI
KSVDLNE
DVIVYALEDLRTKHWKMHSLVEEINSIYSFPLLFSFLQIFLAFQNYTLYFIKSPTTVYTQKTT
KLYQFFG
KGIIAIYAVQMVLMSSICSSLSYEVHKTG

>PaenGR6
MENIKDVTKNWTKRKLPTKRNHLNFHPDFSQTSHDFLKYILLTCQFIGLFPMHNICGKDPA
KIKFSWLSW
KIVYTIIISASSIFCSYSGYYRAFTFHPVITHLLFPVVFTQVAVATLISINVSKNWQKCITSITY
LELEI

NKKYGEPKNVRKYLMFLIFIIYAIGSIEHLFELISNALQFGSSGFINCIRERCNYFYHECNTC
WSAVFLV
FCTISWAVCIAIDVFIINLGVLFASRFKQIADSFESYNDALQIEETSHIYRLDNILKYYKQIRE
DYIKLT
SAVYQVDCLCGNLIICSYISNITSILIQLFNSLRHFNSLLHTIHFLYSFGFIIMRLVLVTIYASSV
NEES
KKIASIVLKVPDDIWSQDLQRIISHIYYDSTALTGGQFFKITRSLTLNVDSCFHY

>PaenGR14
MMFSKKVSKSKENENELICMQFMTKICALAGLFPGNSNTNINKIYVIVLTTASIVGWIYSV
YIKNNFMLT
GCNVFIKITDQISCGLLCLATVTFSLTSTFVYPGKFFNAIQSLKRFDYLINISSPRTVSMGLAI
FIFGNA
VLLFPMVLDTLAWINRYDLNTYKCYFIRNFQYYQLGVITFLWLWFALEIRKRFVLFNKFLI
KMIPTPSIL
LRNDAHHHGLFKMANIPQIRDEIKHKTALYNGLCDIVDLINDVFGAGLLIYVIFTISYVLLY
SIILIEYT
VFDSKTRIDNYIIITSIVWIIGDFVKMMVVALAGETLSSESFKTVRICYGIINSLENGPNQYY
DGIKQEL
NFLIQQATHRKPCLTASGFFVANSTMMGFIIGSITSYVIVAVQFLNEMSN

>PaenGR13
VSKDSIDSKNVSKMFFYFSLFGVTIHGNSKIYKFYVVFMLSLLTKVLLLATSGEHLSKEAN
KTILICYEI
LNNTRGENAHLMKEELQFLIQQAAFRKPRLSAAGFFVVDFSMMGFMIGSITSYVIVSVQF
LKETINNYS

>PaenGR12
KKLSRVLTNLMAFERIYYKMTKKKFSQILGNKPLVITVALPVLACATMVVTHVTMAHFKF
LQVVPYCYIN
MITYLICGVWYMLCDLLGNIALTVVDDFDQILKNISPANKIAEFRSLWMLLSRIIRDIGNAF
GFTLTFLC
LYLFLIITLTIYGLMSQIQAGLGIKDIGLAITGFFAGLMLLLISDEAHYASNCVKVQFQKKLL
LVELNWM
SDDAQQEINMFLRATEMNPTDMTLGGFFEVNRNLFKSLIATMVTYLVVLLQFQISIPEDGD
EGDSTTKH

>PaenGR5
MHEATVFKDTLIVRSFYKFGSFMGVFPSNFATGELSLRITIVVTTITLISLIRFYMYVSQETII
LQISTI
IDFAEIFSYYIFSILTLRSLIRNKRMWKEFFGTIANIDKEVGMWKEFFGTIANIDKEVGIGYT
NKYLLLL
RVLFWIALDLAASIVIFNSGTSILIAVYVIMVILQMTAISVFVLESCLLIQKRQDMLSSKLETF
RQEMFG

KEFDREATSVERAIKNIYFVADTISSAFGWLFFSIFILNFIIMFVALDKMVETSFDDTITYSEG
FINNIY
NYAYLGIFFAMTISLAIAGDKIEKTGLKIKKLCHVLQVDVDNPIIKAHLREFAVYFEELRPVL
TVSGFFV
INRNMIPLLISSLTSYAVILIQLKSGP

>PaenGR4
MIYRNSQLLLSRTFFFYYTFLTATVIYNMALSLRNCLVGLKIYIVKSLGVESSVFLSETKSLR
TIMTPIS
VIGEETFWKLLEASKIFNIIFECVEIFNDIFGVLILFFNVVTVLIILVSLNTLLITGEMEQLTVE
VAIDS
ISVCLMFIIWNSFVTSACGLLKRESVTIITLCCKLQHALPHGSKERQEILNLARQINRKSPRV
TAAGFCD
VDFSLIFSIFSCVGTYIVVLIQFSHIDF

>PaenGR11
RMNKPMSRTEMLYIGLGHLVFILTRFYYHWFKMHHVDWFVILILVYEVMQHYYFFLSTLT
ICHYVRIIQY
GFKHAVDLLKQYNKSEMTDVELVEKIRYIGKDCRYLFRIGENNNDIFGWTILLIFLNGIVQ
VLRTTNLML
YLLQGSHLTLGRAFIFYSKTLLCFAETIYIIISCESSSRAGGRIRLVCHKLQEGVHIKSSARDE
FFKLAT
VVTTFSPKLTAANFFVINRGTLLGIVNLSTTYLLVILQFYMRESKEAEQQMNSTRNFNITCE
NESTIILQ

>PaenGR10
DLTEIRKLYSKLNDGLRMFNKLFGWPLLFLFSGILMDIIICINYVIAPNSTQTITLQTFLKESF
EAIISM
VSLVVLVMSCDSIESKAQEFINFSYLLQSNMEKSALRDELFYMVKWAKELAPKCSVAG

>PaenGR9
KMFGQNIHTIEDVTSERKRSVYLEGAVPFYQKPEKNSNPVTKVAPFQPRGPGPGHNNIPKT
FLKKDGYNS
VLYGSLKPLLTSMKIVGICPIADQELVFQVTLRLLIYSACIFVVILGYIGYIQWDKL

>PaenGR3
MEKKVADLSANERKLTESYQLVVETMGVNKFFGLGPLAGKSKFSAIFFVCATLTASVFAL
KNRFHSLGER
SPIVGRMALGQPLFEALFSLYCFVYFSFFAKDCKKDLIDAMNKLELSVNVRFKENFHWLK
PRIIIILIEF
LALYIYTFYISITNNRFNILIILSAQFIAVNCTLQICVYYLVITNWLRNKYDNFNSMIVSICEK
PYSKFD
EKIDNIIDYLEKSSTVVKLNNKLFGTSIFTSNCLFIYIILYTTVSLFDGREREDSTLNTLKCIFP
IPFVL

YLIVLAMACDAVEKSGKRVIKTCYLFVEELKDAAKKEQLRLLARYAEQWCPVFSAAGFY
DINQSCLTSIF
SAILTYFVIIIQFNMVLSDQ

>PaenGR2
MLKSEKSLKLVEKITAAPPPINADFKLLTNYLGAANFFGVIYFFNKGYCFKTFGLLLTILVA
GVAFFSFK
VQKIIYENLHPTLKGMSIVLTVTDTVFLTFILVSLMLRKREHWKKLFETMHIFENILNTCDY
LDNENGMW
RISLKIILISTTILHSIYSLYYYIFENIFEAFQIILVLRGLVNLYLILITTFYLTLTNWLRNRYTFM
HKY
LTTTITKPGYFLIVRKVIITFKLAETVVQKTNHLFGSIIFTSLAVCVINILYYFVLALDLTSIST
EAKCI
NYIAPLIYAVFLVVLTMSCNSVETSGHSLIKTCYLLHESAELDVDKDHLMLLVKYVEEWRP
IFSAAGFYD
VNQASLSSIFSSIITYLVIAIQFNMVLA

>PaenGR1
TLQFITVTQDNLGLPLESLSLFYGVTVVQIFVFSVMVILSCDKVEKKAEEFIKTCIYIQASTG
DENALAL
ANLAKDLRPKFSAAGFFDINQRILPTFFSNLSTYLIIILQFKFSSL

>PmacGR8
MDLYFVLLQLYKTLRPIESVVEKVYFYISFGLLLLRIFCLCIFGGAVFEEWKDIRFYLNTIAT
SAYNLEI
ERLTNHVATWELSLSGKNFFSISRGLILQMAGAIVTYELVLIQFSSSTT

>PmacGR7
MHHQFWREIRQDYHKLYILCKKMDKHISFLVLISYMHNIFFLCIQLYNSLRERKGVAESTY
FVFSFSFLV
SRIIAVSMYGAWLHDEARKPMEYLYNVPTEYYCTEISRLIDQMYTSPVGITGSGFFIVTRNF
LLQMAGTI
VTFELMLFQFAPLDSKNRAYNRSISCI

>PmacGR10
FTIQIHLKMSSADQHQNLGSLISDVGHLSTEFILVLILVIAGDRVEKEGLKIIKVSYLVNREN
YYVEDPN
MQEQLRNFSVFLENLRPTLTAADYVTLNRNLIPILVSTVATYTIVLIQINLF

>PmacGR6
SSMVCSYSGYYRAFTFHPVITHLLFPVVFTQVAVATLISINVSKKWQKCITSITYLELEINKK
YGEPKNV
RNYLKFLIFIIYAIGSIEHLFDLISNVLQFGSSGFINCIHERCNYLYHECNTCWSAVFLVFCTIS
WAVCI

AIDVFIINLGVLFASRFKQIADSFERYNDVLQIEETSHIHRIDHILKYYKQIREDYTKLTSAVY
QVDCLC
GNLIICSYISNITSILIQLFNSL

>PmacGR9
YSDMIVKKIGKHMLEDTLFLQCIFRIGSIFALFPWNVKTCEVFIQHTLMIILNIATITSVYVSI
EKDGDS
IYSGQILVFVHSAFHLTLSISYFVTLVLNKNRWKRFFITFSRIDKNLGKCYISRKTIALKSMS
WIFLLAL
GLSVFISLLQFFSKLVFVTYMMLFIELFIITMFIFEASEMVNNRLIKCQKYLDLTCKNRINVK
EDFIKNI
KRIESAHRDIFLIIEILNSAFGVQILLLFVYDFLETLADINYALDNNASFTIKWDALYLFVICA
MSISLA
IAGDKIEKAGYNLINKCHILEVEVENTTTKEILQDLSSFLEDLRPTLTVAGFIDLNRTVIPLLL
STVTSY
AIILIQLKL

>PmacGR5
MHEAIVFKDTLIVRSFYKFGSFMGVFPPNFATGELSLPITIVITTIILLCLIRFFLDISQETIILQI
STF
INLAEIFSYYIFSILALRGLIRNRRIWKEFFGTIANIDKDVGVGYTNKNLLLLRVSFWIALDL
AATIVIF
NSGTSIFITLYWIMVILQMTAISVFVMESCLLIQKRQNMLSSKVETFRQEMFEKEFDLEATSI
ERAIKNI
YFVADTISSALGWLFFSIFILNFIIMFVALDKMLETSFDDKITYSEGFINNIYNYAYLGIFFAM
TISLAI
AGDKIEKTGLKIKKLCHVLQVDVDNPIIKNHLREFAVYFEELRPVLTVSGFFVINRNMIPLLI
SSLTSYA
VILIQLKSGP

>PmacGR4
MQTYYFVCVDYVSRMSTLIKMLSTILENFASLYSLTLSFFGLFLPSKDFSNWNRYEILRKYS
GLLYATLT
TIGYCYLQWYASQNTYKTSRTSNVILSTSTDTLLTIICLISTLGNAFCKKGKWIRLLKLQNF
CRKRTRID
GFWRNKFYLVLEVLFGHAIFCFVVGAAQYKNVIEDDLSKLAATFIKNVFFYYTFLIATVIY
NMALSLRNC
LVGLKIHIVKSLDVESSVFLSETKSLRTIMTPISVIGEETFWKLLEASKIFNIIFECVEIFNDIF
GVLIL
FFNVVTVLIILVSLNTLLITGEMEQLTVEIAIDSISVCLMFIIWNSFVTSACGLLKRESVTIITL
CCKLQ
HALPHGSKERQEILNLARQINRKSPRVTAAGFCDVDFSLIFSVFSYVGTYIVVLIQFSHIDF

>PmacGR3

NKYDNFNSMIVSICEKPYGKFDEKIDNVIDYLEKSSIVVKLNNKLFGTSIFTSNCLFIYIILYT
SVSLFD
GREREDSTLNTMKCIFPIPFVLYLIVLAMACDAVEKSGQRVIKTCYLLVEELKDAEKKEQF
RLLARYAEQ
WCPVFSAAGFYDINQSCLTSIFSAILTYFVIIIQFNMVLSDQ

>PmacGR2
MLKSEKSLKIVGKITAAPPPINADFKLLTNYLGAANFFGVIYFFNKGYCFKTFGVLLTILVA
VVAFFSFK
VQKIIYENLHPTLKGMSFVLTVTDTVFLAFILVSLMLRKREHWKKLFETMHIFENILNTCD
YLDNENGMW
RISLKIILISTTILHSIYSLYYYTFENVFEAFQIILVLRGLVNLYLILITTFYLTLTNWLCNRYTF
MHKY
LTTNITKPGYFLTVRKVIMTFKLAETIVQKTNHLFGSIIFTALALCVINILYYFVLALDLTSITI
EAKCI
NYIAPSIYAVFLLVLTMSCNSVETSGHSLVKTCYLLHESAELD

>PmacGR1
GIGTCLLFAMFCVFTSTLHFITVTQDNLGLSLKYLNLFYGVTVVQIFVFSVMVILSCDKVE
KKAEEFIKT
CIYIQASTGDENALALANLAKDLRPKFSAAGFFDINQRILPTFFSNLSTYLIIILQFKFSSL

>TcasGR170
MTKSTVYDTVSWLILLSLLLGLYPSYLQTVGKCQRLKTDKNCTIVIIFHLILFAVLLYFASLE
KNPLFDG
QLYSFNTFAKLLIFVVSLSGNSAVFIMLIISFMHKKSFKNFVNNVAALDETLAKLGQYINYE
TDYYVCLA
MTITGPLVIIGNIAMELWNMPRENIEPLPNVILACHMLSFLMVHQGETQFVAANVILKTRF
KTINNILEN
LWAKKLIVVKDKKKSRSDEQTIDICMRCHDQLCDMCGAINMLFGFPIIIGCLIQFNTIVFSF
CYCYYNSK
MTKDGVYNTLFFIVYVSLLLGPHPTYFGKTGRKTVLKTNKYCNFIILFYLSVFVILVYYAS
LDETPNPVT
NGKLYNFNTFAKLLILVVSLSGTFGFFGTHILSYINKASFKKIVNTVATFDETWAKLGLEINH
KEDFRVC
LVFTLTGPFFTFCNVLMEMWNIPRENIDPIPLVVVLTHLIPFMLIHQGETQFVVANIILRRRF
ALINSIL
RKLYKNKYRKLIVDGKKSEEQIVDICIRSHDKLCDVCDSVNRIFGFIIIIGCLIQFNTIVFAFC
YCYYSI
SIRPISTLGWFFWSLLRIYELARKAAFAHLNSNEARATLNWVSKLIIRSNPSLEEKLQIFALQ
LTHRAPT
FTALGLFPINGSFAFTVVGAATTYITIIYQFQVNKPTCGP

>TcasGR26

MYTVISPQLHYTNPNSFKMLRSITTNRSLFHTLSPLINPARILGLCPITYTKQKNFVTIRWSW
KIYMANC
ATALTLVCWGFWGFVHDMEIASFVSLGFTGSVDVVISSFDISDVLLSCLYFIVSMPFKCAKL
SIVFHNLN
KVDAIITPVFCDRFYSNLVWFSRCWFVFLPVLYTLDVFMWGNTSWLGVNNYFAYYVSYSI
VVLHELQYYQ
VVKMAQLRVSGINKTVKENIKKDTSRIKLEFIFDLIHCYNNTTDAIETINSSFNKTVTLMLF
SCYVHLVT
CPYQLFVMITSNETSILNYVYCLWVLLQIFRLVLVVEVCHNCEEEIQNTRILVSQLLNCRLD
KNVKKEAN
TFLFLMVKKKIKFSAYGLPKVGRHLLLSVASSIGGYWMILLQFSSRTSKI

>TcasGR27
MQVASFTALGFRGTADFVIACFDVNDVIVSAIFFVTSTPFKFKHFVQIVENFDRIDARISPIL
VEQIRKR
SNIFVKVLVTFLPTLYVLDLFMWGKNNWEGLNNYFAFYIMYSIVVVHELQYWHIMTMMY
ARILGLNKTLR
DYFKNKTGFCEHEILVVTQSFNSINDSVEEINKCFSYSTTTIIFSCYIHLVISPYQLFVVVSST
ETSLFN
YVYLLWISLHIMRVLTIVEVCQKCENENRKTRSLVYQLLLCKLNEKVKNMVRVLFFLVTT
RKILFSAYAL
PKINRRLIISILSSISTYWMILMQSTSRTIQVV

>TcasGR127
MVYITTNLRITQKLSIVTQLADLIFSASANASGVATVFFCLFYQNKLIEVISKLHKLDNKLK
HMLIWKSY
KRTQIFITCELFFVILLWISFFLNFMLHCNNTTWRCLYRWIVLYTLSKMSQVMLIQFCAFVV
VLKQKFCV
VNQYIKQVCKLNNGHYKNFLSQVEIIHNEILVTHNEIQTIFSVPLLMKIASQFVGIFCSLYFC
IFGYIYD
DEMVVPQNFHDIFLPLLCILTNTLEILITVTVCELTILEYKRTKKLLYRIPVTKTDSMLIRNIN
LFSLQL
AHQKLEFSACGFFLINGTLLHTIVGAVTVYLIMFIQFDIATTTKGG

>TcasGR30
MLLSRYRFVKRLLMEGRGIQVLRKCAYNLFVLKETTDIFNEIFGWPVLFLVLYTSLKLLYY
FESAINDVV
RVKTELIIVDISLIFIYVIGTFVIFVKCDDVLKEAEEIFYLLQKIKAKNKKLQDVIVTNVYVL
PKFSAAK
FFSLEKATIFKMLSSLITFVLVIFQLKFLMWDVFDEAHHRK

>TcasGR93
MTWFVVHLYVIVSEIRRRFELLNTKFEYIIRTTSSEAAAINCLKCHDLLCGLITKVNKTFGF
QIMIVDKL

IALRVVETSYLCLLFAIEERTKFSVLTFVNNFIWSIDFIICGALISFSCGKTNKQAQFSLILCQ
NLQNEV
GNDPLKGEILTQFAKQVVAARPEFNCLGFLPVNYGSFLKILGSIFNYIVIVLQFDH

>TcasGR98
MSYRLSRNDIRFLKVMYKLSHFLSITPNYDFENFVIISPRCDKISAICFLLSTILGTCWIIYVR
IYCKEI
RFFEISFEILGSLDSLILLMVLVSIILGSLKTKEWAKLNNKFQYIDEKLKTRDQKERNLFKNA
YFQLVLS
ISVYSSSVGYTQYVRIAAKGLPTLKLFALHEFYGFCYLWVLILICNIALAFKQRYQLINEQM
AVRINPKN
CASFVIEVRKLSRLLGEMVGLFNDIFGWPLVFITGRFVIKILVALNFFTSTLEIDNLYLKHKL
EISSLVQ
TAITLVAMCGLVLICDSAKSESQQTVFLCYKLMEKFPERSHEQQELYMAAQVIEKSVANFT
AAGFFDVKR
STLFGILATTTTYLIVTIQFNQGLNK

>TcasGR4
MDTTLNKNFSYPKHLKLLVTALTTLFLLFGIVVYFLKQMAFFSKLSETEGPISIELFYKHSG
KTIFWIIP
YNWITATIFTFLQFNSFIVFIFTDILLISISLILANRFQQLSKTLTKRQKIPQFHPDNASFWKNV
RKDYC
KLSSLLFLIDDHISLAIIFSYCLNFFSLLRFLTKLLRDSEQNLIVKFHDYCDALNFTLRIICLTL
FSSWI
NEASQEPVAILNSVVEREYSAEVGRLLLQIGFDEVALTGCKMFKLNKGLFLNIVSAIVTYEL
IVIQYNNN

>TcasGR166
MLPKQFLKLFKDPCDVYTAIHPLFYVCTFFGLAPYSLVRVENGKKVFKFAWWPLTRNALL
VLILLGALTY
HAIFDLISFKDSDLQQKLRYFEEVFSSLLSCCSVIFGCIFALKVIEVFKNIEEVDVAFRSLAV
WVPYKHL
YVNILIHLSGLVTIVATLTVTIIFFASYQYGTKTYSLFIVFMTVILPYFINLLMELQYCHYLNI
LRVRYQ
LLNEYLETLVQETNRTSVEGWTDVSNVKRKSKEISKLPKSMLAISDPVFIVDQVAALHIKL
TDTAHMINY
AFCVQQLLRITVAFISIVTALFLVAINFNKSSSEENEGKTTQLDYFFTFWAFSNACEVMAIV
WITSETCE
EANTCPRILHKIRNNTTNTNLQDTIEIYSLQMYHNRLYFTVCGLFPLDYTLLYTIVAGVTTY
LVILIQFN
NSDFVQRNSTEFDNATESY

>TcasGR3
MPKTHRSIPKSALNSNAAGWGCLPSSSAFSAGMKSGCGSAQSMGKRLDTHLGLVRALSR

RWPTKNTDTTH
AIQFSSALSWPQADASHPRPGCVTSRLSRTLLLLLLGSAQRRRQRRVWTTPRRPKAAGEEL
GVVSTINSS
TMYHQDQAVSILGEAIPKRRSVFLESGVNSADSFKASKVGPAPPIKFINKSSTDKFGNGAIY
EVLKPIYA
LMRIVGIFPIKNTEPGMFRVAPELLGYSVVVFVVVMGYIGFIEWDKVEIVRSQEGRFEEAVI
DYLFTVYL
LPIIINPLVLYEARKLANVVTDWVNFERIYYKLTKKKLSVFFGNKPVILTVVLPLLACGVM
VVTHITMAH
FKIIQVVPYCYINCLIYLIGGFWFMQCDVVGKVASQLAEDFQMALKHVGPSSQVADYRSL
WMLLSKLIRD
VGNASGYTVTFLCLYLFLIITLTIYGLLSQLQAGFSTKDIGLTINAGLAIFILYFICDEAHYAS
NCLRVQ
FQKKLLLVELSWMNDEAQQEINMFLKATEMSPTDISLVGFFDVNRNLFKSLLATMVTYLV
VLLQFQISIP
EEASPTNSTTITTQTPN

>TcasGR8
MKKWTQVDEAMSGYGFPPKLERKLRIIFAITVVASLVEHGLFIGVEYMSCRGNNLSEALD
RFLMFHYDYV
FALVPYHVVLGIILEIVNIFSTISWTFMDLFIILVSLSLSARFKQVAKYIKFLVERNVLNKNSW
QRARQD
YTRLTNLCKDLDEVMSSTILLSFGNNIFIILVHLYNSLQKPLEFGYLDEIYYLYSFICLLVRIS
AVALHA
ATINTESKRPIYLLSTIPHHRYNLEIDRLLLYTKYETAALTGYKLFRITRTLILKITLAIVIYEL
VLVEY
LKVESY

>TcasGR52
MYIFVVLGLLTCGLISTTITNNFYLGDIYMKIVVAYLTELNLYIFSLYVLVVVNLRQRKRWY
LLMRHLEI
IKIKIKVPKKKGWKLPYFSKFWGFCFLTLLCEALVDFRWAFLFGWRYLNKFNVRLVQIFFT
NYYKFMLCV
VLNTFRVCYQKIRHQKTLNETVQIFVQLRQTIDVFNEIFQFPIALMVAFTTFEVLNYILFTLY
VKIEVSV
FESIVLASLRLGPTVVWTTVLLLLCDYVRQEGEKIVTLFYEMRLQNYELFELTEYVEENLP
VLTAGYFRL
GKPTILRMFATVANFVIVVVQVTNKN

>TcasGR45
MRLKWYYITISMAFIIFVSLEIYHIYTVVEESGFENFVKTYLVSDFENYVTFFCSLFIVVILK
LILAKYR
QQDVTLLQITKAKNLLYSKQIVTILKKTKSNIFILKESVDTFNDIFGWIILCNIFEAAAKSLIY
IDMIIK

KNVTQQNSDVFIFDNVWLFILWVGILSTIFLCDTILKKVDDILSQAYKLEASFDDLATYETD
EVQIFIDV
VQHNRPEFKAARFFSIDRSTLFSVLNSLTTFLLVMIQFKEN

>TcasGR153
MTIITRSNNVVGGKFYKKRNKVSNFGLTFEFIFGIIILYTTYSMSFVKRNHVRDVVLQLNRI
DELLAKMK
QKFRYTRAVWYQLIIFFSGFLMILIIASMQIQNIRIENFPPLSLFMCIIFLFPLVILYDLNSQYG
FTAIL
IYERCFIKCKTTQKHTFRYFKQNNDFDGNPRNLMQGGKHHKLKLQHSDATSHNARIFHQS
LLNFLLLLDG
NVTVVDTILNLQIFQHSSKPFSASCWLAWGFLKSYEILHVTISCHLASQQANIVGRKVHKV
LIRTQNDEI
EEKLLMFSKQVQHSSFKFTLCGLFNIDAGLLFNMIGSSTTFIVIMIQFQETITPTICVSNKKA
MF

>TcasGR125
MKKLNYTVEISYDLEEGVKTAHCTCPRGNVACHHMAAALYYAHYNVSATDIECQWSAPS
KTTPQTEVIKL
ADVYKPKLSNYTALSRSSTEDEIIQFRAEIGVTNVVGFTWLLRPEASEEARKIIADIEEILQS
LEYVQAI
DKQKFLLEKCRIDEARIKLVEACTRGQHVNENWHVARKHRLTASRFGMVLSACSRRRFPP
SLFKNLAEGY
SLDRVAAVQWGKTHEKTALREFEEATNLKVQETGFWLEESGFLGASPDGLVEEDGILEIKC
PYKYRDTDS
LSEALKDKKYFYWRDENEDINLNSNHNYYHQVQGQMHITGRINVKSSSNMIKFSKVKSY
FTTHHDTLHNI
YTSMKPVYAICKLIGLNTLRIGRKGELKQHKSDYFYFSFYITSYTLLSVYSLFRIATNENNSL
VINKRLI
FIECFVMMALTLIVTLFTFLARGTLIKSFDMLSHVDVSFIKAGFRLEYKQLLKRSYLIISFVL
FSLLARV
PVMLMTISADFIQQIMLFVSALIKAFSKYQFVVLVLQLQHRFGKINRTMRSFFSDNKQDDK
IPQISDNLY
ILCRLHYKLTSVMQKINSAFSVQLLVSIGVSLFDVLFQAYYLYYVATGKASFVTVPMIVCPI
VWLMDEVV
EIYLLVYACASTCEQANDTPSILHELRNNYFHMDLENNVQSYSLQLLHQKVQFSVLGFFV
VDYTLLYSIV
GAVTTYLVIFIQFDQSSNSRNNYVLTNNSTC

>TcasGR1
MRNDHGSNTHLHPDDAIRRAKIVKVAASPTSANPDEEPDPELLDRYDNFYQTTKSLLVLF
QIMGVMPIER
SGKGRTTFRWLSSTSIYAYFIFGAETIFVTMVFKERLYLILRPGKRFDEYIYGIIFLSILIPHFL
LPVAA

WTNGTEVAKFKNMWTRFQLKYYQVTGTPIIFHNLTLITYSLCVISWAVGIGIMLAQYYLQ
ADMLLWHTFG
YYHILAMLNCLCSLWFINCTAKGRVAVWMCNNLHKALESRNPAKILGAYRDLWVDLSHM
MQQLGKAYSGM
YSMYCLLILLTTIVASYGSVTEIMDQGISFKEAGLFMIAFYCMTLLYIICNEGHHATRKMGP
EFRERLLN
VNLSAVDQKTRQEVHMFLMAIEKNPPIMNLNGYANVNRKLISSNLNERNCFSNIVKLRTL
SPTQT

>TcasGR129
MTLLPTTPHYLKFFYQLTGFVQFSYNESLESLLSRLWCLPFYLYSLYLCFYFNSSDAYSGTG
IFFYISIF
SNSVNVFSIFTTFWICYTRSTSLRQILSQISQIQVDVKQTSENNWHTFCIIGLLMANIATLLV
VNTPKNF
ITYLYMLPLVINCAINLLIRDVLNLVFCKFESVNYQLGRQVTSKNLKEIFPSTKINRIEVLQD
IEVDNDM
KLVENLSHIHYNLVKLSFMVCENFEITIIANLILWFEKIIETVYYAVYISINETINNLVVDYVI
NLLFVA
YSFYWLFTLIGHFCRVEREANKTATIVHDIWNKVAVERKFDKRIRHLQLVSVKLLNTKLKF
TARNFFTLD
WTCCHMVKGKI

>TcasGR132
MNQFVEPVTLLIFYYIVPITIKCFDNLFKRDILNLLLVKFKFLNKEFDRQSDIADLPKLFPLT
KTKKIKF
LENEEIDSNLRYIEELSQYHYHLVQLSLDICKIFEIEVVITLILWFEKIIESTYFAIFTLVNDDY
NHKTL
FHALDVIHATFEFYWLFTSIENFNRVQIEANKTPILAHDVWNKYVTKKFDDKVRHLQLISV
RFLNTKLQF
TAAGLFNLDRSFCHTVIFGVTVYVVILIQFYIYI

>TcasGR144
MEVRSNKIAKINFNFLLQSFENSAIYVNIIKPIAKYSLYLGISPFTTKNGQIVLSRIRLVPSTIL
VLTNI
ALTIIYSTSFGETSDFTKAETIKFLTMFRDLGSTTMMVLNLFFSQMYFTKKLEQMRQVHEV
DNQLKQMGC
DLDIPNSVVQKHLILLMVLVNLIFNTCGEVFSAWVRCQDQILIFLVNWYPRLIIGIMNSTLN
LIFLLIQT
RFEMINNIITRGDVTSTTIKKLFNLHKILVKVVREINGIFAFQVLMCTSMNFVLLIGDLHTSI
YIIFFDM
FYQHHKIVLDMGKNCVTYVFDLFYLSKRASDLCNEANKTKILLVGLKIDIDQEEERNVVV
TSVLKLMQNK
LEITACRLFSIDNALLFSICGAASSYLFIMLQLDIGSKTQGTNSTLY

>TcasGR149
MKSIRVVLAIHSAGMFVLMVLIIVFVFINWAKVRHQVTQLVLIDSELKKHYQIHNETKLIL
VLALELVVF
NILSGVYMAAIKPNNRVVFFVIITLPRIVVSHMFVIFTTIVVVLYQNFKIVNSMVTSDPSRIR
HVLLLHK
SLTRIGTTINSLFSLQLLVFITINFVVLLGDLYVTLYIILTNNQAKYCTTLIGLIKNCVIVIFELY
YLSH
VCQNVSTEANRTKFILVSTRIDISDEKKRNLVIGNILRLMHRRFEITALRLFTIDNKLLFGIFG
SVASYL
FIVLQLEVVPT

>TcasGR21
MTITISKELFHVLSPVLYLSRFFCLQPLKWTKTSAGNYIITKSRFYTIYTLAASCLLVITSITG
LSQVYQ
LDVIYLVRLGDTTRRFVTYSDIVVVLLPCVIGPVFALFKTNQTINYLSHLKQFDSLQNQPTK
STKIFQIT
ALTTFCTAFTLSMDLFLWLKLSHNYIFLLCLPYYISYWSTVVIELLFWHFVHLIQIRISVINK
KLAKMVV
TGLNSVTTLKKPHAEVEDLVKGYEKLIEATNSINYCYGFPILVIILGCLIHLLVTPYGLYSIIM
STGDST
SILSQTVWMTAHILRLFLIIEPCHECFIKTKETSQLICKLLCLSVNQEVKKSLEFFLTYLGEC
KIEFSVY
GFTKINRELLTTIAGAITTYLVILFQFK

>TcasGR205
MSFAATHIILELRTEKVTKVAVKLVQIAVTLDILVVVYKSRNKFVDCMNSVLYYEEQAEKN
NIVMRYRYS
LRILLLMKKVMVLSTSFIGNLSCFMTGNTYISCVVSFITYNVTYLISSIVLTQFCGLILVARQ
HLIFVNR
ALFNLSKDPQQAIEKMNELMTYHFEVCNMTIKINQAFSAHLLLIIVRTFVEFFNSLHSIVKE
KSHLLVMY
DFFWSTCAIFEFIFMLFACKRATE

>TcasGR89
MKRYKELTSKKFVKILQTISAITALSVPDLFTYGGLPVIYILYSILIAFTLVALSVYTLYFKVI
YFYKNL
LATVSILDSVTIASVTLTNVLSIFFAVIFRRRKIVSLVKNLQEIERVLNEKFQVSCEINEKSLLI
VFGLL
ETFLFSYIAFDCVSQIRTHGFFLYSLSVVMHLNVFLVSITVSQVQLFSTCIKQYFSVVNEQLA
TTKVATI
KMKFFLQTYDKLCDIVDLVSDSYGIQIACVTFIIIICLIESLNLLMKCALRVQTLGEVWPITL
LIADTWN
SIIYILFGIILSICCQQVSNEANNTSVLSYKILLNFPSRPKSQNDEINKSELLLLAEHLSQRRV
KFSAAG

LFDVDHTMLYMIFGSIAAYLVIVLQFK

>TcasGR91
MIEVGKTRVKRASNQLFRFGQFCAICVPKITSTNNEVPRWYICYTIGVIITIGCLCIWSIFNK
WNQFRSY
SNFSVFLFDIITIVTLTSTSILLIVNAVFVKEKRIRTLLIKLSEIKENTRLAEKQARISILELVAV
HLVL
AFLYSFDLFANGITFGWHTYIFDVPNFVNEYVQAIEVLYICSYTVLIRNKISNLNTSLIHSTH
LNGDIVK
EHQNLHNKICNLIDEFNECFGLQILGILFVGVIYTTYTIFLLLIFGSGSATPENNVSVVYVLIL
YILESL
LYMIFGIMITKACMSASNLIQRTPNICYKLLSNLGIFPKTTNEQLLRKELLLFAEQMGQRQV
AFKVGGFL
NIDYGTLYSLFGSTAMNVIILLQFQKQGKYN

>TcasGR61
MSLKLIKLVFKVGGLLAITPAKMGKRGLFFPSKAYALLWIILLTAAVVVSAVYKKASYENL
TSVLLTLSV
ATDIILFVFNLCTIMTTVTKRHQWIKFIKILKTLQSNNNEHLFYLLPFLITNVIFVINFGYETY
LWSQII
GAEFYKTYAVEYFQCYSQFIVYFLVYAFLKLILDNVRNISRNINRLKMQPNRLNNCALKAL
KTDFLALAE
CTDIFSDIFGSLILLLMGFCILQQLTYLHSLVVKSSRDTISILVYKIMFITWHMVGTFISFFLC
DMIEQG
VRHVEMVAHQIETNRVEEETEALEMLVDTIKHNFRYFTAARFFNLNRKTFLGTVNALFTFL
IVGIQFENF
KI

>TcasGR64
MSLKLIKLVFKVGGLLAITPAKMGKRGLFFPSKAYALLWIILLTAAVVVSAVYKKASYENL
TSVLLTLSV
ATDIILFVFNLCTIMTTVTKRHQWIKFFKILKTLQSNNNEHLFYLLPFLITNVIFVINFGYET
YLWSQIM
GAEFYKTYAVEYFQCYSQFIVYFLVYAFLKLILDNVRNISRNINRLKMQPNRLNNCALKAL
KTDFLALAE
CIDIFSDIFGSLILLLMGFCILQQLTYLHSLVVKSSRDTISILVYKIMFITWHMVGTFISFFLCD
MIEQG
VRHVEMVAHQIETICVEEETYFTAARFFNLNRKTFLGTVNALFTFLIVGIQFENFKI

>TcasGR160
MSARNIITSLYPCYISQLLIGCAPIKITEIRSNLQIKHRRVYDYSTILILLLIISFIYYRYSLMDF
YDFN
SSALVKFMTTVYFVIVSGLISTNVIYSRVQRQVMTQFLYKIYTVDKKMRNIGIKLKYENIQ
YFSTKVLLT

GFATFTIYLICLLTTQGFQNRIQGLITYGLLYLAVLHYISSTCSFMTTIQIITNMAENLMKKTE
DLLIYK
SFYRQIPLREMMKLHQEIYDLLQLSTKIFAAQVLFSFSVAFVIITFQSFSIICAVYNNSPYLRY
SIASAI
WIVVFVTEKMVLTFSCHKCMFRSIELRQKLGKYAVAHSKNKLLVKEINAFGLQLLHEPLHI
YAGSMFLID
LPIFTSVCGTATTYIILLLQTDHKVDLIYSMKKVFGVF

>TcasGR72
MATKLASFIFRIGSILAVTPSYNTGEMTFVRKLYGLLFATIMTISIVVSTINRDFYRSIPTVPAI
ERTIL
DINMALLNYYLIFSVMFWKRIQWKDLIKKIHVLVDKNCFPFAVIFLLIQIFDLVFLITAFCFK
LDLMGLE
YVKKYNFVYVQDYMVFFFNSIMSFILSIVLLKYKQLYNSLLHTYRIQTIEKVQKNLRFLKV
VNESFNDIF
EWPIFLIISYTTLHLLCHFDNIFMTSMSHKYGSVPTKKIVADLSILFFCFFGTAGMILLCDAV
LIEAEKV
LTVSYKLTEFYEEKLYLTRVVLENFPSFSAAKFFNVNRSTLLKVLTSVIGFLIVFVQIRQV

>TcasGR23
MTITISKTLFHVLSPVLHLSRLFCLLSVKWTKTSCGNYKITKSRFYTIYTLATSCLLVTTSIY
GLSQVYQ
LEVVYLIRLGDTTRRFVTFSDIVVVLSPCVTGPAFALFKTNQTITYLSHLKQFDSLFNQPKK
SPKIFFTT
ALAIFCTAFILSTDIVLWLKLSRNYIFLLCLPYYISYWSTMAIELLFWHFVHLIRIRIGVMNE
KLAEMVV
TGLNSETTLKKLQDLVKGYEKLIGATNSVNDCYGFPILVIILGCLVHLLVTPYDVYSIIMST
GDSTSILS
QTVWMTAHILRLFLIIEPCHGCFVKAKETSQLVCKLLCLSVNQEVRKSLEFFLMYLGECKI
EFSAYGFTK
INRGLLTTIAGAITTYLVVLFQFNKNG

>TcasGR190
MWSPNDVSEILVVFCKIWTCFGLPVYKFNVQGNFCLEKSLTFFVLNLSILTVGLLLHVVFIP
VANNLWSH
ISFCQYCIHYVQTFGIVIFSHVKRKEFEEIFNDLLAIESKIRLFGGGSFGYHKLGKELVVFVI
QQSLLVT
ILAIANFFTTAETTEIVLYLLGYYFGYLSNFHFGFFLLIMPKIGFKLYCDFINSINGQPVKNV
MKMYGKL
YSLPVSLVKTLQGFILLRVFSTFTIATLDIFFAISLVIPQELPWFKTFCLIFVNILWLSGLLLNE
FLILL
YFEKMLEQKLVICRLIRNNRPVRYKYLDLIYLRFNHEPRHFTVCGLFPLKCTLIFSVVAGVA
TQVTYLFQ
FVNKLEQ

>AchiIR1
MFVVARFSPYEWHNPHPCEIDNDLVKNQFSLANSFWFTIGTLMQQGSDLNPKATSTRIVG
GIWWFFTLII
ISSYTANLAAFLTVERMITPIENAEDLAGQTEIPYGTLESGSTMTFFRDSMIETYKKMWRF
MENRKPSVF
VSTYEEGIQRVLQGNYAFLMESTMLDFIVQRDCNLTQIGGLLDSKGYGIATPMGSPWRDKI
SLAILELQE
KGEIQMLYDKWWKNTGETCTRNEKGKESKANSLGVDNIGGVFVVLLCGLAFAVIIAIFEF
CYNSKKNALT
EKRSASAPHQSLCSEMGGELCFALRCRGSRQRPALRRQCSKCLPGVTYVPALLDIPPHPPE
PPTRVPPSN
GLSPGLSPCDRPRLYREDHMFDPDRQRPHPTAHFDN
>AchiIR2
MKMPSNSASLVSVLLVYLSFGYCHTNVFKIGAIFEDVKSGDGALVLKEAFTTAISDINNSE
DYDFKFLPI
IEIIGDNPYEAMNTTCTLLEKGVMAIFGPSLEDNSNVVQSITDAKEIPHIEVRWDDHPQNG
TIVNIHPYP
DVLTRTYLDIIEAWGWRDFVILYENNESLQRVGELLKMFNPSKHRIVVRQLKDEFRTVLKE
LWRSGATHF
VLDCTTDILPEVLNKAQQVGLMTNREHYIITNLDFHTLELTRYRYSETNITGMLFVDPDDK
RLIKAAAKI
YNMEEGMEFAAGWKLRLEEALLYDAVKMFAMAVKEAPQLKEYPFDVSCHNNQMFSEGT
TIINYMKNFDYT
GLTGRINKFDNEGFRKNFALDVIELMEGGITKVGVWNYSSHVQPLTITRIVNESTEDFSDIR
NRTFIVAI
TLTQPYGMEKESTENLQGNDRYEGFAVDLIQELAAMRGFNYTIVVKDGKVGKFNNATGK
YTGLIGDLHDR
KIDLVVTDLTITYEREEAVDFTSPFMMLGISILYQKPTKAPPGFFSFADPFAFEVWRLLVLAC
VGVSFVL
FVVGRISSSEWENPYPCIEEPEYLVNQLDLRNCAWFVTGSIMQQGSEIELKSVATRMVAGM
WWFFTLLMV
SSYTANLAAFLTTESPDPHFNNLRELVNNAEEKGIKYGAKLDGATANFIRDKADTDKDYA
KAWEFMKKHD
DVLVNETKDGVDRAAKEKYAFFMESSSIEYEIQRRCNLNQVNGLLDEKGYGIAMRKNES
YRNSLSTGILI
LQNSGKLEEIKRTWWEQRKGGGQCLDKSSEAEATSLNLVNVGGVFGVTIAGTILALLMA
MLETVLHAGKK
IS
>AchiIR3
MKALDEAIQDEYDIASQVSDEEWEAIRPTKLERRNYLLDRIKTYLAKHGTCENCTFWRM
STGETWGKEYQ
LANYFNNSEIQSVIEILPVGTWRPSDGPSMNDELFLHVAHGFRGKTLPVVSFHNPPWQILKI
NESGEVTE

YTGLVFDIIKELSNNLNFTFTVEVVNKTVIKNSSVGNNSLEITNIATNSIPTEFIDMIRNKSVA
FGACAF
TVTEDNKHLINFTSPISIQTYTFLVARPKELSRALLFISPFTGDTWLCLSAAIISMGPILYYIHR
FSPVY
EYKGIPMKGGLSSVQNCIWYMYGALLQQGGMHLPYADSARIIVGSWWLVVLVVATTYCG
NLVAFLTFPKI
DLPITTIDELIGHKDTVTWSFRRGSFLDMQLKTSTERRFRTLYDDAMKEHNISNLIKQIENG
KHVFIDWK
MRLQYIMKKQYLETDRCNLALGLDEFFDEQLALIVAQDIPYLPTINAEIKKLHQVGLIQKW
LQDYLPKKD
RCWKNRHIIEVNNHTVNMDDMQGSFFVLFFGFLVSILLLLLEKLWHKHAVKKKEKIIQPF
MS
>AchiIR4
MDLIQEVANIIGFRFEFHLAEDKEYGKWDEKKKKWTGLVGDLLERKAQLAVCDLTITHQR
REVVDFSMPF
MTLGISILYKKEEKKDINMFAFLDPFSVSVWIYTATLYLIISVVLYFISRMTPGDWE
>AchiIR5
MKNCLWLTLASVMTQGCDILPKGTTSRIAVTMWWFFSLILVSSYIANLTVFLSIDRTEPVID
GAEALAGQ
SKVKYGVVNGGSTMAFFRDSNFSTYQRMWVNMDSSGPSVFESNNLAGMRRVQTSQGKY
AFIMESTQIEY

>AglaIR25a
MDDKMLLRIKLKRKKIWECILLKEEGQERIVEWYNKGISTLNTNLRFETTFIDIIDEKADV
DNTQYVLMN
HVILLTVHLFSYSSLSCPVCNAISIGVTLILDITWSSNDVARNLASDVGIPYVEVDVSISPALL
LLDKYL
DFRNSTDVSLIFDNPQYVDQALYYWIDSTRLRMMILETLDKITAKKLREIRPYPNNFALVA
NTQNMNRLL
KIVLDEELVTRPDRWNLVFLDFNYDNFEEKLMYNKSINLLTLDKNMCCNLLNRKDFCYCP
DSFNLQEEFF
KKVLSVIVKSLEALVKDGQKHTNDIRCNATAYDNQTKLSFEEIFENKISRNQLYKNNSIVSV
KVEGTIKI
GRNESYEVVLRYLNDVMELQQGKTVNPIRPFYRIGITHAIPWSYKEQDPETEEWYWTGY
CVDFAKKIAEE
LDFDFEFVEPKNGDFGKKVNGTWNGVIGDLASGQTDLAVAAIIMTADREEVVDFVAPYYE
QTGITIVIRK
PVRKTSLFKFMTVLKLEVWLSIVAALIVTGFMVWFLDKYSPYSARNNKKAYPYPCREFTL
KESFWFALTS
FTPQGGGEAPKALSGRTLVAAYWLFVVLMLATFTANLAAFLTVERMQAPVQSLEQLARQS
RINYTVVKNS
NTHQYFINMKFAEDTLYRMWKELTLNASTDDTRYRVWDYPIKEQYGHILLAINDSNPVAT
PEAGFRNVNE
HLDADYAFIHDSSEIKYEISRNCNLTEVGEVFAEKPYAVAVQQGSHLQDDLSKTILILQKQR

FFEDLRTK
YWNHSSKGQCPSTDDNEGITLESLGGVFIATLFGLALAMITLVGEVFYYRRKGKSNAIKSN
KAHVFPEKQ
LKPVPPRLINASTYPVTIGTTFKPVNAKQKGEKDDLRLSHITLYPRARNTITKINN
>AglaIR40a
MQRLSASGISSTLFNLTSPDVQEKYFDFLNYHTEYHLEAYTIFFANHHLYEHILLEIYDRNYI
RRNLVYI
FNWGRLPLERNFVRNIEYAMKIYVITNPRTDTFRLFYSQATSHREHHLDMVNWWNHGKG
LFSHPTLPIMK
SVYKDFKGKVLQVPVLHKPPWYFVRYNNSNNGNFSNINNTMEVLGGRDDRILSLLSQKL
NFRYNYFDPPE
HIQGTPDSESGVFSGVLGLIWRREADLFLGDVALTYERSNFVEFSFLTLADSGAFVTHAPS
KLNEALALL
RPFHWQVWPAILVTCLAVGPMLYIIIALPNAWQPRFRVRSHARLFFDCTWFTITILLKQTGR
LPSSSHKA
RFFIILLSISATYVITDMYSANLTSLLARPGRERAVNNLYQLEKAMESRGYSLFVESHSSSY
GLLENGTG
IYGNLWDLMKRNQGGNVLVQSVEEGVKLVRDSDKIAVMAGRETLYFDIQRFGPSNFHLSE
KLNTAYSAIA
LQLGCPYIEEINRILMATFEAGIITKMTENEYEKLGKQKEISMEVEENLVSEAERENPRKTK
ANEDSEKL
KPISIKMLQGTFYLLCLGNAFSGLILLAEILFYKHQKKYRVKKKRLVAMRKFSKFVRFQIN
KLRLTIRRL
YRNVMHDAFVATLEYIE
>AglaIR21a
MVDQQAYLVKLLKKVTNDYLSECTPVILYDTFTELYDNLLLEKLLTNFPFAYIHGQITDNY
ELKVKTTLD
KKAQSSCVSYILFMKDVMRSKDVIGEQNCNKVIVVARSSQWRVIEFLSHKESQNFVNLLVI
VKSGQVVPQ
NEESPYILYTHKLYVDAIGSSKPDVLTTWMKGKFTRHVNLFPKKMVYGFSGHRFLVAVSH
QPPFIVNKGR
NENGDNIYEGIELRLIEMLAKRFNFTTDYKEAPDADVMGSGEAVAQMVQKRKANLGLGG
LYITQDRIDRI
AISQWHSQDCAVFISLTSTALPRYRAIMGPFHWTVWLAVTIIYMLAIFPLTFSYKLTLKGLIT
NPEEMEN
MFWYVFGTFTNCFTFSGTEAWNKSDKFTTRILIGFYWLFSIIITACYTGSIIAFVTIPIYPAVV
DTVRQL
LDGRYQIGTLDKGGWPHLFANSSDEGTEKLLRHLDLVPDVESGLRNTTKSFIWNYAFLGS
RAQLDYIVRT
NFTTKSKRSILHISEDCLAPFNIGVGYPKNSIYSEILDVGILLALQAGILNKLKADVEWDM
MRTATGRLL
AASSKVGGIKALSYEDRALNLDDTQGMFLLLGAGFLVGAGVLMSEWLGGCFMFCKGKK
RNDSVSSIESNP
RSYEGRTPREKLNSIQYVRMRNRLDSTIDLEQNFDYEVPRAKRVQMKKQESNGENHVNC

VVHEVADSHSS
GGSKDSDDDDFNEEINKIFEEVFGEKNVDSDSSHEEVTDTCDKENVIETTT
>AglaIR93a
MCRVMFVGIILYICVRTKAENFPSLITSNASLAIIIDREYVGDEYENIKADIENYLTYAKREL
LKHGGVN
HYFYSWTAINVRRDLSAVLSIASCSDTWKLFRSAEDEKLLHMAISETDCPRLPSNKAITIPLI
KKGEEMP
QLILDLRSAGIYNWKSVVIIYDRTLDRDMTTRVIKSITQQSNLNIKATGISLIKLERNITKYH
LNEIMST
IRTKTVGGNFLVIVSHQLVETIMEYAKSLNLVDIKNQWLYIICDSIYNNSDITSYKRLLREGD
NVAFVYN
TSITSENCRVGKKCHSEEVLEGFMKALDEAIQDEYDIASQVSDEEWEAIRPTKLERRNYLL
DRIKKHLAR
HGTCDNCTFWRMSTGETWGKEYQLANYFKNSEIQSVIEILPVGTWRPSDGPSMNDELFL
HVAHGFRGKTL
PVVSFHNPPWQILKINESGEVTEYTGLVFDIIKELSNNLNFTFTVEVVNKTVIKNSSVGNNS
LEITNIAT
NSIPTEFIDMIRNKSVAFGACAFTVTEDNKHLINFTSPISIQTYTFLVARPKELSRALLFISPFT
GDTWL
CLSAAIISMGPILYYIHRFSPVYEYKGISMKGGLSSVQNCIWYMYGALLQQGGMHLPYAD
SARIIVGSWW
LVVLVVATTYCGNLVAFLTFPKIDQPITTIDELIGHKDTVTWSFRRGSFLDMQLKTSTERRFR
TLYDDAV
KEHNISNLIKQIENGKHVFIDWKMRLQYIMKKQYLETDRCNLALGLDEFFDEQLALIVAQ
DIPYLPTINA
EIKKLHQVGLIQKWLQDYLPKKDRCWKNRHIIEVNNHTVNMDDMQGSFFVLFFGFLVSIL
LLLLEKLWHK
HAVKKKEKIIQPFMS

>AmelIR25a
MRFVEFTIATCWLLGLCGRFVIIYGQRSVASNRESGKTRSVNIFIINDEANKVANQSITTALE
TIKENYP
NHLGNVWSVQVNESDINNTLDRVCNNWDSAVEKGGAEVPDLVIDTTTAGLAAKISNSFTA
ALGIPTLSAQ
YGQVGDLQYWRKLSLDQQDYLIQVMPPTDLIPEVIRQLSIQLNITNAAILYDYNFVMDHK
YKSLLLNVPT
RHVINETSQQIIEMKRQLLRLRDLDIVNYFILGNENTISIALEAADALNFTDKKYGWFLLTP
DINIWPRC
ECRDISVLFMKPEFDRRNNSDSVEFSLPKPILLSAFYYDMIRLAVLAMKSALDDGEWPMEP
RHITCDEYN
NTNTPERKLNFFGKLKDAYKNMTPTYAGIKWGSRNGEHQAKFVMSVHLVTIKDGVVSNT
VDSGSWNASIS
SPLQLTNNDVMNTTAVKSYRVVTVIHPPFVMYNEEKNEYYGFCIDLLNEIKKTVGFQYEIR
ETDDKKYGS

LNLDGSWDGMMRELIEKRADIALGSLWVTAERERVVDFTVPYYDLVGLSIMMLKTKTTT
SLFKFLTVLEN
EVWFCILAAYLFTSVLLWIFDRWSPYSYQNNREKYKNDDEKREFNLRECFWFCMTSLTPQ
GGGEAPKNLS
GRLVAATWWLFGFIIIASYTANLAAFLTVSRLEIPIETLEDLSKQYKIQYAPVINSSAYIYFKR
MANIEW
KFYEIWKEMSLNDSLSDVERANLAVWDYPVSDKYTKMLQAMEEAGFPASTEEALRRVRR
LDSNNEFAYIE
DSTTIKYLTMTNCDLIQVGEDFSRKPYAIAVQRGSPLKDQFNNAILILLNKRKLEKLKDKW
WKKNPNKKD
CDAENSQSDGISIHNIGGVFVVIFLGIIFACFTLAFEYWYYRHRTKITKIDRNNAMKNKITQ
VKPLRFNL
QPAPTHAFQSVHFRPRF
>AmelIR93a
MHYSQVSSVIYCANNTRGYFSLSIEMISVLLLVWWINYGSSYNNFPSLITSNATMAVIIDKG
FFSNKDEY
QNATKVIQDLITDAVKKEMNLGSISIRVFRDMNVNFKDYTILLSVATCYLTWRLHEVAQKE
ELTHFAITD
PDCPRIPDTDGITVPSIVPGEELSQIFLDLRMTDILSWNVINILHDDTFGDKATSSNDNVTIL
LSNANTC
SRLVSDRDTISRVLKAISNKLPNKRMNLISRSIFSLRYGNTGSGRKSSVKKMLNDFHVEQL
GHCFLVIAT
VDMVADVMSVANSLNMVHPGSQWLYVITNSVSGNLINTSFINLLAEGGNVAFMYNATNL
DGFYKIKLKCY
IKDLIEALAKALEYSLKNEIELFKRMNEDEFEMIRLTKSKKRAELLKNVRIHLSRNTSASNS
VCEQCLLW
RFFSSITWGNFFSHDRNMAHLLDIGTWTPIIGVNLTDVIFPHIVHGFRGINLPIATYHNPPWQ
IISMSKT
GKKLYEGLIFDAINYLSMKLNFTYTVIMPETSQISRSWNTSQFAKLGEKIKEMTMSTTKKV
PLEIIDLVR
QKKVLLAACALTVNECGNTTFNYTVPIFVQTYSFLTAKPSQLSRVLLFASPFTKETWACLA
VSIIIMGPI
LYLIHKYSPYSTKASGLNSSWQCVWYVYGALLQQGGMYLPQNDSARILIGMWWLVVMV
LVATYSGSLVAF
LTFPRMDTSILSVEDLIAHKDSISWGFPNGSFLEMYLQNAEEPKYHVLFSRAERHNDTEEE
RLVERVKEG
KHALIDWRSSLRFLMRKDFLLTGSCHFSLSMDEFLDEPIAMIIPYGSPYLSVINAELHRMLE
SGLMNKWI
TEKMPMKDKCWEAPGSNQMVNKRKVNVTDMQGIFFVLFIGITLAFFFLFCEFYCHRRKIA
KERKLIHPFV
S
>AmelIR21a
MLLVTLFLQFIVLASSKRVLYKLHQCENNEANLKSLAEEIVEEIIEQTNCIIFITDSTYQNLID
IKNIKG

SSNVSKYEILLRDNEQFSRPRRRIQRILVDGRTVDCNAYIMLISNGYLTAEFLQYTERERLIN
TRGLFLL
LYDLRLFQLNLYYLWKKIINVVFIRQYNAYKHRSGEISFKERIDLNTVYFPPRKRRLTATKY
IDTWYQGK
LRYGTNHFTEKTNNLQKKHLQIAVFEHIPAVTEKSKLYYNKQPNNIIQGLGIEFELIQIISKA
MNFKPKY
YIQQNIPLKQKDIEGSNQTDTGLISKVIEENAAFYLGDLHYTLQNLNYLDLTIPYNIECLTFL
TPESLTE
NSWKLLILPFKFYTWIALILTLILGSIVFYFLSLSYKKHISSYKSQNTSIKNETKGLYLFTEIG
NSILYT
YSMLFQVSLPHLPSPWAVRILIGWWWIYSILVAVAYRASMTATLANPVARVTIDTLAQLAKS
SMEVGGLN
EESKNFFLKSSDLSSQEIGNKFIIIKHEDEAIEKVANGSFCYYENSYFLQYARVKRQIFEKEK
KRNETAN
NRSSKHNLHIMEECIINMPIALGMEKNSPLKPKVDILIRRMIEIGLVKKWLNDVMEWPKIM
EIRQEAESE
KALVNLHKLKGAFFAIIFGYLLAFMILIGEILYWKYIVLKDPKFDKYHLDIFYNSNNNSKI
>AmelIR NMDA1
MKYESIALFISLIILNDEIYNIIASPQLNNPTLFMIGGVFSNNKSKKYFEQTLNELNFNLNYV
NKGVTYK
HTIIEMDSNPIKTALSVCKSLIERQVYAVVVSHPLTGDLSPAAVSYTSGFYHIPVIGISSRDSA
FSDKNI
HVSFLRTVPPYSHQTDVWVELLKHFNYMKVIFIHSSDTDGRALLGRFQTTSQNLEDDVEI
KVQVESVIEF
EPGLDSFTQQLIEMKNAQARVYLLYASKMDANVIFQDAAVMNMTGAGYVWIVTEQALD
ASNAPEGLLGLK
LINAENETAHIKDSLIVLTSALQEMNKSKSITEPPKNCADSGSIWETGKNLFEFIRKQVLSGS
TGKVAFD
DNGDRIFAEYDIINIQENGDQVSVGRYFYPNGTEKMTLSVNESNITWPGRLQTKPEGFMIP
THLKVLTIE
EKPFVYYVREIAFSESCLPEEILCPHFNVTDGETTKTFCCKGYCMDLLKELSKTINFTYSLAL
SPDGQFGN
YIIKNNSVGGKKEWTGLIGELVNERADMIVAPLTINPERAEFIEFSKPFKYQGITILEKKPSR
SSTLVSF
LQPFSNTLWILVMVSVHVVALVLYLLDRFSPFGRFKLANTDGTEEDALNLSSAVWFAWGV
LLNSGIGEGT
PRSFSARVLGMVWAGFAMIIVASYTANLAAFLVLERPKTKLTGINDARLRNTMENLTCATV
KGSAVDMYF
RRQVELSNMYRTMEANNYDTAEEAIRDIKIGKLMAFIWDSSRLEFEAAQDCELVTAGELF
GRSGYGIGLQ
KGSLWADAVTLAILDFHESGFMESLDNHWILRSNVQQCEQLEKAPNTLGLKNMAGVFIV
VGVGIIGGIGL
IIIEVAYKKHQIRKQKKMELARHAADKWRGAIEKRKTLRASIAAQRRIQSNGLNDPTTVSL
AVDTVARSN

VTPRSPGRAWPGDSDIRQRPIPRSDDIRLSPAAYTANVSHLIV

>BmorIR25a
MHFACAFFFVLADFLGAFAEIYLRFVFIVERHEPELFRMIGEAVKHAEDLKQLALDDSIVSL
DRENEDEA
RGKLCSELSKGVSALIDLSWSPWEEAEHLASEAGVPIIRTALGPQPLVAAIDRYLESRNATD
AAILLESE
IDVDKVLYELLGRSNTRIWVHAGLTRDSARALKTMRPDPSFYVLVGGSGFVMETYKRAV
KEKLVRRVYRW
NLVFTDYLDLSALDLSSVVQPTMILQAHPEDCCRIIASKDCTCSDSFEVMSGLFLRHRYVL
SSYKPDSGL
EAVAVWSANQEYRLLPGVTLEPLKHFFRVGTSPAVPWTLPKLDPETGDPLYNEDGQPIYEG
YCVDLIQKL
SEAMNFDYEIVSPRSGGFGRRLPNGSWDGVVGDLTTGETDIAVAALTMTAEREEVIDFVAP
YFEQTGILI
AIRKPIRKTSLFKFMTVLRTEVWLSIVAALVLTGFMIWLLEKYSPYSAKNNPGAYPYPCRD
FTLKESFWF
ALTSFTPQGGGEAPKALSGRTLVAAYWLFVVLMLATFTANLAAFLTVERMQTPVSSLEQL
ARQSRINYTV
VEGSSTHQYFINMKFAEDTLYRVWKEITLNATSDQAQYRVWDYPIREQYGHILLAINASGP
VADAETGFK
QVNDHTDADFAFIHDSAEIKYEVTRNCNLTEVGELFAEQPYAIAVQQGSRLQEDISRALLE
LQKERFLEQ
LTSKYWNETLRQSCSDADESEGITLESLGGVFIATLFGLGLAMITLAWEVFYYKRKEKNK
VQSTKENVER
PPIKSAKLGGKMAVGVARLRKRATKIGKKKNVTIGDSFKPSVSYISVYPKGDYRP
>BmorIR93a
MVSRVVQSLTSQIDEESARPVSVTVFKMKHEMNEYLRRKEMHRVLSKLPVKYIGENFIAI
VTSDVMTTMA
EIARELLMSHTMAQWLYVISDTNAHASNLSGFINTLNEGENVAFIYNITENGPDCKNGLM
CYSQEMMSAF
ISALDAAIQAEFDVAAQVSDEEWEAIRPSKVQRRDILLKHMQQYILAKSVCGNCTLWRAL
AADTWGVTYR
QNDVPEQINEHANGSTGVIEHLELMNVGIWRPIDAMTFADLLFPHVHHGFRGKELPIITYH
NPPWTFLQA
NESGAIVKYSGLMFDIVNQLAKNKNFTIKIMLPSHVKHGFSNETDMMHSQSARLTIAAIA
KGHAALAAAP
FTVLPGPNQGINYTIPVSTQPHTFIVARPRELSRALLFLLPFTTDTWLCLGFAVILMGPMLYI
VHRLSPY
YEAMEITREGGLATIHNCLWYIYGALLQQGGMYLPRADSGRLVIGTWWLVVLVIVTTYSG
NLVAFLTFPK
LEAPVTTISELLKNSDAYTWSVTKGSYLEMELKNSEEPKYKRLIKEAELLKETGGIEGTIH
AARGTLDRV
RGQRHLIFDWRLRLTYLMSADHIATETCDFALAVEDFMEEQVAMIVPAGSPYLPVINKEIN

RMHKAGLIS
KWLSAYLPKPNRCLKISTVTQEVSNHTVNLSDMQGSFFVLFLGFFSASTVLILEWFYNRRK
RKSEEIVIK
PYVE
>BmorIR40a
MKPFFLMLFLLLTDADCFFDIKEIISDKMTKLPKDFNVAIKDIAESLPSKEMTVVRGNSTNI
RSQDVFEL
LRLLCQHNIQVVNLDIAAMENKEMYYGYLKKALDVSDERTNLILCEPYECENLLLELREN
NLIHRTILYI
FFWPYGSVSDRFLNTMVEAMRVAVITNPRESVFRIYYNQATPNRLNHLSLVNWWAFRLYK
SPLLPSADKV
YKNFRGRVFDVPVLHAPPWHFVKYNNDSSINVTGGRDDKLLKLIANKLNFRYRYYDPPD
RSQGSGIIGNG
TFKGTLGLIWKRQADFFLGDVTMTWERLQAVEFSFLTLADSGAFLTHAPAKLSETLAIIRPF
RWEVWPLV
CATLFITGPALWIVIAAPSLWQRKKRDQMGLLNNCCWFTVTLFLRQSSTKEPSSTHKARLV
TVLISLGAT
YVIGDMYSANLTSLLARPAKEPPIGTLPALEEAMREHGYELVVESHSSSLSILENGTGVYG
RLAKLMKRQ
RVQRVHNVEAGVRLVLNRRRVAVLGGRETLYYDTERFGSHNFHLSEKLYTRYSAIAFQIGS
PYLETINNV
VMTLFEAGILGKMTTDEYKNLPEQSRRSEPVTESENLSTEKTGETAAVTQIQNETSKGLEP
VSLTMLRGA
FCLLGIGHLLAGVTLLIEIQLYRRARKRALPPQTRNPTNTFKAKAKKCILRGWRRIKAAAIL
AIDRALAP
DRGID

>CbowIR8a
MRNVKIILLENEGQDTILTWYRSIVQAFKSPIKFEEFLISVDGEEFDRERICQAFSNGAMMIL
DLTWTGN
DLARTVSMEMDVPYLRIDVSLSPFFDLLHEYLNFRNSTDVALIFDDPSRIDQAIYYWIDNV
QIAMSISES
LDAMAAKKLRDFRPTPNSFAIFAETKNMEKMFKIALEENLVTLPERWNLVFLDFHHKSFD
RGLLKKMPVN
LLTLDAGLCCQLLNLNSYCECPSRFNTSKMFLKIALNMLVTAIEELFKDDFKFHDNIDCDS
NFTKDNEES
VRKTFEEVLNKAVGNDNLIRLDNSSNLRLKTTGSIEIGTDVGTEVFAKYENEAITALRNKIV
KPIKAFYR
VGITHALPWSYQIKDPVTKKLVWTGYCVDFTAKLAEKMNFDYELVEPKKGTFGKKHNG
VWDGVVGDLASG
QTDLAITALIMTADKEEVIDFVAPYFEQTGITIVMRKPVRKTSLFKFMTVLKLEVWLSIVAA
LIVTGFMV
WFLDKYSPYSARNNKKAYPYPCRKFTLKESFWFALTSFTPQGGGEAPKALSGRTLVAAYW
LFVVLMLATF

TANLAAFLTVERMQAPVQSLEQLARQSRINYTVVSNSQTHKYFINMKFAEDTLYRMWKE
LTLNASTDDSR
YRVWDYPIREQYGHILLAINDSNPVANAEEGFKNVNEHLDADYAFIHDSSEIKYEISRNCN
LTEVGEVFA
EKPYAVAVQQGSHLQDGISKMILLLQKDRFFEGLQAKYWNNSVKGDCPNTDDNEGITLES
LGGVFIATLF
GLALAMITLAGEVLYYRRKRKTKELNIKQSKVFPEKPLDVFPPKPLLLGNNQITIGNTFKP
VNLKEKIRK
EREAMKISHISLYPRARKPINPFEIK
>CbowIR6
MNLRTKMGLYKNILLFQLLLGYCEGQTTQNINVLFVNEEGNEVAEKALDVALTYLKKNN
KLGISVDIRKV
VGNRTDSNAFLESLCSTYSSMLDAQAYPHLVLDTTMTGLGSETVKTFTQALALPTISASFG
QEGDLRQWR
NIDDNEKDFLIQISPPADIIPEIVRTLVLNQNITNAAILFDKSFVMDHKYKSLLQNVATRHIIT
AIKDGN
QVVDQLSQLRKLDLVNFFVLASLKNIKRVLDAADSVGFFNRKFAWHVITQDDGEIKCVCR
NATIMFVKPL
PNAAYQDRLGTMKRTYQLNVEPIISSAFYFDLTLHSFLAIKEMISDGVWKSVTNYITCDDY
NTENVPKRN
GLNLKKYFNKESTESPTYGPITVLSNGLSYMEFQMQLTSVGVRDGASDKSTILGTWSAGF
YNNLTIVEQQ
VMVNLTADVVYRVVTVEQKPFMFRDESSPRGYSGYCIDLIEKIADILKFDYEIATVDCFGT
MDENGKWNG
VVKELMEKRADIGLGSMSVMAERENVIDFTVPYYDLVGITVLMKLPETQSSLFKFLTVLE
NEVWLCILAA
YFFTSFLMWVFDRWSPYSYQNNREKYKDDEEKREFNLKECLWFCMTSLTPQGGGEAPKN
LSGRLVAATWW
LFGFIIIASYTANLAAFLTVSRLDTPIESLDDLSKQYKIQYAPLNGSSTQTYFERMANIEERF
YQIWKDM
SLNDSLSEVERAKLAVWDYPVSDKYTKMWQAMKEAGLPNTMDEAVAKVRASKSSTEGF
AFLGDATDIKYM
ELTNCDLTVVGEEFSRKPYAIAVQQGSPLKDQFNTAILQLLNRRELERLKEKWWNKNPEK
KDCEKADDQS
DGISIQNIGGVFIVIFVGIGLACVTLAIEYWWYKYRKGSKIIDVREVAHNPTKPPTFPKQKFS
EHNPDNT
KPKLPKRSKF
>CbowIR5
MGLIEFVVASLCLNATCEPEDAVVPGVSTHLLKLNELAEELKEETLTVTTFENGQLSGYISQ
NGSFLGTG
VAFDIFHILQEKFGFNYTIVLPDADIFMDGFNKKGAKSLLEAKQADIAVSFLPVIESFRNDV
VYSRVFDI
AEWNVLMNRPKESATGSGLLAPFTTAVWILIIFSVLVVGPIMYLMILIRAKMCKDDNNKIF
SLPSCMWFV

YGALLKQGSTLNPKSDSSRILFSTWWLFILILTAFYTANLTAFLTLSKFTLPITDPTDISRKNY
HWVTNK
ANGLRDYIEYEKHDRLSNGRTLVQDIGKDRYYADMKDLDILEEYVKKRNMMFIREKTLIK
NVMYRDYQEK
TKRGVDEEKRCTFVMADFPITMFSRGFAYTHDFKYAELFDRTFQYLIEAGIIQFKLRENLPD
AEICPLNL
GSIERKLRNTDLMLTYVIVASGLGIAASVFLLEILWRMSKAKYKRTRKRKATTWLEKNNN
LMKAKCLHLH
TNSSPPPPYQALFRPPFYYSDRDGGQKKTINGRDYWVIDKSDGLREIIPIRTPSALLFQISN
>CbowIR75q
MLNSLTLFMILFLNFSSAMKNYTEIINIVDELLTKQNIPSEVTAYLCWSKVLKANLFKRLSA
SNILTKII
ATDDIVDLFPSEYQIYLVDLDCEGSNEILKKAQRKKLFSRPFRWVFCGNIEQPLFNDLYFGV
DSRIFFID
NAGSEYHIKMPYKREKNSKKFTVNDLAEWNSLQGFTRFDEFAAARNRTDLFGMNINISCV
YTDSDTLNHL
EDYRNIHIDPLTKLSWILVHHLMSILNASATVIFRNTWGYRDSNTSLFSGMIGDLQTGEAEL
GGTASFFT
IDRIDVVEFYASSAPTYMKFIFRAPPLSYVTNVFTLPFHTYVWYCSFLLLVLIFFAIYVIVKW
EWKDVVF
REKLERMHDGSISPLRPTFFSVLLMEIGAITQQGTDSEPKSNAGRIATIFTFIALMFMYTSYS
ANIVALL
QSTTESIRTLEDLLNYRISLGVQDIVYAHHYFEVRIQLSIDSYPNQTPGLKWLNTQDLGKL
WR
>CbowIR41a
LFLPGDNLLENLSHIYSMQELKYIADIVIVNREYKDEDSQGLYMSDNVFSLWTHSYRGMD
ENAKRMFLDL
WFSKNQSFMLDENLYPDKLVNQMGRKLEMATFQYEPYSIIGSSETESKGSEMVTCLTFAR
HYNMTPVLVV
NDEGYWGDIFDNWTGYGLLGNLVEDKADIGFSALYTWESDYYFLDLSKPLVRTGITCLVP
APSLAAGWTT
PLYSFSTTMWAAVGSMFFVCIFVQFFMHYFHAKIYDDTNQSLKLLDRSILCVLKLFVQQV
VTTRETPPGR
SGKYFMGLLFTFSLFLSSSYSSGLSSIMTIPRYGRPINTVEEFAESKISWGATQDAWTMSLK
GVEDPT
>CbowIR21a
MDYIIRANYTSKSKRTVLHVSSQCFALFKVAVIFPKKSIYGETLSQGVKLMIQAGLLSKIRS
DVEWDMIR
SPNGKLLAANSRTTGLKIISYEDRALTLDDTQGMFLLLGAGFLIGAAALTSEWFGGCLKLF
KRIRPPSSD
SSIASNPRVHTGRMPRKK
>CbowIR68a
LYTYGMLLLVSIPKLPTGWSLRMLTGWHWLYCLLVVTSYRASMTAILAKPAPKVKIDTLQ
ELVSSQLTCG

GWGEMNSEFFKSSDDPLVTTISQNF
>CbowIR64a
MDPPRTIFTLKDLAESQLRIGIEDILIDRNYFVQTTDPDAITLYEKKIKGQSNSSGFYSPSEGI
ALVRNG
GFAFHVETSTAYPIIEEIFTNQEICELDEIQMYRTQPMHTNLQKNSPFREMMNFCMLKLVEN
GNMDRLRK
HWDARRPNCIESAKKQEIHVSLSEFCCSPIALTLGVCFSLIFLLVECSINYKERLKKVWTFK
NHSKSQYP
FME
>CbowIR2
MVTEFSCDKLGSFEFLKKLITQGIPTRILLIDKDELSYLFPPNCQIFIVNLQCENSTNILKKA
NSLKLFS
FPFRWIIYHHEPINETIFEESFLSLDILVDSDVTLLEENKNKSVSATKIYKRHRNHPLVIEKM
GYWTKTA
GLRDDREEKIMVRRRKNLQQIPLNTCIVITHNDSLKHLTDKRDKHIDSIAKVNYVLVEHLS
DIVNVTLNY
SIQNTWGYKNNKSEWSGMIGELTKNEADIGGTPLFFIIDRVDIIDYIAMTTPTRSKFVFREP
KLSYVTNV
FTLPFDDYVWASTIALVCIISMVLFIILKWEWKKKDLPSEKDSSNVPELKDSLTDVILFSFG
AFCQQGAP
SIPFSVPGRITTIILFVSLMFLYTSYSANIVALLQSSSTSIQTLEDLLKSRLQVGVDDTVFNRF
YFPNAS
EAVRRAIYLQKVAPPGKKENFMSIEEGVKRMRQGLFAFHMETGPGYKLVGEMFHESEKC
GLKEIQYLQVI
DPWLAIQKNSSYKELLKIGLRQIQESGLQTREVSLIYTKKPICTSRGSSFISVGLVDCYPAAV
VSAGGAI
LALIVWILELGLYYRPYMWISVKKVFAKSHEKIPGSIEQWPEWPYLK

>DmelIR25a
MGSRLDWGVADVALWAIADQIDYHQVFINEVDNEPAAKAVEVVLTYLKKNIRYGLSVQL
DSIEANKSDAK
VLLEAICNKYATSIEKKQTPHLILDTTKSGIASETVKSFTQALGLPTISASYGQQGDLRQWR
DLDEAKQK
YLLQVMPPADIIPEAIRSIVIHMNITNAAILYDDSFVMDHKYKSLLQNIQTRHVITAIAKDG
KREREEQI
EKLRNLDINNFFILGTLQSIRMVLESVKPAYFERNFAWHAITQNEGEISSQRDNATIMFMKP
MAYTQYRD
RLGLLRTTYNLNEEPQLSSAFYFDLALRSFLTIKEMLQSGAWPKDMEYLNCDDFQGGNTP
QRNLDLRDYF
TKITEPTSYGTFDLVTQSTQPFNGHSFMKFEMDINVLQIRGGSSVNSKSIGKWISGLNSELI
VKDEEQMK
NLTADTVYRIFTVVQAPFIMRDETAPKGYKGYCIDLINEIAAIVHFDYTIQEVEDGKFGNM
DENGQWNGI
VKKLMDKQADIGLGSMSVMAEREIVIDFTVPYYDLVGITIMMQRPSSPSSLFKFLTVLETN

VWLCILAAY
FFTSFLMWIFDRWSPYSYQNNREKYKDDEEKREFNLKECLWFCMTSLTPQGGGEAPKNL
SGRLVAATWWL
FGFIIIASYTANLAAFLTVSRLDTPVESLDDLAKQYKILYAPLNGSSAMTYFERMSNIEQMF
YEIWKDLS
LNDSLTAVERSKLAVWDYPVSDKYTKMWQAMQEAKLPATLDEAVARVRNSTAATGFAFL
GDATDIRYLQL
TNCDLQVVGEEFSRKPYAIAVQQGSHLKDQFNNAILTLLNKRQLEKLKEKWWKNDEALA
KCDKPEDQSDG
ISIQNIGGVFIVIFVGIGMACITLVFEYWWYRYRKNPRIIDVAEANAERSNAADHPGKLVDG
VILGHSGE
KFEKSKAALRPRFNQYPATFKPRF

>DmelIR40a
MACNELHNGYRAKFLTIVYWIAATYVLADVYSAQLTSQFARPAREPPINTLQRLQAAMIH
DGYRLYVEKE
SSSLEMLENGTELFRQLYALMRQQVINDPQGFFIDSVEAGIKLIAEGGEDKAVLGGRETLFF
NVQQYGSN
NFQLSQKLYTRYSAVAVQIGCPFLGSLNNVLMQLFESGILDKMTAAEYAKQYQEVEATRIY
KGSVQAKNS
EAYSRTESYDSTVISPLNLRMLQGAFIALGVGSLAAGVILLLEIVFIKLDQARLWMLCSRLQ
WIRYDRKV

>DmelIR31a
MNLLISMFILILAAGEGEIIPSMEESVVTNFVKSLVKTKQAIVFSCLFKDFKEISLALMRINQ
FVSVVNL
NQSYSLTSILTRENYARTSVMVNARCSGSSELLFEASENRYFNKTYQWFLWGVDLEVQSL
FPLNLNYVGP
NAQITYVNETADGYAYWDIHSKGRHLKSNLEINLIATLINDTLNIARDIFHLQSIDFRGQFN
GLTLRGAS
VIDKEDIISNEQIESILSRPTKDAGVAAFIKYHYELLGLLRERFNFTVNFRNSRGWAGRLGN
TTFRLGLL
GIVMRNEADIAASGAFNRINRFAEFDTIHQSWKFETAFLYRYTSDLDTHGKSGNFLSPFSDR
VWLFCLLT
LGAFSIIWVLFEIIDYKILRIRVNSQKLEHLNQKSSVICIKTTCIERILQTFGACCQQGLDPNP
VDRSVR
FLVMTLFLFSLVMYNYYTSSVVGGLLSSSDQGPSTVDEITASPLKISFEDIGYYKVLFRESQ
NRSITRLI
EKKLSSSRSLNELPIFSHIEDAVPYLKAGGFAFHCEVVDAYPVISEYFDANEICDLREVSGL
MEVEILNW
ILHKNSQYTEIFKTAMCNAQEKGFVERILRRRQIKKPACQSLYTVYPVSLSGVLPGFVILIC
GFGASLLL
LCLEKVYAHFGPRKFCGF

>DmelIR7c
MLHSAVHNVSLVYALVWAIDNYYGMATSTPLAVVQFPTSRESRRLHNDLIDAALGRSSGT
GRIQFLLEDD
RVEMTETDTDPPPPSGLTGRPIAIWFLDSLRSYFRLEMYLNQLGSPYKRNGFFLVIYTGLED
QPMESLKI
MFRRLLNMYVLNVNVFLQRDGTVHLYTYYPYGPHHCQSSLPVYYTAFQDLAAPANGFGL
TKPLFPRKLTN
MHGCEMVVATFEHRPYVIIEDDPKTPGGRSIHGIEGLIFRSLAERMNFTIKLVEQKDKNRGE
ILPDGNFT
GILKMMVDGEVNLTFVCFMYSKARSDLMLPSTSYTSFPIVLVVPSGGSISPMGRLTRPFRYI
IWSCILVS
LIFGFVLICLLKITALPGLRNLVLGRRNRLPFMGMWASLLGGLALYNPQRNFARYILVMWL
LQTLILRAA
YTGQLYLLLQDVEMRSPIKSLSEVLAKDYEFRILPALRTIFKDSMPTTNFHAVLSLEESLYR
LRDEDDPG
ITVALLQPTVNQFDFRSGPNKRHLTVLPDPLMTAPLTFYMRPHSYFKRRIDRLIMAMMSSG
IVARYRKMY
MDRIKRVSKRRNLEPKPLSIWRLSGIFVCCAGLYLVALIVFILEILTTNHRRLRRAFNVINRY
AA

>DmelIR68a
MRCLWILIVAFISLAMATSIPIPIANPAPLSGYEMQLKILLQKILWVANVKRCFAVITDDLHY
PIYDRIF
FESVGRRVIPFFVMRTNESDDLQRPSRQVELFVKAIKSSDCELNVITILNGWQVQRFLGYIY
DNRSLNMQ
KKFVLLHDLRLFESDMIHLWSVFIDAIFLKRQLDNKYTISTIAFPGILSGVLVMKNIANWEL
GKGLNGRI
LFADKTSNLFGTSLPVAISEHVPMVLWANATKSFQGVEVEIMNALGKALNFKPVYYKPNQ
TENMDWTELD
GGASVAYGSGNPDGYAQNGTHIDSMLVDEVAAHSARFAIGDLHLFQVYLKLVELSAPHNF
ECLTFLTPES
STDNSWQTFILPFSAGMWVGVLLSLFVVGTVFYAISFLNAIINGNVSSEFFRCLRPNRNVP
MDPKIYRRI
SFRIAISRYRSSKGDRMPRDLFDGYTNCILLTYSMLLYVALPRMPRNWPLRVLTGWYWIYC
ILLVATYRA
SFTAILANPAARVTIDTLEDLLRSHIPPSTGATENRQFFLEANDEVARKVGEKMEVFGYSDD
LTSRIAKG
QCAYYDNEFYLRYLRVADESGSALHIMKECVLYMPVVLAMEKNSALKPRVDASIQHLAE
GGLIAKWLKDA
IEHLPAEALAQQEALMNIQKFWSSFVALLIGYVISMLTLLAERWHFKHIVMKHPMYDVYN
PSLYYNFKRI
YPQH

>DmelIR76a

MFVYTKEFEDKKDSYLSGYIFQDQPNILVITSQYLNSSTFEIKTNRFVGPRNFNKNPEPVEF
YILQRFDA
KGTKATWETQSAMSSKMRNLKGREVVIGIFDYKPFMLLDYEKPPLYYDRFMNTTDVTID
GTDIQLMLIFC
ELYNCTIQVDTSEPYDWGDIYLNASGYGLVGMILDRRNDYGVGGMYLWYEAYEYMDMT
HFLGRSGVTCLV
PAPNRLISWTLLLRPFQFVLWMCVMLCLLLESLALGITRRWEHSSVAAGNSWISSLRFGCIS
TLKLFVNQ
STNYVTSSYALRTVLVASYMIDIILTTVYSGGLAAILTLPTLEEAADSRQRLFDHKLIWTGTS
QAWITTI
DERSADPVLLGLMEHYRVYDANLISAFSHTEQMGFVVERLQFGHLGNTELIENDALKRLK
LMVDDIYFAF
TVAFVPRLWPHLNAYNDFILAWHSSGFDKFWEWKIAAEYMNAHRQNRIVASEKTNLDIGP
VKLGIDNFIG
LILLWCFGMICSLLTFLGELWRGQG

>DmelIR8a
MELPLLVLLLALRFAGSEVLKITFWIEPVQRAEFDTDIAMVLKELDALRLDVKVDDTTLTL
TRSEDGLDM
QRFCEILSTVGASAVIDLTYSHWEEGYNLVRSLGIGYVRLERIMRPFLDMFGDFMRQKRA
NNVAMVFMNA
RDAVEAMQQMLVGYPFRTLIMDASQTDPGQHFLERIRSLRPAPTYIALFARAAAMNGIFEK
VQKADLFQR
PLEWHFVFLDTRDRVFKYRRQAELCTRFTLNPRAICRSMPMPDLYCGSGFTMQRAMLLN
VLRSLINAAQV
SPGYPLAIYQDCNATASSSEVSDPLEKDDYNWLDMVHWSNFLAYAPPLPHIQDQFQSPVP
GLTFAVNISA
GYYSSEHEAKTDLAAWSSVGEMRLLNETISPARRFFRIGTAESIPWSYLRREEGTGELIRDR
SGLPIWEG
YCIDFIIRLSQKLNFEFEIVAPEVGHMGELNELGEWDGVVGDLVRGETDFAIAALKMYSER
EEVIDFLPP
YYEQTGISIAIRKPVRRTSLFKFMTVLRLEVWLSIVAALVGTAIMIWFMDKYSPYSSRNNR
QAYPYACRE
FTLRESFWFALTSFTPQGGGEAPKAISGRMLVAAYWLFVVLMLATFTANLAAFLTVERMQ
TPVQSLEQLA
RQSRINYTVVKDSDTHQYFVNMKFAEDTLYRMWKELALNASKDFKKFRIWDYPIKEQYG
HILLAINSSQP
VADAKEGFANVDAHENADYAFIHDSAEIKYEITRNCNLTEVGEVFAEQPYAVAVQQGSHL
GDELSYAILE
LQKDRFFEELKAKYWNQSNLPNCPLSEDQEGITLESLGGVFIATLFGLVLAMMTLGMEVL
YYKKKQNALE
ITQVRPVNDSSGSGGNSSTAPPTATSTTKQAWHIPVLEAEEKPAKVSPPPSFETATFRGKKLP
ARITLGD
GKFKPRHGLYARRNLGASDSHSGYME

>DmelIR7g
MNVTSLLNFESMKYIGAQTQAASINHHVAQALRVFIEDFYQRIAPAFIVVLSCRRPSPMNF
YRNIMQLLY
ESVDTMIVQLVLVELGRPRRIAGPRTHNLLLVDSLDALLDIEIHTYTAQSDTSEYYFIFLQQ
RDALIPHD
MQGVFAYCWRHQLINCNVMTQSSGGQVLLHTYFPYAPGQCNDSQPTRINMFLGESWKH
RDYFPSKLHNLN
GCPLIVLARKVSPFLDLDEGQRELRGLEGRLLQELSRRMNFSIQFSGLQDQLKNRTTWTEK
QLLQKLVQE
RIAHLAIGYVRKRIQYATNLTPVFPHYSNRVVGCLLLNAHNLTSLEIWSFPFQALTWICLVF
SFLSISCL
ALLHXRGAGDRLALVLAVYAASLGLPIDPPERPSLQLLFASWLIFGLIVRSMYSALLFFILR
YHLHQRLP
GNLQDLTHGDYAAVMGRTTLQDLREVPSLQDLLGLKSVIVTSEREEEVLRTLDRCTLREG
AGSHPLFFGL
ISQDALLHLTQRGHRAGAYHIIPQDVLEQQLAIYLQKHSHLASHLDHLVMSIRSVGLVHHW
AGQMASERY
FRSRFLYREKRIRQPDLWAVYILTAGLYLLSLVVFICELLASRRAGL

>DmelIR67a
MLPILVPVLLLFNETSWINPILTSIYKDRHHETVLLLQHSQHGNASGLERFPWPVFSFNEQM
DFYVRGKY
NSEMLVLIWQTGNSDWDLDLWQALDRSLLNMRKVRVLLLRKWEKIPTADVAATAEHLLF
LHVAVIGQGNR
IYRLQPYAPQSWLQVDPIESPIFIKIRNYFGRYIVTLPDQFPPRSIVYRNPKTDEIQMTGYVY
KFLLEFI
RIYNFTFRWQRPIVQGERMNLILLRNMTLNGTINLAISLCGFETPSELGVFSDVYDMEEWY
IMVPRAQEI
SIADVYVVMVSGNFLIVLIIFYFIFTILDTCFGPLLLKERVDWSNLMLNERMISGIMGQSFN
MSARNTIS
SKVTNATLFLLGLVLSTLYAAHLKTLLTKRPTSQQISNFKQLRDSPVTVFFEEAERFYLKHA
WDRPIRYI
KDQLNFRETIEYNALRMGLNRSNAFSALTSEWMIVAKRQELFKQPIFTVQPELRVIQTSVL
LSLVMQSNS
IYEDHINDLIHRVQSAGIVEYWKHQTLREMITMGMISQKDPFPYVAFREFKVGDLFWIWLL
WVSFLFMSF
VIFLCELLVDCFISKTLIRNKRPH

>DmelIR60e
MVIKMISFLLVSVLLCLVGASDSESMQVQVLQDLNLALQTELNVFIDFECCATSEILHKLD
SPRILLSSN
SREARDLRIRGNFTESTLIIVSVMDSDLNPLVASLLPRLLDELHELHIVFLSNEEPGFPKQDL
YTYCFKE

GFVNVILMSGKGLYSYLPYPSIQPISLSNVSEYFDRARIIRNFQGFPVRILRSTLAPRDFEYS
NEQGGLV
RAGYLFTAVKELTYRYNATIESVPIPDLPEYDVYLAVAEMLHTKKIDIVCYFKDFSLEVAYT
APLSIIRE
YFMAPHARPISSYLYYSKPFGWTLWAVVISTVLYGTVMLHLAARGARVEIGKCLLYSLSHI
LYNCHQKIR
VAGWRDVAIHGILTIGGFILTNVYLATLSSILTSGLYDEEYNTLEDLARAPYPSLHDEYYRSQ
MKAKTFL
PERLRRNSLSLNATLLKAYRDGLNQSYIYILYEDRLELILMQQYLLKTPRFNMIRQAVGFTL
ESYCVSNS
LPYLAMTSEFMRRLQEHGISIKMKADTFRELIHQGIYTLMRDDEPPAKAFDLDYYFFAFVL
WTVGLISSL
LVFFAELVSGHL

>DmelIR41a
MFIDLSWSLVLSAIVGKYLNESTICIFWNDKFEFQLLHKSDYISFVGINIKSFDDNGGHYIID
TGLKKKE
LQNKHLFLDELVIKIIISIEVTHCETFVVFDKDIDRFVNAFNKASVYSIWRSLHNKFVFAHIA
NESPESR
NHFFEDQPNILFVVRDHSSASSFDIKTNKFVGRKAENPSQMILVDRYLASEQRFQFGKSLF
ADKLNNLQG
REVIIAGFDYPPYTVIKHNMSTNAQDMGVSGESDFKNVYIDGTETRIVLNFCEQFNCTIQI
DSSAANDWG
KVYPNMSGDGALGMLINRKADICIGAMYSWYEDYTYLDLSMYLVRSGITCLVPAPLRLTS
WYLPLEPFKE
TLWAAILLCLCAEATGLVLAYKSEQALYVLPGYREGWWTCTSFGVCTTFKLFISQSGNSKA
YSLTVRVLL
FACFLNDLIITSIYGGGLASILTIPSMDEAADTVTRLRFHRLQWAANSEAWVSAIRASDEAL
VKDILYNF
HIYSDDELLRLAQDQHMRIGFTVERLPFGHFAIGNYLGPQAIDQLVIMKDDIYFQYTVAFV
PRLWPLLDK
LNTLIYSWHSSGFDKYWEYRVVADNLNLKIQQQVQETMTGTKDIGPVPLGMSNFAGFIIV
WILGSAIATL
TFLLELSLTYILKQSNLK

>DmelIR94c
MSKVFKLLVLPLIYLSLTKGSKNPQLKFLRELINVIEEGREIRTIMVIKHSRDEYCHLDQWN
PRGSPILR
TNEMGSIRISGYFNDQAVILACMGENSDYGLLKSLANAMDNMRQERIILWSEREPTKMLM
DYISQQADRY
NFAQIIIVTMNEDVDAVPSLHQLNPYPTPRFRQITNISNIRRTSFFGCGLSFQGKTAILKESVV
SNIRFK
VWSPSGPIPLSELKDYEIVQFAVKYNLSLKLYDQNESKSDHFDIQLGPLFITKDFPTQMAFV
SPNTACSL

IVIVPCSPKWRFMDVLHKLGVLKLIGCLLIAYAVFVLIETLILWLTHRISGREVRLTSLNQLL
NPRAFRG
ILGLPFPEFRRSSISLRQLFLVISVFGLVYSNFVSCTLSALLTKPAQNPQVRNFKELRDSGLITI
MDKYT
HSFIEKHIDPEFFDHVLPHYLILQKKEALRMIWNFNDSYSYVMYTTTWKSLNTVQKSFDE
RVFCESESLT
IAWNLPRMYVLGNNSVLKWMLSRYITYMPQTGIPDSWTEQLPKVLKLLYNVTSPRRIKEG
AVPLSIQHLS
WIWHLLFIGESIATLVFIVEILLQKSNQHTSNMRERSSEDDDFV

>DmelIR94b
MSLIFNLLFILILSQAVSQETEFLQLKYLNNIVRSMIKLHKMETLVIVKHHLDNNCSLQNW
NAHGMGIIR
TNDQGKLIMKDTFNSRTLAIICIGQNSHITLLRNVFETFGKVQQKKIILWTQMELKEKFFQE
ISKKSRDL
KLLNLLVLKAVTKDKLLIYRLNPFPSPHFKRIENIWTPNDTLFMDTKFNFHGMTAVVKHD
YNWTIQMGNI
RKFPISRIEDKEVIEFALKYNLTLQFFNDVERFDIELRKRIILKSNSTQPIDSGIPMVFSSLLIV
VPCGN
YLSIQDVIKVSGIEKWIFYIILVYVIFVLIEITFLGVTILISRQSRHQMIPNTLVNLCAFRAILGL
PFPE
TRRTSLSLRQLFLAIALFGMIFSIFINCKLSSMLTNPCPRPQVNNFEELKTSGLTVVMDHDA
ENFIEKEI
GVDFFNQYMPRKVTLTFTERAKLLFSLKGNHAFTLFSESFAIIESYQRSKGLRAHCTSEDLI
VAERVPRI
YILENNSILDRPLRRFIRQMQESGITNHWLKNIPSSLEKNLMQITIPYDRERVHPLSIEHLTW
LWCILIL
GYSISMIVFFVEMSLKRRKKNLENRAPNICIC

>DmelIR93a
MNPGEMRPSACLLLLAGLQLSILVPTEANDFSSFLSANASLAVVVDHEYMTVHGENILAH
FEKILSDVIR
ENLRNGGINVKYFSWNAVRLKKDFLAAITVTDCENTWNFYKNTQETSILLIAITDSDCPRL
PLNRALMVP
IVENGDEFPQLILDAKVQQILNWKTAVVFVDQTILEENALLVKSIVHESITNHITPISLILYEI
NDSLRG
QQKRVALRQALSQFAPKKHEEMRQQFLVISAFHEDIIEIAETLNMFHVGNQWMIFVLDMV
ARDFDAGTVT
INLDEGANIAFALNETDPNCQDSLNCTISEISLALVNAISKITVEEESIYGEISDEEWEAIRFT
KQEKQA
EILEYMKEFLKTNAKCSSCARWRVETAITWGKSQENRKFRSTPQRDAKNRNFEFINIGYW
TPVLGFVCQE
LAFPHIEHHFRNITMDILTVHNPPWQILTKNSNGVIVEHKGIVMEIVKELSRALNFSYYLHE
ASAWKEED

SLSTSAGGNESDELVGSMTFRIPYRVVEMVQGNQFFIAAVAATVEDPDQKPFNYTQPISVQ
KYSFITRKP
DEVSRIYLFTAPFTVETWFCLMGIILLTAPTLYAINRLAPLKEMRIVGLSTVKSCFWYIFGAL
LQQGGMY
LPTADSGRLVVGFWWIVVIVLVTTYCGNLVAFLTFPKFQPGVDYLNQLEDHKDIVQYGLR
NGTFFERYVQ
STTREDFKHYLERAKIYGSAQEEDIEAVKRGERINIDWRINLQLIVQRHFEREKECHFALGR
ESFVDEQI
AMIVPAQSAYLHLVNRHIKSMFRMGFIERWHQMNLPSAGKCNGKSAQRQVTNHKVNMD
DMQGCFLVLLLG
FTLALLIVCGEFWYRRFRASRKRRQFTN

>DmelIR92a
MLLQPLVMHLSQLLRIIVGQYFAEFPSILIVYNNSASTTPLQLEYLSALELVLRELSKPIRLQ
WINVAFL
KDLNDLEDQVMGALNSSVTEGFITILSQTHHFIHARYYATRNANVRLKDKRYLFLCEDESP
AELLCMDIL
QFYPHHLMVRPGTETAPTGPTGPHPDPRRGGGASVSTKNKDDGEGGAGNKTTSPYRDINF
ELWTQKFVGA
VGNLDALLLDAFLPNETFANRVELYPNKLLNLQRRSLLVGSITYVPYTITNYVPAGQGDVD
PIHPQWPNR
SLTFDGAEANVMKTFCQVHNCHLRVEAYGADNWGGIYDNESSDGMLGDIYEQRVEMAI
GCIYNWYDGITE
TSHTIARSSVTILGPAPAPLPSWRTNIMPFNNRAWLVLISTLVICGTFLYFMKYVSYRLRYSG
TQVKFHH
SRKLEKSMLDIFALFIQQPSAPLSFDRFAPRFFLATILCATITLENIYSGQLKSMLTFPFYSAP
VDTIEK
WAQSGWKWSAPSIIWVHTVQSSDLETEQILARNFEVHDYSYLSNVSFMPNYGFGIERLSS
GSLSVGDYVS
TEALENRIVLHDDLYFDYTRAVSIRGWILMPELNKHIRTCQETGLYFHWELEFIDKYMDKK
KQEVLMDLA
NGHKVKGAPQALDVRNIAGALFVLAFGVAFAGCALVAELLIHRMDLSK

>DmelIR7b
MKYWLYILSCCSLVASTMESSSDWDLAEALAQVVANSEMGRFKTLYIYTHTNSQSTGGHL
EELLDQVLMI
VPNNLQARRLLLQQSMEYKPYVHAVLALVDGLPSLSAIYARIRATQDLSHTLIYMSMPTD
AYGEEMQATL
RFLWRLSVLNVGVVLRPPGDHILMVSYFPFSALHGCQVISANVVNRYQVGTKRWASQDY
FPSKLGNFYGC
LLTCATWEDMPYLVWRPDGSGSFVGIEGALLQFMAENLNFTVGLYWMNKEEVLATFDES
GRIFDEIFGHH
ADFSLGGFHFKPSAGSEIPYSQSTYYFMSHIMLVTNLQSAYSAYEKLSFPFTPLLWRAIGLV
LILACLLL

MLLVRWRHHHELPRNPYYELLVLTMGGNLEDRWVPQRFPSRLVLLTWLFATLVLRSGYQS
GMYQLLRQDT
QRNPPQTISEVLAQHFTIQLAEVNEARILASLPELRPEQLVYLEGSELQSFPALAQQSGSSAR
VAILTPY
EYFGYFRKVHPMSRRLHLVRERIYTQQLAFYVRRHSHLVGVLNKQIQHAHTHGFLEHWT
RQYVSAVDEKD
ESVARIASTSYSTLDGIDGDPSLSESEEDQQVAPVRQNVLSMRELAALFWLILWANLGAVV
VFVLELLLP
RIKLRKILRKMKSDIKKQISKLVRK

>DmelIR87a
MSTPEQRFWLAALLFLLSQHSEVRGFGINLMKVQTEDKGQEACILALLRKYFDSGDGLSG
SVLCINRNYQ
LPNIEEQLLRGVNNYENYPWSLLITNSREGPSPAKFLMNEKPQCYFLIVDNLEDEDLDEVF
EHWKGMVNW
NPLAQFVVYLASLEETDEEMNDLMVELLLTFINKKIFNVNVIGQSEENQFYYGKTVFPYH
PDNNCGNRVI
SVELLDACDYPSEETDSEDENDEDEGDGAQEEDDGPQEEGDGEQEEEDGPQEQEDGDQA
KGDEGQENDDG
GLENKVENEFRIGASDDDELENDLSSNSSEPEAIIEEFFRAKFEDKFPRDLSGCPLTASFRPW
EPYIFRN
SEEQPVDDYYYGLQGDEDDYNDTSPNYGESDDESYADPGEDGDGAIPDTETQSGGKLKL
SGIEYEMVQTI
AERLHVSIEMQGENSNLYHLFQQLIDGEIEMIVGGIDEDPSISQFVSSSIPYHQDELTWCVA
RAKRRHGF
FNFVATFNADAGFLIGIFVVTCSLVVWLAQRVSGFQLRNLNGYFPTCLRVLGILLNQAIPAQ
DFPITLRQ
LFALSFLMGFFFSNTYQSFLISTLTTPRSSYQIHTLQEIYSNKMTVMGTSEHVRHLNKDGEI
FKYIREKF
QMCYNLVDCLNDAAQNEHIAVAVSRQHSFYNPRIQRDRLYCFDRRESLYVYLVTMLLPKK
YHLLHQINPV
IQHIIESGHMQKWARDLDMRRMIHEEITRVREDPFKALTFDQFRGAIAFSGGLLLVASCVFA
FELCYVKY
VYRTEKRERKTKKITKKVHNIKIQHD

>DmelIR84a
MIKLQVKVISWPLIILTAFLRVLQIESINTNFLELAAFEDFLRSEHLSHVLVVRGDDADGDW
KIECHQKL
LANYRVQFYRPEMSANFEDLMFYGSPRTAVLVLNSEHVLVRRQVFGVASEAGYFNNSLA
WFILGSGRESL
PVEQLIDQLLSGYRMGIDADITVALRGPDNASMLFYDVYRISRQANTPLIIEKKGLWTHSG
GYQKFGNFK
NTWVIRRRNFLNVTLIGSTVLTEKPPGFGDMEYLADDKQLQQLDPMQRKTYQLFQLVER
MFNLSLAISLT

DKWGELLDNGSWSGVMGQVTSREADFAVCPIRFVLDRQPYVQYSAVLHTQNIHFLFRHP
RRSHIKNIFFE
PLSNQVWWCVLALVTGSTILLLFHVRLERMLSNMENRFSFVWFTMLETYLQQGPANEIFR
LFSTRLLISL
SCIFSFMLMQFYGAFIVGSLLSESARSIVNLQALYDSNLAIGMENISYNFPIFTNTSNQLVRD
VYVKKIC
KSGEHNIMSLQQGAERIIQGRFAFHTAIDRMYRLLLELQMDEAEFCDLQEVMFNLPYDSG
SVMPKGSPWR
EHLAHALLHFRATGLLQYNDKKWMVRRPDCSLFKTSQAEVDLEHFAPALFALALAMVAS
ALVFLLELFLH
WLPDFRRRLGTMST

>DmelIR94h
MLSNISFSSAPELVDLYGLVLKFLVSSETTLFYFNPTGQKCSWETLPRTILSNHPQIIWFREE
TYPGLYK
RHSSNLFVMACLSSTSYDGQLQLLAESLTRYRSVRVLIEVQDKEGSFLASQILLLCQQHSM
LNVVLYFSR
WTRTLNVFSYLAFPYFKLLKQRLSGSLRPKIFINQLKDLQGYKIRVQPDLSPPNSFSYRDRH
GECQVGGF
LWRIVENFSKSLKGDTQVLYPTWAKAKVSAAEYMIQFTRNGSSDIGVTTTMITFKHEERY
RDYSYPMYDI
SWCTMLPVEKPLSVEILFSHVLSPGSALLLILAFILFFLIVPQLIKCLGITFRGRLIGMASRIFA
LVMLC
SSSAQLLSLLMSPPLHTRIKSFDDLLTSGLKIFGIRSELYFLDGGFRAKYASAFHLTENPNEL
YDNRNYF
NTSWAYTITSVKWNVIEAQQRHFAHPVFRYSTDLCFSSETPWGLLIAPESFYREPLQHFTL
KINQAGLIT
QWMTQSFHEMVRAGRMTIKDYSRTNLMKPLRIQDLRKCWVIFAVGLGTSTVVFTIELLLI
YTNVFLNSL

>DmelIR94g
MSTAVNSVHSKLVSLISRGQELTSIFFYAPAKEKCHLEDTISSATWGLPLVIWRTDRTVILNG
FIGEGLL
VLACLPGFHWRALLGSLARSLKYLRQARILIELMQDRDEFLVSEVLQFCLSQDMINVNAIF
DDFPETENL
SSFEAYPSFEVVNQTFTPDTQVSDLYPNKMLNLRGGVIRTMPDYSEPNTILYQDKEGNKEI
LGYLWDLLE
AYAHKHNAQLQVVNKYADDRPLNFIELLDAAQSGIIDVGASIQPMSMGSLSRMHEMSYP
VNQASWCTMLP
VERQLHVSELLTRVIPYPTLALLLLLWIFYEVLRGRWRRHSRLQSIGWLVLATLVSSNYVG
KLLNLFTDP
PSLPPVNSLAALMESPVRIISIRSEYSAIEFTQRTKYSAAFHLALHASILIGLRNAFNTSYGY
TITSEKW
KIYEEQQKRSSKPVFRYSKDLCFYEMIPFGLVIPENSPHRAPLHSYTLLLRQAGLHDFWVN

RGFSYMVKA
GKINFTAVGERYEAKTLTITDLRNVFIIYVSVLLISLILFTCELFVSWVNYWLGF

>DmelIR94f
MWQQVLLAETSNWFRSDVLQRFWTHLRVEIRFRTMLNYRLESCDCWFDNVLGSDNSTA
LLWNDQTYPHYL
RRRQDTDILVVSCLRFHQYQEVLLALSLMLDQMRSMPVVLQLCGDEDSMQELNSARLLL
KHSQDLKMPNV
VLLSSTFFTSATLYSYEMFPEFNVQKLVYQAYLTLFPYKLGNLKGHPIRTVPDNSEPLTIVR
KTLNGSIA
IDGLVWQFMIEFAKHINATLQLPIEPHPEKSIKLVQILDLVRNQTVDIAASLRPYSLNVQRSS
THIYGSP
MMVGNWCMMLPTERVIGSHEALTRLMKSPWTWLILLLFYSVHRFLAQKTRLRSSLIHLIK
LLINLSLICF
LQAQLSAYFIGPQKVNHISNMQQVEESGLKIRGMRGEFMEYPIDMRSRYASSFLLHDLFFD
LAQYRNSLN
TSYGYTVTSVKWELYKEAQRHFRRPLFRYSEEICVQKLSLFSLIQQSNCIYCYRSRIFILRM
HEAGLIRL
WYRRSYYVMVTAGRFPIGDLSTVHRAQPIRWTEWQNVVLLHGVGLLFSVVVFVIELTVH
YANVCLNNL

>DmelIR54a
MWTVITGIVLWAPVLVAGSAVDFIFRAAAEHSLSVIMIRIDYCPYNWAKDIFENQTIPVVVL
SDSETFIN
IRMFSRPLHVACLPGHELQKDLALLENFTSSLMDFPSQKKIVYISNNFSDPTRMDYIFETCY
HRRIWNIV
GLLASDEHRYFYRYHLYPSFRTEYRSLESSTIFDKDFPNMHGHPLTVMPDQWLPRSVLYVD
RRTGKQILA
GSVGRFFHVLSWKLNATLQLSKKVTTGRFLNATALKELSESFSVDVPASLTIMERVEQLAS
TSYPMEVTH
VCLMVPVARRIPIKDIYFILSSASNMFLAIVIVSSYGLALNLLRNMTHRDVRLVDFVLNDK
ALRGILGQS
FNLPLSRSFSTRLIFLMLGIVGLNVSSIFGAGLDTLMAHPPRQFQARSFAGLRRTKIPLVTTE
EDFPTWM
KLRVPMLVVNVSEYNHLRNGRNTSNAYFASRLYWNLFSEQQKRFTRELFIYSTDDCLWSL
ALLSFQWPQN
SLFTEPVSQLILEVNANGLYDFWVGMHYYDMTAAGLSGLEDPSLQLKEREHPTSLRIVDF
QWMWQAYGTF
MVIAILVFLLEVSWHRITSLFVSLVY

>DmelIR52a
MALGWSVIILGFIGQLSAQILNYTQSRDLELLEGSLFRVLSRLNLEEEYNTLLIYGKECVFH
SLLRKLEI
SAVTVPSGSTDYDWSFSTAILILSCGYDAENEENSYTLMKLQRTRRLIYLEDNSEPESVCM

RYSLKEQHN
IAMVKSDFDQSDTFYSCRLFQTPNYVEGHFFKDQPIYIENFQNMRGATIRTVADSLVPRTIL
YRDEKSGE
TKMMGYLGHMINTYAQKLNAKLHFIDTSKLGAKKPSVLDIMNWVNEDIVDIGTALASSL
QFKNMDSVWYP
YLLTGYCLMVPVPAKMPYNLVYSMIVDPLVLSIIFVMLCLFSVLIIYTQHLSWKNLTLANIL
LNDKSLRG
LLGQSFPFPPNPSKHLKLIIFVLCFASVMITTMYEAYLQSYFTQPPSEPYIRSFRDIGNSSLK
MAISRLE
VNVLTSLNNSHFREISEDHLLIFDDLSEYLVLRDSFNTSFIFPVSVDRWNGYEEQQKLFAEP
AFYLATNL
CFNQFMLFSPPLRRYLPHRHLFEDHMMRQHEFGLVTFWKSQSFIEMVRLGLASMEDLSRK
RNEEVSLLLD
DISWILKLYLGAMFISSFCFILEILRCGERCKRLWRCRW

>DmelIR21a
MSYYWVALVLFTAQAFSIEGDRSASYQEKCISRRLINHYQLNKEIFGVGMCDGNNENEFR
QKRRIVPTFQ
GNPRPRGELLASKFHVNSYNFEQTNSLVGLVNKIAQEYLNKCPPVIYYDSFVEKSDGLILE
NLFKTIPIT
FYHGEINADYEAKNKRFTSHIDCNCKSYILFLSDPLMTRKILGPQTESRVVLVSRSTQWRL
RDFLSSELS
SNIVNLLVIGESLMADPMRERPYVLYTHKLYADGLGSNTPVVLTSWIKGALSRPHINLFPS
KFQFGFAGH
RFQISAANQPPFIFRIRTLDSSGMGQLRWDGVEFRLLTMISKRLNFSIDITETPTRSNTRGVV
DTIQEQI
IERTVDIGMSGIYITQERLMDSAMSVGHSPDCAAFITLASKALPKYRAIMGPFQWPVWVA
LICVYLGGIF
PIVFTDRLTLSHLMGNWGEVENMFWYVFGMFTNAFSFTGKYSWSNTRKNSTRLLIGAYW
LFTIIITSCYT
GSIIAFVTLPAFPDTVDSVLDLLGLFFRVGTLNNGGWETWFQNSTHIPTSRLYKKMEFVGS
VDEGIGNVT
QSFFWNYAFLGSKAQLEYLVQSNFSDENISRRSALHLSEECFALFQIGFLFPRESVYKIKIDS
MILLAQQ
SGLIAKINNEVSWVMQRSSSGRLLQASSSNSLREIIQEERQLTTADTEGMFLLMALGYFLG
ATALVSEIV
GGITNKCRQIIKRSRKSAASSWSSASSGSMLRTNAEQLSHDKRKANRREAAEVAQKMSFG
MRELNLTRAT
LREIYGSYGAPETDHGQLDIVHTEFPNSSAKLNNIEDEESREALESLQRLDEFMDQMDND
GNPSSHTFRI
DN

>DmelIR100a
MATTLQLIMLALVGGTLGQANNTDHKQVLTSIVKQLEGGLELHLRTSEDGGNDLVQFLM

QEKSSIIISAK
QEEVPSRAKIMRHHFFIFDGVHQMQEIRTSLFNTDGFYILALENNTIEDDVLLMEFAADVW
LQHGHSRIY
YVQLSKKSVLLFNPFLQRLVVVQDSKTYSRIYKDLEGYHLRIYIFDSVYSSVIGDGENKVL
SVTGADAKL
AKTVARQLNFTADFVWPDDEFFGGRLANGEYSGGVGRAHRGEVDIIFAGFFIKDYLTTHIQ
FSAAVYMDE
LCLYVKKAQRIPQSILPLFAVHMDVWLCFLLVGLLGALVWLILRAVNLILGIEGVPDGSRAT
RISYFGAA
RRIFVDTWVIWVRVNVGRFPPFHSERIFVASLCLVSVIFGALLESSLATVYIRPLYYRDVNTL
RELDESG
QPIYIKHPAFKDDLFYGHNSEVYRRLDAKMMLVAEGEERLIEMVSKRGGFAGVTRSASLQ
LSDIRYVMTK
KVHKIPECPKNYHIAYVLPRPSPYLEEVNRIVLRLVAGGIVGLWTGEAKERAKWSIQRFPE
YLAELDVGR
WKVLTLSDVQLAFYALTIGCLLSAIVCMAEILLGRQRRLHSPK

>DmelIR85a
MSIQWLKHILLLAILVNLAGTRENHIPLDLKKSSIVMVKMSQILCKARIKVLFVYFENQTS
HEHTGQILK
EVTKCDISNQNTPLEAVKDDGILMYMVMITTNISQPLELSLIRKKSAAKHRSHVFLLVRDA
DTVSDAWMR
ASFRQFWKIWLLNIVILYWRDGRLNAYRYNPFMDNYLIPVDNKPNEVPTLEQLFPKTIPN
MQRKPLRMCI
YKDDVRAIFWRQGTILGTDGLLAAYVAERLNATMMITRPHSYNNHNLSSDICFLEVAKEY
VDVAMNIRFL
VPDTFRKQAESTVSHTRDDLCVIVPKAKTAPTFWNIFRSFGSLVWALILVSVLVANVFCYIL
KSEVGRVP
MQLFAGALTMPMTQIPPNHSIRLFLIFWLYFGLLICSAFKGNLTSMMVFQPYLPDINQLGAL
ARSHYHII
IRPRHVKHIQHFLTLGHKHESRIREQMLEVSDTQMYEMMRNNDIRFAYLEKYHIARFQVN
SRVHMHLGRP
LFHLMNSCLVPFHAVYIVPYGSPYLGFLDSLIRSSHEFGFERYWDRIMNSAFIKSGVKVVN
RRRGSGNDE
PVVLKLQHFHAVFALWLVGIGMACIVLAWEHLTHNYNLAVTKRRD

>DmelIR11a
MRFAILWLFSGCLLPGIQVGIWVVVRAQPTGRDVLLSRLGNQQNELNTRRLANASSYLTR
NYIANRINTL
VVREICVECPYELSERQRQLVDQILASLAPELSVLLHKGTAEETTWEYTLFVVNDHTAFTG
QVFIFPDEL
LEREFFCIVVVSEIQSRQFVRQTVGSIVKSNLQMHFVNVVVVAQLEDGTVGTYSYKLFKA
NCTPGITVRQ
INHFDRITGKPQQSMPDLYPVRNGHLGDCPFNVGAAHMPPHLIYKRHKDPPPASNVSIPAE

DLAGIDWDL
LQLLAKALKFRIQLYMPQEPSQIFGEGNVSGCFRQLADGTVSIAIGGLSGSDKRRSLFSKST
VYHQSNFV
MVVRRDRYLGRLGPLILPFRGKLWGVIIVILLLAVLSTCWLRSRLGLSHPIEDLLTVIVGNPI
PDHRLPG
KGFLRYLLASWMLLTLVLRCAYQARLFDVLRLSRHRPLPKDLSGLIKDNYTMVANGYHD
FYPLELTCRQP
LDFSARFERVQRAAPDERLTTIALISNLAYWNHKHPNISRLTFVRQPIYMYHLVIYFPRRFF
LRPAIDRK
IKQLLSAGVMAHIERRYMQYENKRKVASNDPVLLRRITKSIMNGAYRIHGLVIVLATGMFI
LELLAGRSN
GRLRRWMEWVHQ

>DmelIR47a
MRQIKLLVWLLVVGVVSSTEQLQFLKNFLEAVHKERSISTILLIQRKVHKNDFLHGLYPIF
WPIICLDET
KRVELVNNFNKDFLALVYMESEADTLLLSALAADLNHIRDARIMIWLQMSPSENFLDRIV
FQASKQKFLN
LVVIENTLKTRRFYPFPQPKVQVIDKPFEEKEIYPALWRNFMGKNAIAVPDLVPPRSFNSFD
PKTGHRRE
SGSIYNVFKAFTQRYNITMLLKWPLIRNTTQEEIIGKSVRGEIDLPITGQLISFRHPNGSRSQ
PLLGMTA
LSIAVPCGPELPMFDRFFLFYGLATPITITGYYVLLNTIEIILGTLSDRIKRHPRRKKILNLVLN
LRVFS
CILSLPTPQGNRLRSVKGQLTMVMSITGLILSCIVAAQTSTILTMKPQYRHIKNFQELSDSNI
TVVCNHL
NYLTIKQQMDPKFMAKFMQNIWIVNSIEQMKMIFDLNTSYAYQTFSYKKDPFTLLQMHTT
RKAFCRTPGL
DLVSGLAYTAVLEKNSIYALALQDYTLKAFSAGLVYYWAEESIRDLISTVGRTQFEKLPIVI
GYQSLKLQ
DYNVCWKILLIGGALAFCVFIVEVVVGLINRRI

>DmelIR51b
MCKVLTLLVVILLLALTNAAYNVTLLKSVLSLISTREPWINTPIFVGHNTQGGDLNDLIIWL
HQTMGVTS
LTMNLFLQPEHIRPLGHFKITRYNGIALFFCHDKHDIMWLTLDRNLRKLRRIRLIIILRNQRS
GSQGAIK
SIFNALWQYQFLNVLVLQRDQLYSYTPYPAMRFFKLDIHTEPLFPHAARNFHGYVVSTPAE
NDIPRVFHV
HDPLTKSRKVLGYAYRTFVEYLDHYNASLRLTNPDENLDPTTSVNMNHIVQLIIDGQLEISL
HPYVFTPP
TATKSYPLLIYPNCLIVPMRNEIPRHMYLLRPFQLYSWYILLFAVFYITGILYCISPKLNKSSW
PQRLGL
NFLDAISKILFISPPITIYRPTWRHLIIFLQLSVLGFMSTSWYNIELDSFFTTIVVGEQVNSMD

QLVHQQ
QRVLVKEYEINTFLRHVEPRLVEKVSRLLVPVNASEQVSALLSFNRSFAYPFTEERWQFFA
MQQQYAFKP
IFRFSSACLGSPHIGYPMRVDSHLETSLNHFILKIQDTGLLNHWVVSDFNDAMRAGYVRFV
DNVLGYQSI
DVDTLRLGWCVLGIGWILSALVFSCEYWHLYPWRFIA

>DmelIR20a
MLASLNRSTGLSAELLDLYGLVVHFLLSGEHTTLVYFNPAGLDCSWGVLWQRNLTAHPQI
VWQRNYSYPD
LYYQFNAKLLVLACLPMDSRAAIQLEILANSLSHLRTVVRLLIEVAGPDQVTLARQYLSFC
LRRSMLHVE
LYFRDYHHSLILYSFRAFPSFELVMRWISVGQGVKLFLHKLDDLRGHRLRVIPDLSPPNTFF
YRDARGDN
QVTGYLWDFLATFAGRLNAGLEVVRPSWRAGSASDSSYMLEYSAKGLIDVGLTTTLITK
WNLWAIHQYTY
PLLVSSWCTMLPVEKPLATPDLFGRIVCPTLAMTLLLIILVTWLVFRQLRCLTRLKNSRPARI
VPHLLTL
LLLTTCSAQLLSLLIFPPYHVRIASFEDLLRGDQKILGMRNEFYNFDGAFRARYAGVFYLID
DPNELYDL
RNHFNTTWAYTMPYIKWLVIKTQQRHFSKPLFRWSKDLCFFDFMPTSVIVAPDSIYWESIK
DFTFRIHQA
GLMKHWIRKSFYDMIKAGKMSIKDYSDLETLKPLNIGDLEIVWRVCGAAIAVASAIFIMEL
LYFYINVFF
NSL

>DponIR21a
MLLYALVALLAPIHCTVLESTDDIYTSVTKRALQKSHEKPRIQKLSEFFLETDYYHEDPDKS
LIGLLNSI
ANQYLSECTTVILYDNYTENTELVFLKKFFRTYPLTYVHGSIPSDYHIQIGELVNKNDKKCV
HFIIFIKD
VMRCQDVVNKRNERVVVVAKSSQWRVQEYLSSEFSQEIANLLVIVKSDKYGPQKAETPFI
IYTHRLFVDA
LGSSQPIVIASWSRGKFSSNASLFQTKLNHGFSGHRFIVATAHQPPYVIKKQRSDKDEFEYS
GIEVKLVE
LLAKMYNFSTDYKETADIKVLGSGEAVVKAIKAGNVNLGIGGLYITENRYNAGIFHWHSE
DCASFISLAS
TALPRYRAIMGPFHWTVWLGLIAVYLAAILLFSYSDKLTLKHLIRNPIEIENMFWYVFGTFT
NCFTFSAG
TSWTRAERDITKLLVGIYWLFTIIITACYTGSIIAFVTLPIYPAVIDSIDQLTGKRYQIGMLNK
GGWPSW
FQNVSDETSERLLRKVDYVPDVESGLRNVTKAFFWPYALLGSREELQFIVKTNFSLGSKK
SMLHISQQCF
VPFKVGIALPHHLVYSEILAGGIQMILQSGLNIKMKNDIEWEMLRSSTGKLLAANSRSGTL

TILSRDDRA
LTLDDTQGMFLLLAIGFLAGGGVLISEIFGGCFNLCKKIDNSRATSSNSSIPSNPRFHERQTI
RERNRSI
SLASFQQRHNSIQSEIAFEKAQAEEHHQGGLVECQIHGTTPDQNSQGVVLEETNADIDYNE
QISKLFEQA
LGEETCGSSHSGHNQKLT

>DponIR8a
MHVLGLFGAVACVLSVRGQQFKIVTLHQPDQAAEVKYFENAFLKVNKDEEIAFLDVLLN
EDESGHYKQIC
DALSTGFSLILDFAWSGTEVAQDLTSNMSLPYLHVDVSVAPFLVLLDSYLDSRNSTDVVVV
FDKEEYIDQ
SLYYWLDSVRLRLVMADALNRSTASKIESIRPIPHSFAIVASAKNMNKLVSQALNEDLMSL
SDRWNLVFT
DFETGIFDKSLFQNQTPSLMYLKPELCLDLSIQSRCPSNFVLKEQFLYWLAWGLSRLAKMA
AEESLEFPE
KEFQCGKTTFSEDTKERLGDMLDSIVADNSNVLSLTGRSVKVAVRGNVEKMINGSFQTIA
QYTNGKLTPE
PGKQIDPIRAFYRIGITHAIPWSFKAQNLQTGEFYWTGYCADFAQKISEVMNFDYVFVEPA
TGTFGEKVN
GTWDGIVGDLAVGETDIAITAVIMTADKEEVIDFVAPYYEQTGITIVMRKPVRKTSLFKFM
TVLKLEVWL
SIVGALIVTGFMIWFLDKYSPYSARNNRKAYPYPCREFTLKESFWFALTSFTPQGGGEAPK
ALSGRTLVA
AYWLFVVLMLATFTANLAAFLTVERMQAPVQSLEQLARQSRINYTVVQDSETHMYFINM
KFAEDTLYRMW
KELTLNASTDDTRYRVWDYPIREQYGHILLAINDSNPVANASEGFRITNEHLDADFAFIHDS
SEIKYEIS
KNCNLTEVGEVFAEKPYAVAVQQGSHLQDDLSKVILDLQKDRFFEQLQAKYWNDSAKGD
CPSTDDNEGIT
LESLGGVFIATLFGLALAMITLAGEVLYYRRKGKKIKPKKRKAKSKNLPLNTAGLKLSKLD
GQSVDMFNV
NKTVTIGTTFKPVNLKENLTKEMETVHISHISLYPKARNRIPRVE

>DponIR25a
MKNNNIVAGGFFSIFLLNVADICGQTTQNINVIFANEEGNFVADKAVTVALNYIKKTSKLGL
SVDLRRVV
GNKTDSQNVLDSLCAAYQQMLDDNNPPHLVLDATRAGLASETVKSFTAALGIPTVSASYG
QQGDLRQWRN
LQPNEEEYLVQISPPGDIIPEMVRTLVLNQNITNAAILFDDSFVMDHKYKALLQNVATRHLI
DEINEDVN
KIPDHLESLVKLDLKNFFVLGSLQTIKNVLEAAEKKSLFNRMFAWHVLTKDPDDLKASIKN
ATIIFAKPI

VNNLYQDRLRNIQTTYQLSSVTPEIEAAFYFDVALKGFLAVKEMLLDGSWKKNNVTNYV
TCDDYEPKYSP
KRFNLNLRSYLQKESSEPPTYGPFAIESNGMSFMEFSMALSAVYVRSGASDKSLPLGTWH
GGFNNNMTLL
TPKDMKNYTADVVYKVVTVVQKPFIYRDDTAPKGFKGYCIDLIDEIAKILHFDYEIDAVAD
GMFGNMDEN
GKWNGIIKDLIEKRADIGLGSLSVMAERENVIDFTVPYYDLVGITILMKMPETPTSLFKFLT
VLENEVWL
CILAAYFFTSFLMWVFDRWSPYSYQNNREKYKDDEEKREFNLKECLWFCMTSLTPQGGG
EAPKNLSGRLV
AATWWLFGFIIIASYTANLAAFLTVSRLDTPIESLDDLSKQYKIQYAPVNGSSTMTYFQRM
ADIEAQFYE
IWKDMSLNDSLSDVERAKLAVWDYPVSDKYTKMWQAMKEAGLPPDLDTAIERVKKSKS
SSEGFAYLGDAT
DIKYLEITNCDMAIVGEEFSRKPYAIAVQQGSPLKDQFNTAILQLLNRRELERLKERWWNK
NPEKKQCEK
ADDQADGISIQNIGGVFIVIFVGIGLACITLAFEYWWYKYRKNTRVTNVAEAPNSRHHKV
GGVQKGFPRQ
FEGESDMKITKLYPKTKF

>DponIR75x
PAAKSIRNVIYSRRRAFLDKVRLHKAILEENLNMRVVFAKARVDAFENINTKRYEKFTYFL
DMQCPDSAD
ILDKASNVNRFGKFEFIYSWYLLSKVNQELEDIFHIFTRFKTRMDMDVKVLAVDDFGRFE
VSEIFNPGIN
VGLTTRRIGKLQNGSVIIDKNWSYYESRMNMTGVLIRSANVIRYPFTTSFDEYMTDPKLM
KYDIYSKFHY
QLFQGLVHIHGFEYNTSLPFSWFGNTSSGEDGGLAKMLWDDTIDISSAGCILRLLDSDRIDF
YDYIMPYY
KFRSCFFPQSRSRKAELLRSIKAV

>DponIR76b
MGLMEVVLTTLATLCFNSTCVDQDLINASKQRLAHLKEELKHETLTVTTLKNGPLSGYEI
VNNTVIGTGV
AFEILNIVQREYGFKYNVIVPDHDSFEPVNGGEGGVRNMLLNETIDVAVAFLPQQYTDVVS
YSRSLDTAQ
WVVLMKRPKESASGSGLLAPFTATVWSLIIISLLGVGPILWLTILLRARMCKEDHDIVFSLP
SCMWFVYG
ALLKQGSTLNPRTDSSRILFSTWWIFITILTAFYTANLTAFLTLSKFTLPISEPKDISRKHNKWI
TNRGN
GIVEQLYLSKKYANGDGNSLFEEIGMPQWEPDVDEDTMLSTYVIKQNMMYIREKTVLESI
MYEDYKVKTK
ADVEESKRCTYVITKFAVCVFPRAFAFRPGFKYKELFDFTIQHLSESGITDFQQRKSLPDTTI
CPLDLGS

KERRLRNSDLAMTYMIVGGGLIISTIIFAVELIIYYAKMHCFNKKSHVNNNNTLVTQSNNG
LFVKNHQHQ
GNFRASKQFVSPPPSYHTLFHPPNLTNGEYKNKTINGRQYWVFNDKQGMTSLIPQRTPSA
LLFQFTN

>DponIR75s
LVVLYFLRKWEFAKIKQQELLHKTAYPDVVFITIGAVCQQGAATLPHSVPGRIATLWLFVSL
MFIYVSYS
ANIVALLQTSSNSIRTLEDLLKSRIPIGVDDTIFNHFFFTTTQEPTRRALYEKKVAPPGKRPNF
LSIEEG
VRRMRQGLFAFHMETGAGYKIVGETFTEDEKCGLQEIQFLQVVDPWLAIQKNSSYQELL
KIGFHLLRETG
IQQRENNLIYTKKPSCSSKSSTFFSVGLVDCYPAVLIFAEGLAAGLALFLLELYIHKKFSNQ

>DponIR64
MDNGIDVQNHRERTSDTSWSLSTICTFGVFCQQGIISVPRCLGGRTSAIVALWCGLVIYQFY
SASLVSFL
LNVPVNVLSTVQDILDNGFDVGYERVLYAMSLLKAATNPAAQEVYRRVSNKNQTGYLRR
EQGLELVKNGR
YAFHVELVTGYPFIEQHFDESMICELKEISLFPAMYMYSGYQKWSPFREFLDACLHRLEEN
GVVSRELYF
WHPRKPQCMRSRSTI

>DponIR75q
MNTLYFGFFMWFALICGGHCGVHADDVLLLIEDLLDFYRFSKKVYTHVCWDKELQLHLP
RFALHAKYHEF
QSTKSQVVILMDMSCKGADFFLENMQKRNLLTPRVILLLINPAALTPHYFPVNSNALFLKP
SGEGFAISK
IYSGVSNVTIGTWTKTHRYIERPIAKKKLRATNLKVCYLVGDKHEGLESSEYQPVAEATTK
LNRVLLEDG
IRMINSTKTDAFQIGSGEPELVNDLIAGKCDIGGTPLALTAEKIGRLDVLAKTLREDKTFVF
RAAPHSYI
SNVFTLPFDSYVWCSCFGLMGVIFLIVHLVVCWEWKDPVFKLNLQPNISLRPNPVDILLME
VGSAAQQGF
EAEPRSNSGRIVFMSTLISFMFLYTSFSASIVALLQSTTDSLNTFDNLFQSRINVGFKQNISLD
FFNDLK
HQPNRADYHGKLKEPQFFTLEEGVKRLQNDFFAFYAETSEVYRYINRWFQESEKCSLREIP
FKNTHINHW
LFMGKNSQYRDALRIGMNSIQERGIRSREYKRLFPLKPFCDSIGGNFESVGLVDSYGAFLI
MVYGVALSS
LLFLLEHMSLQYNLSTKAGRIRNRIFNSIHRGDE

>DponIR41a
MGSEMKILETFSQYVNASISPVINQADYWGEIWNNWSGSGLMGNLVEDKADIGAAALYT

WEFAYEYLDLS
KPTVRTGITCLVPAPKLSAGWLTPFRVYSLEAWMALIGTLALSFLALYALNKLQISVKPQLK
SKHHINQL
KGKLLSKTLMSVSKPFVMQSITNKEMAQGNLAKYLMGLVFLSTLVLSTTFDSGLATIMTV
PRYDNPINTI
EELAESGLPWGGTQDAWILSINNSLEPNLMKLVARFVAHSEANLRKYSLGDQFAFGVERL
PNDNYAIG

>ItypIR25a
RGNREKYKDDEEKREFNLKECLWFCMTSLTPQGGGEAPKNLSGRLVAATWWLFGFIIIAS
YTANLAAFLT
VSRLDTPIESLDDLSKQYKIQYAPVNGSSTMTYFQRMADIEARFYEIWKDMSLNDSLTDV
ERAKLAVWDY
PVSDKYTKMWQAMKEATLPPDLETAVERVRKSKSSSEGFAYLGDATDIKYIHMTSCDFVV
VGEEVSRKPY
AIAVQQGSPLKDQFNSAILQLLNRRELERLKEQWWNRNEESKQCETSDDQQDGISIQNIGG
VFIVIFVGI
GLACVTLAFEYWWYKYRKNSNITNVIVSDPKHRRVAGFPKDVGGKANEGELALRPGKLY
VKPKY

>ItypIR76b
QSTGVSYTKLFDFTIQHLRQAGIIQFKHRELLPDAKICPLDLGSKERRLRNSDLAMTYQIV
GGGLIISS

>ItypIR64a
LVKLGLNLVKQGHYAFHVELVTGYPFIRKHYSESMVCELKSVSLFPSMFMHANYQKWSP
FKDLLDVCLHR
LGENGVINRELIFWHPKKPECIRSSSTININTGLESFYPALVVLLLGILASLNILLLEILWFKY
QKRQIL
PYTE

>ItypIR75s
SKFVFRQPKLSYVTNVYTLPFVSKVWYSTVILTVLMALALYGLMKWEHAKNHFLLEKSL
AGRGRQNQTEL
RDSIKDVAFVTIGAV

>ItypIR75p
QSRFIAEREMYEENAPDFLDIVLMQIGVVCQISYSFKPRSTAAKIATLSLLVGFVYIYNAFC
ARIVILLQ
STANNLNNYKDLYYSKIDMGVEEAPYNKYYFSNPNNRANEAWRKLIYETKIMSKNKHPV
FYSTAEGLKLV
KQSYFALHVEYTTATDVILATFTNEEMCAVRVIESIYKEDVPYISCPVNSTFTEYLLIGFHRL
FETGLHS
REARRKFSKLPKCIGRNSIFVSVGIIGCYFAVEVFLVGVILSLVIFAVELV

>ItypIR68a
VRGDTTQFKDEGNQSVSNKIRANIMLSNATELFSGLEIEILDTLSKVMNFHCELYEPDRAD
TELWGRKQY
GGVFTGLLGELFTSKADMALGDLYYIPFILDVMDLSIPYNTECLTFLTPESLTDISWKTLVLP
FS

>LdecIR25a
MCTKKSLLIFQFLLFGYCEGQTIQNINVLFVNEEGNEVADKTLDVVMTYLKKNNKLGLSV
DVRRVVGNRT
ESNAFLESLCSTYNAMLETQTYPHLVLDTTMTGLGSETVKSFTQALALPTISASFGQEGDL
RQWRNIDEN
EKDYLIQICPPADIIPEIVRTLVLNQNITNAAILFDNSFVMDHKYKSLLQNVPTRHIITPIKTG
NQVVEQ
LTQLRKLDLVNYFVLASLKNIKRVLDAADSVNFFNRKFAWHVITQEDGEMKCVCRNATIIF
VKPSPNAAH
QDRLGTLQRTYQLNTKPIISSAFYFDLTLRSFIAIKEMISDGAWKNSVTNYITCDDYNGDNS
PNREGLNL
KKYFSKEISEPPTYGPITLVSNGLSYMEFQMQLTSVGVREGASDKSTVLGTWSAGFDNNLT
IADPQVMVN
LTADVVYRIVTVEQKPFIFRDESSPKGFSGYCIDLIEKIADILQFDYEITAVDHFGTMDESGK
WSGVVKE
LMEKRADVGLGSMSVMAERENVIDFTVPYYDLVGITILMKLPETPTSLFKFLTVLETDVW
LCILAAYFFT
SFLMWIFDRWSPYSYQNNREKYKDDEEKREFNLKECLWFCMTSLTPQGGGEAPKNLSGR
LVAATWWLFGF
IIIASYTANLAAFLTVSRLDTPIESLDDLSKQYKIQYAPLNGSSTQTYFERMANIEAKFYEIW
KDMSLND
SLSEVERAKLAVWDYPVSDKYTKMWQAMKEAGLPNTMEEALEKVRASKSSSEGFAFLG
DATDIKYLEKTN
CDLTVVGEEFSRKPYAIAVQQGSPLKDQFNTA

>LdecIR21a
RALNGDSIFEGIELRLVNMLSKKYNFTTDYKEATEITQVGSAEAVTRIVEKKKANIGISGM
YITQDKLNR
VGVSQWHSSDCAAFVSLSSTALPRYRAIMGPFQWTVWLTLIITYLLAIFALTYSDKHTLRH
LLKNPEEIE
NMFWYVFGTFTNCFTFTGKASWSKANKLTTKILIGFYWLFTIIITACYTGSIIAFVTLPVSPT
TMDSVSQ
LLSGSYQIGILGLSGDNTNLFCYIISYSRHHQKMANEVLPKCCENNFMRIKYTEHGVKPEK
KAHHTYTVS
KRSMLHISSQCFALFNVALVFPKRSVYGEILNQGIQSIIQTGLLSKMKSDVEWEIMRSPTGK
LLAANSRT
TGLKILSYEDRALTLEDTQGMFLLLGAGFLIGAAALTSEWFGGCLKLFKRKSRPSTDSSIAS

NPRIQTGR
NRRN

>LdecIR40a
MQERVRRKLIIMNKNVQKAKCFLFQYFEYLSYQTENHLESYNIFFGTHRLYEHLLLEIASR
NFIRRNIIY
IFYWGQSLFSRYFLRNIKYCMKIYAITNPRNDTYRLFYSQATSHREHHLEMVNWWNHGK
GIFNHPTLPTT
KSVYKDFKGKILHVPVLHRSSFLKSQNRVYAYFDPPERTQGTADSETGFFNGIIGIVWRRE
ADLFLGDVE
ITYEKSNFVEFSFITLADSGAFVTHAPSKLNEALALLRPFQWQVWPAIGLTCFIVGPVLYLII
ALPNFWQ
PRFRVRSHSRMFFDCTWFAITILLKQTGREPSSSHKSRFFIILLSISATYVISDMYSANLTSLL
ARPGRE
KAINNLYQLEHAMETRDFKLFVEKHGSSYGLLENGTGVYANLWNLMRKKQGNNFLIESV
EAGVKLVRHST
TNAVMAGRETLFFDIQRFGPGNFHLSEKLNTAYSAIALQLGCPYIDEVNRMXVYVFSFFL

>LdecIR93a
MYLNLPLESLPFIDKNHTSVSSLTLYLKKTFLFVQGGRKCQIEEIIEAFTRALDEAVQEESDT
ASQIAEE
EWEAIRPTKVERREFLLGKIKKYISKNGICDNCTFWEMKTGETWGQEYQNLSRNITSQLIP
VGTWRPSDG
ATMIDELFLHIAHGFRKKILPMVTFHVS

>LdecIR NMDA1
MWKLLIYFCVYFGFNMVLLLANQRDWNSPNPTEFNIGGVLSSNESERYFKETIDHLNFDS
QYVPKGVTYY
HTAILMDPNPIRTALNVCKHLISSKVYAVVVSHPLIGDLSPAAVSYTSGFYHIPVIGISSRDSA
FSDKNI
HVSFLRTVPPYSHQADVWVEMLKYFNYKKVIFIHSSDSDGRALLGRFQTTSQSLEDDVDI
KVQVESVIEF
EAGFDSFKEQLLEMKNAQARVYLMYASKIDAKVIFRDAAALNMTDAGYAWIVTEQALEA
DNVPEGIIGLK
LVNATNEKAHIRDSIYVLASALRDMNQSKEITEAPKDCDNSGSIWETGKDLFNFIRKQVLI
NGETGKVAF
DDQGDRINAEYNIVNIQRKKKKVVVGKYFFNKESNKMHLNVDEKSILWPGRQNTKPEGF
MIPTHLKVLTI
EEKPFVYYVRKLIDIQQEGCSAEEIQCPHFNTSDDLNDVYCCKGYCIDLLKELSKKINFTYSL
SLSPDGQF
GNYLIRNTSGSAKKEWTGLIGELVGERADMIVAPLTINPERAEFIEFSKPFKYQGITILEKKP
SRSSTLV
SFLQPFSNTLWILVMVSVHVVALVLYLLDRFSPFGRFKLANTDGTEEDALNLSSAIWFAWG
VLLNSGIGE

GTPRSFSARVLGMVWAGFAMIIVASYTANLAAFLVLERPKTKLTGINDARLRNTMENLTCA
TVKGSAVDM
YFRRQVELSNMYRTMESNNYDTAESAINDVKQGKLMAFIWDSSRLEFEAAQDCELVTAG
ELFGRSGYGIG
LQKGSPWSDDITLAILDFHESGFMESLDNKWIFQGNFQQCEQFEKTPNTLGLKNMAGVFI
LVGAGIVGGI
GLIVIEMAYKKHQIKKQKRMELARHAADKWRGCVEKRKSLRASSTAQRRIKSNGVNEAV
TISHVFDKFQR
IGQFYGPERTWPGDSDIRQRRIDDVGGGVQPVPRYLPAYTQDVSHLIV

>MaltIR1
LNAEPVITAAFYFDLALRSFLAIKEMISDGAWKKNNVTNYISCDDYDGKNSPKRMNLNLR
KYLSKESTET
PTYGPISVTYNGQSYMEFQMQISAVGVREGASDKSTTLGTWPAGFDNNLTLVEPQ

>MaltIR2
TVLENEVWLCILAAYFFTSFLMWVFDRWSPYSYQNNREKYKDDEEKREFNLKECLWFC
MTSLTPQGGGEA
PKNLSGRLVAATWWLFGFIIIASYTANLAAFLTVSRLDTPIESLDDLSKQYKIQYAPLNGSS

>MaltIR3
GSSTQVYFERMANIEARFYEIWKDMSLNDSLSEVERAKLAVWDYPVSDKYTKMWQAMK
EAGLPNTLDEAV
QKVRDSKSSSEGFAFLGDATDIRYLEATSCDLTVV

>MaltIR4
SGYRKNGKELVGKGIAFDIIHILQEKYNFNYTIVAPQDNVFLSSTKKLGAKDMVVNGRAD
LIAAFLPVMN
SFHEEIVFSRRFDTAEWVVLMKRPRESAAGSGLLAPFTTPVWILIILSLLVVGPIIYLLILVQ
AKLTRDD
NNKVYPLPACVWFVYGALLKQGTTLNPTTDSSRLLFSTWWIFITILTAFYTANLTAFLTLSK
FTLPINE

>MaltIR5
TVSASFGQEGDLRQWRNIEENEKEYLIQICPPADIIPEIIRAIVLNQNITNAAILFDNSFVMD
HKYKSLL
QNVATRHVITPIRGNGDQKVIQDQLLQLRKLDIVNFFVLGSVANIGTVLNAGNEINFFNRKF
AWHAITQD
DGEFKCNCKNASVMFVKPSPNTA

>MaltIR6
IDAISKLNYILVHHLVEFVNATSIKIFKDTWGYKNATTSLYSGMIGDLQMGNAELGGTPSFF
TIDRIDII
DFIAATTPTFMKFIFKAPPLTYVTNVFTLPFDTYVWYCSFALIAVILVVVYVIVV

>MaltIR7
EWRDPVFRNKSVTVYSLRPNIFDVFLMEIGAITQQGYDAEPRSNAGRIATIFTFIALMFMY
TSYSANIVA
LLQSTTESIRTLEDLLTSRISLGVEDIIYAHYYFENAQEPTRKAIYEQKIAPKGQKPNFMTAR
EGIERVQ
QGFFAFHIELSTGYKIVNEIFQESEKCSLKEIVYINLIEPWLAVKKNSSYKEIFKVGLKKIQES
GIQSRE
V

>PbreIR
MRTLLVLLTAIVSVKSYLDDCSVGLLQDDDDQIKKIAFMAESEILGFENMIEPVTAFRSYDT
LTVTCHMI
DSGVIALFGPQSEDNAQIVESVCNNKDIPHMETRWNDQPGKLTNTLNLYPHGPTFSKALA
DLVEARQWDT
FTVFYEDNESLARVSEILKRERELSISIKQLDAMGTGNYRPILKEAWRSGHTRFVIDCKVD
NLIQLLQQA
QQVGMMTSRYYYIITNPDLQTVDLEPYKYSDTNITGVRIIDPEAEATKRLVGMINQKQIDL
ELDPSHAQI
IAPHELRVETALTIDGLNLLRMSLDHLPEHTRCGPEIACQDKKGWIHGSTALNYMKMTSF
DGITGLVKLD
SEGHRTDFNLDIIELSEDGLVKVATWNLTDGIKFLVGDDDYDLHDLSFVVITALTEPYGMLT
DSQVSLVG
NARYEGFAIDLIHELSLLEKFNYTFIIREDKSNGSKNKVTGKWDGMIGDLIDKKADLAITD
LTITSEREE
AVDFTTPFMNLGISILYQKPQKAPPNFFSFAEPFAFEVWLWLGGAYFIVSISLFIMGRLCPSE
WTNPYPC
VEEPEFLINQFSLRNSFWFTIGSLMQQGTEIAPIAYATRMTAGIWWFFTLIMVSSYTANLAA
FLATENPD
IPFNDVYELVEKASKSNIKYGAKNKGATMNFFRDSNNDDFKKIYNYMIANEKQVMVGDN
KEGVLRAEREP
YAFFMESVSIEYEIQRHCNLSKVGDLLDEKGYGIAMRKDSPYRHKLNTAVLKLQENGKIS
DLKRKWWEER
KGGGQCSGEVESQEAKPLTLKNVGGVFWVTVGGVAVAVVLVFVEMFLHVMKESIKHKA
AFWAELSEELKF
YMKFKGLVKPVRLKKGDSKSPDDSDKSEKSEQHEMQGEANGRSYGFLPELIKQPLE

>TmolIR6
MSILEYITFAVLSVLSLSINTKGQTTQNINVLFVNEEGNLVAEKAVDVAVNYIKKNNKLGVN
TEPVKVIG
NRSDASALLDSLCSSYNDMLASSMFPHLVLDTTMTGMASETVKSFTAALGLPTISASFGQ
EGDLRQWRNI
DENEKEYLVQILPPADIIPEIVRTIVLNQNITNAAILFDNYFVMDHKYKSLLQNVATRHVIAP
IKEPEKI

GDQLRQLRKLDIVNFFILGSFENIKRVLDAADSVGYFNRKFAWHAITQDKGDLKCNCRNA
TIMLAKPVID
AQYQDRLGLIKTSYQLNAEPEIAAAFYFDLALYSFLAVKEMIADGAWKRNNATNYITCDE
YDGKNSPKRI
GLNLKKYFNKDTSETPTYGPMSIISNGYSFMEFNMQLSAVGVRESSSDKSIPLGTWKAGY
DSNLTLLDPQ
NMKNYTADVVYRIVTVEQKPFIIKDETAPKGYKGYCIDLIQRISEILNFDYEITPVSDQKFG
NMDENGKW
NGVVRELMDKRADIGLGSMSVMAERENVIDFTVPYYDLVGITILMKLPKTPTSLFKFLTVL
ENEVWLCIL
AAYFFTSFLMWVFDRWSPYSYQNNREKYKDDEEKREFNLKECLWFCMTSLTPQGGGEAP
KNLSGRLVAAT
WWLFGFIIIASYTANLAAFLTVSRLDTPIESLDDLSKQYKIQYAPLNGSSTMTYFQRMANIE
AKFYEIWK
DMSLNDSLSEVERAKLAVWDYPVSDKYTKMWQAMKEAELPNTLDEAVDRVRDSRSSSE
GFAYLGDATDIR
YLEITNCDLQMVGEEFSRKPYAIAVQQGSPLKDQFNTAILQLLNRRELERLKEKWWSRNP
EAKKCEKQDD
QSDGISIQNIGGVFIVIFVGIGLACVTLAFEYWWYKYRKGSKVVDVQGAPHVKHPDMMS
KINDGFNAKVN
KLYPRSRF

>TmolIR5
MGLIEIVLATLCLNSTCLLDEDLTYQDVSVNTRKMQFAQLAEELRQENLIITTLKNDRLSGI
LKDNGTLT
GTGVAFDLLNILQKKFQFNYTIVLPKTNVWGSQKTGILNMLKEKRANLSAAFLPVLTQYS
TDVSYSPSMD
TGEWVVLMKRPQESATGSGLLAPFTLPVWLLILLSLVVVGPVIYFIIYLQSKLCPDDQNKV
FPLPACIWF
VYGALLKQGTTLNPMTDSSRLLFSTWWIFITILTAFYTANLTAFLTLSKFTLPITEPKDIGAK
KYKWVTT
QGNALEDTVTTDGSLTDIGNMLGKPYRYLDQSDRTILKQYVDRRDMMFIREKPIVEYVM
YDDYKEKTRNQ
IEEPKRCTYVITRFSIVSFSRAFAYSKDFKYKMLFDSTIQHLVESGIIKYKLREELPDTEICPL
NLGNKE
RQLRNQDLMMTYEIVGGGFIISAVIFVIELAVKRSHKPKQAKTSGKSNLLLNQKHTIHVNI
NNNYEKFGQ
PPHNAKFITPPPSYHTLFNPPNNENLKKKNINGREYWVYDSLGGGTKMIPMRTPSALLFQ
YTH

>TmolIR4
MVKNLLPYKCVVLLSDDIYNGIYSSAWYRRFGLYMTFIVVNVDEYEDLLSPYETTQAALS
TAKNEGCQMY
IFLVSNGLQVARLLRFGDRYRVINTRAKFVILYDNRLFEKNLFYLWKRIINVIFIRRYGGQKS

GDDKNMP
WFEITTVPFPSQITSILVPRRLDIWTKSKFRKGADLFRDKTFDLRNQTFKVAAFGHIPGTTK
NMKVKSFR
AVLGNFSGVETEILQTVATAMNFKCEVYEPVNADAELWGGKQTTGKYTGLIAEMVSTRA
DIALGDLYYTP
YILERMDLSVPYNTECLTFLTRESSTDNSWKTLILPFKPTMWAAVLICLAISGSVFCALARF
HETISRIK
TEKSRNLDLYNKKKKIITLSMCPELEKLDPNVKYTMMKEQYNPPRFEGQAMGLYQFSQPF
NSMLYTYSML
LLVSLPKLPTGWSLRMLTGWYWLYCLLLVVAYRASMTAILARPTPRVTIDTLQELVNSRLK
CGGWGDINR
EFFKASLDPTTKLIGENFEV

>TmolIR3
QSHFLLESVEDGVKLVKNSRNKAMIAGRETLFFDIQRFGASNFHLSEKLNTAYSAIALQLG
CPYIEEFNK
ILMAIFEAGIITKMTENEYENLGRQKEVTSESENDLIGGNKKENRRAAKVSEDNEKLQPIS
LKMLQGTFY
LLCMGNIFSGLILVAEILVYKHRKKYIRKKRKNRCVYLKKMKNCIVSRVTHCANSIRRAYR
RAMNEAFIA
TLEYLE

>TmolIR2
MDSRTFLICLIFIEISTCKIVLPMINDFVEHYNKTQIVLGFVCRKNDALELVKHFSKKLHQV
KAVSVDTR
ELPYPIPPTYVTFVLDGQCSGAKHLLLRADAERLFATPFKWIVSYDYSYDVADLIENFTEL
NLLVDSDVT
VAIMENRETFSLQKIYKRHVNGSMLVENIGNWTKRHGFVDNGYEKIIYKRRRDLKKTILN
SCIVITNNDS
LNHLTDKRDIHIDSIAKVNYVLVEHLSDTVNAVLNYSVRGTWGYKNNESKWSGMIGELTR
NEADIGGTAL
FLTSDRIEIIDYIAMTTPTRSKFIFRQPKLSYVANVFTLPFDGLVWASTCALLLIMAMVLYVV
VKWEWKK
KKYVEIANESNDIEIPNSWVEVTFTTVAVLCQQGSSSIPFSIPGRITLIFLLVSLMFLYTSYSA
NIVALL
QSSSNSIQTLQDILHSRLDVGVDNTVFNHFYFPNATEPVRRALYLQKVAPPGQKPKFYSIDE
GIRRMREG
LFAFHVETGPGYKFVSETFQEHEKCGLQEIQFLQVPDPWLAIQKNSSYKKMLKVGLRLLQ
EIGIQGREVG
LIYTKKPQCLSRGSSFISVGLVDCYPAAVVLAGGLGAAVMVLLLEIYVNHRLKYYSRKWR
RNKRIRQ

>TmolIR1
KVDEALYFWIDTTLLRMVISEDLDKTTANRLKAIRPIPNNFAIVATTSNMEELFQTAIDENL

VTLPERWN
LVFLDFQYDNFNKQLIKNLPINLLYVDKSICCMFLKSETCECPHDFSLQESFLILATDIIKRVL
YAMVTE
NSELTTNLSCEDAHYNAQSTNRFYELLGQEVDQNHLVVKENNALRVDFNGSIEVGVGESS
EKVASYNFKR
GVTVLEGKKINPITAFFRIGITHALPWSYKETDSSGKTFWTGYCVDFTDELARLMGFQYEF
VEPKSGTFG
KKRNGVWDGVVGDLASGETDLAITALIMTADREEVIDYVAPYFEQTGITIVMRKPVRKTS
LFKFMTVLKL
EVWLSIVGALIVTGFMVWFLDKYSPYSARNNKEAYPYPCREFTLKESFWFALTSFTPQGG
GEAPKALSGR
TLVAAYWLFVVLMLATFTANLAAFLTVERMQTPVQSLEQLAKQSRINYTVV

>MaltSNMP1
MKLPVKLGIGSSVLFVFIVLVGFVVFPKLITSKIKGMITLKPGSEIREMFLKVPFGLEFRVYI
FNVTNPM
EVQRGQAPSLKEVGPFCYEEWKKKVSVQDVEGDDTILYNATDTFIQKMWPGCLSGNEEV
TIPHPMILGVV
NTVAIQKPGALTLVNKAIKSIYSNPTSIFVTAKASDILFNGIIINCDVKDFAGKAICSQLKEAP
SLTHIS
ENELGFSLLAPKNATPGKRIKAYRGVNNFKDVGRIIEYDGADKIDVWPTEECNAIKGTDG
TIFPPLLEED
EGLASFAPDLCRSLVAEFQQKTKYDGIPVRKYSATLGDMSTNENEKCYCPTPETCLKKGIM
DLYKCIGVP
IYATLPHFYEADEGYLKGVKGLKPEKSKHEIVILFEGMTGSPVFAKKRLQFNMPLKANPKI
ELFNNFTET
ILPIFWIEEGVELNNTFTKPLKDLFRMKKIVKVSTWVILLGSLLGLSAAGYLFFKESGKADI
TPVHKVRP
SDGRKTISSVSGNDLEGHENHAMSKTEMI

>DponSNMP1a
MNFPMRLAIGSACSLLFIILVGFVGFPKMIKGKVKDMVNLKPGMEIREMFVKVPFPLSFNV
YIFSVLNPA
EVQGGAKPHLKEMGPFCYNEWKTKINVEDNEGDDTISYDPVDTFENAKRPKCLSVDTLV
TIPHPMILGMV
NTILRQKPGALTLANKAIKSIWSNPSSLFITVKAQDLLFDGVVIHCGVSDFAGKAICTNLKA
EPSLTHLG
EDDLGFSLMGPKNGTAGKRIKAFRGTQDFHKVGRIIEFDGKSKLDVWNNSKCDTIVGTDG
TIFPPMLKKE
EGLASFAPDLCRSLIAQFDKHDKYDGIPVSSFFASLGDQSKNPAEKCFCTTPETCLKRGLM
DLYRCAKIP
LYVSLPHFYDSHESYLKGVKGLKPDVEKHGIRIMFELLTGSPLSARKRLQFNMPLEPNPKV
ELFHNFTPT
VLPIFWVEEAVDLNSTFTKPLKTLFLTKKLVNIVKYLVLLMSIGGFCAAVYLYFKSDDSMN

VTSVQKVQP
DQNGHRNIISTVFNGNHTAGQDNEAYEDKY
>DponSNMP2
MFRNCCSPRLVFLYNLLAVLLLIASLVLAFWGLPQIISKQIHKQTELTENTDQWDRFKELPF
PMEFNIRFFLVTNPADVL
NGSMPILKESEPYKYKSTIKRTDIRFDDIEEDSVTYRRSFSFEFDGSGTTREDDSITVINPLL
MASFQLTNDIQRLAMAG
CRKYILEPAGLDQVFLTTTVRKLLFDGIYFGFQNATGKGVACEMVRKELGKIVANVRVVE
HLNDTDCYRLAIFNYKTDNF
LKNSPDGIYTINRGRNNATALGSIMRWNGATTSTTYGTSTSINNLTCHSIKGTDSTIYSPELK
AGENLMIFNTDLCRTIQ
LVQVSSNEVFNGINAFRYSTGYTLFRPETILKENDCYCSHGTKGADGKPSCFLDGLLDFRP
CLGAPVLISQPHFLHADVK
YIRAVSGLSPDEDKHDIYLLLEPNTGTPLEGRKRVQMNSVLRRQPLLSMITPPNMYEAVVP
LLWLDEGFTLPQKYLDDLN
AKYFKTVRIATGFKFGFIAVALALLVGCLFVACRKMYFRNAK
>AglaSNMP1
MFLKIPFGLEFRVYIFNVTNPMEVQRGQAPSLKEVGPFCYEEWKEKVDVQDMEGDDTILY
NAKDTFIQVM
WPGCLSGTEVVTIPHPMILGMVNTVVIQKPGALTLVNKAIKSIYSNPASIFLTAKANDILLD
GVIINCDV
KDFAGKAICSQLKEAPTLRHASENELAFALLAPKNATPGKRIKAARGVNNFKDVGRILEY
DGVDKIDVWP
TDECNAIRGT
>AglaSNMP2
MVRKTFCSIKVLLITTLALLVVLVGVLVLSFVGVPKIIDSQVKSSVQLEKGTEQWDRFVEL
PFPLHFKVWLFNVTNPEEI
QKSAKPILNEVGPYYYDETVIKDIMLYNPSDDTVTYKRKLSFAFNKEESGTLTEDDELVLL
NPVMLSMSQLTTVLERMAL
GGCLDRIFPDRYNNLFIKVKVGMMLFEGYEFAVKHDDLGVACGIIRNKVIQKTANIRNIEK
VYKDGKLYSLRFSFFHYKE
SPDGVYSVSRGVDDISMLGHIRRWNEAIQLPFWGRSISFNNDTCNTVRGTDSTIYPPSITEN
SELDIFNTDICRVIKITY
AGQDTYKGVDGFIFTINENTLRNETEFAENDCFCVQQTLNELGESSCYLDGLIDLKSCVGA
PILLSFPHFLYADDHYREA
VEGVDEPDESIHKLYLLVEPNTGTPLEGKKRAQLNAVLRPANFLSFSEDLPQVVIPMFWVD
EGVSLPQKYVDELNDMYFN
RLQIAAGVKYGVIAVTLAAVVTSTGFLVRKKFFTG
>CbowSNMP1a
MRFPVKLAIGSISAFIFIILVGFVLFPRMITSKVKGMVNLAPGNEIRDMFIKVPFALSFKIYLF
NVTNPM
EIQSGEKPIVKEVGPFCYEEWKEKMNIEDKEEDDTISYNQKDTYLKKWWPGCRNGQEEV
TIPHPLILGIV
NTVARQKPGALSLINKAIKSIYSDPSSIFLTAKVDDILFDGVVINCNVSDFAGKALCGQLRT

AEALTKVG
EVEKFSLFSSKNATLQKRIKAYRGKKNHRDVGRIVEYNSSKMMDVWPTEECNSIEGTDGT
IFPPLTKPGE
GLFMFSPDLCRSLIAFFVRKSTYDGIPCGEFTADLGDMSKNEKEKCYCSTPETCLKKGMM
DLYKCSGIPI
YASFPHFYNSDTSYLKGVGGLSPNKTKHEIKILFESITGSPLYARKRLQFSMPLESTQKVEL
FKNFTGTV
LPIFWIEEGVGLNRTYTGQLKSLFTLTKVVKVSKWLILIGSLGGLAAAGYLFFKVDGRADI
TPVHEIRRH
ESKSGSTVNGAGGHVLSGNGLEKY
>CbowSNMP1b
MRLPLKLGVAGFLLILLSVIVGFIALNPIVRFGIRQQTALKRKSEIRNIYLKLPFPLDFRVYFF
NISNPM
EVQKGATPILTEIGPYCYDEFKEKIDVLDNDAEDSLTYYPYDIYKFNAEKSGKLSDTDYVTI
LHPALVGM
VNQATRDSPALLSIVNKAIGPIFRDPESIYLTAKVKDILFDGVELNCKVTEFAAKAVCTQIKS
QIPGIKS
DPEKSIFLFSLLGVKNATVGKSIKVSRGISNSRDLGKVLEFDGKKVLKLWYEEQCNHFKGT
DGWIIPPLL
KPEEGLWSFSADLCRNVVAEYVEDSVTKGVKTRRYEATLADMQNNEEDKCYCPTPKTCL
RKGVFDLSKCM
GVPILATLPHFLEADEIYLQQVKGLNPILDKHIIRIQLEPMTGTPIEARKRLQFNLPVSASEKI
TLMRNV
STSLHPIFWIEEGVELDGALLEKVTEVFTFLGVFQVFRWLGLLIGFVSIAYAVYHHMKHSR
SVHITPISG
SSSSDHVDINRSTNELVGKMKEVFQSDKGHTNPVMTGHEFDRYS
>CbowSNMP2
MKMFGASRFCNVKILFVTTVVATVVLIGVLLLSFVGMPLIVNDQLAKKLRLENNTEQWD
RFVELPVPLNLNVFVFNVTNS
DEVTNNKATPILQEIGPYCYEERITRKILSANSTEDSITYEQSFNITFDEKRSGQWKESDKIV
MVNPLFLILSQITNVIE
RFVVMGCIDKLFPPKYSTMFFEVDIKTIMLEGIEFGVASDDIGPACNIVRNKLLEKTLPMKN
VERIPSPTDPSVINSLKF
AFLQYKIRGPDGQYTTNRGIDDITQLGHIMRWDHSAEIDVWGRGESTNNATCKEVKGSDS
TIYPPHVTKSTKLDIFSTDI
CRTVQIRYKGTGTYQGDSGYYFGIDENTFRPATPSPENDCYCIQQTMAPDGEPSCFLDGVV
DVYPCFGAPILLSFPHFLY
ADESYLDGVIGIDPPNSSIHEIFLLIEPNTGTPLQGMKRIQLNVVLRPVEFVEYTANLPSTVL
PLIWIEEGVNLSQDLLD
KLDKMYFNVIKAADAAKYAAIGVLTAFVLISGGFFVRKRYFK
>CbowSNMP3
MKFYSVLFVVKDRANMLNKFNITVSGKIIVILGVFGLFCIFAGFYVGFKAVPDVITDKIWD
MKVLKENTEQWGMFMKTPF
PFTFKVYLFDVQNPQEILQGAKPVLRETGPFVYKVYKWKSEVEWDTPDDISYFSYMRFEF

DRKASGIFSEDMKVTLFNTA
YYGMLQKIDETQPEVLSTVEGVLPSIFGENHGLFIKVKVKDYLFDGLKICENEGKDGGFV
AGMVCKQMIARLPESKNLRL
EDNSILFSNMHYKNNTHQGRFTVKSGGQNRTETATLTLFNGKSYISSWTGEKSMCNKIRG
ATTVFPVNIEKNMTFEAYSE
DICRTIPLEYSAEETVKDIVGYKFSAMNDSFSSTKKENFCYCTNTTRTLDGEYGCLKDGVT
DLKTCIGSSILVSFPHLLY
GDEEYLDSVIGLNPEKSKHETTVILEPISGFPLSVTQRIQFNTFLRPIDNVISLENVSKSLFPL
LWVEESLILDDQYTDM
LKNELFRTIKIVDIVKWVTIGSGAACVLIALILRMSSKTT
>PstrSNMP1
MQLSVKLILGGAAMLGATVLVGFLGFQPLVNVVVKDQTSLRKRNQMRKLYLNIPFPLDTR
IYFFNVTNPM
EVQNGSKPILQEVGPYCYDEHKNKINVADNDAEDSLRYDAFDVYRFNKNRSGNLSDEDY
VTIIHPLLVGM
VNRVASDSPALLSILNQAFETIFKNPQSIYLTDKVKNILFDGMELNCEGADFAAKAVCTQLK
SLVPGIKE
KPTNKNVLLYSLIGPRNATVASTIKVLRGTRNYKDIGRVLEVDGKKQITLWGSDFCNRFRG
TDGWIIPPL
LDPADGIQSYTPHLCRNIDLKFVKDDVIKKIQVRRYETTLGDQTNNTLDKCYCSPKRCLK
KGVFDLTKCV
GAPIMATLPHFLETDQSYLSQVDGLHPNWEDHILNINIEPMTSAPLDIKIRIQMNLEIGPQP
KISVMKNL
PVALHPIFWLEDGLELEGELYEKIANIFVLLKMAQILRWTFIVVSIAIIAYGYYLIMKNRKSV
KITPVHS
ASSYDSYDNEAFNNRSTNAIISQLKTEMNYPKNYRNVSNNNTNNNNVGGGHEFDRYS
>PstrSNMP2
MVNKNGLKLSNKVLIFVSVLGALMVIGGAYLGFKVVPDIIVNKIWETKILKENTEQWEAF
MKTPFPYSFKVFVFDVQNPD
DVLQGAKPRVKEVGPFVYKVAKWKDDVQWTSPDEISYHSYTKFEFDEASSGEYTENTEV
TILNSPLYGILLKVEATKPEV
FGLVEQAVPVAFAGHSQLFIKVKVGDLLFKGIKFCENAGENGGFATSIFCRNVMQKANESQ
SLRLENDAILFSNLHYKNN
THLGRFTVKSGGKERKESAALTLYNGKPFLSTWPGENSSCNRIRGFTTVFPANIKTDMVFE
SFSEDICRHVALEYDSKDA
VKEIAGYKFVAKNDTFSSKTNKENSCFCSNRTKTFTTAEGCPEDGIIDLTPCKGGPVMVSFP
HLLYADEGYARSVEGLRP
VKSRHEPFVILEPLSGLPLYGSQRIQFNMFLRPIEGMENPWNVSRSLLPLIWVEESFVIPDQF
IGKLNDNLFSKLNMINI
VKWIVIVSGGAGLLLAGLTLVYRQAP
>TmolSNMP1
MRLPVKIAIGCAIGACVIVIFGFIAFPKMIKGKVKKMVNLDKDSEIRKMFVKVPFALDFKV
YLFNVTNPM
DIQNGALPVVQEVGPFCFEEWKEKIDLADGDEEDVMFYNPKDTFYKANWPGCLDGSQM

LTIPHPLILGMV
NTVARTKPGMLTLVSKAINSIYKTPDSIFVTAKAMDILFDGVVINCGVKDFAGKAVCTQLK
ESPDLRHVT
DDDLAFSFMAPKNGTAGKRFKVLRGVKTSHDVGRILEYDEKKEMEVWPTKECNQYKGT
DGTVFASFLAKE
EGLASFAPDLCRSLVAVYSGDTKYDGIPVRIYTATLGDMSKNADEKCYCPTPETCLKKGL
MDLFKCAGVP
IYVSLPHFYESDESYVHGVKGLNPNKKDHGIQILFESITGGPVAAAKRLQFSMPLEPNEKV
PLFNKLPST
VLPLFWVEEGVALNNTFTGPIKDLFKIKKIVKITTWVVLVGCLGGLGAAAYLFFAKKGEA
NITPVHKVKP
ANETNGISTVGTDGFGGQVNHAMSDNEIEKY
>TmolSNMP2
MASCSCCTLKVSLILFVVSVAFFIVAFAIAFKVFDDVIYSEVNKAVRLEEDTEQYDRFIELPF
PVDFKVYLFNVENPDEI
ISGTAKPNLTEVGPFVYKQHRKKTVLNTNSEEDTISYTQLETFEFDTDASAPLTEDEVITVL
NPALMSMYQVAEEFFMAG
AVDQCTKSTFPAGYETLFITVPVKDLLFKGFNFCKNDGDDLCALVNDIVCKIGSTKRNIDIL
EDLSLQFSFLNYKQREPD
GKYTVKRGMDDITTLGHIDAWNNMKFTQYWGESTTCSEVKGTDSTLYPPRVTEDNAFYI
FATDICRFVEITYKGEETYKD
IDGYLFGTSKETLRSTQENPEDDCYCSKLSKDETGTKSCFLDGIIDMQTCFGVPVLFSFPHF
LWADEKYANGVEGMAPNE
DIHKTYLVVEPNTGTPLKGVKRIQLNVAMRPVTGVKSMLQATRAIMPLLWIEEGVALTDE
YVDKLRSSYFDKVNLVDGFK
WALVVISSLLLVVSGGFVIWKKFFQQS

>DmelSNMP1
MQVPRVKLLMGSGAMFVFAIIYGWVIFPKILKFMISKQVTLKPGSDVRELWSNTPFPLHFY
IYVFNVTNP
DEVSEGAKPRLQEVGPFVFDEWKDKYDLEDDVVEDTVSFTMRNTFIFNPKESLPLTGEEEI
ILPHPIMLP
GGISVQREKAAMMELVSKGLSIVFPDAKAFLKAKFMDLFFRGINVDCSSEEFSAKALCTV
FYTGEIKQAK
QVNQTHFLFSFMGQANHSDSGRFTVCRGVKNNKKLGKVVKFADEPEQDIWPDGECNTF
VGTDSTVFAPGL
KKEDGLWAFTPDLCRSLGAYYQHKSSYHGMPSMRYTLDLGDIRADEKLHCFCEDPEDLD
TCPPKGTMNLA
ACVGGPLMASMPHFYLGDPKLVADVDGLNPNEKDHAVYIDFELMSGTPFQAAKRLQFNL
DMEPVEGIEPM
KNLPKLILPMFWVEEGVQLNKTYTNLVKYTLFLGLKINSVLRWSLITFSLVGLMFSAYLFY
HKSDSLDIN
SILKDNNKVDDVASTKEPLPSANPKQSSTVHPVQLPNTLIPGTNPATNPATHHKMEHRERY
>DmelSNMP2

MIHWSLIVSALGVCVAVLGGYCGWILFPNMVHKKVEQSVVIQDGSEQFKRFVNLPQPLNF
KVYIFNVTNS
DRIQQGAIPIVEEIGPYVYKQFRQKKVKHFSRDGSKISYVQNVHFDFDAAASAPYTQDDRI
VALNMHMNA
FLQVFEREITDIFQGFANRLNSRLNQTPGVRVLKRLMERIRGKRKSVLQISENDPGLALLLV
HLNANLKA
VFNDPRSMSVSTSVREYLFDGVRFCINPQGIAKAICNQIKESGSKTIREKSDGSLAFSFFGH
KNGSGHEV
YEVHTGKGDPMRVLEIQKLDDSHNLQVWLNASSEGETSVCNQINGTDASAYPPFRQRGD
SMYIFSADICR
SVQLFYQTDIQYQGIPGYRYSIGENFINDIGPEHDNECFCVDKLANVIKRKNGCLYAGALD
LTTCLDAPV
ILTLPHMLGASNEYRKMIRGLKPDAKKHQTFVDVQSLTGTPLQGGKRVQFNMFLKSINRI
GITENLPTVL
MPAIWVEEGIQLNGEMVAFFKKKLISTLKTLNIVHWATLCGGIGVAVACLIYYIYQRGRVV
EPPVK

>AchiSNMP3
MSPVKRSSILSFIFPKAYLVRYVVDTIRRFYNLMVQSATKIKMKLPVKLGIGSSVLFVFIVLV
GFVVFPK
MITSKIKSMVNLKPGTEIRDMFLKIPFGLEFRVYIFNVTNPMEVQRGQAPSLKEVGPFCYE
EWKEKVDVQ
DMEGDDTILYNAKDTFIQVMWPGCLSGTEVVTIPHPMILGMVNTVVIQKPGALTLVNKAI
KSIYSNPASI
FLTAKANDILFDGVIINCDVKDFAGKAICSQLKEAPTLRHVSENELAFALLAPKNATPGKRI
KAARGVNN
FKDVGRILEYDGVDKIDVWPTDECNAIRGTDGTIFPPLLSEEEGLVSFAPDLCRSLVAEFQQ
KTKYDGIP
VRKYSATLGDMSKNEDEKCYCPTPETCLKKGIMDLYKCIGVPIYVSLPHFYETHESYLKG
VKGLRPDKSK
HEIIILFEGMTGGPVYAKKRLQFNMPLQANPKVDIFNNFTESVLPIFWVEEGVELNNTFTK
PLKDLFKIQ
KIVKITTWTVLLGSLLGLSAAGYLFFKESGTADITPVHKVHPSDSRKTISTVSGNNLEGIDN
HAMTKTDT
D

>AchiSNMP2
MRMVDNTVLFSNLYYKNVSHQGRFTINSGKDNSEETGVLKQFNGKTYISTWLGEKSICN
KIRGVTTVFPA
QVKKSMVFESFAEDICRAMSLRFEKEKKVKGVLGYKFVAANDSFSVANDDNSCYCVNKS
KTLEGEFGCLR
DGLGDLTTCTGAPVLVSFPHLLHADPEYISSVVGLQPNASKHETFLTLEPISGFPLELAQRV
QFNMFIRP
FEAISSLENVTKALIPLIWVEESTVLGDKYVDKLKNELFKNLMILDVIKWGFIGIGVALVMS

AFFLFIYV
KSP

>AchiSNMP1
MILSHKLSMAGGSLIVFSVVFGFLAFEQILKFGIRDQTALRKRNAIRSLYLKLPFPLDFHVY
FFNVSNPM
EVQTGSIPILEEIGPYCYDEYVEKVDVVDNDGDDSLTYSPYSVYKFNQEKSGILRDDDYVT
VIHPLIIGM
VNLVSRDMPALLPIVNKAIGLIFPDLESIYLTAKVKDILFDGMAINCKVTEFPAKAVCTQIKS
KIPGIKT
TGNEVYLFSLLGPRNATKGKRVKVLRGISKSKDLGKILEVDGKREIHLWGTPECNTLRGT
DGWIFPPLLE
PEEEISTYVSEMCKSVSAAPAGTTVLKGINVLHYDMDLGDMDHNEDEKCYCDTPKTCLK
KGVFDLSKCMG
VPIYVTLPHFLKTDEIYFKQVKGMHPLPEKHKLEVFFEPMTAAPIAAYKRIQFNLPIAPNNK
ITLMKNLP
EALHPLLWVQESVDLEGPLLKKVKSIFVVVKVIKVARWFGIAVGIAIIGFAVFHYFNNKKE
VKITPVHHP
QSVSNSLTESNSNRASIKESNIDKRKVRSILNGHEFDRY

>AmelSNMP2
MWSYQVCAIICVIFGIYACITNLFSDGLFSIKNAILKNLPLIKGKDMYDEWILPVNLIFKCYF
FNVTNPD
EVMEGNNPNLVEYGPFTYREVFEKQIVDVDEELDEIIYDVKSTFTFDKYASLNISKRDTVTI
LNPAYIGT
ISMASIIGLTTLPPSYIEKFGNNIPKLFPNRSSIFLKANPKEILFDGVKLTCNERKFPELSTICK
TLKAL
RSPVLKEGEKEGVYYLSIFQRVNGTIRGRFSVNRGVNNISELGNIGSYNGRRVQTIWRTEK
CNTVRGSDT
ITWAPLINPMPSVLSFIPDLCRSIEADYDKEVSIYGLIGSRFVMRERTWFLNQSQCYCLERN
KVPNCLPQ
GLIDVSDCLVMLRYVMLQKVPIIMSEPHFLHGDPQLLMYALGLNPSEDLHETFIVIEPYTG
TPLSGQKKI
QLNLKLERQPVDLLSNISEGYFPLLWCANVRIFSKIIKLQY

>AmelSNMP1
MKPKKLGIIGGSLLAFGILICAIAFPPFLRSQVKKQIALKDGSEMRELWSNFPVPLDFKIYLF
NVTNPME
ITAGEKPILEEVGPFFYDEYKQKVDLVDREEDDSLEYNLKATWFFNPSRSEGLTGEEELIVP
HVLILSMI
KLTLEQQPAAMGILNKAVDNIFKKPESVFVRAKAREILFDGLPVDCTGKDFASSAICSVLK
EKDDALIAD
GPGRYLFSLFGPKNGTVLPERIRVLRGIKNYKDVGKVTEVNGKTKLDIWGEGDCNEFNGT
DSTIFAPLLT

EQDDIVSFAPDICRSMGARFDSYTKVKGINTYHYKADLGDMSSHPEEKCFCPSPDSCLTKN
LMDLTKCVG
APLIASLPHLLGAEEKYLKMVDGLHPNEEEHGIAMDFEPMTATPLSAHKRLQFNLYLHKV
AKFKLMKNFP
ECLFPIFWVEEGILLGDEFVKKLKTVFKTISIVGFMKWFTIVSGTCVSGAAAALFFKNKDK
NKLDITKVT
PQKGEEKKWPNQMTISTIQSAAVPPNLDAD

>BmorSNMP1
MQLAKPLKYAAISGIVAFVGLMFGWVIFPAILKSQLKKEMALSKKTDVRKMWEKIPFALD
FKIYLFNYTN
AEDVQKGAVPIVKEVGPFYFEEWKEKVEVEENEGNDTINYKKIDVFLFKPELSGPGLTGEE
VIVMPNIFM
MAMALTVYREKPAMLNVAAKAINGIFDSPSDVFMRVKALDILFRGIIINCDRTEFAPKAAC
TTIKKEAPN
GIVFEPNNQLRFSLFGVRNNSVDPHVVTVKRGVQNVMDVGRVVAIDGKTKMNVWRDSC
NEYQGTDGTVFP
PFLTHKDRLQSFSGDLCRSFKPWFQKKTSYNGIKTNRYVANIGDFANDPELQCYCDSPDKC
PPKGLMDLY
KCIKAPMFVSMPHYLEGDPELLKNVKGLNPNAKEHGIEIDFEPISGTPMVAKQRIQFNIQLL
KSEKMDLL
KDLPGTIVPLFWIEEGLSLNKTFVKMLKSQLFIPKRVVSVVCWCMISFGSLGVIAAVIFHFK
GDIMHLAV
AGDNSVSKIKPENDENKEVGVMGQNQEPAKVM

>BmorSNMP2
MLAKYTKTIFSVSVAFLVVSIVLATWGFPKIIRKQIQKNVQISNTSKMYDKWVKLPMPLDF
KIYVFNVTN
RDAINQGEKPNLKEIGPYVYKQYREKIILGYGDNDTIKYNLKKTFVFDPVASGDLREDDEL
TVINFSYMA
AIISVQEMMPAAVGMINRALEQFFTNLTDPFQTVKVKDLFFDGLFLNCEGDNTALGLICGK
IRAEKPPTM
RISKSANGFYFSMFSHMNRTVSGPYEMVRGTENLSDLGHVISYQGKRIMSAWDDQYCGQ
LNGTDSTIFPP
LEDGNIPEKLYTFEPDICRSLFASLVGKDTLFNISTYYYEISDMTLGSKSANPDNKCFCKRN
WSVKHDGC
LLMGVLNLAPCQGAPAIASLPHFYLGSDELADFFGDGIKPDKEKHNTYVHLDPITGVVIKG
VKRLQFNIE
LRNVPSVPQLKEVPSGLFPLLWIEEGAEIPEWLRKEIMDSHTMLWYVDAARWLVLAVAVV
AVLVSATLVA
RSAALIPWPRNSNSISFILGNSVNTSKVHS

>ItypSNMP1
MPHPKNIAWAGGALAFGGVLFKVWLFDVLVRFGVKDQTALRYRNEVRGIYLKIPFPLNFK

IYFFNVTNPE
EIQNGAKPVLNEVGPYWYDEYKERVDVIDNDTEDSLTYTPYDLFKFNPNMSTPLSDNDY
VTIIHPVIVGM
VNLLLRDSPMLLKVVSKAIPFIFNDPKTIFLTGRVKDILFDGVVLNCTSKEFASTAVCGQMK
GQVPGLKP
TPGQPNLLLFSLLGPRNATRTGSLKVLRGIKHFQDLGRLLEVNGRKSIGIWAGDQCNRYDG
TDSWIFPPL
IQPESGLKSFSTDLCRNIKMKLVNETVVKKIPVGVFEPTWGVKVVTRRKSATVPTLPVXX
XXXVFDLTKC
MGVPLYATLPHFLDTDPNYLKLVDGLKPDHEKHRIVVFFETMTGTPLKAAKRMQFNLELQ
QTNKLELFSK
LPAALFPIFWLEEGMELEGYFLKKIQTVFMLLLFADVTIYVTIATGLSVCGAGFYQYWKNT
KSLSITPLT
KNNNGLSEPKLN

>ItypSNMP2
MRFLQRVKFNLKTVFLCGISGVSLLVVALFLGFIIFPKVVNDQLLETKILREDTEQWAIFKKI
PFAFTFN
VYLFTVENPEEILKGAKPVVKEKGPYVYKLYKWKEDIIWNYTTDEISYYEYEKYVFDQEA
SGSLTEHDKV
TLLNLPYLTFLYTAEANEATSGFLPLIDEALEFIFSGHNSPFLVNVTVRDYLFEGVEICKNGC
EDDGFVA
KMACGKIKDNLKVAKQMRLHHKDILFATFHYRNNTHQKYLTVNSGRQNHLEIGAITQLD
NSSTMNVWNQF
GCNQVSGLTGIFPINLGFKTTFQSFSAEICRPVKLHFSTIKPFGSIKGYKYVALNTTFNTSMV
ENQCYCT
GKIPNLDGNLGCLYDGVLDLSTCLGAPIVVSFPHFLYADWRYVNNVKGLSPNETNHQIFV
NLEPISGTPL
EAATRIQFNLFLRPVRNITSLDSVADALVPLFWIEELTYLPQKYQDVITGKLYRSIFILNAIKY
VLLAIA
LVIITVCILIFLYTD

>TcasSNMP2
MGCSCCTIKVLLVCVVISVALLIVSLALAFKVFPDLLESEVNKAVRLEDGTKQYDRFVELP
FPVDFKVYL
FNVSNPQQVLDGTEKPKLEEIGPFVYKQYRKKTILGKNEEEDTISYTQKETFEFDAEASKP
LTEESVVTV
LNPALMSIYQLAEDLHLAGAADTCIKQTFENNQGKVFIEANVRKLLFDGFSFCKNTSPGIC
GLVNDLICA
IAATKRNSDLVLPDYSLIFSYLNYKRKPDDGKYTVKRGLTNIEKLGHIVAWNDSLYTKFWG
EGTTCSEVK
GTDSTLYPPRVTTDSAFYIYSTDICRFVKINYKGEESYKGIDGYLFETSEDTLRSSAPEEDCY
CSKLSRD
MEGKKSCFLDGVIDMQTCFGVPVLFSFPHFLWADNKYLSAVEGLNPVEEKHKTYLVVEPN

TGTPLKGMKR
IQLNGVIRPIVGIKSMLQTKRALLPLLWIEEGVSLPQKYVDELKSSYFDKVQIVDGVRYALI
VISAILVG
AFGIIILRKRSHAKHHV

>TcasSNMP1
MIKGKVKSMINLNKGSEIRQMFVKVPFALDFKIYMFNVTNPMDVQKGALPVLKEVGPFC
FEEWKEKVDLD
DNDDEDVMFYNPKDTFYKANGPGCLDGSQMITMAHPLILGMVNTVVRTKPGAISLISKAI
NSIYGNPDSI
FMTASAMDILFDGVVIKCGVKDFAGKAVCSQLKEAPDLRHVDENDLAFSFIGPKNATPGK
RFKVLRGVKE
SHDVGRILEYDNKKEMEVWPTKECNQYKGTDGTVFPPYLTKEEGLASYAPDLCRSLVAV
YSGDTKYDGIP
VRIYTATLGDMSKNADEKCYCPTPDTCLKKGMMDLFKCAGVPVYVSLPHFYESDESYVK
GVVGLNPNKKD
HGIQILFESTTGGPVKAAKRLQFNMPLEPNPKLPIFANLPNTVLPLFWVEEGVALNNTFTKP
LKDLFKIM
KIVKIAKWLIMLGCLGGLGAAGYLYFSKKGEANITPVHKVKPAENGVSTLGGEVNHAMS
DNEIEKY

>AchiOR1
MEDLYNVQLLIQYGASVFILCSLSYIIPLVDNVGELICSFIFLSATLGEIFVFTYCSQTLSLEL
QNVKDA
VYNLDWPCYPPRLRRNLVFLISKLQKPCLLTA

>AchiOR2
MTPLFSGQFVILIVYFSAIMIELAMYCWVGNEIILKSLQIGDACYMSKWYEFSPRTNKILFLI
MERSKRP
LTISAYKFSVLSMSAYLKIIQCSYSYFTVLRRVYMKD

>AchiOR9
MIETDIGRKEVRIVESMLEQDMQQNYDFSSYFRPSIIMLKLFGFWRPDRNLKFKGIYNCYT
ALCSSIWVA
FLLSQIIYIINNRNDVQEITAALSVTVTFTVDLIVMMFTYKNMNCLKVLIKEMNRPLFQVK
CQKHYHIAK
NTERMYKLMFKSCLYLAILTDALVTVVPLMGKEKKSSIKGWFPYDYTTPLYFILTYIFQNL
VFIWNTFIS
FNIGMIILALLIQVGLQCDLLCCTLDSLDDFYTEGNVLYEISLEDKLKLTTDRERFSKEMTK
NLVICIEH
HRQILRVVKDVERISGTGLFILFVGGGLILCSSLFPLSVVKIGSIEFIMLLFYLICMLTVQFLY
CWLGNE
IIFKSSLILQSAFNTPWIGCNVKFQKILLVFMMKTSKPISILTGGLFRMSVPVFVSILRTTYSY
FTLLKN

IQ
>AchiOR10
MRNRLRFCIGYHVHILKLGYRISSLVKHTVGHVSLLSALVCGCMANQMLTTKPIGSFILLIA
WLMGVFSY
CHSGQRIKEKTMSIGDALYESKWYNADNETMKDVQFILMRCRKPICYEAIPLGITDYPFYF
MMIKTGYSY
FTLLNQST

>AchiOR11
MELKTPYFKKHLKWLLVLGVDIIPVKNVWYKYFYKLWSVLIIGFVVLYTLLEVIDILNTSD
FNSMTFGLC
YSATHLLGLAKIIILIVKKKKVREMLNELESGDFLPNMERGGEEEIRLINIAVTRCARHAEIF
NLIVYSI
VSIRCLYALFDTGYNDELFDEQLNTTTPIHTRILPYRIWLPIETTKSPIFEIVFFFQAFTLTLYG
YYIGM
MDSMVYGMMFHMNTQYLILKRVLERYVSIATNMVSKNLLDKGVKDIRNGVISLPVGYER
IDLLSEPVQEK
VREIVHNCAKHHVHILEFCEKVEKEFSYLMLSQFLFSLYTLCFQLYQLSLMANVFSFDFIS
MCCYLTLLM
YQLFCYCFYGNEIMVQSEKFSEALYNSDWLVLDNSTKRSLLLMMMRAQRPIRFTAGKFAL
LSLQTFMAIV
RGSASYFMVLRQMSG
>AchiOR15
MDSRFLLYIKHLMIIGGTLPVDLINSRKRIYIFYRYVTHTFVSLVGIYWLGGNMNVDGFDR
TLFNYIYDR
IFVMISYVNLAITTSGNFQKLIYEMIAYEAEVLDVGSEKHLKIHEQVVKKSRTLQVYYISIL
ASCSIAFV
VPAFVEYALVQSSEESTNSTINTHNYELWIPFEESKEYLVWLLVQSCYTLIVTCIYCSYQIILI
NLLLVV
ILRLKILRARIENMKDIERLDAKRLIRIYAKEHIDLIRNCKHVDDTVKYVMLMEFLFASLRL
ALSIFLLV
TANTPNSKIYFGTVIVNVLINILILCWNADQIREESIGIADSIYQLPWFEYDKSDVMSLHIM
MIRSQTPL
TLTTGPFGTVTLDLAGKIVKATYTYATFMHQMYEN

>AchiOR13
MYNLMILAQTLTSLFNVASCLFSASREPVGSAVFFATLVYFTSILIELGVVCWFGGEITTASE
DIMFALY
EVDWFSASQRFKHSLILTMCRMQRPIYLSIGKFFPLTLSAMVSVCKASFSYYTVFRRTDE
>AchiOR17
MKMKRHIDLLKYIKEERTCMVFGGFYSTKKYKTLHSLSAILIMAVVSLYNLLGLLHGFQHI
SNVAVFSQS
IAYLLTGISFSCKMINLIVHKNNLLLLDEILQNPVSTELETTEEEVVLRNKLKFGQTLKKIYK
LYTSATV

TVQVLYPMINNPGHKNFPLLFWFPFNPEDHYYKVYFAEILMIFCICTFNVTVDLLNVLFMD
LCAAQFELL
KYRLKHFGREFHGGEAVNDRTLYEKLNKIIVHQNLVYRFSKLTEDTFSAGVFCHLACTVIV
LCCAIFKAV
ITPIKSMQFLMMATYSFCMTLEVSLYCCYGQKVLDSSSTVTEACFMANWYNCNVKVQED
LVIIMNRANKS
VTMKAGGMFPLTLETLMRIWSSAYSFLTLLMQIYNENY
>AchiOR18
MCISMYNITMNQGSISDKVRMFVIIFIMVAEFFMVYGLPAQLLMDESAAIADTVYSECKW
YSSKLQHIWN
DFFIVIIRSQKSVCIRAGNYHIISNATVLLMVKS
>AchiOR19
MKKFKENDENITVEAVIRYCVLEHLKIIQITNTLNNCLRNVTLLEFIVLSAQIALIAFEGFTS
QSANTVV
VCIVHVLMLLVHMLLFYWHADEIRHESMAISEALYETDWYEYSRSTSSTIHIMMMRSQRP
LSLSVGPFGE
MSLTMALKILKGVYTYMTFLQHSYGQTSSLGTN

>AchiOR23
MEIENDHDVEIYIRKLKSILKYQQFLMRYVDYLNELLSVPIALLMVTSVSLKCISMYNMTI
NQGSISDKG
RTVVTILSVIAEFFLAYGLPAQILMDESTATAHIIYSECKWYLPKLRCLRSYFLIMMTRSQRG
VCIRAGN
YHIINNGTVLLMVKTAYSFYAFLQNVT

>AchiOR25
MEKFSKAMVKNLVVCVQHHREIIKLAKDIQRIKEIGIFVLFASGALVLCTCLFQLSMVQFG
SVESMMLLF
FSICMLTEQFLYCWFGSDVIYKGSLILQAAYNTPWTDCNSKFRKILLQLTTQACCPLNILAG
GLFIMSVP
VFISVLQTSYSYLTLLHSIQ

>AchiOR29
MKMNKTFDYSQLMAHIFKIYKILGFWRPDPDMKHKNLYHCYTAFWLSLSVTFMSSQVIY
MYNNRKSLKEV
LAALYITLTFVSILARQLTTYKAMNELKEIIKQLNRPLFQVKCQKHYEIAEETNRNQRLLYN
ICLFLGVS
TDIFAALFPLFSKEKVILAKAWFPYDWTKPFNYFMTYIFQNTVLIWHTFVCYSIDIFTFILLV
QIGIQCD
ILCYTLNHLDNFYFKDGMLHEITPRDKLEFRKDMEKFSQAMAKNLVVCVRHHREIIKFTK
DIQRIK
>AchiOR30
MDKLRYFLHLMGWIGMLFITCFYGQMILDESVTIADAAYQSEWYNGPEHLKKMICLIIVR
SQRPLLLRVA

SIGVVSLETFVSVIKTAYSYFALLLTIAK

>AchiOR31

MYLSELYIPLTMCRCMGVYPEEKYRTGQIFIFLIIFISQWIIIYLAILHLIYKDHITISDITNALE
TLFL

IFHAMIKLSMFFVKESEFHDLLERINYFWKVNDLEDEVERKKHQKYLKLIKIRSSMYNFW
ASATSVAFML

KPFLLKGDNSIFTTVSPAWIPSGVMTFYEEILFAFGVYGPIVGMDLFTLALLLLTRMQFNML
NQEIQCVF

QNMTNSEENVEETNEKIKKIVDHHNFLLDYVNRINNAISEGMFLYIVTILLSMCVEMYIAS
VQKSVMIAI

QAMMYASNGLLQYCICYCLPAQSVTDEAELTANYVYFNNWNEHPLPSIKVAQIMIIARAQ
QKTLILAGGF

IKLDLETYLKTLKTMISYAMFIRTMGIGQD

>AchiOR32

MATTFISCADIFAFSLILFGVGQIKILKLILSNFQEYTMKIKNLLHCSQEEASYITLRECILKH
QEIIEY

IKEYNLVMKNIMVLDFLMSSMQLASIVLTLLVTKVTLINTVYSGQFAVCMFLRLLVYYWY
ANEIMVHGSE

IGLALCNSNWYEESERVQKMMVIMLMRCNRELCLEIGPFAAMTLRTFLGILKATYSYITVI
YR

>AchiOR33

MPSNDLVQRSFKYHLIIMKIFGLYPFDSWPQYFTPYALFLYIIFTIATPILAVIHLIVDEKPIVD
VITEN

AFMIVELIALIAKFLPFKIYPERTKKALSALNKEIFNNHLPEQESVLDETVANCRFIFRIFCTS
CAFAVL

SWASLPLMYEERRFPIDVWLPFEPFENTAVYLSVYLFVCLSGVHAGFDNATVDSIVALLIY
NASSQVIIL

KDTLMHLSKRTEDEISKENRSLSTEEKENLKSDIIYKKICHCVDHYNAIYQFVEDLEDIFSM
VVFSQLIA

SIIIICICCLQLSVAVPFTIPFFGAVSFLTAALLELFLYCYSGTLLFEESGTIVTAIYMSNWYNY
DKKSK

KALLTMMERAKRPMMVTAGKLMNFSLETFSTVIRRSYSLLAVLKNY

>AchiOR34

MPFEIDYFPTRQIFLVVQLFGAHQFYMIAGMCAWFVFETVQHFRIRIRHVNFLFQEAIKEE
DPQRCREKF

NHATRYHASLLGLEDRMNGAFGTFMFTHMATTAPIIGIGVFAIVSGGSASSFLVCLGWSGG
LAMVCFVGQ

WLQDECFAVGIDLYD

>AchiOR37

MLLIGTWNFKSYNNAILVMYKAYSYFIFWHYVVMTQLVMVSIPIQWDCKIRVIELICFYIQ
YTNNIIMMV

LSKYSNKMQKVFDHILDYEDVKMRSTTNVEQKIYFKYAKLNKRVSLFVTAILLGTSVYW
YTTSVRYSLFG

QPTDVCPLTKGTIYQIWLPSIIRKYHWLMIVNDIVFFISVINITLYREIIMFAISIFMLGQIKILQ
QNVR
DLEKTSEEVRNSRNVSYDDALLISVVKCAKEHQQVTKLMEIVQSATSIFILVLYFSNTFEM
AAYLFQLIS
EKSIYNILRPLYVFTLMVSQLYIFYNYTNEILVESTALCDVIYNETNWIDYNQVIKKNLLLM
MRRSQKQL
SFKAAGLGDMSLQTFTNV
>AchiOR38
MEADDIVIMENSLRYLSRNLIFPKKKDVNNPGVIFHLKFIALNLSTVMFLTGNILHLTINIKR
KTYINLD
LDIALAISLFGSYYFNFSYIRQVKNTINIYKQLSDLRSYGIPKDFYATNKKLNNYSKYHYIYI
VSAVLGL
SVAPLLEYKKCQKENVVKNINEICGLIGSIWLPIDLDSIPYKQMYYIFQVYSSFVIYQTSSLI
SFSIMET
AEHLILRLNHVKHGFLDALMEREDRVRREKFSRAVKYHVNIIRISKLLNTSVSTIMFGHVL
LTGAILGCI
EYRLLKSYSLGAICLFVGWLISMSMVCIGGQRLRDQSLSIGDAICKSNWFDVNKELQRDL
VLVILRCQKP
IFIDAGPFGYMTYAMILTVLKTSYSYLTLLSSTS
>AchiOR40
MVTSVGMLCVNMYAMTIYQASVSNEFRFGIIIFSLTAEFFMVYGLPAQLLMEESAATTDLI
YFECKWDLP
NLRPLRTDFLIMMMQGQRGVFIRIANYHILSNRTVLMIVRTAYSFC
>AchiOR41
MYEISKEKSFYSSLTILRYTYAIPSTTEKSGLTWFRFIFIVIRLASLLNFVILPCLHLISTVRAK
TGVDI
SEDLSTMFGAFGFITNAVTFLILYEKWSQFFIDVEDCNRFEIPEGIEERKKKLNLYSLLYALY
AVCGVLI
YGVIASSETSYCKRMNEEHGLSETCGMFTPIVLPFDGSNVYIRSIIFGIQMLLGMFTLPSTAL
ISFLLYE
GVETLILHITQLKKCFIEVFDVASNAERKNRLRFCVYYHINILSLCQRLSSLGKYTSGFLCM
ACALVFGC
IGNLILKSKPIAGISYLLEYVFALFLLCHGGQRLLDETLSVADVIYDTKWYDGDVQIMRDIR
FILARSQI
PVMIGALPLGCMNYALFLMIMKTSYSYLTLLTQNT
>AchiOR43
MVIFVAMNMNMNLLSIVEALLFLATQMAFLCKLFNVLNKKHKLLEIEDILANPAFYGYPK
EKRHFIEDSV
RFTKILGICYRSICAMGFIIYGCFQLMKDEEWPLPLSGWNPIQVNTKFKYGIIATFQWVAFF
MSAYINSG
IDILIYMLISVVTSQFEILKDNLTNIRYETDTAKRDFAKNVVLHYGILKFVRVIEDTFSYATFF
QFFTSV
IVICFAGFEMMIAPLNSIQFVSMCTYFSIMIFQVVIYCWFGHTIIASSDKINDAIYMSNWYEA
DLSLKKS

IMIFMEKCKEPVVLTAGKICPLSLGTLTSIMRSSYSYLAVLQSIYGQE
>AchiOR44
MMSIYFHGSIFSITCILIPLFKETRETPYKTVYPFEYSSSPKFEIMYLMQSFINFYVILGVIIGV
DCLFM
AACYNIIAQFRLLKGVVLKLGTNEVKEINSKLTILSSESDNIGRNVTKEKEFLIRCIKHHQLL
LRTTEDI
ETVYSVIGLFQLGFSIIAICMSSFVVTTGEIEYVQLVNISIFISGHIVQLFCYCSVANEIGFEMD
NLSQH
IFSSYWYQTDFVNIKQDILVIMKKSQEVKRITALKLVPLNYDTFIQVLRISFSFHTLLSNITV
K

>AgerOR42
MNLKIKELNSMEVDGDLGEYIKGLHTIIKYQQFLMRQAEDLNELLRAPVALLMIISVTML
CVVMYILTIH
QGTTLDNARVMVVGCATVAEFFLVYGLPAQLLMDEAAATADMVYDETKWYHINMRPLR
TYFLTMIARSQK
GVYIKAANYHIVNNRTVVLILKTAYTFYTFLQRVAVVNNN
>AgerOR41
MYPDNINFYLKLFNLIGVHPEKGGNVFQIMTYIFGAFVNLSTVFLSILLLVVKEQVTITDIV
DCIESIGL
LLHGYLKMTSLFIKRSNILNLLRTMESHFWNSENMKDSSTKKDLQKIFKFLKNVSDFFTFL
CTIVAILFI
IKGFLGQTIYEIYIPQWFPFYLSVFYQSLACVMTITCPVVGTDMFIFTTFMLTSLQFKMLNE
EIKSIYGG
SEENRIDTHTIKSRLKKCVGHHIFLQNYVKLLNDTFSEVLFIYNGEIVLSLCVEMYIVSTQN
SIQAAVKA
ALYVFTGLFQYTVCYCIAAQAITDQAGEISNSVYFSNWYKYPEKYIRTATILMMLNGQKPII
ITALKFMK
VDLETCLKTIQTMLSYFMFLRTIGIQ
>AgerOR40
MWLIMEIVEHVSVRIDHIKELFVDALNEGDSEEMKRKFGFAVRYHSDLLELGFYINKCFSS
SMLCLILLG
AAILGCASFGYMQAGSSTYLIVCACWFFGLAIICISGQHLTDESLSIGDVIYDTKWYEVGLS
LRKDILFV
MMRCQRPMILRAAGFGVMNYIMIVSVLRTSYSFVSLLGATS
>AgerOR39
MDLCATQFEILKSRLTRISSNFMGDEMVDEKVRLQKLRKYVIYHNYIYSCSELVRETYSIG
EFCQVSCSV
MIVCFGLFKVIITPLKSVQFLMLVTYSTTMIYQVSLYCCYGQRLLNASSTITEACYMSKWN
TCSLEVQRH
LVMIMNRANTPFIMKAGGIFSLTLETLMTIFTSSYSFFAILCQVFNADE
>AgerOR38
MLKNITVGLMLHASDLEKYNSEYFQYAVMKRLKICIRHHCNLLKYGKSIDKYTSTILVPQL
IMSYISLVI

NGYILSADGVASLRSFEILVFTFAIFAEFICLAIQASDLKDQSISVISAVTASDWYLFKAPIKK
ALVLLM
LNAEKGIVITVGGMIEVDNPLIISIIHKVFSAITLLKALILDEGV
>AgerOR37
MLHISSAVFFVSFALLYNLLGIVNALQNIYDLKKFSTTVSYLLKGVSYTCKIGNLLIFKKNL
LLLDDMQQ
DPIIAELQSEEEEIVLKKSLKSSKMLKKFYIAYIVLSVSIQAIYPLVNKSGSNLWYPFNSQDH
FFGVHCF
EMLEVIISSCFNAAANMLTVTLMDMSATQFILLEHRLQRIEPGLSQGDIMEDILEKIQNYVD
HHNYVYSF
TALVEKTFSIGIFCQMGCSVLVVIISVLRAVVMPIKSVEFLIMAAFCLSMICEIALYCCYGQK
ILDASNK
ITESCYMSKWYQCDLEVQKYLSMVMNRGNKSVIMKAIGVFPLTLETLMSICSSAYSFFAIL
VQIHEGSE
>AgerOR35
MLIIHVFLTSAILAIIGYQIILVDSLIDKLRYFLHMMGWVMMLFLTCFYGQFILNESTTIADA
AYQSEWY
NGPLRLRKIIYLIILRSQKPLLLKVASIGVISLETFLWVMKTAYSYFALLLTISK
>AgerOR34
MVAMCFQLTLYCWFGNEVTLKAAELPLCIWQSEWIEADNDFKKSMILTMMRAKKPLYLT
VGNFAPLTLST
FIVIIKGSYSFFTVIKSTGE
>AgerOR32
MFCSYYNCNELILESVNLGNNIFLSKWYEQSLSIKRSLIIVIIRSQTPLELRIANLYTISNDLT
VGFVKA
GFTYVLLSRFDFQ
>AgerOR30
MLCATLDSLEDFYTRDGVLYEVKLEDKSELKKDREIFSEEMTKNLVVCVQHHKEIMWTA
DEVERIHRISV
FVLFLGGTLVICSSMFQLSVVQIGTLEFFMLLFYLICMLIEQFVYCWFGNEVIYKSAAIFQS
TYNTPWVD
CNVKFRKILLQFMTQTYRPISILTGGLFTMSVSVFINVVRTAYSYFTLLKGIQ
>AgerOR29
MGGWLLVYYTYTYLEGSFCLLHLVSPIVDSIINGTKLLPLVDCYPFPIFVSPTYQFMYVYQ
ALVLIWLCW
QNYNIDTLITGFLSFSALQCDILCDNLINLKLGEGEEDDEIKKMQEKLVACVEHHSRIDRFI
RDVEFCFS
WNILEQLACSVITFCTTMFKISLSEPMSKEFFTTIIYQAACFLQVFIYCWSGSVLEEKSEKIP
SAAYQCD
WVDASKGFKSTLLNFTQRVQRPLRMRTVLFPLSLDVFLQIIKSSFSYYTVLSTLNEK
>AgerOR28
MGYYPLTKGKYRKLQTMAATFFTMTSVIYLALGCIFAAQNVSDVRLFSATCAFLLTDLALI
VKMLNLLYH
RKNIFLIEDMLKNPLFNEIDAEEEIVLRQSLKMSKIIKIISATILITNILFETVYPLVYKKFFLL

MWFPF
NPDDYFYKVCGFETLIAFTGASFDVAVDVLTVLYMDLCAVQFVLLKHRLVQLGSTYTGDE
VMDDRLRVEK
LKKYVNHNDYTYRLCNVVEETLSIGVFVQILCSSAIICFSLFQLLLTPVKSIEFGLLIGYTID
MLWQLGT
YCILGQKVLDSSDTITEACYMSKWYSCSLEFKKNLLVIMNRTNKPVILLAGGVFPLNLNT
MMNILSTTYS
FFTLIWNMYNEN
>AgerOR27
MDIVDDQTKFYEQHLRRVEKFHMFVMSTGMLAFAMKPFVLSDMGSMFGCYVPPEVPYP
LYYIFELYVLSF
LSAGFVSFNVLICSLIILVVNQFRLLNYKIKAIDFSGIENGQGLDARVEELKHKIKYHQFLIR
C
>AgerOR26
MLQSRLFANVTERERTILENHIQEGRILAKIYRVLCFLVVLFYALFPFLDDRSGKTHRFPLPC
WFPFDES
KYYYQVFFAEILSIAVGAWVNSNIDILTVMLCILATAEFEILRNRLTTIIEPSPKSTKIGEDEAV
KVRLG
ECVDQYDELLCFVNQIEVTFSKGIFVQFFCSVIVICLTGFQMLVISFNSMQFVLLIVYFSCM
MCQVAMYC
WYGHTVMESSDYVRDACYMADWNRSDLSVQKSLVMIMERAKKPAMLRAGGFFVLNIPT
LMRILRSSYSYF
AVLQRLYSKNELQ
>AgerOR25
MMKFKVSGLVADLMPNIRLIQTSGHFMFNYHADNSGALHTLRLAYSCMHLVFCLFQFGCI
FGNLVVEKDD
VNYLAANTITVLFFTHCITKFIYFAARSKLFYRTLGIWNQSNSHPLFVESNNRYHALALKK
MRTLLICVM
TTTVFSAVAWTSITFVEESVHNVKDPNNENETITEEIPRLLIKSWYPWNAMSGMAYYVSLV
FQIYYVLFS
LSHANLMDSLFCSWLIFACEQLQHLKEIMKPLMELSASLDTYVPKSADLFRAPSAKSQDN
YIENDYNTKN
EELNLKGIYNTRQELGGNFRSGALQTFGQGGGGVGPNGLTKKQELMVRSAIKYWVERHK
HVVRLVTAIGD
AYGVALLLHMLTSTVMLTLLAYQATKINGVNTYAATTIGYLVYSLAQVFHFCIFGNRLIEES
SSVMEAAY
SCHWYDGSEEAKTFVQIVCQQCQKAMQISGAKFFIISLDLFASVLGAVVTYFMVLVQLK

>AgerOR24
MLEFILLSAQIALIVFEGSTTQFLDIVAICIMHVILLLAQMLLFYWHADEIRHESVAISEALYE
TDWYEY
NTSIKRTIHIMMARAQRPLSLTVGPFGDMSLTTALKILKGVYTYITFLQHSYGNKFGAKDK
>AgerOR23
MQWYEYNKSINTSIQIMMIRSQKPLSITVGPFGEVSLEMAVKIIKAAYTYVMFMKQVYEE

K
>AgerOR22
MGHVNFCVKYHTTICDFATKINDAFSTMMLVHITWTSFIISVLGFEIIMDTNYSNSVRFSLH
LGGWLGML
FLICFYGQILMDDSSTVSETVYQTTWYEKSPTVRKSLVLILLRSQRPLVLKAAGVNVMSLA
TFLGVLYNA
YSYFTLLLKIKP
>AgerOR21
MGTVGGGDVTVTSVSVTQTVSVTQSVAHSVSVTQSVAHSVSVTQSVTQSVSVSYVGGGS
DVVGVGFDNLG
LSNDHWLGYPHGYGGGDVNGDCLRNSYWNWMRFTNRYRSWHINWHWSGHFHVVGP
WYHNGSGYVDSHGHW
VWYTDGVSSWYVVRYLDGYWYCYRIGYVHWHGLWYWNGFSDGYGNGLRYSYWYLL
GQVAHFVGVLKETDM
AESVRAGIS
>AgerOR19
MTCALIFGCIGNLILKSKPLAGICYLMGYITALFLLCHGGQRITDETLSVADAVYNSRWYH
GDAQIMRDM
IIILARSQRPASLGALPLGAMNYSLFLMIIKTSYSYLTLLNQNT
>AgerOR17
MCLYFLTMILQVGMYCWFGHNMIASSDNINDAIYMSNWYEAEHSLKKSIMIFMEKCKKP
VVLTAGKIFPL
SLVTFTSIIRLSYSYLAVLQTIYSQE
>AgerOR14
MSLICAIVFGTIGNQALKSKPLGALLYLLGYVVALFLPCHAGQRLLDETLSVSDAVYESKW
YDCDVRTMK
DILFIIARCQNPQYLDALPLGSFNYPLFLLIVKTSYSYLTLLQQST
>AgerOR13
MGLSLAPLLEYKKCERENIEKNIQEICGLIGNIWLPIDLNGTPYKHMYYIFQVYCSFVLYHT
SSLISFSM
LETVGHVILRFRHVKNVFLEALSERQHSVRVEKFKNAVRYHVTVMRIAKLVNSSFNMCM
FVHVLLSGAVL
GCGGYRLLKDFSLGAICMCVGWFLAMSMVCYGGQKLREESMSIGDAIYESNWPHLNKE
LQKDIMLVILRC
QTPISLHGGPFGYMSYTTILTILKTSYSYLTLLSQTT
>AgerOR12
MLLKELLTHITDDIMVGSSDVEKFNSVHFQKTIMDRLKICAEHHSRLLNFGKNIESFCSLVL
VPQLIMTY
ASLVVNGFVLSTDRSDVSKTVILFNLSMSTLLQLILFALPSSQLNGQSLSVLDAVYDSKWY
LFNAPMKRA
CTFIMMNGQEGICIKAGGIAKIDNPLLVDMLQRVFSAITLLRALVEG
>AgerOR11
MSGLIMVVCAQLHILNDSLLNMREQAEAELRNAGVKVYRRMTKELQDKMNEKLVECVI
HHQCIMEFAKEL

TFLFTTSILGQFIVSVVIICITLFEITLVPVMSIKFFSMILYQFCMLLEIFLLCYYGNEVIRESTE
LTKC
AFCSDWTDCSWEFKRNLLFFMTSSQTALKLYAGGFFTLSLETFVKILKSSWSYFAVLNSVH
AGE
>AgerOR10
MGLAGYYPLAKDKHITLQRISATFFTFIALSYLILGIIFCIKNLSNLGLLSEACAFLLTEVTLI
VKMFNF
FYYKRNFLLMEEMLRSPLFTEVDAEEENILKENLKTSKIVEILTIIQVLVNISVETLYALFEK
KLFLLMW
FPFDPAEHFYAVYFFELIACFSGATLAVTIDVLLVYLLDLCAVQCTLLKHRLIKVGATFTGDE
AVDDRVR
IEKLEKYTIHHDEIYSFAGLVESSFSMGTFVQIVCSSVIICFCLFKTLITPLKSIEFGMLVAYSY
DMLWQ
LVLYCYFGQKVVNASETITEACYMSNWYNCSLKVQKHLLMIMNRANTSVVVTAGGLFPL
TLEMLMAILST
AYSFFTLIWKVYNDA
>AgerOR8
MASPFLQYVQYPMMISGFLPVALTESHKRLYTMYRYILRTFIFFFAFFLFGATPEFNASRDE
LFQFFNER
IMFIIVYMKIIMCMSKNLEKLIFEMIANETKISYSKNRKLTEIYRQTVRKSKIIQLSYIALVYI
CYVLLM
LPNFVTYVQIKVAESKANSTVNIYLTKSYYCWVPIEEDKHYLFLLLLENGFSLLSSNVYCS
VQIILINLL
LNMTLRLKVLGSNLKNMNNSGKRIVEKLITGYIEEHIDIIRNCEFLNDTLKYLMLLEFLLTT
VQLSMLMF
QGITTKDLNVKVFSAMYVLQLLVQLLILYWYAHQIRVESIAIADAVYEMPWYEYNRSTAIT
LRIMMMRSQ
KPLSLTLGSFGIMTLDTAVKIIKGSYTYVTFMQQTYGNID
>AgerOR7
MLFRLFINYWYANEIMVQSSEIGMALYKSKWYEESLKLQKMMIIMLMRCNKELCLEIGPF
AVMTLATFIG
ILKATYTYMTIIYR
>AgerOR6
MDLNEVLSAPLALLITSNVTVFCMNMYVLSTSQNLTESCRTMVTTFALVFEYFMVFGLPA
QLLMDEATST
ADIIYQECKWYLPSVKPLRSDFLIMIMRSQKKNCIRAANYHIISNQTVLLMLKTAYTFYTFI
QNVKM
>AgerOR5
MNCLKTQLEIVQCAFRSIRERCVKRLNLPDDYQIFVDDNNPVLEKVLYDELSHCTKHLNIL
LRIRDDIEN
VFTYVTLAQTLASLIIFASCLYVASTVSMTSPEFFAQVEYFLCVLVQLSIICFFGNEITIASAQ
TGVSLY
ECDWFSSSLRFKRSMILTISRIQRPVYVSIGKFSPLTLATLVTVCRGSFSYFTLFKSVQ
>AgerOR4

MTTSVTAMCVDMYILSKRQNIDDLLRICVGSSAVIVEYFLVYGLPAQLLMDESLATGDTIY
FDCEWYLPT
VRPLRTDLLIMIMQSQKKLRIRAANYHTVSNETPLLILKTAYSFYAFLQKVGG
>AgerOR3
MMYTLLEVIDILNTSDFNSMTFGLCYCATHLLGVAKIIILIVKKKKIREMLDKLETGLFLPN
TDRGGEEE
FRLINAAVTRCAKHAEIFNLIVYFIVSIRCLYALFDVGYSEELFDERLNTTVLVHQRTLPYRI
WLPLDTT
KSPTFEIVFFLQPVTLTLYGYYIGLMDSMVYGMMIHMNTQYLILKRVLDRYVFIATNLVSK
TMPGKGTQS
NGNGVISLPPGCERIDLLDEHVQEKVEEIVHYCAKHHVQILEFCEEVEIEFSYLMLSQFLFS
LYTLCFQL
YQLSLMGNVFSFDFISMCFYLTLLMYQLFCYCWYGNEIMVQSAKFSEELYNSDWLVLNIS
TKKSLLLMMM
RAQRPVKFTAGKFVLLSLQTFMAIVRGSASYFMVLRQMSA
>AgerOR2
MASPEFFAQVEYFMCVLIQMAMICFYGNEITVASEQTGVSLYECDWFSSSQRFKRSMMLT
MCRLQRPVYI
SIGKFSPLTLATLVTVCRGSFSYFALFKSVQ
>AgerOR1
MSMIFGGFYPIKDKKYKALRILSAVILMTLSSLYNLLGLIFGFQQISNVAVFSELISYLLTGLS
FSCKIV
NVFYYKNNLLLLDDMLRNPIISELETEEEEMVVKNNLKFGQTLKKFYKIYITSIFAVQFLYP
LINNSDHK
SLPLQLWFPFNQEEHYYTFYCIETLFIFSPCYFNVILDLLNILLMDMCAAQFELLKHRLKQF
GADFTTGE
AVEDDKLLFEKLKKIITYQNHVYRCSELVGETFSMGSLCQLACSVVILSFVIFKTAVTPIKS
MQFLKMAT
YSFLMMTQISLFCYYGQKVLNSSSTVAEACFMSKWYNCSLRIQKHLVIVMNRGNQPVIM
KAGQFPLTLQT
LMSIWSSAYSFLTILMQMYRKGDS

>AglaOR49b
MLTGYSFFVLFSFNIKSAAKLFILLSDFNKFGKPPKFDERNEQLNRFSRYHYIYINLASFAFL
SVTNIFK
SHQCIKENQEYNFNEVCGLITNAWLPFDIDYFPLKQIYATLQVFSIFYVYVTAGTVTWLVV
EVVEHISVR
IDHIKHLFTSALKEKDPEEMKRNFRFAVQYHSWFLELEDELNKSFSIPMFSMMLLGAPILG
CAAFGYMEV
TGANSLLVICLSWFNALALVSFGGQRLINENLTIAEEIYNSKWVDVAPILGKDIIIILTRCQKP
MKLRAA
AFGVMNHAMIVSVSRAAYSYLNILRASSK
>AglaOR85b
MSTIEDYDLRNAFKIERKLLLLCGIYPNEGRINKKLYNLSAFCHISFSLLITLSMVIFLAMN

MKNILSVV
EALLFLATQMAFLCKLFNVLNKKHKLLEIEDILANPAFYGYPKEKRHLIEDSVRFTKIFGM
CYRSICTVV
SITYAIFPLMDDDEWALPLSGWNPIQIDTKFKYWTIFTFQWVSYYMSVYINSGIDILIYILIT
VVTSQFE
ILKDNLTNIRYETDTAKRDFAKNVVLHYGILKLVRVIEDTFSYATFFQFFSSVVVICFTGFEM
MIVPPNS
IQFISMCTYFNAMIFQVAMYCWFGHSIIASSDKINDAIYMSNWYEADLSLKKSIMIFMEKC
KKPVVLTAG
KIFPLSLVTFTSIMRSSYSYLAVLQSMYGQE
>AglaOR1
MFSRDLTESTFKYNIAIMKIFGLYPFDNWPKVSILYGFISYVVLTFITAVLVVVLLIVRIKDSV
QILSED
GFIMVELIVLSVKILPCKLNIKGIKRTMHALKQEIFNSQLPEQDRILAETIDNCIFIFLTFCTCS
VITVS
LWACVPLAYEARRLPIAIWLPFDPFEDTAIYISLYIFLIYVVVNGGVENVCIDTLLAFQVYH
AASQIKIL
KDTLAHLGERAEEQILKEDKSLSLEDKDNLKNNIIYKKICHCVDHYEAIYRFVEDLETTYS
FIVFSQMIA
TIILICVCCLRFTVDIPFTMPFFGTATFTAAALIEIFLYCYSGMLLYEESNSIINDIYMSEWYTY
DEKSK
KALLTLMERAKRPIKVTAGKLLDLSLATFATIIRRSYSLLAVLKNY
>AglaOR2
MLKNTSNAIKIQKFILTLTGLWPEEHPTLYGKISGRISVVTAIIFTATLIAEAIKQIGNYVVLIE
HLSLI
ISPTSFLIKLIMFLRKTGQFVRLYRNLDMDIFNKHPDQFNTIKRKSETTSAVIGLSYMFSCFV
ITFFFCA
RPLYTSANMPVRFSFEMGQYKPIVAVFQIFCMFNAALSNSCLDVIAMTLMGIASVQIDILN
RNITNFKKE
CDESATGTDGYIRYLNHCVKHHNEIIRYIGDIEEVFSLVFLAQYLTSGALICNIGFLLVHIRG
LNLQFFN
TVFYFAAMMCQLGMYCWFGNEIIVKVNICAIFLSSDTKTACYESDWIDCEVKVRKILIIIM
ERSKRPLFL
TAGKFSVLSLNSFTTVMNSSYTYFALMQKLYSKTNN
>AglaOR4
MKMKRHIDLLKFIKEERTCMVFGGFYSIEKYKVLHNLSAILIMTVVSLYNLLGLVHGFQHI
SNVAVFSQS
IAYLLTGISFSCKMINLIMHKNNLLLLDEILQNPIFTELETTEEEVVLKNTLKFGQTLKKTYK
LYTSATV
TVQVLYPMINNPGHKNFPLLFWFPFNPEDHYYKVYFAEILMIFCICTFNVTVDLLNVLFMD
LCAAQFELL
KYRLKHFGREFHGGEAVNDRTLYEKLNKIIVHQNLVYRFSKLTEETFSAGVFCHLACTVIV
LCCAIFKAV
ITPINSMQFLMMATYSFCMTLEVSLYCCYGQKVLDSSSTITEACFMANWYNCNVKVQEDL

VIIMNRANKF

VTMKAGGMFPLTLETLMRIWSSAYSFLTLLMQIYNENY

>AglaOR94a

MVALKIAGFYPLRGNKYKHLHTISATYFLSITILYIILACIHSFLNLTNITELSETVTFLMTILA

YLGKI

LNLFFYRKNLIKLEDMLQNPILTKLETEEEENILKRNFRYSRLFTNIFKAKSATAASVHAVY

PLLGDYGS

KNFLFLLWFPFDPKDYYIPVYFFEMTLLLSSTWFDITMDTLNILMMDLCATQFEILKKRLIR

IGTSFTGD

EAIDDKLRLRKLRKYIIHHNYIYSCSELVRDTYSIGEFCQVGCSVMVVCFGLFKLLIIPLKS

AQFLMLVT

YSTTMIYQISLYCCYGQKLLNASDTVTEACYMSRWNDCSTQVQKYLAMIMNRANTPFLM

KAGGIFSLTLE

TLMTIYTSAYSFFAILWKVYHSEDQAA

>AglaOR33b

MKKFQLKEYLHYNLVVMKYLGLWPKKDFTSDKSYLIYTIIVNGFFNFTTSVGLTGYILTSS

NLLEDVIGA

GYIILAIMATAKTFFIMKYSKMFKLLVDAEIHRSVNVELNEEQTKILCDYVGFWKKVHLIY

RPVIVSISG

YSKLVESYFSKYLLIQFTSSCMSTALIMTSMSMNHDDNADIWFLSVSQLGVWADLYIYCW

YGNEVTEKSK

KIPYAAFESNWVTASKGYKKNLLIFICRTQTPIKLYAVDFFELSLNIFISILRTAYSYYMLLRQ

LSADE

>AglaORco

MMKFKVSGLVADLMPNIRLIQASGHFMFNYHADNSGALHALRLGYSCAHLLFCLFQYGC

IFGNLVVEKDD

VNYLAANTITVLFFTHCITKFVYFALRSKLFYRTLGIWNQSNSHPLFVESNNRYHALALKK

MRTLLICVT

ATTVLSAAAWTGITFVEESVHNIKDPDNENETITEEIPRLLIKSWYPWDAMSGMAYYGSLI

FQIYYVLFS

LAHANLMDSLFCSWLIFACEQLQHLKEIMKPLMELSASLDTYVPKSADLFRAPSAKSQDN

YIENDYNAKN

EELNLKGIYNTRQELGGNFRSGALQTFGQGGVGPNGLTKKQELMVRSAIKYWVERHKHV

VRLVTAIGDAY

GVALLLHMLTSTVMLTLLAYQATKINGVNTYAATTIGYLVYSLAQVFHFCIFGNRLIEESSS

VMEAAYSC

HWYDGSEEAKTFVQIVCQQCQKAMQISGAKFFTISLDLFASVLGAVVTYFMVLVQLK

>AglaOR59a

MGAIKVVFFYFQKHKLLAIMDTLENKEFRYDSCTEKSFFPGLISRRYKNIGVKYTILFFILA

HATLFSSY

LPPTISTLMNSSRRGAEGQGLYVLPYYSWMPFKYNTDDSFLLALGYQAIPMFSYAYSIVG

MDTLFMNILN

SIGFNLEMIQGAFLTIRNRIIKQTGKSIYKSSILLDSEELKLKLCNEMKKICHHLQIIYKVCED

LENVHK

YLTLAQMTATLFILCSCLYLVSSTPIASKQFYAEIVYMVAMGFQLTLYCWFGNEVTLKAAE
LPLYIWQCD
WLTADNNFKASMILTMARARKPLYLTAGNFAPLTLSTFVSIIKGSYSFFTVLKSTNE
>AglaOR30a
MIETGTGCKEARIVEWIPEQTMQQNYDFSSYFRPSIIILKILGFWRPERNMKFKGIYNCYTA
LCSLIWVQ
FLLSQIIYIINNRNDVQEVTAVLSVTVTFTINLIQMMFFYKNTNYLKILIKEMNRPLFQVKC
QKHYHIAK
NTERMYKLMFKSCLYLAILTDALVTVVPLMGKEKKSSIKGWFPYDYTKPLYFILTYIFQKL
VFIWNTFIC
LNTAMTIIGLLTQFGLQCDLLCCTLDSLDDFYTEGNVLYEISLEDKLKLTKDRERFSIEMTK
NLVICVEH
HRQIIRVVKDVERISGTGLFILFVGGGLILCSSLFPLSVSSLILQSAFNTPWIGCNVKFQKIML
LYMMKT
SKPMSILTGGLFTMSVPVFVSTLRTAYSYFTLLQNIQ
>AglaOR13a
MYEISKEKSFYSSLTILRYTYAIPSATEKSGLTWFRFIFIVIRLASLLNFVILPCLHLISTVRAK
TGVDI
SEDLSTMFGAFGFITNAVTFLILYEKWSQFFIDVEDCNRFEIPEGIEERKKKLNLYSLLYALY
AICGVLI
YGVIASSETSYCKRMNEEHGLSETCGMFTPIVLPFDGSNVYIRSIIFGIQMLLGMFTLPSTAL
ISFLLYE
GVETLILHITQLKKSFIEVFDVASNAERKKRLRFCVYYHINILSLCQRLSTLGKYTSGFLCM
ACALVFGC
IGNLILKSKPIAGISYLLEYVFALFLLCHGGQRLLDETLSVADVIYDTKWYDGDVQIMRDIR
FILARSQI
PVMIGALPLGCMNYALFLMIMKTSYSYLTLLTQNT

>AmelOR4
MENISGIAKAEEDLKYATRFVKPIMGMIGAWPISPSTSFLLKVLQRLRHIFTYFLFFLIMIPTL
MYVFLK
EKNNKVRLKLMPPIINCSIQCFKYTIILWRRKEIQEGLYAIKHDWIKATEEERLIFRSKAKIG
RRVVLVV
AFTMYGGGLCYRMILPLLKGTIVTANNTMIRALPCPSYFFILNEQQSPIYEILFVLQIIAGIAI
YAVICG
FCGIFALLVLHAWSMLRILVNKIKKLVDKSDMSEVVLQRKIMDIIIFNRFLKNIETITEYICLI
EMIGST
CMICLVGYCILMEWENTNTMAIVIYITIQISIIFCIFILCYIGQLLVDENYIVSQASSTINWYRL
SIKNM
RCLILIIAMSNYPMKLKAAKMMEMSLITFTDIMKVSMGYLNILREII
>AmelOR57
MHVSVRDPINELRNPNYEKDIAYVTKHNKWVLASIGIWPTVLKNIGKILPKIVIGFNNLLC
FFTLTQSAL
HIILEQKDTLLRLKFLGLIFFSFMSMMKYWALMIRKPEIEHCIEQVQLDWKQVEIENDREL

MLKYGIIGR
NLTIYSILFMYLSGIIYVSIMQYAMGSQINEHNQTIKMLIYPAYGGYNIQKSPTYEIIYGVQCI
CEYVFD
TIASGACGLAALFVTHACGQIDVIMSRLDDIVAGQYKKNSNANIRLMEIIKHHTRILKFSAV
VETVLQEV
FFLEFVSSTFVICLLEYYCITDWEQKNIISLTSYILLLISMTFNMFLLCYIGDLLIEKSGNVGV
AVFMID
WYHLPTKTIQNLILIMAMSNTPAKLSVGRILDLSLSTFGNVLKTTFVYLNFLQTAVM
>AmelOR170
MNFQNLNRLNAFANMVSGNFLPMTNINEKLSTILKIYFVVAWIIELIYVAASFLGLFNVSGE
KALKDGTV
NIAISFEVIVFNIYLHSRKKLLHKLIGKLNHLLITEDEIFRSVIIDTVKPLEMPLKIYVIASVAS
LMIWI
LSPLIKLFQKDEFYYEDFIMPAVFSKQQPFSNDVFICGIFLQLLGGEDTIIRKISLDIYTIYLCL
LITAQ
YKYLRIKFAIILKEEREITKDHYKNIIWRNDNVRQEMKLVTRHFETVIETTTILKKLISPNIGF
LYLSYV
FRFCFLSFMFAMTTAKYFEKCLLASYTIGALIQFYILCYCIQRLFEASSSIADDVVYEKWYY
YDVRFQRV
ILMISLSNELKCKISNFQNIDLTLPTFMSILNQAYSVCLLFLKARQD
>AmelOR115
MDFAMGWNRFNLTLLGVYPEPRKMSRNSRLMSSLIFWFTTLVTFTFICAPQTANLILKSTS
LDEVIENLS
INIPIVFALIKQIVLRYYKKALTELLGEMLADWSGPIGDQDRETMLRNARLSRAISIVCSTLT
YFMLLAF
VSLQVWSNAENASETDLGGLLHPATFPYETSKSPNYEITWLGQLMGTVLTAICYSCFDTFL
AVLVLHLCG
QLTVLGTALEDLVNATRRNDYKTFEQRLSSIVNRHNHLSRFAVIVEDCFNITLLVQTLICTA
MFCLTGYR
MITSVDREDEADVPIVGIIFFIIHVIYTMLHLFIYCYVGETLLGQSTGIGLSTYHCNWYDLPS
RRAVLLM
IVIRRANVSFQITAGKFSPFSLEFFNAVLKTSAGYLSVLLAMKDRLVEGK
>AmelOR50
MTNDINVAKQRSDNLSEYSIKLSRWYLKPLGAWPASSSTTKMERIISQILIVICWCIILFTVIP
GILYIL
FVKQDIYVKLKIFGPLSHWCIDGFNYAILLLRKNDILHCIEHLRADWKLITRTQDQQVMLR
NAKMGRYIA
AFCAIFMQVIIFFTCFILGIFKRSIHIDNKTVELYNLPCPAYKIPFDTDPTIHDIMLGTQFLSAF
VVSSS
ASASFTLATIFTCHVLGQLNIMMIWINEFVDRLQRKENKDNHINKIGVIVEHHLRILSLIARI
ERITCPI
YFMELFKCMMGMCMPSYYFLAEWSERNIQNLTIYVMVALSMSFNILLVCCIGEILREQCK
KVGDMVYMTN
WYQLPDKDILNLIMIISRSSVEVKITAGKIITMSIYTFGNIVKTVFAYLNMLRQITMM

>AmelOR52
MFDRSYNNSQLKNIHYENDIHYTLQMCQWLLKPIGVWPFVYDRTSRFEQLISIILMATCFS
SLLFIILPS
GHHIFFVEKDMHLKVKLLGPVGFCLSSTIKYCYLGVKGVFFEQCIKHVKNDWKMVQDPS
YRIIMLKYATI
SRKLIIMCAVFLYTGGMSYHTVMQFLSKEKDNNNTFKPLTYLGYDPFFDTQSSPIYEIVFC
MHCFAAMIM
YSVTTVAYSLAAIFVTHICGQIQIQATRLQNLVENKDKKNNCDPFALIVHDHVEILRFSKNV
EEALREIC
LAEIIESTIIMCLLEYYCMTEWQNNDAIAILTYFTLLISFTFNIFIFCYIGEILSEQCSQIGTISY
EINW
YKLPAKKAHDLILLISISQYPPKLTAGKIIDLSFNTFSSVVKTSVIYLNLLRTVTD
>AmelOR53
MHDRSHDNINGQLKNSHYKSDIHYTLQMCQWLLKPIGVWPLIYNQTSRFEQLISIILMGTC
FSSLLFIIL
PSGHHILFVEKNLHMKVKAFGPAGFCLSSTIKYCYLGLKGSSFERCIEHMRKDWMMVQD
PNHRTIMLKYA
TISRKLITMCAVFLYTGGMSYHTIMQFLSKGKNKDNYTIRPLPYIGYDPFFDTQSSPTYEIV
YCIHCFTA
MIMYSISTVAYSLTTIFVTHICGQIQVQIARLQDLVESKEKRKYKDCDPFALIVHDHVEVLR
FSNNIEEA
LREICFTEIIECTIDMCMLEYYCIMEWSVGDTITLLTFFTLLISFTFNIFIFCYIGEILTEQCSQI
GTVS
YEIDWYKLSPKEAYDLILLISISQHPPKLTAGKIIELSLNTFSTVAKTSVVYLNLLRTVTDW
>AmelOR56
MYLSIQNPINEPRNPNYEKDIAYVTKYNKWVLTCIGIWPIILKNINKILPKIVIGINNLLCSFI
LIQSAL
HIIYEEKDVLLRLKILGLIFFSFISLMKYWALTIHKPEIKYCIEQVQSDWKQVEMENDRELM
LKYGILGR
NLTIYSILFMYMGSITYMSITQYAMGLQFNEHNQTIRVLIYPTYGYNIQKSPIYEIIYGVQFM
CGYVVDT
ITSGACGLAALFVTHACGQIDIITSRLDDIVAGQFYSKNLNPDIRLMGIIKHHIRILKFSAVVE
TILQEV
FFLEFIGSTFVICLLEYYCIADWEQKNIISLTSYVLLLISLTFNMFLLCYIGDLLIQKSSNIGVA
VFMID
WFHLPTKTIQNLILIMAMSNTPAKLTVGRIVDLSLSTFGNVLKTTFVYLNFLQTAVMQ
>AmelOR58
MHLFVRDQTNQPRNLNYEKDIVYVTKHNKWILNSIGIWPTVLKGIDEYLPKIAIALSNLVL
SFTVIQCVL
HILLEQKDPILRLKILGLTFFSFISLMKYWVLTMRKPKIKLCIEQIQHDWKQVEFERDRKLM
LKYGIIGR
NLSMYSIVFMYSGGIIYHTVMHYKLGSYVDEYNRTIKLLIYPTYSRLYDVQKSPVYELVYI
LQCICGYMF
DAVTVGACGLAALFATHICGQIDIVMAKLEDLVDGKFSKENSNPNIRLVEIIEHHIKILRFSA

MVETVLQ
EVCFLEFIGSTFVICLLEYYCITDWQQNNTIGLTTYSLLLISLVFNIFLLCYIGNLLIEKSSNIG
IVCCM
IDWYQLPIKTIQGLILMIAMSNSPAKISAAGIADLSLSTFGSVLKTSFAYLNFIRTTIM
>AmelOR63
MLKKMKTTSNKDFAYAMTPLKFLAWPVGTWPLQVFNTFSIIRATFSTFLLLLMLTILQVEL
YLDSSNPEY
NLDALILINAGILAVTKVICFHVRSLGLVSNFTSAVKDYKELNSEENRVIVRRHAYMGRAA
CISLIFCSY
VGCTLFMIVPIVAGDKEEVINVTEESAMKYPVPFENTLILINMPENMYFLIFIVEYLMLLLT
TTGNLGSD
SLFFSIVFHLCGQVEILRLEYNKLSNENERTTKHITLLIKRHIYLLKLGDMLNKTISSILIVQL
SSSCML
ICTTGFEFILALSIGNIVMIVKTFAVICVLLIQLFAYSYVGEYLKTQTEGLGNSIYFCTWYDM
PKNVSHN
ITFIIMRAQHPVLLTAGKFFVINMETYMSILRISMSYLSVLRVMVNS
>AmelOR98
MLISMIAIFIPTTFEIYVSIHDKNTDAVMECLPNLCASLSSVVKILNVHFNRENFNKLLEFVV
KEWDELK
LNELHILEEITIQGSKIAHLYRNTLLSFLILFLLVPMYFPILDMIDALNQTRSRQQLLRVNYM
VFNADDY
FFYVYLQLAWGAIVIVMIVITVDSLYIIIIHHVCGLFAVCSYEIQKTVKDLTVFTDIEKCSYK
ELKNCVI
KHKKAIKFYNILNNSSQLSYLLQIGINIMGISTTAFQLAVNLDTRPQEAIRNAVFCGANQFH
LFVLSLPG
QILLDHCAELSNTIYCSMWYKLPVKIQKMFNIMLMRSKKSCALTVYGLYELNMENFGTTF
KACISYFTMM
LSLK
>AmelOR105
MSMLQKSNEQEYNAFDIAYYKTLKLYLTICGINPYQNNSISIIIIIMIISVCMSFLCPTSIQLWE
AISNK
DFDNIIQNIPQVITVIASMIKILNIYSNKMQFKNLFYSLAQDWKLLESKEELIMLDKFTQYG
SKLALLYR
RTLLTFLVIFLFLPLCNPILDVILPLNETRSRQNIFNVNYIILDNYEYFYIVYMHLSCSAVIIVII
IISV
DSLYISIIYHACGLFAACGYQIQKLTKVHTIEKNGPNISNIDYEEFKQCVIMHYKCLQLYDV
LEKCCRNL
YLIQMGLNIMIISVTCVEVVVFLDRPKEAIRAIIYVIAQQFHLYAISLPGETLLNQSSKLADKI
YDSEWY
KIPMKVQKVLHIMQIRSNKPCILTAAGLYEMKIESFGITIKTCMSYFMMFLSLRE
>AmelOR107
MDQQAMEELYLKDNKFFGQLVGVWPDQGKFMKFLMRFIILIVMIIAFIAQISRVAVFYSVD
VLSDQIPYI
DLGFALMLKQYNYILNEKKLRELLHNIISDRLVKRSKEEEEIFEIYFKRAMFFCSFYEVSIYS

CGFMFLS
MPSIPLIMNVIMPLNESRSRELVYPSYYFVDEQKYYYLITGHMLAVCLGHVFVYIACDINLI
HVVHHGCA
LLTISGYHFKHAMDNVDLCNEKYSDELMEKTYAKVSQSIDAHKKAVEYVNKIDACHIHY
FFILLGMIIVT
FTGTFIKLTSMEIGGRFFTFCTFTIGQLTHLLFLMVMGQFLIDSNEEVFKTIYDARWYYGSS
KTQSLYLL
VLRKCLNPPKLTGGGLIALNLDSFVKVLKTSFSYYTVFRSS
>AmelOR131
MDVFDKYYHTYRIVLKIIGLWPYNNSVYVWIQRLCISALFLGNIIFQILSLIRSEITLRNCILI
LSTTCP
LIIILLRYISFIIFFPMVKLLFHHICVEENAVQDLIEIQIRMKYIGNSRHMIEILLRVTFLTITLFS
IFL
LYFVTMDFIMPLNEFHRHILLYVTLFSVNRTIYFYILYLNFLFVITFGLLSLICTESIVGLYSY
HTGMLF
KIISYRIRKIITYLTMFNVSSKQIDSKLAELHRVVDIHNQAIGLVVNAITIKKDQLEILITLIIFA
NHLM
IMFLCNYNGQILINSNEEFFHELYIPVWYFVPLKVQKILLLIMIRSSMACIFHIFGVFIPCYVG
FTTMLS
TSFSYFTLIYSIQ
>AmelOR130
MNVFDNQYRIYRIILKIIGLWPYDNSIYVWIQRLCLLSYFFANIIFQIVSLLRSEITLQNSILIL
SITCP
LVLFLLRYIGSIACFPTIKIVFKHIRTEENIVQDSIESQIRMKLIDDSHHIINIFFWMTYTTIVIFI
IYV
SYPIILDFMIPLNESRTHFIYYITTFSHNQSIYLDILDFNFMFTGIFGLLSVACSESITGIYSYYI
CILL
KIVSYRIQKIIMYLAMFKLSPKQIDSKLIELYRVVDIHNQTIELLVNATLIKKNQLEMLFCFT
LVAIHLV
IIFLNNYNGQIVMNSSQELFDELYNSMWYFMPLKAQKILLLIMLQSTTKHAFNILGLFTPC
YAGFSTMLS
SSFSYFTLMYSIQ
>AmelOR10
MVQIRNAKEGLRHTFWFAYPFSRMLGHWPLSVSSSAFSKILNSFIIFISYLLQMIVVIPSLLY
VILKEKN
PKKKIKLLMPHLNSIVQMIKYTILLRQMKLIDKLLDEIKKDWSIATEENRRIFSRTASVEHK
LTSIIAIT
IYSGGFFYRMILPFSKNKIVSNNMTIRLLPCPGYFGFLDEQVSPNYEIIFILQVFGGFVIYTAV
CSTKSI
CLMLCMHMCGLLKILTNKVMELTNDNDERVVQEKIVHIVEYQMKIKEFLKQIDQFVPTIY
LFEVFIQVLI
MCIIGYCIIMEWKESNGMGLITYVIVQMTCLIGSFSVCYVGQLLIDESENIRQAFIALKWYQ
LPVKKSRS
LILLIIISNYPIKVTAGKIIDLSLVTFITIIKTAVSYMNMLQQIT

>AmelOR51
MRSSNIDDLPLNDRYESDIHYTFQFCHWILKPLGIYYFIYNQANKFEKILSMVLILICFFIIQF
VIVPFG
YYILFYEKDMNTKIKFLGPLTFCLSALFKYSYLGIKSSELGHCIKHVEKDWKMLQNEDHR
VIMSRYVIMG
RNLITLCAAFMYTGGLSYHTIMPLLSKRKVENFTIRPLTYPGYEAFLNIQKSPTYEIIYCMH
CIYVIVVG
NITMAAYSLTTIFITHACGQIKIQMLRLENLKNEKKVLETGIESHLAVVVKNHVEILRFAKN
VETTLREL
FLVEVIVSTLLMCLLEYYCMVEWETSDSAAILTYVILLFSFTFNILIFCYVGELLLGQGSEIA
TALYEIE
WYNLPGRKARDIILLLVISKYPPKLTAGKIFILSMNTFSVVSLKIISSLFEHVANDH
>AmelOR5
METKHTEKDLKQAFYAQSFLKIVGVWPIPIGSPLSSKIRNWFITFFSLFLQICIVGPCILVMFL
KEKNGK
RKINLFKLLTNTLNQLFKYIITLNRANELAIAMNEIKNDWLTATSEDRWIFTANSKMGQKV
MLIVAVTVY
SSGLGYRMLLPILKGKIVLPNNVTIRLLPCPTYFTFFNELVSPYYEMIFMLQLLAGFFSYTV
LNGTVGIS
LMLSLHMCSLLKILTRKMANLTDRSITSENIIQEKIVEIVEYQTKIKRFLGNAELITEYFCFY
DIGCNMC
LMCFIGYSAILEWENHNIAAIVVHFMLLGTCIFIIYIVCYIGQLLLDESNNLAQQCITLSWY
HFPTRKAR
CLILMIIMSNYPVKLTAAKVVDVSLTTFTDVMKAAMGYLNMLREVI
>AmelOR13
MGQPYSLKLVYPLLKILGAWPKSSPSSVLSTILKCCLISICYLIQLMVLIPGILYIFLKEANLG
GKIKMF
VPHMNGITQVSKYTILLRQIKEFNIILKEVKRDYSLATDKNMWIFTTRAYIGHKMMIAIAIA
MYSSGVGY
RMILPFLKGRILLPDNTTVRLLPCPGYYMFLNEQVTPNYEIIFTIQVLGGFLNYTTLCGTTGI
TTMLCLH
MCSLLEILINKMNDLTCQSDECEIIVRKKLADIVEYQMKIIDFLNHVEQLTSYLYFCEILEYV
CGACVIG
YCLITEWENSNAAALIVYFILEFLCIFCTLTICYIGQLLIDESDKVRQISVTLDWYRLPVNEA
RGLILVI
IMSNYPIKVTAGKIVDISLITFTDIVKTSVGYLNILRTVA
>AmelOR14
MSRVGKAENGMRHTVWFAYPLLRILGAWPNRVSSSTLSKIFNWYLIFTCYTLQLIVLVPGF
LHVFLKEKN
GRKKMKMMIPQVNGYLQLCKYSLVLRWTNKLRVLLNEMKEDWLNTTEEDQLIFRAKAS
FGHRVMSMIAIV
TYSAGLGYRTILPLSKGRILLPNNTTKRLLPCPGYFVFFNEQVSPIYEIIFIIQVLGGLLTYTI
MCGTIG
MCVMFCLHSSSLLRILLNKIYQLTKQLDVNEVVVHEKIVDIVKYQTKVKGFLKNVEQLTT

YLFLLEIMVE
TSIGCVIGYNVVTEWEDSNAAAMIIHLMMQVSTISCTFIMCYVGQTLIDEGNNVRRMSITL
DWYRFPVKE
ARNLILVIIMSSYPVKLTAGKVVDISLATFTDIIKTTVGYLNMLQKVT
>AmelOR25
MEKQQYVIAQDDGKKANLSIQWNRWLLTPIGAWPNLRKSRIGKCYSLLISIICYGLIGFML
VSCSMFLMV
EIKKVYNRIKMIGPLSFFLMTFMKYYLLLLHENDIREGIECIEWDWKNMKHQEDRNIMIEY
ANYGRKLVL
ICTFFMYSAFAFYYLVLPFSVGKIEDGNLTFIQLPFPSSSLIADIRYSPYNEIVLSVQILTGVV
MHAITS
AACSIAAVFAVHACGQMQVLMNWLDHLVDGRSDMSKAIDDRIANIVIQHDRILKFLALTE
KALQQISFVE
FLGCTANMCLLGYYLIVEWNPKEIILSVTYVALIISITFNIFIFCYIGDGVAEQCQKVGEMAY
MIEWYRL
TGKKKLCCILIIAMSNSSVKFTAGNMVELSIYTFSDVVKTSVAFLNMFRALT
>AmelOR2
MKFKQQGLIADLMPNINLMKATGHFMFNYYTDSSTKHIHKIYCIVHLVLILMQFGFCGINL
MMESEDVDD
LTANTITMLFFTHSVVKLVYFAVRSKLFYRTLGIWNNPNSHPLFAESNARYHQIAVKKMRIL
LLAVIGTT
VLSAISWTTITFIGDSVKKVIDPVTNETTYVEIPRLMVRSWYPYDPSHGMAHILTLIFQFYW
LIFCMADA
NLLDVLFCSWLLFACEQIQHLKNIMKPLMEFSATLDTVVPNSGELFKAGSAEQPKEQEPLP
PVTPPQGEN
MLDMDLRGIYSNRTDFTTTFRPTAGMTFNGGVGPNGLTKKQEMLVRSAIKYWVERHKHI
VRLVTAIGDAY
GVALLLHMLTTTITLTLLAYQATKIHAVDTYAASVVGYLLYSLGQVFMLCIFGNRLIEESSS
VMEAAYSC
HWYDGSEEAKTFVQIVCQQCQKAMSISGAKFFTVSLDLFASVLGAMVTYFMVLVQLK
>AmelOR26
MMNQLNEQSVLMPVSYARDYEYSIQVNRWLLKPIGAWPNLTKATRTEKLLVKLLNFICHS
LIIFTVMPCI
MYIFYEDESLKTRMKAIGPTSHWLMGELNYCCLLMRAKEIVYCIEHIKYDWKTVRRARD
RELMIKNAKLG
RFIACIAALCMHSGIMSYTVITGFKKITFQIGNDSYSMYRLPCPFYTNLLDVRFSPMNEIVF
ALQLLSGF
ISTSVTVGACGLAAVLAMHACGQFNVVMIRSDKLVKDNNEKKQDEQTLHKKLGFIVEHH
LRTLSLVWYME
KVMNMICLVELVGCTMNMCILKYYFLTEKSKTILGIYAIVYASMVFNIFIFCYIAEIVTEQG
KKVGEKFY
MTEWYQLPHKTALGLVLIISRSSMVIKITAGKLIQISIATFAAVFKASFAYLNMIRTIAM
>AmelOR11
MVQIRNAKEGLKHTFWFAYPFSRTLGYWPLVSPSAFTKFFNSFTIFTLYFLELIVLIPGLLYV

LQVKNPR
TKIKLLMPHLNSIAQMAKYTIILQRAKEFSKLLDEIKKDWLLATEENRQIFSERASIEHKLT
TVIVVTMY
GGGFFYRTILPLSKGKILLPNNMTVRLLPCPSYFGSLNEQATPNYEIIFTLQVLGGFIIYTVL
CGTKSAC
LMLCLHMCGLLKILTNKVMDLTNDSDEQVVQEKIVHIVEYQTRIKEFLNQLDQFVPAIYLI
EVVIQVLII
CIIGYCIIMEWEDSNAMAMVIYVVFQVTCVIGTFSVCYVGQLLLDESENIRQAYNTLNWY
RLPVKKARSL
ILLILMSHYPIKVTAGRIMDLSLVTFTSIIKSAVGYMNMLRTVT
>AmelOR12
MARIRNAKDGIRHTFWFAYPFSRMLGYWPLSVSSSAFAKISNYFIIFLSYLLTLIFMVPGLLY
IFLKVKN
GRSRIKLLMSHINGIVQMAKYTILLRKTKEIAKLLDEIKKDWMTASEENRQIFSTRASIEHK
LTMVVVVT
MYGGGFFYRAILPLSKGKIVLSNNVTIRLLPCPGYFGFLDEQVSPNYEIIFTLQVLGGFVIYT
AVCGTKS
ICLMLCLHMCGLLKILTNKVMELTNDKDEKVVQEKIAHIVDYQTRIIEFLNDLNQFVPSVY
FFEIILEVL
IICIIGYCLITEWEDNNTMATVIFVIFQITCFIGTFAVCYAGQLLVDESENVRQACSTLNWYR
LPVKKAR
SLILLILMSNYPIKVTAGRIVDVSLVTFTSIIKNSVGYMNILQQVT
>AmelOR9
MARIRNAREGINHTLWFAYPLSKMVGCWPLNIPSSTFSKIFNAFIIFISYLLSLIVLVPGLLYL
FLKEKN
GRRKIKMLMPLMSTIAQMTKYTILLRRMKEFNKLLDEIKKDWSTATQENRQIFSAKASIEH
KLTTVIAIT
IYGGGIFYRMILPLSKGRIVLPNNVTIRLLPCPGYFGSLNVQITPNYEIIFTLQILGGFVIYTAL
CGVKS
SCLMLCMHMCGLLRILTNKVMELTSDKDEKVVQEKIVYIVQYQTRIKEFYNYVDQFVPY
VYFIEMIVGVL
ITCVLGYCIIVEWEDSDAMAIIAYVVLQVTCVFGTFSICYAGQLLVDESENVRQACNTLKW
YRLPTKKAR
SLILLIIMSNYPLKVTAGRIVDVSLVTFTSIIKSAVGYMNILQQIT
>AmelOR30
MEKNRSIIGHDDYERNVNLSIRWNRFLLKSLGTWPNLRESRIGKCYSVLIGIVCYGLISFML
TSSNMFLV
VEVKDTYNRIKMIGPLSFFAMTLIKYYFLTFHEENIRKGIEHIEWDWKNVKHEEDKRIMIE
YANYGKKLA
LISIFFVYSAFVFYYFVVPISVGKIRDENLTFIPLPFPSSKLIADMRQSPANEILFSVQVLSGVII
HAIT
ATAVSIAAVFAVHACGQMQMLMNWLECLVDGRSDMNKIVDKRIAKIVVQHDRILKFLALT
ERALQQISFV
EFLGCTMNMCLLGYYLIVEWNPKEISLSLTYISLLISFTFNIFIFCYIGDLVAEQCQKVGEMT

YMIEWYR

LTGKKKLCCVLIIAMSNSSIKFTAGNMVELSIYTFSDVVKTSVAFLNMLRALT

>AmelOR19

MNMEHFIVEKKSYNASYKNDLFFNVQLNVWTLRTIGTWPKSLDRSWLETIEHVCLCFLN

YVLLAFILIPG

VMYFLLEMKDFYDQMKLGSALSFFLMAVMKMCVFIIRENDIRKCIECIEDDWKNVKYQE

DRKIMLENASF

SRRLIVICGAFMYGGVVFYYIALPFTRAKVVEEGGNLTYRRLVYPFPKALLDARRTPANEL

LYTIQLLSG

FVAHNITVAACGLAALLAMHACGQLQILMSWLEKLVDGRENDDENLDQRLVNIVEQHVR

IINFITLTEDL

LREISLVEVVGCTINICFLGYYSMMEWDTEHLIRGMTYIILLTSVTFNIFIFCYIGELLAEQT

VKVGEKF

YMIDWYRMPWKKSLAISLIISISRSTTKITAGNIIELSISSFGAIIKTSFAYLNILRTLTS

>AmelOR27

MMNQTAITEEIKTNSDYSLQLNRWFLKPIGAWPLFSTTTKFEKTVSLILNIICYAIVILCATPS

LMQIIL

AEESFYLKLKTLGPVSHWFVSTVNYTALLMKSKDIRYCFEHMEADWQTIKRMEDQQTML

KNAKFGRYVAA

SCAIFMQGGILCFCFVTILTTETIQVGNETRVLHVLPCAVYKKLVNVEENSINIFMLCFQFVA

AAIANSS

TVGIFSLAAVLAAHAYGQLSVVMVWITEFVNQSRNQKKTDDFKEIGIIVERHLRVLNFITY

LENIMNRIY

FLELFRCTMIICIVGYYILTEWAEKNVQNLTTYFMMLLSICFNIFIICYIGEILTEQCMKIGEV

VYMTDW

YYLPDKTILNLILIILRSTVVVQITAGKLFNMSIYTFGDVLKTAFAYLNLLRQMT

>AmelOR37

MMADDIATVQKEFENLNEYSIQFNKWFSKTIGVWPLPSSTSKFEKIMTRILILFCWIIALFD

AISGLLHF

VLVKEDIIIKLKSLAPISYIFGGGLNYAVLLLRKDDILYCIEHMETDWKTITRMTDRQIMLK

NAKIGRII

SCCILAFMQVSAVCFCTVLGVFKRTIKIGNESMEIYVLPSPIYKIPVDTNPGHDIVLGFQYLA

AYITSAT

VVSAFSFATVFACHASGQLTIMIIWIKEFINRPQKENKNRIDEISVIIEHHMRILSFLERAEHL

LSPICF

MEMFKNILSICLFSYCILAEWSEHNIRILGTYIFAVINITLNTFLICYIGEVLTERCKKIGNMV

YMTNWY

RLPKKDILNLIMIITRSSVEYKITAGKIIDMSVITFGNIIKTVFGYLNILRQTTML

>BmorOR4

MEQAKSEIDGSLVLSSFCMKRIGLSFESPKEFSSLLRQKIMFLLSFSGICYHVFSEIVYIGLTL

TNSPRV

EDVVPLFHTFGYGALSIAKVLSLWYKKDIFSQLLKELSEIWPTIPLDEDAQSIKTRSLSALH

VTQRWYFA

TNVLGVWFYNVTPIVKYMYRWLKEGDGQIGFVWVSWYPFDKNGTVAHIAVYIFEIFAGQ
TCVWIMVSTDL
LFSGMASHISMLLKLLQRRLQCFGTTPQTDDVNYQEIIAIIKLHQRLIRYCNDLEGVFSFSN
LVNVVLSS
MNICCVVFVIMLLEPLPALSSKLFLVSALIQIGMLCWYADDIFHANGDVATAAFNSGWYRT
SPKCRRSLL
FLIRRAQKPIAFTAMNFTNISLATYSAILTRSYSYFALLYTMYREN
>BmorOR47
MKLVFDNFIFALKVTLNWCRYFGIFIPDELTGRRQKLLVQAYSVFMFMLFIGFFIITQIILFIL
VWGDLS
LMTDVGLVLGTNLALSAKIAVFFFKREELASILKKNDDTLRFETREEGKKIISEIDRETNAF
MKVFFCFG
VGTVIAWFLSTPKGELHIATWYPCDTKRSPAYEIIMIHQLAITADLLMLSMIAVCRCRVKLV
GLYLQTIC
DDLPCNVKNKLTSDEEVIVAKRIREYVIEHQAILDCISELQNHFSPALLVQLLTSVVIICVTA
YQLAVEK
SSDLLRKFTMASFLFAMSTEMFTFGYQGGHLSHDSMEVATAAYSCPWYTFPTSLKRSLLVI
MIRAQQPAL
LTAGGFTTLSLETFVTIMKASYSFFTVLQEATD
>BmorOR45
MKVLDNVNHAVKVTMNCCRLYGLFVSDDLTKRQLIIMRAFSLMLYLFFVGFFITTQSALII
TMWGDLNLM
TNVGLVLGTHLTLSAKVFTLHYKEKEITNVIYKNEVRLRAETREQGKYIISEMNRETTLFM
RLFIPFGMG
TVTAWLLCTPKGELYTPAWYPCNTTKSPAHEIILAHQGIAVILTATLEIAIVLLMTSIVAVCRC
RLKLVG
LSFETICDDLPSNIMNKLTADEQVIVAKRVRENVIEHQAVLECINDIQDCFSSAMLVHIAIST
MIICATA
YQLAVEKSLDLTQRMTMASFLGGMSTEIFLFCYQGGHLSIDSMEVATAVYSCPWYTFPTSL
KRSLLVIMI
RAQQPALLTAGGFAPLLLDTFVSIMKASYSFFTVLQNASE
>BmorOR42
MDIPKFEELLKQIQMNFWLMGIPFDNPKIQIRYYVLLLTLSLMLIDEIAFFGSRMSSENFLEL
TQLAPCI
CIGVLSVLKILALTAKRQKIYELTQNLECLHKIILNDTRKTELVRKNLVLIKFITKYFFVLNA
VLIFVYN
FSSPVIIAYNYIVSNEVQFVLPYAVLLPFKTDSWIPWLIVYVYSIFCGFTCVLYYATVDVLYC
VMTSLVC
NNFSLISFKLQKVNRNTAHLLKEVVKEQQYVLKLAEDLENIFTAPNLFNVLIGSVEICALG
FNLMIGDLT
QIPGCILFLSSVLLQILIMSVFGENLISESSRIAEAAFLCKWYEMDQKSKKTILTIMIRSHKPK
KLTAYK
FSIISYGSFSKIISTSWSYFTILRTMYTPPGTKFQDDL
>BmorOR41

MMGNSTDLFLDRTKSILNFFAMWRSFEKPIPLKVYMAFIMTTQYLFLIFEIIYIVNVWGDM
AEVSEASIL
LFTQASVCYKITSFISKTNNFVILLGLIESEIFSAQTELHEKILILKARKIKRLCMFFLVNAVT
TCSLWA
VIPLLDISSKMLPFKIWMPASTGESPHYELGYLYQMITIYISAFLFIGVDSVPLSMIMFGCAQ
LEIIMDK
IGKVKSRPLDQQPMQRQAVLNSNYELLVECVRRYQSVVRFIELTEKTYHANIFFQLSGSVL
IICNIGFRI
AIVDSNSLQFYSMLTYLVTMLSQLFQYCWCGHELTIRGEELRETLYQSPWHEQDIRFRKVL
IITMERMKR
PIIFKAGHYIPLSRPTFVAILRCSYSYFAVLNRVRNE
>BmorOR38
MNLSQSVNEQANEYVKMRLERISKIHSPMLPFEDIQDFRELCCIPLAVYAVTGSITASYVYA
FLISLLWF
LFARCTDPEDFQVAMVVFSLGISSEIGSTKFFNSIIYIKELRKLFKDYLLYDATCPAQGRLRL
HLLTTLR
YVKRRAIIYWLVIIGNGFIFAIKPLLVEGRHLAQDDLVLIGLEPMRQSPNYEIAYAIMTMGV
CFICYPPA
HVTMFLIIIVGYTEAQMLALSEELKHLWNDAIEHYEKHSRTEREADAAMKSKILNSFVNF
RLVQIIKSHS
TNVNLIGRVENVFRGSLAVGYVFLIVGLIAELLGGLENTYLQVPFALIQVAIDCFIGQRVND
ANIDFEKA
VYDCKWENFDKRNMKIVLLLLQNAQKTVSLSAGGIAKLNFSCFMSVIKSIYSAYTTLRTT
MK
>BmorOR37
MELGCSRHLKLPCSLHPIGISKHGNTLSELLIYFPAIPKITYAILAVLLTVYYYIYLCSITWFV
FVRCPQ
TGDLAAASIVFSLGVSSEIGAIKLFIIAKLRDITGEYLQCEADMAPGRLRARVGRSLRTVRR
RAFVYWLV
LVVNAFAYDLMPAFLPGRHLSEDVFVIYGFEPMFESPNFEIASTLMGVSVVFICYTAGSISA
FLIVIVGY
SEATMLALSDEISCVWDDACASECQQPNDFIRARLGKIVAIHTKQIRLIREVEVVFRGALA
GGFACVAFG
LIAALLGGLENTFLQLPFCVIQISVDCFVGQRLRDANVAFETAVYNCKWEYFDKSNMKTV
LLILQNSQKT
MGLTAGGVAALDFTSLMTIFKSVYSGVHHSQTDD
>BmorOR35
MKLWQSIREFGLEYCDLPTTLQNVASLLRAITLNIDSRHTARIPFICYVMTVVITLSYFYVFL
VSMAWFV
FVRSAETRDYLAAMVVLSLGISSEIGTLKFFYTFIYIKKVQRIVREYLECDHMVVPESRFAD
NVLKTMRN
VKKRAILYWVVVIGNGVVYVTKPLFMSGRHHMEDRYIVYGLEPMFESPNYEVAYFLMMF
GLCFICYPPAN
VTVFLIVVVGYTEAQMIALGEEMLRIWEDAVAHYNNKYHTVGALTNSSEKNKIINQYVKF

RLTEIIKMHT
TNIQLLRQVEFVFRSAIAMGYVFLVLGLIAELLGGLENTYLQIPFALIQVLVDCYTGQKVM
DASSLFEQA
VYDCKWENFDKSNMKTVLLILQNSQKSMRLSVGGITVLGFSCMMSVMKSIYSAYATLRT
TMS
>BmorOR34
MIYYRKSKMELNFDKIFRIAIISQKFSGTYPYTKRDKKWATHFILMHGELTIICMLFIYNIIEF
DLKAAD
YSQMCRNMCLSFVYLVITLLYINMLYYQSKLKMLIETMKAEYEIAKTMSEEEQNVILEYA
KKGRWLCRAW
AILTTCGMAQFFLKSIIVCTIYSAIQGNFRIVQYYEVIYPEVIERHRNNPVIFITMYFCTFFYS
LYTSAL
YTSVLPLGPIFLLHGCAKLEIVRLNIKNLFDNDDYVVQERLKKTVLQMQEIYCYSNEINEC
FQVIYEFLL
KSSSLVLPITIFAVIQALGRGQFIPEFFAFIFGAFVVGTTPCYYSNMLMEKSEDVCMTLYSCG
WETRFDL
NTRKCIILMLCRALRPVSIRTIFRSVSLTTLTGVFQQAYALFNLLNAVWN
>BmorOR33
MIYYRKCKMELNFDKIFKIAIISQKFSGTYPYTKRDKKWATHFILMHGELTIICMLFIYNIIE
FDLKAAD
YSQMCRNMCLSFVYMVITLLYINMLYYQSKLKMLIETMKAEYELAKTMSEEEQNVILEY
AKKGRWLCRAW
AILTTCGMAQFFLKSIVCTIYSAIQGNFRIVQYYEVICPEVIERHRNNPVIFITLYFCTFFYSL
YTSALY
TSVLPLGPIFLLHGCAKLEIVRLNIKNLFDNDDYVVQERLKKTVLQMQDIYCYSHEINECF
QILYEFLLK
ATSLVLPITIFAVIQALGRGQFIPEFFAFIFGAFMVGTTPCYYSNMLMEKSEDVRMTLYSCG
WETRFDLN
TRKCIILMLCRALRPVSIRTIFRSVSLTTLTDVFQQAYALFNLLNAVWN
>BmorOR30
MSVSNLKFEVLFKPTTMSLHMNRSHPSIKRNKIWLLQFISLMTLTVFCATGLITSLLFHDLK
FGKYMEAS
KNGTIAMLSFTTTFKYSLLLYLQKSLNRLIAKIDMDYEIAKGLTPQEKAIVLNYAKKGVIVS
KFWLFTAF
AITFCFPLKAFIIMGYRFFIKNEFRLEPMFDMTYPEPIESYKTSFPVYFILFVVFFLFGCYASS
LFVAFD
PLVPIFVLHACGQLDLLSLRITKLFSDTKNPRIIAKELKVIISKLQELYGFVNFIKVNFSILYE
YNMKIT
TISMPLSAFQVVESLRRGEFNIEFTYFFFGCILHFFMPCYYSNLLMERSENFRFAIYSCGWE
NHNDKNIR
QMLLFMLTRATEPLGIATVFTNISLDTFAEMCRQSYTIFNLMNAAWA
>BmorOR25
MFEKALRSANFYMRVIGIPTDIRDGNRTLMERLRNRWFYCINFLWLNTDVAGEITWFVKG
LLNGSSTLIE

NTYLIPCLTLCILGNVKTFFTIKYANHIIDLVAILKDLEIKNNAARKNETEIVKERLKFLTTSN
KFLLFV
IGTGIIAFGIGPLMLTASIYFSSGDMKLKLPFLIWYPFDSSDIRYWPFVYVHQVWSACIACCA
VYGPDCF
YFTSCTFIHIHFIHLQNDITNVIVESSRARKNGLYRGCHQAFLELTNRHKDLIRCVNLLEIIY
SKSTLVN
VVSSSLLICVTGFNVMVTFCWFAAPFASFLALGLVQTYLLCYYGDTIMCSSTEVSDAVYNS
TWYGTNISQ
MRDYLFVMKRAQKPCKLTAYGFSDVNLRTFSRILSTAWSYFALLITIYRGNGQQ
>BmorOR21
MNKNMNKNHYILKTYCDKIFLVGSGNFWYQKTESRNDKTLLYKIYSCVLFFTYGFMTVL
EIMAAMMGDFP
EDEKRDSVTFATSHTVVMIKFISIIKNKELLKTLNRKMMMICEAHEEQTLMDEMYRTVKIN
VVAYCVAVY
GSATFYVFEGLRKFYNGSHFVTIVTYYPSNDDDTLAATIVRIATTLVLLMMLLTMIISVDTY
TMAYLIMY
KYKFITLRHYFKRLRENVDELVAAGKARLAAEKLAQGLVEGIKMHNELLSLSKDIDKAFG
TVMALQLCQS
SGSAVSLLLQIAVTMYLLLALFLCNAGEITYQASLLSDEIFYCGWHKCNSPVLSTQRNIRDI
VLIAILRA
QSPLVMKAFKMVVRSTYSVFALFYAQNK
>BmorOR19
MHEFVINVQNETTKLYDQLNIILYILGLQGIWVDEIKLSRRFHVFFKVVTFILHIMCGMFAG
LQFFAIFT
QNSLNSQQKSDVIVIGISNPMAYIFCINFIRNRNEIKDLFYHLAVVLKIYYNDVEIEKSMVN
KIKSYLST
YVFASITILVSNGIIAFFQTINSDEPFLGIITAWPDKTDTSKTASYARIGFYLFWCIHFFRISTVF
AVIV
CILISIKYQYKFLCSYFESLNKIFDDETSSHEVKEAEFENAFCNGIKIHTQIIWCVRRCQIMC
RTVFSAN
IMLDTFVLVILMLAMVNSENDFYGLCSQMSSVLVTVVLMAFFMWTAGDINVQASQLPDA
IYGSGWYNCRG
KSSARIRSLVTISMNKAQQPILMWALGFVELSHKNFVAIIKSAYSVFSVFY
>BmorOR16
MPVSPERSPHYHLGYSFQLVTICMSAYMYFGVDSVAFSSVIFGCAQIGVIKDKIMSIKPLGI
YRNHKTYT
NISRYNRKTLIECVKHHQAVISFTELVEDTYNSYLLFQLVGSVGIICGLAQCPITIPIAILCYL
SVMISQ
LFVCCWCGHELSATSEELHTILYNCAWYDQDVKFKRDLNFMMARARRPILLRAGYYISLS
RQSFVSILRM
SYSYFAVLNQTNK
>BmorOR15
MMTLVYQTDIFKPNVFFWKMFGIWADRKSSKTYKYYSFVFLFITLIMYNSLLAINLLYTPL
KIELLIREV

IFCFTEITVTTKVLMILFKRNKILDAFDLLNKNEFRGNSEESSAIIQKNNSAYKTYWKLYAIL
SNFAYSS
QVLGPLIVKLIWKTKLELPICNYYFLNEELRHDFFSGWYIYQSFGMYGHMMYNVNIDTFI
SGLLMMAVTQ
LKIIQTKLLSLKLNPRERKMDRGLMNITEVLKLNEILKHYELVLKYCSTVQSILDVAMFVQ
FGVASAIIC
VAMCGLIMVRSSTETLLFMVTYLFAMTLQIFVPAWMGTQLHFQSQELVFAAYNSEWIPRC
QSFKRSIIIF
VERAKIPITITGLKMFPLSLATFTSIMKTAYSFFTLIRNMQTLQEE
>BmorOR12
MTRITDVFSLNFIFWKFLGLWGKSAPSKYNMAYTVFYLFASLFVYDIFLTLNLIHTPRKLET
LVRETMFY
FNHLVAVTKILMMFIMRKKILVIFDLLDCEEFKPNDENSQEIMKRKTDFYYIYWRIVAVTSN
LSCFMLVI
GPLIKMLIWKIELGLPVCKFYFMSDELRNKYFVIWYIYQSFGIYNQMVNNLNLDTFNCGM
LWMAVGQLQI
LKTKFVNLKLNDFENGLDLKSRDDMQIERLRKYLTHYEIILKYCATVQDILNITIFVQLGM
SSIVICVGL
CGFVAMPSNTETAIFMSSYLITMTMQIFVPSWMGTQISFECGELMSAAYCCEWIPRSKLFK
RSLILFVER
AKTPVRITGLKIFTLSLDTFTSIMKTTYSFFTLIRQLQVDEVN
>BmorOR10
MRTNAKSFLFVPSKVLTLCGVWPVEKTSIFSLIYRSIMLSSQFCFLVFNGIYIGLMWGDLKA
VSDALYMF
FTQTTCCSKAIGFYFNFMKIKRIVASMDDVLFTAMSIEDQATIFSHSRTVNKLYKGVLGFTG
FTLVQWTV
LSLIGSGRTLPFNEMWVPTDISKSPNYEITFVVELWMMVISAALFMSVDTITVATMMFSCA
QLDIIMKKT
QQIQEIPLSPDLSSRNRSELHEKNNGILIDCIKQHQAIVRFSELCEGTFQVHSFFHLGGIVFMI
CVIGFR
MAGESPVSAQFWAALSYLVIILGQLYLYCWCANELTTKSEQLRDKLYLTPWYDQDVKFKR
NLCIAMECMA
KALTFRAGSYIPLSRAMFVSILRSSYSYFAFLNQANEQ

>CbowOR40
MYEKEFRNVFWLLNFVGMHPLKKYVTPFIVFNAILTFYITVLIILKLLWDKELVAVESLCVF
SQIWLKIF
VLTTKKRKIKQVIDDTQQFWENDPENSENKQLLKNLAKLERIFLTYISCSTCMFLFKPLLV
KGTSIYYYY
KIPQIPFYVSYPIEFYVTIVTMALAIAVNLFISIVIVIGAGQFSNLNAKMKQLDLSIAEDCQD
GLRTCTL
EMNKNIEYHDFLIKYVSHLDEIFSMLFVVLTGIITSLLCMNMYVLSQPTTTAVDMIRCGTM
VCAFTSEFL
FLYGVPAQRLMDEAEEVANSAFYHCQWYLPNIIPLRKSLSFMIHRSQKSVCLSAMGFIDIN

RQTIVAMIK
TAYSFFTFLQTIETTGGAK
>CbowOR39
MHNVLVTLVLCCLLYSSTAPLESFVLSVIVTLTLVVLIEVFILCWFCQEFTTEFSNVLDAAYE
LDWLNYS
PKLRRTIILTIMRLQKPPFFTMAKSTRLDMIFFSNLLKMAYSFYTLISKVN
>CbowOR38
MFAGCIFTCTFWAICPFTEDVASLPIAAWIPFKTDSSPSFELAFAYEIIATVIGGITDLNADCF
MAGFIM
VVCAQMKILNDSLLNLRHFAVEELNAETGGNNDDDGIAEELQKIMNRKLVECVHHHRYIL
EFAEEANSLF
TTSILGQFAVSVIIICTTLFEMTLVPFASIKFISLILYQYCMLMEIFIVCYFGNEVILESSKLTKY
AYHS
DWRDCSQEFKRNLLFFMTRSQRALKLYAGGFFTLSLDTYVKILKSSWSYFVVLIQVNKDS
G
>CbowOR37
MYFNTIQKVLPILYIIGADPREGFTKSQFFLYFYNIFSAIGMVYLLVLKFANAENKVTVKDIT
DAVICLF
LFCHGMVKSTTMFVKKNSVQTLLAQMEKHFWPMNNYKYSYIHNGILNICKTIRNTTNFI
WFMHFCNAMGF
LVGPLITKDPVLPFECYRPEWMGYYTLLLFEDVTSIITILCPVLAMDVFFVTIIKLTQIQWK
MLNSEIQS
MFDLSPSGKISREDEENIMVMKIKKCVVHHNFLLNYQQLLNDTFSIPLFFFLIVIVLCMCVE
MYVISTVS
DWESLRTAIVYTATGCLEFMLCYCYPCQDLSDEADNISYSIYFSNWYRNPEYFRDTQLIMQ
KGQKLVAIR
PGGFMIMDLKTGLSVGIFPSP
>CbowOR36
MYFFYVSFLYGTGVIFFVCEFMIFNETIGKISKFVSHIGMLFTHVVGILKMSILIFGRYRILKI
MNVLQN
EKYHYAPLEDSQPGLLVVKEKFVSSGISILVFVLYTFVGVSAHISSLITINEEIKGDSFEGTNK
TCHDYM
PYFFYIPFPTETKGQCGIAFAFMDVGLGIFAWVIACHDGVFVGLLNCLKTQLLIVCNIFTTIR
ARSLKAV
NLPKNYKILHDEYNPALEKELYRQLSHCTEHLKLLLVVRDDLEIMFTFVTLSQTLASLLIFA
SCLYVAST
VPMTSPEFFAQMEYFLCVLVQLSLICWFGNEITRASELIRLSLYESDWLSCSRRFKSSMILT
MIRMQRPV
YLSIGKFSPLTLATLVAVCRGSFSYFALFKSVQ
>CbowOR35
MSHNEIVCMTTSIKILRTYGVFPSQSRELHPGMVFYIRFLVLAVVTSLTLVGSTLHLIKTIQN
NEYNYTE
MDLVYIVSFVTAYALIGSFVMKVKASGEMFVFLSNFEEFGKPINFDKNNKLFNRYSKYHY
VYLESLILII

LFSSNIFKSKTCRLENELYNLKEVCGLFTYTWMPFNIDYTPVREIYLTIQLLGNHHIYMLAG
LVAWQVFE
TIQHIIIRIRHVKHLFVEALQEGDVKVRRKKFNFAVRYHNAVLSAFFSLEDKLNAAFGIFMV
THMVLTAA
VIGTGIYCLFRRRSLSSFLVCMGWFWGLFMDCFSGQRLQDESLELAVALYDSPWYEMDKE
FIKDIMFVLS
RCQIPMKLRAYAFGVIDRAMFLAVMKGTYSYITLLRSQ
>CbowOR34
MTFFFKIKGLSPPKTTAVKIIYLSLALPHILMCTFVLILSEWMAFALSQQTFKERMFNMSVA
TLDTILVF
RTTVWAFNKAKLDEIRAIITRKSFNFRCFDLLKVGCEQVLTVGRTKEVEKERGLSCKEIKQ
LWQKAKFVT
EKKCNEMDVFRKELMLNTRLLCCFIHLAITIISVLTYTLSFVNDFTNDTYEAYNPILNRTSL
YRKFQYPL
YLPFDTSFDGYYWLAYFYNCYAHLGNIITFLPIETTLTCSLIHLISQTAVLKEAFKYVDENNF
GYQSFGE
ASIIKEIRIVKCINEIQEIYRAVELLENLCNVQLMVQYGFATFLLCSICYVIPLVENTMEGICC
LIFFAA
SLGQIFTFSYCCHTLALELQAIGVSVYNLDWTNYPSKLKRTLNISILRTQKPANLTAGKIIVI
DLLFFIQ
VVQKSYSFYTLITKTN
>CbowOR33
MIAGFTTFIGLQCDILCDGIINMGKDRENELRVEEFIEHHKLILRFASTAGKVFSEIYFLHFIS
STSTLC
MTLFLLTLVDVNTSEFYYLVVYQSSVFSLLLVPCWFSSEMQRKSENLPNAIYSSPWIDASIS
LKKDIAYF
ICKTQEPIKFKALGVFLISVDTFMAVVRSSFSYYAVLNNLNVKGE
>CbowOR32
MEEDQKIYHFKTLKYILILMGQWKFRNRSRFFIKLYELIISRLVVAYLILSAHMFLINVAFAW
DCKARVM
EMLTAYMQNLNVIIVTLIMRSQRMRNVLRYVQHYENIKLKDENIAVRDIYMQYVSINHKIC
RLLIGVVVF
TALFYFATGMRNSFLISSTEECPMMKGIMFQLWYPMDTKKYFYLVVLNDNLIIINIVITIIHA
KGLIIAN
MIFAISQMKILQYELTLVGQIEQETDEDVELRVKKCIMIHQEIARMMDLFVSASKDIILMQY
FITSSELA
LYLLQMMLADSFAVFGRLLMNFIYLFVEVFLLFWCANNVLVESMAISDVIYNESNWITLS
NGAKKDMLMM
LSRAQVPMAFKATFVGNISLETFTKLLKLCYSVVAFLSNVVRE
>CbowOR31
MMAFGYPKNFFHMNEATVRFLGVWLPSKKHHILIRLLHPFYFIFVYSTLIYFVIGQYMKVE
MKNVTTIIS
SLAVLLTTHAGHVKGSLVVFGGRRIQEIKDILQDVNYQYYPVGEVNPGSTFQKEKTFYTM
LSYALLVGFM

MPGASGTVTTASRLMIEMKGNNTFESIDKNCNDFITYTFYVPIFIETKWECIISSSLMYSGM
TMYEGIAH
AAHDGLLAGLLICLKTQLLILGDIFRTLRQRVLSRLNIPEDYSVIHDEENPALEEEMYRQLC
LCTEHLKI
LLTARDKIEKTFTYMLLMQTIASFPVFASSLYAASQTPLSSTDFYTNIDFFGCVLVQLAMFC
WFGNGITE
AGEAIRSALYEGDWYSCSPRFKKSMILTMTRMQRPVYLSIGRFSPLTITTLVSVCQGSFSYF
TLFKSL
>CbowOR29
MTCNEFASFTFYIPFFTATKWQCIISSTAMYSGMLFEAGIHAVHDGIFFGLIYCSKIQLQIIGD
VFRTIR
QRSLSKLNIPEDYSVLHDEENPALEEELYRQLSHSTDHLKILLRVQDEIKKLFTYVLLAQIIS
MLFTLAS
FLYLVSTISINDPGLFLQFFWFMTILLQLTLFCWSGNGITEGFDSINTALYESEWLSCSRRFK
KSMILTM
TRLQRPVLLSLGGFSPLTLATLVGVCQGSFSYFTLFQSYQ
>CbowOR28
MYASNLDWCLKLNFLLGVHPAKQKSFTQTLQYLFIIFGSCAIMILTVLLLYYKEDAVSMKD
ITDVSTNFT
MFPHGMIKLTTLYMKRAEILDLLRRTKEHFWQIKDDREDVKRSYKLAKLLKNLFFNSVVL
FIISAIVKPI
IIGGNTLTYKCHKPELIPRWLFLIFQDAMCVAILFTLSCTDVLILTLLILTQIQFRMLNEKIQTI
HDNEV
DFHDRLKECVDHQNFLMDFVDRFSKVFSKTILLFIGNIILSLCMCMYIITTESANINVQMEA
LFHLIAGL
NEICLCYSIPAQTLMNEADEVGKNAYFSKWYEHPKDAKLILQIMIRDQKRMVITAGDFVRI
DMEMFLTAC
KTIVSYCMFLRTMSMVDQ
>CbowOR27
MIPSEDNSLSSMWLTKLILKSIFMWPDDYSDTKRKTFYKISMTICLFIQSGLVLNLTQNYHD
WEKNLAVV
SSMSTIFQTVFKMTALYQNSDHIKFVLMCMCRKFWPHNLDNENSEYIFKQSHSRRMRLM
VFLLASGFLFS
LGSVISPMFTRDTPFKSDYPFNWRRSPFYELIYLIQVAANGYLINMTVIGFDFLFMDICAAL
TNQYVLLG
SCFERLGTENMQDFYARIRERGCQKWPPKVGGARRFLGICVQHHQLLTQITKVVGHIFNV
VAFLQLCSSV
VAICVSGFIATKDDVTTSQIATMGSYLIGHLIQLYIYCSVGNELLFQSSTLTNHIFGSNWYNL
DSTTTKK
DIIFIMKKAQIPAKLNAFKVFPLNFATFIAVVRLSFSSYTLLTSITNK
>CbowOR26
MKHKKLYNFYTIFCTSVWVTFILSQLVYMFSSFSNMDEMTSIIYVAGTVTIDLVKMLAIYS
NMDRIKPLL
NDLNNPLFQPKCKEHVELALAVKKFHSRLFYFCLYFGVQTYICFSAIPFILEENVTLTQGWF

PIDWTYSP
NYEIVYAFQNIVILWNTLIFLNLDTFTSGLLMQVGLQCDFLSTTFNKIDAFHVSSGVLIENG
EQMALSLK
DNHEFFNRVMTENLIVCVKHYRKIRRLATEIEDIHHISVFILFLGGAIIICADLFQLSIVQTGG
VEFVLF
VSFLMCMLMEQFMYCWFGNEIIFKSDNIFAASYNTPWLDCDLKFRKILLNFMTQSIDPIGL
KAGGLFTMS
IKAFVSVLKSAYSYFTLLQRIQEKECSELN
>CbowOR24
MMTEKDEKMPKKLEIVCMPTSIRIFRFYCAFPPSDKLLNPGKMFYIRFALIALFSSVVLVGS
TMHLIKNV
KDRTYNHIELDFTYIVSNLAGYGLLCSYFTKVNAAVQLYLILSDFEEFGKPINFDNTNKKF
NKYAKYQYC
YLESITVCILLGSNMFRGAQCRKDNAELDQHEVCGLFAYTWLPFDIDFFPVKQIYLACQLF
GIHYVYMMA
GLASWMVLESVEHIATRLRHVSHFFNEALKEAEQRKRREKFNFAVRYHVAVLDLESKLNQ
TFSVFMFTHM
VMSGIIMGYGVYSYMKGKNVSTILIATGWLIGLLMDCYSGQRIQDESTLVGTALYDADWS
DADDELKRDI
RFVMMRCQKPMIVQATSFGIMDHPLFLAVLKATYSYVTLLSQSDL
>CbowOR22
MSSFSESPENEEQPFSATTKMMRLLCVYPLGFEKWQMVRFYVNVVVVKLFSFFFCCVLCL
LHLVMTKIDG
AHKADLSEDVSMIMAGTGMLATNLLFAYKVKKWNSLMGKVADSPEMRNIQNFEAIKKR
CNRLARLFTMYC
VIGAGIYLLSGYYESLVCIRKNEENGSNEICRTLMPVWLPFRLSSAAELTLFALQAFAGINL
SLPGANMP
FLVWEITEMISLRISHLKKISESIVVEKNIKSRQERLKHWVMSHQQIIECISLLNEQVRLCFG
HISTIAA
LVLGCLANQAINSVHLGAMAELGGWMVGLFLLCSSGQKITDITESVAEAIYAMEWYSTD
VQTMRDIRFIL
MRSQKPLVLQAGPLGALNYPLYMMMVKASYTYLTLLANTI
>CbowOR21
MKTQFQKAFQKEKWILTSLGSYPQWEPEALWQHCRRFFCMTVSITYITLMCTGPLLENSI
MILCTVMGVV
KEMQLLFSKQQFREIEEYIGSMKPCKIPISRLLGSFRNSVVTVIIFLGLMPYTKRSLRMLPY
KSWLPYDV
SRAPVYYVTFVAEVLTIIMAAFTNTTIDVLYYCLIDICCAELDLLKMELMEIDMSESYEVV
QNKLKNVVI
YHQRIIRLVEVIQDVFSSVVFIQCMTSVLVICFLGFQIVYVDEIPSGKATIEVSFIGCMLLQIFS
YCWFG
QSIMMKSLEVADVCYNSNWYDADLRIRKMIFIIMERCKQPLELRAKIITINLQTLLAILRSS
YSYMAILR
TLYSD

>CbowOR20
MRDVEEAEKSLMVTGWFPFDTREYFAVAYLFQLQIAIIGGLFLVALDSLIISLIMVAPLRLKV
LANYFRH
FGDKKSMNSLLSLKNLISEHQGIIRYVEDLNASLKWLFLADFVVKSYNISIVLSNAVSIYEF
RNIVIIIY
YVLQTRGRNKSELAFSALFLCFLLSQLYCFYFHANEILLESTNLAENIFKSKWYEQNSQIK
RSLIIVMIR
SQKPLQITIGDLHAIENILFVKIVKAAYTFLLFQYLGL
>CbowOR19
MFRVGDAIAFQSTINYMTFFKVFTVQTDTRYVGTILRFWSFCLICLFNTFHLVYVKIENIDV
DTSEDLVV
ILGGMGILLICIFSASSSRRWTSFLHNLIDFEKYGKPDGIEHAIERGNYWAHFFGLYYIIGTVI
YGIVTY
MEAPSCHRLNNEKNLHLICDTFVAIWLPFDIPLKSIRLSVFFIQFVLITCNVNPAAMACFLT
WECTEILR
CHLRHLKKHFHKMVKEGDVRKRPDGIGYWIRYHNHILSLSYELKSLFKISVGHTSLISGLV
IGCTENQIL
KSKPLGASLFFLGWMAAMMLLCHAGEILMEETLSVADTLRDSQWYLADLETRRDMVFI
MLRSQKPVHLEA
MPLGVMNYALFVMILRTSYSFMTLLNQSS
>CbowOR17
MNIEHIKEIEVLENSLKFLRIFFLFPRKSEINDPKKNVYWKFILLSLSTAYFSIGAAIHLVVNV
RSGAFV
NVDKDVGTIISYYGALYFISRYLGNIKYIIILYKQFSDFKTYGLPNNFEKTNKLLNKFSKIYF
VYHMFIV
TGMTTSTLLTIGTCEEENLENNINDICGLVGPTWLPFEFDYFPLKQIVYGYQVYCSFVIFQL
AGHLSYTL
MESVEHLIIRFEHVGHTFVEALNEKNSYTRREKFYVAIQYHNDVIQMGKLLNSCFAPSLIV
HISLTGPVL
GVAGYRFLTEIPLDSTCLFFGWMFSTFIVCRGGQRLSEASLAVGDVIYRVNWYNLETDLQR
DLKMVMLRS
RKPVYLRAGPFGPMTYSTIVTILKTCYSYITLLKQTM
>CbowOR16
MDIVVLTLISLTAIQFKLLNKGVAKIFKGITNLEQADVVIRERIQKYKNHHAFLMTFRNELN
NLFSNAIL
AYMGVIITTQCTELYVLFSWNSIQEGIRAVLYASTMFFELYICYCLPAQDLIDEAEKLPISIYC
SKWYQY
PNHFKDVLLLLGQFQLNMLISAGGVAILDIQTGFAALKSMVSYFAFLRTVGASSEK
>CbowOR15
MKEVHFKNTVLNFYEFYNTDFKLLKFFGIWIPDSSNSKFHKIYFIVINFVFCAIFNLAQVSN
LLHEINNL
KNLAACGYVVAIACMANVRSYYFLKNREEFLYLIRSLNDSHFQPESEDQICSAKKSLRFYS
KVKMIVSIL
CTITVFISMSTPVFYKKNELNLPFASWYPFDVSSYPIYQIAYVHQCISVIYVTSINTYVDIIM

AGFNTFI
GIQCDLLCSRLYNISKDHSSEENETTLLDCIRHHKLIVRFANNTEILFNRIYLGQFIACTSAL
CMALFLL
TLHQESRFESSFLVFYLTAIFSLLFIPCWFSSEMQGKSENIPEAAYSCNWVTASKLFKKDLIF
FILRAQK
PLKFYAVGFFQISVETFVLIVRSSFSYYTVLNNMIMKEG
>CbowOR14
MATEFQKAFETEKWILSLFGFYPQWNPEPLWKHARRVFCIAVTLTYIISMCTGPFLENSVMI
LCTVMGLS
KMVQLLTSKRQFREIEHYISNMKPSIIRRSSLIGAFRVSVVTLVGFLGIMPLSMKNQRMLPY
KSWLPYSV
EGASPYYSTFIFEVISIVMAAFTNSTIDMMYYCLVDICCAELDVLKLNIIEIDMSDHVDIVE
DELKKIVI
HHHKIIRLVGIIQEIFSSVVFVQCMASVLVICFLGFQLIYVDKLPSVKALIELSFIACMLIQIFC
YCWFG
HNITMKSSEVGDICYHTKWFESDLMIRKIILIIMERCKKPVELRAKIFTLNLQTLLAILRSSY
SYMAILR
TLYTDE
>CbowOR13
MDYGYPKNFFEANDVVKRISGIMLLQGKEDNIFFKWYQIIYIIFVYSSTVVFTVGQYIMTK
NSVNKISNL
VSSLGVLLTTHVGHFKFWLLLSKKKELENLKNDIEGENYQYATIGNSNPGLLLTNEKKFCT
VCTYVYLAG
CYLIGIFGNITTVVRLNGALTGNNTFESINMTCNDFASFTFYIPFFTANKWQCIISSTVMYSG
MAFESGI
HAACDLLFFAMIHCLKIQLRIIADVFRTIRRRSLLKLNVPEDYTVLHDEENSALEEELYRQL
SHSTEHLN
ILLRVTQEIEHLFTYVLLAQTLSSLLIVASFLYLTSTISINDADFFLQMQFLLVILIQLALLCWS
GNEIT
EGFQLIKTALYESDWLSCSHRFKRSMILTMIRLQRPVLLTLGGFSPLTLATLVGVCQGSFSY
FTLFKSFQ
>CbowOR12
MTENLIVCIDHYEKIKGLSREIENIHHTSIFILFLGGGVIICSGLFQLTLVEIGGLEFFMLISFL
MCMLT
EQFIYCWFGNDIIYKSAQISNAAYNTPWTECDLRFKKILLQFLIQTKKPIQIKVGGLFAMSID
AFKSVVQ
SSYSYFTLLKRLQDMS
>CbowOR11
MGAVKVLFFYFRGDKLIKIMATLESTDLHYEKCQKRKFFPGSISTNYKKVGIKYTLLFFML
AHATLISSY
IPPTIAAIQSELDNPGKSLPDRLPYYSWMPFKFDTSTTYLIALGYQAIPMFSYAYSIVGMDT
LFMNIMNC
IGMNLEIIQGAFLSLRERAADKIAGPLMTQDGLHNSHELKTALNREMKKVCRHLQIIYRLC
EDLENVHTF

LTLAQTVATLFILCSCLYLVSTTPASSKQFLSEIVYMVAMGFQLILYCWFGNEVTLKADMIP
FFIWQSDW
ISADREFKHAMIFTMIRAKRQLHLTAGKFAPLTLTTFIAIIKASYSFYAVIKNTST
>CbowOR10
MMVKQTKNEAKRFFRYIGMLFTPTQALGCYVLLRIKQHGIEKVREELLDEQFHYKSCGSF
RPGKIFNDAK
SFCDKFVVITIILYSLVVASAHISAYVTLNLAFEGEYFPANITCYDFMPNYFVIPFPTPTKSSC
KNALTY
MDVSLNVYATLLASYDTTFCSVLICFKTKLQILSGAMRSMRERVTTEMNLPLNSSLELDDP
EVEAKLYEE
IKQCARHLESLLSVCKQIEDIFKYGTLMQIVNALVIISSCMFVLSITPQSDPDFFVMIHYIIAL
FVQLFT
VCYFGNEITEVADELNNSLYQSNWLSCSKRHKQCMIIMMSRMQKKIHVMIGKFSPLTMN
MFVAVVKGALS
YCAVFRAVDNAEI
>CbowOR9
MLTFSLIHITSLVQVSETLSFNLTQLAYLCKLLNFQIHSKRLLELEDFLRKTTLTNVTVEEEA
IIRNTMK
GSRRLATVYRSLCVIIVFLYALFPLIDENSGDEKKLPLPMWFPFDTNNHFGKVWFFEIFSIAI
GAWTNSN
LDVICVTMITLTTCQFNIMNSRLSNLRKSTDDVEEEDTVQKALKECVIHYNDIISFQILVETT
FSLSIFG
QFVFSVLVICMTGFQMLVISFKSVQFVLLLSYLLGQTCQIVMYCWYGQSILDSSEAINDAC
YSSEWFNCS
KETQKMFLIIMERSKRPVKMRAGKFFFLNLDTLMSILKSSYSYFAVLRHIYSSKFT
>CbowOR7
MISPTSYLIKLIIFKSKSVHVLEMLSFLELTEFNNYPKGLSGIVERTVKFSRYLGYAYQFMCC
LVITLYS
TIPLFTKADLPIRFSHDVGKLKPAVYIFQVIGLSSAASNNSCLDVLAMSLMGICSAQIDILNK
KLITLGK
NEDEDETDGSNNSYLRLKKCAKHHVEIIRFQKALERVFSSIFLAQFATSVMVICNIGFQLVH
VQPASVQF
ALMLFYFIAMNTQLVMYCWYGNEIIVKVYHCLSILSSAIRDACYKFEWFDSNMETKKLLL
IIMEHSKKHL
YLTAGKISVLSLESFTSVMRTSYSYFALLQTLYRNNQD
>CbowOR6
MYTIKKSQPFYSSLRTLRFFLVYREFVQKSTFMLLSFFSSFMSFSAFLVFVCGCILHAVMSIR
ENIGGDI
SEDLSVSIGGLAMMVNVAMFKYHQDKWSNFFKDVTNFEKFGKPTDFDATKDRANLLSTL
YMIYCITGTIV
YSCVGVIESSCDELSEETKQKVICGTLAPIWLPFEDVSLTVRNTILLVQYVLANYIITPSAVI
CFLPFET
TELLICHINFLKDKLLKVFGNEDGMIRNDKLRFCVAYHTHILGMADQLKYVVKFSVGHMS
LVCALVFGCI

GNQIFRAKPVGAVIFLLGYMVSLFLLCYAGQRIMNESLSIVDVIYNSKWYKGNTQIKKNV
RFMMARCQIP
VTLDAWPFGIFSFPLFMMIVKTSYSYLTLLRQST
>CbowOR5
MIKMIQIRFHTLGFLACMKIFMLSFGQLLSKTLPLKCYSAKWIPFHVMWFYQSFLTSVCII
MPIIAMDLL
LMTFISLTHIQFKMLNLEIDRVFRRSERAKKSEIARLVDHHNFLIDFSNRINNTFSTMLLAYI
FVFVISM
CVEMYKSSANPSFSVFMNAVTYLSAAVFGLIFLFCIPGQNLTDEANNIPNAVYFTDWYRDS
KQSTSVLMM
ISNGQRDISIKAGEVIKINLATSLSTIKTLLSYFMFLRTVVLDE
>CbowOR4
MLFYVCGFAVVICEYMMFKESIKDIGKFVSHIGMVLTHLAGIVKFCLLTIGHGKILKLMHV
LQNKDYQYC
SLEDSKPGEVLRKGQTVNNVIAYSTFVMYTLVGITGHISSVRNLNEQIKGDNFEGTNKTCY
DFLPYMFYI
PIPSETKWQCEMVFNLMDIGFALHAFVIAAHDGIFAGLLICLKSQLLIVCDVYKTIRQRSLK
NMHLPENY
TITNDMENPALENEMYRLLVHSMEHLKILLWVRDELEYIFTMVVLTQTVASLFILASNFYV
ASTILTASL
EFFAKLEYTFCIFFQLSLICWFGDDITRASDLIKLSLYESDWLSSSPRFKHAMVLTMIRMQR
PVFLSIGK
FTPITLSTLVAVCRGSFSYFALFKSIQK
>CbowOR3
MATITNSLFVILEIGICIIKFLPFKNDPKKIRKTLFALNQDMFNRATESQRRFIEETEAACRNIF
AIFMT
FCLLSLFSWPIKVLFYEQRRFPIDVWLPFDPFENVSIYLGVFAYLFIATGNAPIGNAAIDTLIA
GLIIHA
ACQFRILKDNLRCLSQRADEKLNGLPQELKEMKRNEIVYRNIRECILHYDAIYDFVKEVE
KTFSVVIFSQ
FAVSILVICISCFQLSIAEPLTITFFAMVIYVVSLLLEIFLYCYYGTVLYEESNTLIAAIFDSEW
YDLDE
KSKKALFILMERAKRPMMLTTGKLLSVSLETWTMIIRRSYSLLAVLKNHQ
>CbowORco
MMKFKVSGLVADLMPNIRLIQASGHFMFNYHADNSGALHALRLGYSCMHLVFCLFQFGC
TFGNLVVERDN
VNDLAANTITVLFFTHCITKFVYFAVRSKLFYRTLGIWNQANSHPLFVESNNRYHALALKK
MRTLLVCVM
ATTVLSASAWTGITFVGDSIHHIKDPDNENETIIEEIPRLLVKSWYPWDAMSGTAYYASLIF
QIYYVFFS
LAHANLMDSLFCSWLIFACEQLQHLKEIMKPLMELSASLDTYVPKSADLFRAPSANSQDN
LIENDYNAKN
EEINLKGIYNTRQELGINFRSGALQTFGQGGGGVGPNGLSKKQELMVRSAIKYWVERHK
HVVRLVTAIGD

AYGVALLLHMLTSTVMLTLLAYQATQIGGVNKYAATVIGYLVYSLAQVFHFCIFGNRLIEE
SSSVMEAAY
SCHWYDGSEEAKTFVQIVCQQCQKAMSISGAKFFTISLDLFASVLGAVVTYFMVLVQLK
>CbowOR1
MIGFLKRQTLVDKLKIHVTFFFILILDVLILVKISTTHNKTLEDIMTSYEAVGSYLQMTTKIL
TLIIYNG
DLKQILAMTNQFWKYDKFGPVISNKQQKYPRMMPSFITAYFFFCICTLTVLMLKPVLFHEL
PRSCCIPEG
EVWFYVVSAIQNETLFYCTFTVFAFDAMFALLYTEAAMQFCLLNEAFSRMKNHGDLKEC
VDYHVFLYNFV
KKLNDVYWMFLLVQSFDCLSETCFQLLTMVHTQENLTLRVKAVLYAIALYMQLSFFCFPV
GFLQDESQAS
STAISACPWYLKDAKFKRSVFIVMIRAQKKISVRAGGFFEMDRQAFIYLCKSSFSVYTLLK
SIN

>DmelOR10a
MSEWLRFLKRDQQLDVYFFAVPRLSLDIMGYWPGKTGDTWPWRSLIHFAILAIGVATELH
AGMCFLDRQQ
ITLALETLCPAGTSAVTLLKMFLMLRFRQDLSIMWNRLRGLLFDPNWERPEQRDIRLKHS
AMAARINFWP
LSAGFFTCTTYNLKPILIAMILYLQNRYEDFVWFTPFNMTMPKVLLNYPFFPLTYIFIAYTG
YVTIFMFG
GCDGFYFEFCAHLSALFEVLQAEIESMFRPYTDHLELSPVQLYILEQKMRSVIIRHNAIIDLT
RFFRDRY
TIITLAHFVSAAMVIGFSMVNLLTLGNNGLGAMLYVAYTVAALSQLLVYCYGGTLVAESST
GLCRAMFSC
PWQLFKPKQRRLVQLLILRSQRPVSMAVPFFSPSLATFAAILQTSGSIIALVKSFQ
>DmelOR2a
MEKQEDFKLNTHSAVYYHWRVWELTGLMRPPGVSSLLYVVYSITVNLVVTVLFPLSLLAR
LLFTTNMAGL
CENLTITITDIVANLKFANVYMVRKQLHEIRSLLRLMDARARLVGDPEEISALRKEVNIAQ
GTFRTFASI
FVFGTTLSCVRVVVRPDRELLYPAWFGVDWMHSTRNYVLINIYQLFGLIVQAIQNCASDS
YPPAFLCLLT
GHMRALELRVRRIGCRTEKSNKGQTYEAWREEVYQELIECIRDLARVHRLREIIQRVLSVP
CMAQFVCSA
AVQCTVAMHFLYVADDHDHTAMIISIVFFSAVTLEVFVICYFGDRMRTQSEALCDAFYDCN
WIEQLPKFK
RELLFTLARTQRPSLIYAGNYIALSLETFEQVMRFTYSVFTLLLRAK
>DmelOR7a
MAVSTRVATKQEVPESRRAFRNLFNCFYALGMQAPDGSRPTTSSTWQRIYACFSVVMYV
WQLLLVPTFFV
ISYRYMGGMEITQVLTSAQVAIDAVILPAKIVALAWNLPLLRRAEHHLAALDARCREQEEF
QLILDAVRF

CNYLVWFYQICYAIYSSSTFVCAFLLGQPPYALYLPGLDWQRSQMQFCIQAWIEFLIMNWT
CLHQASDDV
YAVIYLYVVRIQVQLLARRVEKLGTDDSGQVEIYPDERRQEEHCAELQRCIVDHQTMLQL
LDCISPVISR
TIFVQFLITAAIMGTTMINIFIFANTNTKIASIIYLLAVTLQTAPCCYQATSLMLDNERLALAI
FQCQWL
GQSARFRKMLLYYLHRAQQPITLTAMKLFPINLATYFSIAKFSFSLYTLIKGMNLGERFNRT
N
>DmelOR9a
MSDKVKGKKQEEKDQSLRVQILVYRCMGIDLWSPTMANDRPWLTFVTMGPLFLFMVPM
FLAAHEYITQVS
LLSDTLGSTFASMLTLVKFLLFCYHRKEFVGLIYHIRAILAKEIEVWPDAREIIEVENQSDQ
MLSLTYTR
CFGLAGIFAALKPFVGIILSSIRGDEIHLELPHNGVYPYDLQVVMFYVPTYLWNVMASYSA
VTMALCVDS
LLFFFTYNVCAIFKIAKHRMIHLPAVGGKEELEGLVQVLLLHQKGLQIADHIADKYRPLIFL
QFFLSALQ
ICFIGFQVADLFPNPQSLYFIAFVGSLLIALFIYSKCGENIKSASLDFGNGLYETNWTDFSPPT
KRALLI
AAMRAQRPCQMKGYFFEASMATFSTIVRSAVSYIMMLRSFNA
>DmelOR59a
MAEVRVDSLEFFKSHWTAWRYLGVAHFRVENWKNLYVFYSIVSNLLVTLCYPVHLGISLF
RNRTITEDIL
NLTTFATCTACSVKCLLYAYNIKDVLEMERLLRLLDERVVGPEQRSIYGQVRVQLRNVLYV
FIGIYMPCA
LFAELSFLFKEERGLMYPAWFPFDWLHSTRNYYIANAYQIVGISFQLLQNYVSDCFPAVVL
CLISSHIKM
LYNRFEEVGLDPARDAEKDLEACITDHKHILELFRRIEAFISLPMLIQFTVTALNVCIGLAAL
VFFVSEP
MARMYFIFYSLAMPLQIFPSCFFGTDNEYWFGRLHYAAFSCNWHTQNRSFKRKMMLFVE
QSLKKSTAVAG
GMMRIHLDTFFSTLKGAYSLFTIIIRMRK
>DmelOR74a
MSFHRYRPRLPGGELAPMPWPVSLYRVLNHVAWPLEAESGRWTVFLDRLMIFLGFLVFCE
HNEVDFHYLI
ANRQDMDNMLTGLPTYLILVEMQIRCFQLAWHKDRFRALLQRFYAEIYVSEEMEPHLFAS
IQRQMLATRV
NSTVYLLALLNFFLVPVTNVIYHRREMLYKQVYPFDNTQLHFFIPLLVLNFWVGFIITSMLF
GELNVMGE
LMMHLNARYIQLGQDLRRSAQMLLKKSSSLNVAIAYRLNLTHILRRNAALRDFGQRVEKE
FTLRIFVMFA
FSAGLLCALFFKAFTNPWGNVAYIVWFLAKFMELLALGMLGSILLKTTDELGMMYYTAD
WEQVIHQSDNV
GENVKLMKLVTLAIQLNSRPFFITGLNYFRVSLTAVLKIIQGAFSYFTFLNSMR

>DmelOR22b
MLSQFFPHIKEKPLSERVKSRDAFVYLDRVMWSFGWTVPENKRWDLHYKLWSTFVTLLIF
ILLPISVSVE
YIQRFKTFSAGEFLSSIQIGVNMYGSSFKSYLTMMGYKKRQEAKMSLDELDKRCVCDEER
TIVHRHVALG
NFCYIFYHIAYTSFLISNFLSFIMKRIHAWRMYFPYVDPEKQFYISSIAEVILRGWAVFMDLC
TDVCPLI
SMVIARCHITLLKQRLRNLRSEPGRTEDEYLKELADCVRDHRLILDYVDALRSVFSGTIFV
QFLLIGIVL
GLSMINIMFFSTLSTGVAVVLFMSCVSMQTFPFCYLCNMIMDDCQEMADSLFQSDWTSAD
RRYKSTLVYF
LHNLQQPIILTAGGVFPISMQTNLNMVKLAFTVVTIVKQFNLAEKFQ
>DmelOR33a
MDSRRKVRSENLYKTYWLYWRLLGVEGDYPFRRLVDFTITSFITILFPVHLILGMYKKPQI
QVFRSLHFT
SECLFCSYKFFCFRWKLKEIKTIEGLLQDLDSRVESEEERNYFNQNPSRVARMLSKSYLVAA
ISAIITAT
VAGLFSTGRNLMYLGWFPYDFQATAAIYWISFSYQAIGSSLLILENLANDSYPPITFCVVSG
HVRLLIMR
LSRIGHDVKLSSSENTRKLIEGIQDHRKLMKIIRLLRSTLHLSQLGQFLSSGINISITLINILFF
AENNF
AMLYYAVFFAAMLIELFPSCYYGILMTMEFDKLPYAIFSSNWLKMDKRYNRSLIILMQLTL
VPVNIKAGG
IVGIDMSAFFATVRMAYSFYTLALSFRV
>DmelOR85a
MIFKYIQEPVLGSLFRSRDSLIYLNRSIDQMGWRLPPRTKPYWWLYYIWTLVVIVLVFIFIP
YGLIMTGI
KEFKNFTTTDLFTYVQVPVNTNASIMKGIIVLFMRRRFSRAQKMMDAMDIRCTKMEEKV
QVHRAAALCNR
VVVIYHCIYFGYLSMALTGALVIGKTPFCLYNPLVNPDDHFYLATAIESVTMAGIILANLILD
VYPIIYV
VVLRIHMELLSERIKTLRTDVEKGDDQHYAELVECVKDHKLIVEYGNTLRPMISATMFIQL
LSVGLLLGL
AAVSMQFYNTVMERVVSGVYTIAILSQTFPFCYVCEQLSSDCESLTNTLFHSKWIGAERRY
RTTMLYFIH
NVQQSILFTAGGIFPICLNTNIKMAKFAFSVVTIVNEMDLAEKLRRE
>DmelOR94a
MDKHKDRIESMRLILQVMQLFGLWPWSLKSEEEWTFTGFVKRNYRFLLHLPITFTFIGLM
WLEAFISSNL
EQAGQVLYMSITEMALVVKILSIWHYRTEAWRLMYELQHAPDYQLHNQEEVDFWRREQ
RFFKWFFYIYIL
ISLGVVYSGCTGVLFLEGYELPFAYYVPFEWQNERRYWFAYGYDMAGMTLTCISNITLDT
LGCYFLFHIS
LLYRLLGLRLRETKNMKNDTIFGQQLRAIFIMHQRIRSLTLTCQRIVSPYILSQIILSALIICFS

GYRLQ
HVGIRDNPGQFISMLQFVSVMILQIYLPCYYGNEITVYANQLTNEVYHTNWLECRPPIRKL
LNAYMEHLK
KPVTIRAGNFFAVGLPIFVKTINNAYSFLALLLNVSN
>DmelOR49b
MFEDIQLIYMNIKILRFWALLYDKNLRRYVCIGLASFHIFTQIVYMMSTNEGLTGIIRNSYM
LVLWINTV
LRAYLLLADHDRYLALIQKLTEAYYDLLNLNDSYISEILDQVNKVGKLMARGNLFFGMLT
SMGFGLYPLS
SSERVLPFGSKIPGLNEYESPYYEMWYIFQMLITPMGCCMYIPYTSLIVGLIMFGIVRCKAL
QHRLRQVA
LKHPYGDRDPRELREEIIACIRYQQSIIEYMDHINELTTMMFLFELMAFSALLCALLFMLIIV
SGTSQLI
IVCMYINMILAQILALYWYANELREQNLAVATAAYETEWFTFDVPLRKNILFMMMRAQRP
AAILLGNIRP
ITLELFQNLLNTTYTFFTVLKRVYG
>DmelOR47a
MDSFLQVQKSTIALLGFDLFSENREMWKRPYRAMNVFSIAAIFPFILAAVLHNWKNVLLL
ADAMVALLIT
ILGLFKFSMILYLRRDFKRLIDKFRLLMSNEAEQGEEYAEILNAANKQDQRMCTLFRTCFL
LAWALNSVL
PLVRMGLSYWLAGHAEPELPFPCLFPWNIHIIRNYVLSFIWSAFASTGVVLPAVSLDTIFCSF
TSNLCAF
FKIAQYKVVRFKGGSLKESQATLNKVFALYQTSLDMCNDLNQCYQPIICAQFFISSLQLCM
LGYLFSITF
AQTEGVYYASFIATIIIQAYIYCYCGENLKTESASFEWAIYDSPWHESLGAGGASTSICRSLL
ISMMRAH
RGFRITGYFFEANMEAFSSIVRTAMSYITMLRSFS
>DmelOR45b
MYPRFLSRNYPLAKHLFFVTRYSFGLLGLRFGKEQSWLHLLWLVFNFVNLAHCCQAEFVF
GWSHLRTSPV
DAMDAFCPLACSFTTLFKLGWMWWRRQEVADLMDRIRLLIGEQEKREDSRRKVAQRSY
YLMVTRCGMLVF
TLGSITTGAFVLRSLWEMWVRRHQEFKFDMPFRMLFHDFAHRMPWFPVFYLYSTWSGQV
TVYAFAGTDGF
FFGFTLYMAFLLQALRYDIQDALKPIRDPSLRESKICCQRLADIVDRHNEIEKIVKEFSGIM
AAPTFVHF
VSASLVIATSVIDILLYSGYNIIRYVVYTFTVSSAIFLYCYGGTEMSTESLSLGEAAYSSAWY
TWDRETR
RRVFLIILRAQRPITVRVPFFAPSLPVFTSVIKFTGSIVALAKTIL
>DmelOR19b
MDISKVDSTRALVNHWRIFRIMGIHPPGKRTFWGRHYTAYSMVWNVTFHICIWVSFSVNL
LQSNSLETFC
ESLCVTMPHTLYMLKLINVRRMRGEMISSHWLLRLLDKRLGCADERQIIMAGIERAEFIFR

TIFRGLACT
VVLGIIYISASSEPTLMYPTWIPWNWKDSTSAYLATAMLHTTALMANATLVLNLSSYPGTY
LILVSVHTK
ALALRVSKLGYGAPLPAVRMQAILVGYIHDHQIILRLFKSLERSLSMTCFLQFFSTACAQCT
ICYFLLFG
NVGIMRFMNMLFLLVILTTETLLLCYTAELPCKEGESLLTAVYSCNWLSQSVNFRRLLLLM
LARCQIPMI
LVSGVIVPISMKTFTVMIKGAYTMLTLLNEIRKTSLE
>DmelOR1a
MSKLIEVFLGNLWTQRFTFARMGLDLQPDKKGNVLRSPLLYCIMCLTTSFELCTVCAFMV
QNRNQIVLCS
EALMHGLQMVSSLLKMAIFLAKSHDLVDLIQQIQSPFTEEDLVGTEWRSQNQRGQLMAAI
YFMMCAGTSV
SFLLMPVALTMLKYHSTGEFAPVSSFRVLLPYDVTQPHVYAMDCCLMVFVLSFFCCSTTG
VDTLYGWCAL
GVSLQYRRLGQQLKRIPSCFNPSRSDFGLSGIFVEHARLLKIVQHFNYSFMEIAFVEVVIIC
GLYCSVIC
QYIMPHTNQNFAFLGFFSLVVTTQLCIYLFGAEQVRLEAERFSRLLYEVIPWQNLPPKHRK
LFLFPIERA
QRETVLGAYFFELGRPLLVWIFRTAGSFTTLMNALYAKYETH
>DmelOR30a
MELKSMDPVEMPIFGSTLKLMKFWSYLFVHNWRRYVAMTPYIIINCTQYVDIYLSTESLD
FIIRNVYLAV
LFTNTVVRGVLLCVQRFSYERFINILKSFYIELLQSDDPIINILVKETTRLSVLISRINLLMGC
CTCIGF
VTYPIFGSERVLPYGMYLPTIDEYKYASPYYEIFFVIQAIMAPMGCCMYIPYTNMVVTFTLF
AILMCRVL
QHKLRSLEKLKNEQVRGEIIWCIKYQLKLSGFVDSMNALNTHLHLVEFLCFGAMLCVLLF
SLIIAQTIAQ
TVIVIAYMVMIFANSVVLYYVANELYFQSFDIAIAAYESNWMDFDVDTQKTLKFLIMRSQK
PLAILVGGT
YPMNLKMLQSLLNAIYSFFTLLRRVYG
>DmelOR67c
METAKDNTARTFMELMRVPVQFYRTIGEDIYAHRSTNPLKSLLFKIYLYAGFINFNLLVIGE
LVFFYNSI
QDFETIRLAIAVAPCIGFSLVADFKQAAMIRGKKTLIMLLDDLENMHPKTLAKQMEYKLPD
FEKTMKRVI
NIFTFLCLAYTTTFSFYPAIKASVKFNFLGYDTFDRNFGFLIWFPFDATRNNLIYWIMYWDI
AHGAYLAG
IAFLCADLLLVVVITQICMHFNYISMRLEDHPCNSNEDKENIEFLIGIIRYHDKCLKLCEHV
NDLYSFSL
LLNFLMASMQICFIAFQVTESTVEVIIIYCIFLMTSMVQVFMVCYYGDTLIAASLKVGDAA
YNQKWFQCS
KSYCTMLKLLIMRSQKPASIRPPTFPPISLVTYMKVISMSYQFFALLRTTYSNN

>DmelOR83c
MSTSESPSSRFRELSKYINSLTNLLGVDFLSPKLKFNYRTWTTIFAIANYTGFTVFTILNNGG
DWRVGLK
ASLMTGGLFHGLGKFLTCLLKHQDMRRLVLYSQSIYDEYETRGDSYHRTLNSNIDRLLGI
MKIIRNGYVF
AFCLMELLPLAMLMYDGTRVTAMQYLIPGLPLENNYCYVVTYMIQTVTMLVQGVGFYS
GDLFVFLGLTQI
LTFADMLQVKVKELNDALEQKAEYRALVRVGASIDGAENRQRLLLDVIRWHQLFTDYCR
AINALYYELIA
TQVLSMALAMMLSFCINLSSFHMPSAIFFVVSAYSMSIYCILGTILEFAYDQVYESICNVTW
YELSGEQR
KLFGFLLRESQYPHNIQILGVMSLSVRTALQIVKLIYSVSMMMMNRA
>DmelOR88a
MKPTEIKKPYRMEEFLRPQMFQEVAQMVHFQWRRNPVDNSMVNASMVPFCLSAFLNVL
FFGCNGWDIIGH
FWLGHPANQNPPVLSITIYFSIRGLMLYLKRKEIVEFVNDLDRECPRDLVSQLDMQMDETY
RNFWQRYRF
IRIYSHLGGPMFCVVPLALFLLTHEGKDTPVAQHEQLLGGWLPCGVRKDPNFYLLVWSFD
LMCTTCGVSF
FVTFDNLFNVMQGHLVMHLGHLARQFSAIDPRQSLTDEKRFFVDLRLLVQRQQLLNGLC
RKYNDIFKVAF
LVSNFVGAGSLCFYLFMLSETSDVLIIAQYILPTLVLVGFTFEICLRGTQLEKASEGLESSLR
SQEWYLG
SRRYRKFYLLWTQYCQRTQQLGAFGLIQVNMVHFTEIMQLAYRLFTFLKSH
>DmelOR92a
MLFRKRKPKSDDEVITFDELTRFPMTFYKTIGEDLYSDRDPNVIRRYLLRFYLVLGFLNFNA
YVVGEIAY
FIVHIMSTTTLLEATAVAPCIGFSFMADFKQFGLTVNRKRLVRLLDDLKEIFPLDLEAQRKY
NVSFYRKH
MNRVMTLFTILCMTYTSSFSFYPAIKSTIKYYLMGSEIFERNYGFHILFPYDAETDLTVYWF
SYWGLAHC
AYVAGVSYVCVDLLLIATITQLTMHFNFIANDLEAYEGGDHTDEENIKYLHNLVVYHARA
LDLSEEVNNI
FSFLILWNFIAASLVICFAGFQITASNVEDIVLYFIFFSASLVQVFVVCYYGDEMISSSSRIGHS
AFNQN
WLPCSTKYKRILQFIIARSQKPASIRPPTFPPISFNTFMKVISMSYQFFALLRTTYYG
>DmelOR65b
MDIQRFLKFYKVGWKTYRDPLMEASHSSIYYWREQMKAMALFTTTEERLLPYRSKWHT
LVYIQMVIFFAS
MSFGLTESMGDHVQMGRDLAFILGAFFIIFKTYYFCWYGDELDQVISDLDALHPWAQKGP
NPVEYQTGKR
WYFVMAFFLATSWSFFLCILLLLLITSPMWVHQQNLPFHAAFPFQWHEKSLHPISHAIIYLF
QSYFAVYC
LTWLLCIEGLSICIYAEITFGIEVLCLELRQIHRHNYGLQELRMETNRLVKLHQKIVEILDRT

NDVFHGT
LIMQMGVNFSLVSLSVLEAVEARKDPKVVAQFAVLMLLALGHLSMWSYCGDQLSQKSLQ
ISEAAYEAYDP
TKGSKDVYRDLCVIIRRGQDPLIMRASPFPSFNLINYSAILNQCYGILTFLLKTLD
>DmelOR42a
MDLRRWFPTLYTQSKDSPVRSRDATLYLLRCVFLMGVRKPPAKFFVAYVLWSFALNFCST
FYQPIGFLTG
YISHLSEFSPGEFLTSLQVAFNAWSCSTKVLIVWALVKRFDEANNLLDEMDRRITDPGERL
QIHRAVSLS
NRIFFFFMAVYMVYATNTFLSAIFIGRPPYQNYYPFLDWRSSTLHLALQAGLEYFAMAGAC
FQDVCVDCY
PVNFVLVLRAHMSIFAERLRRLGTYPYESQEQKYERLVQCIQDHKVILRFVDCLRPVISGTI
FVQFLVVG
LVLGFTLINIVLFANLGSAIAALSFMAAVLLETTPFCILCNYLTEDCYKLADALFQSNWIDE
EKRYQKTL
MYFLQKLQQPITFMAMNVFPISVGTNISVTKFSFSVFTLVKQMNISEKLAKSEMEE
>DmelOR43b
MFGHFKLVYPAPISEPIQSRDSNAYMMETLRNSGLNLKNDFGIGRKIWRVFSFTYNMVILP
VSFPINYVI
HLAEFPPELLLQSLQLCLNTWCFALKFFTLIVYTHRLELANKHFDELDKYCVKPAEKRKV
RDMVATITRL
YLTFVVVYVLYATSTLLDGLLHHRVPYNTYYPFINWRVDRTQMYIQSFLEYFTVGYAIYVA
TATDSYPVI
YVAALRTHILLLKDRIIYLGDPSNEGSSDPSYMFKSLVDCIKAHRTMLNFCDAIQPIISGTIFA
QFIICG
SILGIIMINMVLFADQSTRFGIVIYVMAVLLQTFPLCFYCNAIVDDCKELAHALFHSAWWV
QDKRYQRTV
IQFLQKLQQPMTFTAMNIFNINLATNINVAKFAFTVYAIASGMNLDQKLSIKE
>DmelOR35a
MVRYVPRFADGQKVKLAWPLAVFRLNHIFWPLDPSTGKWGRYLDKVLAVAMSLVFMQH
NDAELRYLRFEA
SNRNLDAFLTGMPTYLILVEAQFRSLHILLHFEKLQKFLEIFYANIYIDPRKEPEMFRKVDG
KMIINRLV
SAMYGAVISLYLIAPVFSIINQSKDFLYSMIFPFDSDPLYIFVPLLLTNVWVGIVIDTMMFGE
TNLLCEL
IVHLNGSYMLLKRDLQLAIEKILVARDRPHMAKQLKVLITKTLRKNVALNQFGQQLEAQY
TVRVFIMFAF
AAGLLCALSFKAYTNPMANYIYAIWFGAKTVELLSLGQIGSDLAFTTDSLSTMYYLTHWE
QILQYSTNPS
ENLRLLKLINLAIEMNSKPFYVTGLKYFRVSLQAGLKILQASFSYFTFLTSMQRRQMSN
>DmelOR13a
MFYSYPYKALSFPIQCVWLKLNGSWPLTESSRPWRSQSLLATAYIVWAWYVIASVGITISY
QTAFLLNNL
SDIIITTENCCTTFMGVLNFVRLIHLRLNQRKFRQLIENFSYEIWIPNSSKNNVAAECRRRM

VTFSIMTS
LLACLIIMYCVLPLVEIFFGPAFDAQNKPFPYKMIFPYDAQSSWIRYVMTYIFTSYAGICVV
TTLFAEDT
ILGFFITYTCGQFHLLHQRIAGLFAGSNAELAESIQLERLKRIVEKHNNIISFAKRLEDFFNPI
LLANLM
ISSVLICMVGFQIVTGKNMFIGDYVKFIIYISSALSQLYVLCENGDALIKQSTLTAQILYECQ
WEGSDRI
EIQSFTPTTKRIRNQIWFMILCSQQPVRITAFKFSTLSLQSFTAILSTSISYFTLLRSVYFDDEK
KLD
>DmelOR82a
MGRLFQLQEYCLRAMGHKDDMDSTDSTALSLKHISSLIFVISAQYPLISYVAYNRNDMEK
VTACLSVVFT
NMLTVIKISTFLANRKDFWEMIHRFRKMHEQSASHIPRYREGLDYVAEANKLASFLGRAY
CVSCGLTGLY
FMLGPIVKIGVCRWHGTTCDKELPMPMKFPFNDLESPGYEVCFLYTVLVTVVVVAYASAV
DGLFISFAIN
LRAHFQTLQRQIENWEFPSSEPDTQIRLKSIVEYHVLLLSLSRKLRSIYTPTVMGQFVITSLQ
VGVIIYQ
LVTNMDSVMDLLLYASFFGSIMLQLFIYCYGGEIIKAESLQVDTAVRLSNWHLASPKTRTS
LSLIILQSQ
KEVLIRAGFFVASLANFVGICRTALSLITLIKSIE
>DmelOR98a
MLFNYLRKPNPTNLLTSPDSFRYFEYGMFCMGWHTPATHKIIYYITSCLIFAWCAVYLPIGII
ISFKTDI
NTFTPNELLTVMQLFFNSVGMPFKVLFFNLYISGFYKAKKLLSEMDKRCTTLKERVEVHQ
GVVRCNKAYL
IYQFIYTAYTISTFLSAALSGKLPWRIYNPFVDFRESRSSFWKAALNETALMLFAVTQTLMS
DIYPLLYG
LILRVHLKLLRLRVESLCTDSGKSDAENEQDLIKCIKDHNLIIDYAAAIRPAVTRTIFVQFLLI
GICLGL
SMINLLFFADIWTGLATVAYINGLMVQTFPFCFVCDLLKKDCELLVSAIFHSNWINSSRSYK
SSLRYFLK
NAQKSIAFTAGSIFPISTGSNIKVAKLAFSVVTFVNQLNIADRLTKN
>DmelOR56a
MFKVKDLLLSPTTFEDPIFGTHLRYFQWYGYVASKDQNRPLLSLIRCTILTASIWLSCALML
ARVFRGYE
NLNDGATSYATAVQYFAVSIAMFNAYVQRDKVISLLRVAHSDIQNLMHEADNREMELLVAT
QAYTRTITL
LIWIPSVIAGLMAYSDCIYRSLFLPKSVFNVPAVRRGEEHPILLFQLFPFGELCDNFVVGYLG
PWYALGL
GITAIPLWHTFITCLMKYVNLKLQILNKRVEEMDITRLNSKLVIGRLTASELTFWQMQLFKE
FVKEQLRI
RKFVQELQYLICVPVMADFIIFSVLICFLFFALTVGVPSKMDYFFMFIYLFVMAGILWIYHW
HATLIVEC

HDELSLAYFSCGWYNFEMPLQKMLVFMMMHAQRPMKMRALLVDLNLRTFIDIGRGAYS
YFNLLRSSHLY
>DmelOR23a
MKLSETLKIDYFRVQLNAWRICGALDLSEGRYWSWSMLLCILVYLPTPMLLRGVYSFEDP
VENNFSLSLT
VTSLSNLMKFCMYVAQLTKMVEVQSLIGQLDARVSGESQSERHRNMTEHLLRMSKLFQIT
YAVVFIIAAV
PFVFETELSLPMPMWFPFDWKNSMVAYIGALVFQEIGYVFQIMQCFAADSFPPLVLYLISEQ
CQLLILRI
SEIGYGYKTLEENEQDLVNCIRDQNALYRLLDVTKSLVSYPMMVQFMVIGINIAITLFVLIF
YVETLYDR
IYYLCFLLGITVQTYPLCYYGTMVQESFAELHYAVFCSNWVDQSASYRGHMLILAERTKR
MQLLLAGNLV
PIHLSTYVACWKGAYSFFTLMADRDGLGS
>DmelOR24a
MLPRFLTASYPMERHYFMVPKFALSLIGFYPEQKRTVLVKLWSFFNFFILTYGCYAEAYYGI
HYIPINIA
TALDALCPVASSILSLVKMVAIWWYQDELRSLIERVRFLTEQQKSKRKLGYKKRFYTLATQ
LTFLLLCCG
FCTSTSYSVRHLIDNILRRTHGKDWIYETPFKMMFPDLLLRLPLYPITYILVHWHGYITVVC
FVGADGFF
LGFCLYFTVLLLCLQDDVCDLLEVENIEKSPSEAEEARIVREMEKLVDRHNEVAELTERLS
GVMVEITLA
HFVTSSLIIGTSVVDILLFSGLGIIVYVVYTCAVGVEIFLYCLGGSHIMEACSNLARSTFSSH
WYGHSVR
VQKMTLLMVARAQRVLTIKIPFFSPSLETLTSILRFTGSLIALAKSVI

>DponOR2
MLYPVRKGLPFYPNLLILKLAGYYPKSSNYNKKLFFYCLFCWMLIWTGTSWNLIILLYLSI
QNKTNYGFV
EAMGYLLGITSFTLACLHFALKHEDWSHLMDDLMDFQKYGKPPKFNKVQTSASKLGIAF
TTGFFSAAVIY
AILQVLLEEKCEKLSFGNTSCGMLLPTWFPASYAENKLIKRLVLIYQVLACCTMAPYTMIV
SLVLEANEF
IAYRIDHLKSQLRKVGCNGDSDLFLASVQYHHDIIRQVGQTFSIYMDLCAHCVF
>DponOR32
MNSKVNQAEKLDPVDKRSKVFPSSDFLRLYAIFTGQMGIFPWQLMFERNKTYQNLYNLYS
KLILSYYMYV
TVSMWLALVFLCLEDTLRIPEITKNITVSVICTVTIIRLFVMKLHPAFLRNITFIIDAEQYILSS
NDAEV
HRIYKNCKIISNRHTIFFIVLSYLMALFISLRPFFTDAYEINYKNESLQITSLPLSIWVPLNEQE
HFLSV
YFWNVLNNLVMTSIVLSIDIITFLLLIYPVGQLQILHHILSKFENYKNRMKLNYPGLDDDTI
GAITLKAC

IDLHRNIIAYVDDLNACMNIFMVVDFAQSSLLLTSVFAQLLWVEPSITFYGFVFMYVTYLN
QRLFMNYYY
SNEVWLLSENLNLSVWKSNWYEQSHYVKFMIYFFIMRTRKSLKFKIGPFGFMNLSTYIAIL
KASYSYIAL
LHSTQK
>DponOR12
MEKDKFKILSLHINVLIFLQFWPNPLFNNVVNNHIMSIVCFITVTSCIPCIWTIYKVFEGMY
DIGILFES
FICFVNIMAYLTAYWTIFRNKAVIENLINDICIFLPYCPTNLIRDTDASSIRYTKYLIVYVTLG
VFVNLA
WPAISPEGCMRQRQSEYLLKHDPCGMPTHNYYPFDASKPIPFWIAFACEALLTCNICILFSM
VTAIILGL
LMQITEQIKHCCDKFEHINFKGDVETARKEFLECVRYHRAILEYAERVFTVFAPVMSAYLV
VTSFATALI
GYQIVETDNTQDRFRYAMLLLAWGCLFFMICLYAQILQDESVLIADALYNSDWTCNSIYFR
HYIIRVIAR
AHKPLYFNISFLGKISLTRFVSVMKTAYTVFTVLVTVVDRK
>DponOR36
MKPIEDQSLFRACKILVLCGGMWRGNIPNWPIAHQKLYKVFLRGAQFAYFFCLPSLVLSLW
VNVDQDNEK
AISVLKNITFVVVIFCKMIIIQSRPVTMLIEAASEKEQQAILSEDPQISEIHRRHVVYTEFVVK
SIMLCT
FLAGLAYVVGDLYLANEFYKLHPNAAPTDPKPHSIYFWFPFNPDEYYKIALTYEFVHIVQT
VIYNGASHA
VVNSAIIFVKVELKILEYEIRHMMSKPNLSNLTPAQLMKIHIRKHQELIKWVCKFNDSFKYI
ILLEYSVV
SLTLASTLTEILQGIKMVFNGIFFLLSTLSLFILSWNANEIIVTSVFDLSDALYHFPWYELDKE
AQELVL
FMMLRCKRSLNISNGPFGFLTLRGAVSRLKLAYSVVSVLSR
>DponOR3
MMENKSYRIMQLHTNLLKCLFLWPVDTFSPGLNSLLMYGSFCISMCCGIPIISAAGYQFYV
GIEDVNILL
EALIGVYDIIGNTVTYVCFLRKQRQIQEIIDDIKDFFQYCGYETVRKIDSEIMCHTKYFLFY
VTVSVILN
LAWPMLSVNNCLKSRRSDFYIKHDPCGMPTQNLYPFEASEGIVFWVLYIIEAIFCYHTCCFF
SLATVIVI
GFLKHITAQLKCCAYKFEHICDYMGSCKNDENVIIREFVHLIKYHQRIIKYAEKVFGIFDVM
IIVYIGVT
SFTLAIIGYQIAIPKTNLEDRIRYTMLLIGWVLLFYSICFYGQQVRDESMKVGEAIYKSEWY
QHQTLLGM
KTDIMFVMRRTQKPLDFKATLLGEVSLIVFVAVMKRAYQLFTLLLTVTEDGP
>DponOR33
MKNDFFGFCIPLARFIYIMPDKTPQNVYGWRNKLWAVFMYGLAVFCHLTEIIKLFQIVTAK
YFLLGEFIR

NFVITSLHFTSLGKAMFIGGKTGKKAFEKILDFEKHVYKNLGDDIRLIYKNKVTSIQKVKK
YYLIGIILV
VIFYVAAPIFREPIHIQDGNQTIRFRQVPLSSWSPFEQYYWLTFIWTGLTGIYLSIFFVTTDLI
CYSYVQ
MINEGMKNLMVLDFLPGSVQLAGMIYQMMTNLSVIQCILLGQFICSLIARVFIYSNSANNL
SQLSKQLAV
DWFEIDWTELPKDVTNNLNFCIMRSQKNLQITVGDLSVITMESFLTILKGTYSYLMLLMTI
>DponOR6
MIKQPPYEVYATDFFSVNRWILKCAGLWPPSTPNRVVRRLYQLYTIGVFLFVNLWFTGTEF
VSLFYTYKS
QYELIKNVNFFLTHFMGAVKVILWYFYGHLLRDIMNALESPQLHYEGYADFSPHRISHLHR
AIGRRYSLL
FLCLAHATLISSYIPPLIAVAEYLNQPQGGLQKLPSRLPYFCWMPFSYDTPGKYLLAVAYQA
GPMFSYAY
SVVGMDALFMNILNCIAENMVLIQGAFKTVRERSTLHYCVGALAPPCDAIREHPLVLRQM
DLETKKIIKH
LQITLRACKHLEGIYHIITLSQVTATLFILCTSLYLISTASPFSKQFFAELVYMMAMLFELFLY
CWFGNE
VTLKYEQLPMHIWESQWLATDDCFKKQMIFTMLRTNRPVYFTAGKLARLTLPTFMSILKT
SYSIFALIKN
FSK
>DponOR40
MYSKQESLLGMLKPMMMFTGTWRLDGMNSTVRWFYWLYSLIFHGFGVLFIISVVAKFVE
FVKSGADSEDI
ISSQVFLLSGTCIFSKFLIYQICNVSDILKAILQEEEKIWLSKDSESISAYQADIKHVRKWNW
GILLSTM
FTGVALMSAGVASLIQADISSINSEGNEKEEWSMIPMWLPYNEREHRSTVVVLKCIFTFIYV
CMFIVSGM
TFVALMIYSLGLLKMEQVKIGKCNWTSYNMADLSVDMKTLLINNRRVFRFIKHLDRSIRY
VVLVDVLLNS
ISIAALATNITNVQRGDFVFCTGFLLMQVTQVFVLGWFANDIIMLSRTRADVLYNLNWYY
LDLKNRKLFG
MMLMQCQQLSVISIGPFGPMTIGSVISVIKAAYSYMMLMQSYK
>DponOR37
MFSASKWVIMSSGSWGLEVDSKYRILYKIYVLYIRFIYITSTVAVFAMFLVNLGSNNDKAIE
ALSLTLCS
VSCIIRLAVCLKQKVVNLLKIVMEDQFNYAVNDPKIKVMLQEYKSYVTFLCVFVVCYTYS
LVILFNIFNG
IIEFQSFRKLHPNATEYPQYLVSIWLPFNVQTHFTLALICQTVLFLQSCVLNYSSTVLFNTL
MIYVVIKL
KILQHLFQNFNTYPKNLENFHMELRDVLAIDNLKHLIRQHQDIISFVKELDKNIKIGVLIEY
TITSLMLA
TISIQVLTGNKVASFSFYGLILIYQLFLLSWNAAEIKTQSEKIAGAIYATDWYVYGPGVKQII
HFIIMRC

SKGLSLDIGPFGPNDLGAASARLKLAYSYVSVMGNNK
>DponOR15
MKPSKTKKAQLISWTKIFMIGGGFWNQPLSKSYIGEKVYFCYSIFMKCGCLMWWSMMV
AELLRFVAYGYT
LDVILAQFGLVINASKIMFKLIVYIKENLLALFKDITEKDIEIWNLDNEEIHTIYRKNIKLIKS
YVLALS
VSTSLCLGMLDVSGIHIILKTVEHNRAFNDTLEAHAMYETILPLNKLDNLPLLFTWQAYLA
VIGLLYNCL
THLMFATLLVYAATQIQILQIRSKNFIGTEQLSDSDMKDKLLVLKDISKDHQYIIGFVENLNS
RTRYIVL
VEFILSSFDLASVSVNLITLDFSSNDIAGQLIFNLSFFVLLSIQISILGWSCNEIKCESEELANA
LYASN
WYLLNPKGQKMMQIMMARAQKPLIMTIGPFGAMTTTSVLAILKGAYSYVSIMKK
>DponOR8
MAILFALSMLSLTCVRDYYIYNSGSEVIFLSWGLGDSAFETDWYRQSRHFKFMLQFFIQRT
QKPLKLKIG
PFTDLSLAAFLSILRATYTYLTIVTGFDG
>DponOR5
MYGSLRLKVLRIGMQRFHHSPCGGVNMKSFVVEHLDSIRYIDDLNEATKSILFITFLLNSIK
VASVLFPL
MAVKSLSALAFPAIFSSMLVAEVFYLGWICNEVKEQSLKISTDAYKILWYNENKDVSVLLQ
MMIRRAQRP
LTMQMGPFGPMVTDTILSTMKAAYSYTTLMLNARE
>DponOR30
MFIRNQRAFVARTCKLAGLYPVQLLPEDENLRKLYTIYYQALIMLYFICLISFCTELFHLLR
AEKATVDD
ILKSISMTTLFAMTALRQWVIRSSPDVQKILRKAGNVEQRVYEENDPEVVNIFERAGHVAL
LYYIYYAVG
TVFLCLGCILEPLYDNQKVFSGNATAFSRKLPLPLWFPYDIQAHYWETFCVTILLICLLVVF
QVAVDVLF
FYFIRSPVIQLEILHHFFKRFNDYTGRISVEPGNVASNVMMRKCIDMHRKVIKFVDIFNENF
SNIIVLDF
VQSSFRLASISAAIIMIESFTVTSFVFTLIFLWITLVREYYIYHAGNEIIFLSSGLVHSVYETDW
YIENR
QFKYMVKMFMVRAGKPLDIKIGRFGSLGFPALLSILQASYSYVTLVRGIQKS
>DponOR45
MAKTLSFIQLSKFYLLLSGLWPFKISDNFLVDKIYRIYTLCQICYYLCVIFGLLVNLVILIIRF
DQPQRI
IGDINLFIIVFECCLKVVIFQIGKVPFMLNQITQFEDSMEASGDAEVKDYYAKDAIYCRRIN
VIQSIATI
IACASFAQDSVVTFITSDDMSVFKDNPFMHDLWYPFNREDYIYLVICIAFICDMQGLVCNA
ACQTTLLCL
MIYARTRLKILQIRLRKFDKIAVEEYEGDVVRAVKDLIAEHQYLINFVKSLNDRTQHVLLLE
FMLNSLCL

ASGTSQFIIIDTTSGWLATVFLNLYVIVQIFILSWHANEISVEGLAVSDAIAASQWQKQSKEV
QKLLIIM
MMRAQKPIGLTAGPFFRMTNSTAVQTMKVAYSYASIMTQNMPE
>DponOR7
MANLKQALQIYDICSFLEGEHIRLGIGGFYPRRIKRTFIVNLVTVFAYIITIAQMAVVINFVLS
ITDIVT
ITEVLLFSMTQVGFVNKLVNFHRNSRKVATLDELISQEIFTRVTVAEMDIMKTSFQRCQKV
LNIFLLSCF
GVTLLYGVVPAVNGIMTGTKMYPFPGKFPFNPDDYFVLIYGGEVATVAVSAWNNGAMDC
LFTKHTVIATT
LFRILRKKIKDLHYNTNEGERPLENRIKHCVRYYNEIIKYVSAIENIFAYGILVQFMCSAIVIC
LTGFQL
LVVASESGQSGLLVVYLFCMMFQLVLYCWYGHMLMEESNRITEACYAINWHEMKIGQQK
MLITIMERAKK
PIALKALGIFRLNLSTLMTILRSSYSYFAVLQQIYRNDKIMALVTN
>DponOR19
MYPIRKDLPFYASLRMLESIGFYSENTNGFKKRSIVRTLIFCILCWSIILLSAVLLIYENLNDK
NYASVF
FNIAVAVASTSTYCCTLLFVKYQEKWSDILTALVNYEKFGKPRRYNQLKERGDRVAMACW
GGILTGVFLY
MLFAILHENDCELEKTGGGVCGLIIPTWLPAPYDNSLLARRLVLLYDIPNAAAVSSFVLVTH
LNIQVNEF
NIARIDHLSLLFNDIEFCKDPQAQLNKMKHCIEYHQDIIRVSLQFKNLSKRTMGHMTLTFTI
VTASMGCQ
LLQTSKNFYQENAFFFEIIYVINMFIMCYCGQRLEYKMKTVGDFLYSTHWYNLNPKLQSLI
PLVILNSQK
TIRMDAVPIGYLNYELFVTLLKTTFSYFSVLTQLT
>DponOR23
MAIYPKCRLIQISMISSSLVGTFPWQFMFQDNKILKNMYAMYSKLMLGYFTLFVFSQQLEL
LILITDEEV
MRNAIFANISVTPIYTITLAKQLIMMLNSSFRATIKQIIDTEKCKSPIEDDEVFEIELRIVQRSN
KLVKY
YGLMMFVLGTLFCVKPILMTPNIVSSGNTTKAIGFFPLSSWFPFDEQKHYPYAYMWQTLS
LLQGTMYVTT
TDILMFNLIVFTAVQLRKLKHLLKNFVHYKERFMTLYNIVDDEQAAKITLIYFIRRHKEIIE
YVRLFNES
MEIVMVFDFLQSSLHIASVLPEVLMSEISLMVVLTVASFLGSMLFRLSLYYYHANNVIILSA
ELSYSIYE
SNWFDQTPKVKQMILIFMLRTQEPLTLRIGGFAVMSIESLIAILKATYSYVMLMI
>DponOR24
MSVLGETTQIKKKWRSSIAITEKVLVITEIWPNDDTSLYRTMKVVFITIVCIVFNLTVIDELK
MLAIRQD
YKTLSMHLSTFGLYIGFSVKIILFQFTKHGPLKNMLDSMDSPIFHAYPPEMQKHQDNCIRVS
NLIGKFFV

YLVGGTILFYLNKPFYSSYPLPITFSHPLTTTTFYLLLTLQCVCSFYLIMIGICFDMLVMGLA
NVATAQL
DMLIEEITTFTPTSIETLEKEEHRFIKRCAERHNAIISYVNSIEDVFTYIFLAQCVVSVTCICN
GLFQLT
HVAPVFSIHFYYNCIFTFNVLFEIGICCWFATLMTNKGNDVADACYNYNWLHSSSATRKLL
LIMLCRSQK
PLFITVGKIIQLSIGSFLSVLKTAYSYYALMQHLYDKTSQ
>DponOR25
MAIYPKCRLIQISMISSSLLGTFPWQFLFQDNKTFKNMYAMYSKLMLGHFTLFLFTAQLQL
WILITDEEL
MRNAIFANLSVTFIYNITLAKQLIIMLNSNFRATIKQIIETENCKSPIEDDEVTNEIEFKMVQR
SDKIVK
CYGFLLIVLTILFFVKPFLMTPTIVSIGNTTKVIRDLPISSWLPFDEQEHYSYAYIWQVLNAL
QGSTYVA
STDILMFNLIVFPAVQLRKLQHLLKNFAHYKEKVKTLYNIADDEQAAKITLVYFISRHMEII
QYVRRFNE
SMEIVMMFDFLQSSLHIASILPEVLMSEFSVMVVLMVASFLVSMIFRLILYYYHANNVMIL
SAELSYSMY
ESNWFDQTPKVQQMILIFMLRAQEPLTLRFGGFGVMSIESMIAILKATYSYVMLMI
>DponORco
MINKFKVVGLVADLMPNIRLIQASGHFMFNYYADNSGSLHILRLGYCCMHLFFVLVQYGC
IFGNLVKEKD
NVSHLAANTITILFFTHCLSKFIYFAARSKLFYRTLGIWNQANSHPIFLESSNRYHALALKK
MRSLLYII
LFGTIFSASAWTAITFVGESVHFIKDPDNDNETITEEIPRLLIKSWYPFDAMSGMTYYVALV
FQIYYVFF
SLFQANLLDNLFCSWLIFACEQLQHLKEIMKPLMELSATLDTFVPKSADLFKSPGSATSQD
HLIENDFNA
KNDDLKGVYSTRQELGNLNFRSGALQTFGQGGGGVGPNGLTKKQELMVRSAIKYWVER
HKHVVRLVTAIG
DAYGVALLLHMLTATVMLTLLAYEATKIDGLNTYAATTLGYLLYSLAQVFHFCIFGNRLIEE
SSSVMEAA
YSCHWYDGSEEAKTFVQIVCQQCQKSLFISGAKFFTISLDLFASVLGATVTYFMVLVQLK

>ItypOR28
MGLYPASRYFKNPIMWSSILGAFPWQMIFQENAKLQQVYRWYSNFMLTWYFGMVTTEYI
QLYHILNANVI
QMDEVCENVCMSLVFTCTGLRVWVMRRTNGLSEIIQTVVDAEREADGLDDEKTRQYEDI
HVKHMEKVSFI
YAAFVFMSVTNGCLATLYADTKSVIIGNSTIVEKPLIISTWFPFDKNEHYWVAYGLQVFDG
YMAALTVAC
TDILMFNMISYPIGQLTKLQHLVRNMAVYKTHFEAFPTFTKIVQRHKHIIKYVELFNQSMG
TFAIFEFVQ
SSVQIASVLVQTSPDDLTLMSFCFIVLFFTSMLTRLFMYYYSANEVIIQSINLGDSVWESSW

YHQPHQLK
QAMLMVLVRAQKPVSYKIGGFGIMSMQSIVAILKATYTYISVILRN
>ItypOR31
MHIFPKSDHLDFSFALCSMLGILPWKLVFQDNSFLQTLYYLYSKTLLIITVIFITTEWMEVCR
ILNXDPV
NLTDLNNAIAPVLLFTVTAIRMIIFNRNPDFMKLLNYIINRQEFMAQRDDEIRKPSQKFIKIN
KWTGVGY
LIMYLAVIYQLLALPLVLGPIEQQTANQTTTIRILPLLSWIPFDTQQHYWGCYLWQALNLQ
LASCNICHI
DVLMFALILYPIEELGYIKHVLRNFDSFKSRTGIENSNFASITVFKDVIKIHNNVINYVGTVN
DTLSFVM
LLDFLQSSLHIAVILGAVVVGGPTDLASLSFVGTHFFSMVLRPFLYYYYANQVMVLGGDLT
KEVWNVDWF
DESKDVKYMVRFFNMRAQKPLQYFVGSFEVMNLQSFISILRVAYSYVMLLHTLQ
>ItypOR23
MAVYPKSEYLKVPAIYCSTIGIFPWKFMFQDNKNLQTIYRCYSIVMLAWCIGFVVTDYIQL
VILLTSKTL
DMQEISFNTCITLLFTCIGLRAVIVYFSPNSANLIQSIIDSEKVTYLDDAECMKLEKKHLRSV
RLISHCY
FIFIIFSTTSRCVYSFPKEPDIIQNGNETEIVKEHMLSIWFPFNQEKYYLTVYNIELLDSFLGT
FFVAYV
DIYTFNMISYPKGQLKKLQHIMKHFHNYKAKYSSETNEENDFIVFKDLVQRHKQIIQHINA
FNELMEFVA
IFEFVQSSAQIACGLTQSSLENLTIGSFLFVMSFLISMLVRLFLYYYAANDVTVESTKLAQCI
WESNWYE
ESQKIKLSMLMVIIRAQKPLIFKIGGFGAMSVQSIVTILKATYSYITLAYKRT
>ItypOR16
MYNIPENERKNYFLKFSRVTMLMLGIWPVRRGGDLLEKLYESYFLTTFLYYIAFNLSGLAL
AIRTWSNNY
LTTASSMGIVIEYMSNAYKVWLFKTSVFKSLIKEIQDREREIFEGPDEAFKEIYIRNAESNKK
VVLFYTI
MGTSGISLYFITPLVSNVLMPLGYNNVTGVYEHYFIVFNWFPFDPNRYYWAAYLIQFTGCL
IGYSYIVHC
GAFYISILNFIRTQLKILRHVIVNMSEYSLLYKNTYKLTEEQSQFVLLRAVVLEHQRIISFVT
KTNHTIQ
LFTLINFVISSFQLALLVYQIFQVAILQQVTVLSYFITLSTQLFLTYYAAHMILFESSNIASSIF
EGNWD
TYPPQTLKLLQMICMRAQKPLAMTIGPMAAVKVTALFQIFKALYSYICLIIKF
>ItypOR34
MKFRELIHNDFLGICIQLGYYFCIIPEKAVTTDKIESRNYFFYTCIVRTLILYCHICQWVKMY
QIITADI
FIFDELVRNCAITSIHFQSFVKTSIFRQNYQLFENVIDFENVLYKNNDQKVLLIYRDTLQAIK
NSRLVYV
FGILIVIVFYIAAPLFRGPYYVEMGNETVTIIQLPLSAWSPTNNYFSNFAVTGAMGAYLAMV

FVQTDLLY
YCFLYFSICQLNILEHYIVHFHHYSNELVNDHKCSHVMALSLTQKIYIKYHQNIIKNVKQLN
DALKNSLL
IDLVPSSIQFANQFYIIATNLNIMQCVCIGFFTIMLMSRVMAYCYLANQISVQSQKIGSAWFQ
MDWSDFP
NEMKKMISFCIMRAQKPLVITLGNFGNITLMTFVGILQASYSYVMLFITL
>ItypOR2
MKVLQRETEITFFKFNIWVLKTCLLWPEDLNYKYDKKRFIKDLTMVASLMPCFLPILADFL
QQLYEEVPD
LTEAVENMIALNCLIGMFYMVICFVRNRRMIIQLMIDIRTFNKYGNDSITQEVDNKANLFS
KMFMFYGIL
GNFVYMAMPQIRVSKCHLNRTEDMIEKGVPCGLVVRSYFPFKFDYSPVFEIVFVHQIYTCT
MVSVVVLVL
TMLFCGFLMHIVNQLKHLRVLIARLKNVPPEKFERKLIFVVRYHVAIIQYSQNTAGAFSTM
LLFYITLTS
VVLSVLCFEILMVDAFADSVRFTLHLLGWLIILLSICYNAQLVLDQSQEVANDVYSLDWVS
ILSVDVQKK
SKXVIMRSQKALVMEAGGMGVVSLSAFLKVLSSAYSFFTLLLKFK
>ItypOR8
MSELFLLHFPRILMVICGVWRLPYFKSKKVQTVYDIFSIFLQFTFSLMCLSMFFELVNLINT
WNVLNLIE
FSRVALSSYLCIIKALVLRNSSIQRIMVYMIKEERNVLRSKHQQPKALYMDTVKLINRVSFL
LLLVVLPT
LLAFSADCLRKGIIDFDDAVKYVYLPLIDQKKYKTVQLTVQTIFINLIGFYYCMTQAFMVT
AMKFAQGQL
ELLQLYFREFDYYAARQSTTEIAYLKTLLEYHQKIIHFVETLNKEMRLVIIIEFFFSAVNIACS
LFTLLT
MATNLIDILFSVNCLVFLLAQLAILSYLGNEIYQAGLNIASASYELKWYEKNREFQKNLLL
VIKRSQKPL
VLSVGPLGPLTNETFVSVLKASYSYFNLMTRYN
>ItypOR17
MGLMTTPRFFLQLWGIWPVNTLLPPKJYVMYRFCIIGWYSFFNIFQFIASIRLILNNESFERI
SRCISVM
VTLVLMLVNSLIYQKNCIPQLCSTVMEIEQXLARSNDAKITQTYHTTVAKNKYLNLYIVGS
SLFTLVAFI
GLSLLDVIKAGPAFWDFDNVTFMHELYVPFNRGNHQALIITTNIFTACESVVVNGVIQTTF
YALVMYGAL
RFKILQLNLKKIEQTEGDRKWRMRELIQDHQYCIRFVGELNQATKNVLLMSFVLNSLKVA
SVLFPLMAIR
EFTDLAFPLIYSSMLVSEVVFQGWMCNEITEQSLQVAQTIYDTFWYKESKQYNVLLQLML
MRAQRPVTMR
IGPFGAMTTSTILTTMRAAYSYATLMMNSS
>ItypOR18
MYTLSKNKPFYYALVLLRAFFWYPSSPKCSVTFILCSLVLRLLSTLAALGTLAHLVLNLTG

ETKAEISED
IGDLTGFVGCMSACLNFLWHRSRWSSFINRLLTFKQFGTPPGYVKVVRRGNLITLACILYTI
PGMLWYSH
LTHLDIPRCEALNREFGMKEACGMVNPTWIPPGYDRRNGWRFWVLYVLQCTGIFVYLPS
TFVISNIPLEA
VGVIVTRIBHLKYHLKRCGSDLGRLYHCVKYHQDIIEVSKELSDLVQATLGTLLLTGAVVI
GSLGSQVIK
ASTPKAVTFILGYVTTIFMVCHAGQKLINQSLTLADQVYWMEWYEQQPKIRKDLRFVLAR
CQKPLGLVGP
PSMGXAGYSLFLIMLKTSYSYLTLLNEVIS
>ItypOR4
MDKQSQILKFHVKVLKFLMIWPFDGLDPNQNYYLMRGCFAYACFCSIPVFSGAAFQFCVG
IDNVKVLLEV
LVGVGNITGYNIAYVCFLKNQEKIQFLIRDFQEFVQFSGPEIIQNTEEKTTRYTKYLLGYASI
GLVITFS
WQMLSTESCVAQRGGDYYVRHDPCWLPVRNWYPFDASQPKLFWIVFPIEAIYSIHICLFFS
LATSTIIGF
LMQITSQLQYCSNRFEHVFDEVDLKPFQQIKPDFLFLIKYHKKILDYSKKLFNVFDALIVV
YISLTSFIM
AIICYQIVDPKISAQDRIKYAILLIAWCLLVYLICYYGQKVQDEALKIGQSIFKSHWYGGTTA
VELKPYI
LFTLARTQIPLEFKAQLFGTISLLQFMKVMKWSYSGLTLLLAVTDED
>ItypOR13
MSNYQRYNIQTFLKEERLFLGITGFVSGNLKLHLPTGVTYVLTTMQVLASIYYGLSTTDLA
EITAAFMIT
LSHINTLNKLFGLHLKSLTQLDRILVKQIFALADDRELTILKRTLTACHNMLTMYLVTVLGSI
LLYGATP
LVANYTTMERNYPTLAKFPFNPDDYYWAVFAGEFFIVALSALSNGCMDRLFAKHVAIATGL
LKILRHKIK
QIMDVDDQKVIEAKMKHCVLYYNEVMGYANQIENQFSFGIFIQFLCSCIVICLIEFQVLLAT
SSETIGLL
LTYLTCMITQVTIYCWYGHQLMEESNSISMEFYDLNWIEMSVKNRKTMLTSKERAKYPIV
LKASGVFPLN
LATLMKILRTSYSYFAVLHQVYTK
>ItypOR9
MFSSKNLYKYYSHFASTSIIMYTIMLTIRLVQLVIEGQTPSAKLYRCFTINIVIYMMTANLIIF
RRYGLP
DLISQVMKDEEAALNSLDKDIRKTYLAQTKIYEFTSVAQVVSTFASGLMFIALNVYMKVK
GLLKHEAFMY
ELWFPFNRENHDGFVIFFNLYIVVLIMFCNVASRIIPQTMIIYANAQLRVLQILLEKAFDAPC
SDPLVKI
QELVKKHQDLINFITFLNSALRNVIFMEYIINAINVAAGLLQFITVRAAMDLVYAFVHFSLL
VIQIFVLA
LNANNVSTQSEAIANAAYNSQWMDQSNNIKKIIYIMIMRAQKPLVLNIGAFGVMNAESAL

TTMKAAYTYV
SIGLQR
>ItypOR19
MLIFGNIISANVIVLFGSSLINGDYFLVTSSFPFALAIIVVNGSTLSFAVNHKQWSNLFKSLTD
CQKFGK
PPNYDLLKKNGDRKGMICMTCYISTCTLFAIIEAVEEQRCLKNVSKHEICGFIIPVWWPTN
YHPSTFLKT
MVQVYEVGTIIILSNFTIIATLQYQVCEYIAAKAAHLGLNFNAIDPNSDAKTQFEQFKLFVD
YHQHIISL
CAEFDSSCKRTVGHVTFTTAAISALFSYHGMQGNYKLLAFLALYILNLGFMCHTGQNLED
AMLGISNSIY
SSKWYELNIRVRQRIPFLLARTQKRIGLDAVPIGYLNYALFMTVLKTTCTYLNLLNHTI
>ItypOR27
MLNGDVLKMDAICSNICLTLAFTCSALRATVMRVGPNLLKIIEQVMHAEKNPASIEDQTSF
NLERKSIKT
MRKLSHLYAVAITMIASSKCALAPFEKGEIVHIGNTTIIDRPLIMSAWVPFNKNTHYWAAYII
QIYFAAL
GAWHVAYVDMFMFNMLGYPIGQLKKLHYYIKNITTLTRNDDSLEEFKNVIRQHQQIISYV
KFYNDSMGTF
AIFEFLQSSVQIASIFIQTSPSDMNLGQFGFIGGFFIGMLFRLFLYYYTANEVMTESEKVGVS
VWESDWY
EQPTNLKMALLTVMMRGQRPLYYKIGGFGLMSVQSIVAILKATYTYLTVVVRNN
>ItypOR43
MDAALALMKNITFVAVVVFKTVVVQSDAIVKLVKAASVEEEKIRNLTDQAIRKIYKSNVD
YCNRVTKVII
TYLYGSGTIYVLDGLYKSYTYYENHPNVKPEDPKPHTVLFWFPFDHNRYYKIAIAYESFHI
FQTLNYNGV
AQSVVSSVMVFLKIELKVLQHHIRAIQGGSRDYQKVLIKCAIKHQQIIQWVNDFNNNFRFI
ILFEYSMIS
LTLATILIDILQGTKICFNATFFALNFTQLFVLAWNANQISDESSISISDALYACSWYEFDKTT
QDFVLF
MTLRCKKPLNISNGPFGYINMDAALSRVKLAYTVVSVLSTSTK
>ItypOR36
MKIPHMFEHAMKHERNIFKSRDLELIICYQEQVKYGRRVNLSQFLVTTLSTSTFAVTALLD
VYWAADMSK
YEKEPFMHDLWFPFRRETHMNWVIFFNLFMIVQGTCFNTATQATLINLMIYSSSRLKLLGL
KLRKFDAIA
SQNGRDILETVHDLIFEHQDLLSFVESLNVRIKYVLLMEFILNELGLASGIIQLIVIDTTSYM
VSVVTII
ILQLFQIFVIAWTANEITIQGAKIADSVMASNWVEQPTNIKKLFLIMVMRAQRPLGLTAGPF
FNMNANTA
VSTVKAAYTYLTFMMNNYN
>ItypOR1
MWITMFCTKNRHVLISLVQGLSDFTGFPPNFDKFMQQLNFYSKIHLCYLTGGSLMYFVLF

APLHKRNCDE
LKREKNLTETCSLLLPLNAPFVEYQSFGKFPTLQLLNIIIFLSLMYMYMCAGTIVWLNVEL
VEHIRIRIR
HLKHMILRALKSNDKQFRREKFRKAVRYHEYICSMSRLADEFFGTELFLHVVLTGAILGIS
AYLIGDGSL
ETVMIFVGWLNAIIMGSVAGQRLINESLGISDIIYEVDWYNFETALKKDILFFLVYAARNLC
LLGLGMW
>ItypOR3
MPTRNLYPFDASQGWSFWILFVIEAIFCYHTCCVFTLATVTLIGFLKHILAQLRYCGHEFET
IFDGVNEE
SGGKHLTLQHFIRVVKYHQEILRYTEKVFSTFNVMIVVYTGVTSFILAITGFQITSPETGGED
KIRYTML
IIGWALLFYWICYYGQQIQDEASQIADAIYNSKWYENTNTVVLVRRDIIIIYLRTKRVLDFK
VQFLGAVN
MEVFVAVMRRAYQIFTLLLSVT
>ItypOR39
MHDLWFPFRRETHMNWVIFSNLFMIVQGTCFNTATQATLINLMIYSSSRLKLLGLKLRKFD
AIASQNGRX
ILETVHDLIFEHQDLLRFVESLNVRIKYVLLMEFILNELGLASGIIQLIVIDTTSYMVSVVTIII
LQLFQ
IFVIAWTANEITIQGSKIADSVMASNWVEQPTNIKKLFLIMVMRAQRPLGLTAGPFFNMNA
NTAVSTVKA
AYTYLTFMMNNYN
>ItypOR25
MEVQHYSYNDSENKSIDDGVRSIIERHQYIIQYVXFYNKSMGTFALFDFLQSSLQIATVLLQ
FSPTVGTI
IFMLIFFALMLLRLFLYYYTANEVSVQSEKVKMAVWESKWYEQPPKIKYALLRIMTRAAK
PSKYIIGAFG
GMSTYSIIQILKATYTYITIMFR
>ItypOR15
MLNNLVSSQLLLQEFCMTCVVCCSIYRSTSPGVSSTEMGYVSTMGIIAAIEMLTVSWFNQC
FMLEVFELL
NRIYELDWLNYNTKMRKMLVFLMQRVQRPYHFTMGLGFPLNLGVFFTMIKTSYSLYALL
TNTGSKVST
>ItypOR29
MSTYTVFDFLQSSFQIAALLVQTSPNDMTFISFLTVFTFITTVMIRLFVYYHSGNELIFESVNI
SMAIWE
SNWHEQSPQIKSMMLLVMRRAQKPLCYTIGGFGVMSLQSVIAILKATYSYVSIIFRQ
>ItypOR33
MLIDIVPGSIQIACSLFQMVKNLNLVQCIILCEFTLTLIFRIFMFTSTIHNMADLSQKIGSAWY
NMDWIE
LPRDVRNNLMFCIMRSQRPLWITLGDIDTISMGSFLAVLKGAYSYLMVLLTV
>ItypOR14
MEYSFHYHDGFRSHMFLYVMLQGKIAVIILIDYVCSINHMKNCSETTMKHLKIIIKKHLEII

RYNQFVLK
SNQYFYLLYLFNEVFFGLLPMFEYFSPNRSIIAVSSATVYFFCLVYAVNDAAEEYITLVEYFR
TEIFNLK
WYEWDVSCRKVYSILITYLNEPMKVDFVVVNINRALFCQILKLCIQYLISFIQPTELVDSSF
CNSALYVL
SLIISHYW

>LdecOR94b
MSSVPYVEDFFVVNRWILRCAGLWRPETKNKTIQLLYTGYATGIFLFVNVFFTFTEFLSLIY
TYNNEYDL
IKNVSFALTHLMGAVKVIFFYFQGNNLIKIMTTLESAELHYENSFEREFFPGLISRTYKKEGI
KYTLLFF
MLAHATLISSYIPPTIAALQYEEGNPRKTLPEKLPYYSWMPFRCATSTTYLIALGYQAIPMF
SYAYSIVG
MDTLFMNIMNCIGMNLDIIQGAFATLRERSVEKTTGPLLTPDGLYNSQDLKIILNQEMKKV
CRHLQTVYK
VCEDLENIHTFLTLSQTTATLFILCSCLYLVSTTPASSKQFLAEIVYMIAMGFQLILYCWFGN
EVTLKAD
NMPFYIWQCDWITADSEFKRAMIFTMVRAKRPLRLTAGKFAPLTLGTFIAIIKASYSFYTVI
KNTSD
>LdecOR1
MPSHSIIEGSLRYNLLIMKIVGIYPYNNWPKIFKIYANFFYLIFTIPTPILILVNFILMEEKTLER
ICDS
AFLGAQLGTLLVKLWPFKSNPEAIKRTVKALNQEIFNSYRQDQECIVKSAIKEYNFIFLGVI
SASGVSFV
TWFGKIFFYEEKRFPLEIWLPFDAFEDTRIYVGVLVYLFFCVLIGALDNASLDTLIVGMIFQ
SATQVKIL
KNDLQFLHERIEQKVNDNFDSKGVKENRIYKSICKSIDHYDAIFQYAKDIERVYSTVIFSQL
FVSIVVIC
ISCLQLSIVEPFTILFFGMVIYIMTMLMQVFLYCYYGTMLYEESNTLTTAIYESEWYNYDHR
SKKCLLTL
MERAKRPIKQTAGKFFDLSLETFTMILRRSYSLLAVLQNY
>LdecOR82a
MFVNIILIVTFTSGLTVLITMIIFPLVEPPEGKRPLPSINWTPFDTNPSPIYEIMYVIVFCVLVM
TIIGN
AFYDYTLVYAVQHLRVQFTLLKELLKNITDGILPECSDFKKFNDEYFQRIVAERLKICIQHH
VKLLKFGK
NLEIFTSKIMVIQFTMSYVTLVVTGYVLNVSHINISQVVALSVLTGVYLVELLIYCAQGTDL
NQQSLSVF
EAIIESEWHLLKAPNKRALTLFLLNSERGITLKAGGTTSVDNELLVTFLKSVFSAITLLNSIL
EEKK
>LdecOR46a
MLLTLTEAFAKYAVLRMNQQKIQTVREKLLDEQYHYQSCGSFQPGKMFNDSKSFCRRIVI
TTFSVYYVVV

VTASTSVLVKLNKESEKIYFQGNITCHDFLPYSFVIPFPTPTVASCKYALVFMDAGSCILGSF
LASYDTT
FTSILICVKTKLQILSEAFKTIRVRALAKLNLPPKSQLEAVDSQLEEILYEEIKHCAKHLDSLL
RVCAET
ENIFKYVTLLQMLDSLLVMASCLFVASLITQSDPDFFAMAQYMLSVLTQLLMICYFGNEIT
EVSSTLSSS
LYQNNWIDCSKRFKQCMIIMMCRMQKKLSMTIGKFSPLTLNTFLSVVKGSFSYCAVFQQV
NK
>LdecOR2a
MAFGYPKNFFQANQFLQKVAGIWFPESDYNSVFRIMYLLYVLFFYGICLTFFYGTSIMLSE
SMKNLGTFA
THLGILLTNINGLIKVFILAVRWKKIRKVMGALQNESYRYVSLGESHPGFLLTKEMKVNSV
SSYLVSICL
CNIEIEICNGTHLLVMYGFTGISGNLATLIKLTTEIRGDDTFETTNKTCDDFLSFMYKAPFDT
DTKSHCI
IASIIVNLGMLAQSVIHASKKHSHDGFFIGLVICLKTQFRIIGNVLKAIRERSLESIGLPRDYSI
LHDES
NPVLEEELYGQLSHCTEHLKILLSVRDDIENIFTFVLLIQTLASLLIFASCLYVASAISMTSAD
FYAQID
FFFGVFVQFSFMCWFGSEISDASENINLSLYESDWLSCSPRFRKSMIFTMIRMQRSLYFSIG
KFSPLDLT
TLVMVCRGSFSYYMLFKSVQ
>LdecOR63a
MATIGAHPEMNNSKIQKLLFLMNLAVFSVITYLVCLMLFKEDELTITDITGAIESLLLLIHGA
LKLLTLH
LQHDGVRDMLEQTNHFWNIEEVEFEEERNEISKFLKYMKTILYYFMAFACMPTIFFCFRPF
LLHERVLVF
NSYIPEFVPYFIMLLVEDYAFLIIYVACVSFDIFFATLVVLTKVQFLLLNQEIRRVVAINIETDE
DANVV
RNKLKKCVDHHNFLLEFVKKINNTMSNAVLMYFLILIFSICVEMFMLSNRKTVKEFFKYIF
YSVSLSNEF
VVFYCLPSQLLTSEAEESIYYVYDSQWYENLSNTTKCIINMISLRGQRKVFITAGKFVNLS
MESCLAAYK
TVFSYYMFLMTMQRKDDTK
>LdecOR4
MYPIKRDQPFYSSLRTLRFFLVYREYSQEPWQIFLSLISVVLSCMTLTMFIIICILHASLSIEEN
IGEDI
SEDLSFIVGGLGMMITNILFKFHRKKWSRLFEDVTNIDTFGKPANFDGVKSQCIFLSKLYTI
YCIIGTIV
YTCISVLESSCEDPNEGIERKSICGTLSPIWLPVENVSTATRNLILLIQYILGNYMVIPPAIIAF
LVFET
TELLICHIGFLKENILESFQCEKEELESNRLRFCITYHNHILRMANQLNYVVKFNVGHMSLI
CAMVFGCL
GNQIFKTKPVGAAIFLFGYIVSLYLLCYAGQKITNESLSVAEVIYQSKWYEANTQMKKSILF

SLARCQIP
LTLDAWPLGNFCFPLFMMIVKTSYSYLMLLQQAI
>LdecOR42b
MEFGNKLRMFPKNAYSFIKVYVFFVLGVLSMMILNPILFGILPTKVWVPDGRVWFYTTCFI
QTEVHIYCA
IASALGFDTLFALIFIEATIQFKLLNRAFSMMQNHNDMRACVDHHVFLYEFIEKLKNIFSVF
LLAQCFDC
MVIVSIKMLVAVDPNQNVILRTKAIMYVIGINVQLSLFCFPVGFLKDELDRSPEAISSCPWL
LNNKTFRN
DIIFIMMRCQRMISFRAGGLFEIDRQAFTGVFKFTFSVYTLLGAVQ
>LdecOR85b
MCFIHKPSNIVQIDIQNIERFAIRINFSSSFQDDNRNKHDSSFELVIPCFVADEHSHFTSIIESAI
GGSA
LLSVIYVSTCFILKKKEIKRLIDSLKIFEDYLPEDEIDKAEKSAKFYTKTFLFYGIIGNGLYES
SPFMSF
KECRGERTQQMIKLGIPCKVIVRYVLPFKYDTTPFYELVILEQVAVAILGTILVMTISMLVCG
ILTHIAS
NLSYLKKIIEDISRVEEPQLKDHVNLCIKYHTIILEISDKTNNAFEGMMLIHITWTSFIISVLG
FGIIME
SNFWNNLRFVMHLGGWLMMLFLVCFYGQILMDKSTDISEAIYGTKWYEKNPNIRRALTL
LLLRSQRPLVL
KAAGLRIMSLSTFLGVLYSAYSYFTLLLKIKP
>LdecOR30a
MSQSERITTLLKDMLEAERAIIATSNEVDKRIYYDQVEYTNRINLSLTLYTYGLVGIPLIML
NYISYLQF
EKTHILTNTTESKPLPYVSWFPFDSDEYYFVAFGMDSVAAFMGSTYNCLVQLFFIAIMTFVI
GRLRILQE
HFRNFDEIDEFGKNIGGESGTDRNEDHDVLRNLKRLILEHMEIIGCVKKLDDSTKYLLLLE
FLMNSFQIA
SLLVQLLTSLELSNALYESRWYEHSVAVKKCIHLMMLRSQRPLTLQIGPFYPMTMSTALST
VKAGYSYVT
ILRK
>LdecOR13a
MSNIIEAISIPNKFFTLSGHWPGAKTTIFQRMLFCLDILFNFSLLAEFTNHFDDYEILSEHLSI
TISPVS
YLIKMIIFKIKSDAVQNLICSIKLKEFNDCPNHLRNIIEQTVKYSKFLGYVYQSLCLVTILLYS
LVPVFT
EADLPLRFSYNIGNFKPVMYAFQVMGLISAASNNSSMDILAMSLMRICAAQIEILNRKIIGL
SDEYEKYR
PDYIKTELKHCVKHHVKIIRYHEILNKVFSMIILTQYATSAVVICNLGFQLVHVDPMTLQFV
CMTVYFSA
MMIQLAMYCWFGNEIIVKSSAIRDACYEFKWFESDLETRKTIITIMERSKQPLVLKVGKFS
MLSLQSFTS
VIRTSYSYFTLMQTLYRKNLN

>LdecOR43a
MSYSHLYNFYTTACSFLWLLYLMSQAIYVITSLNDIVELTFLLFTGVAYSANFIKTIIMYSNS
EVIESWI
EKLNQPIFQPKCKEHYNMAQSSKKFYKKLFYTFLYFGVQSSVFFCILPFFRKEKSMVSVG
WFPCDWKVSP
NYEIIFAFQCSIIFWNTMTCLTLDLFSAGLLIQISLQCDYLIVTLNCLNTCFVENGVLRKRDE
SSGHLIQ
RDSKRFSDTITTNLIVCIEHYKEIKKLSKEIEKLHRTSIFFLFAGAEIFICSALFQITSVTTGTIE
FFLV
VFSLVTFLMEQFIYCWFGNDIIHKSEKIPDAIYNTPWLECDLQYKKILVNFMIQTKLPINIM
VGKLFPMS
IPVFKSIVQAAYSFFTVLKHMQDKRI
>LdecOR9a
MVDFGQVFETEKSILSFCGFYPGGKNRENRKPLRTFFSISGIILLIISIPMLVVEGEYEKLLDS
SVMFMI
ELTVIVKLLFLMTRGEKLCQIENFIRMMNSAKIPVSILKYVREDTKKRGRMSICFRALIVIY
LFIFGIMP
FLGQGPKVLPVIIWAPYDLEKAIFFYPTFLGELVILAISAFSNSSSDLMYYILVDIACCQLDIL
KENLRG
IDMNGNVDMVQKQLRMFVIDHELIIRFVKTIQSIYSNIIFIQCISSVLIICFQGFQLIAIIKFPSA
KYSI
DTVFILMMFYQIYWYCWFGQNIIIKSVEVGEACYLTNWYDSDLRVRKTITIIMERCKKPLK
LTANLFTLD
IAMLVSILRTSYSYMAILRTLYSE
>LdecORco
KNHRKNENFTFCFTKFCVTKCINCVFRCTFGNLVVERGDVNDLAANTITVLFFTHCITKFV
YFAVRSKLF
YRTLGIWNQANSHPLFLESNNRYHALALKKMRTLLICVMATTVLSASAWTAITFIGDSVH
NIKDPNNLNE
TIVEEIPRLLVKSWYPWNAMSGTAYYVSLIFQIYYVFFSLAHSNLMDSLFCSWLIFACEQL
QHLKEIMKP
LMELSASLDTYVPKSADLFRAPSANSQDQFIENDYNAKNEELNLKGIYNTRQEMGVNFRS
GALQTFGQGG
GGVGPNGLSKKQELMVRSAIKYWVERHKHVVRLVTAIGDAYGVALLLHMLTATVMLTLL
AYQATKISGVT
VYAATVIGYLVYSLAQVFHFCIFGNRLIEESSSVMEAAYSCHWYDGSEEAKTFVQIVCQQC
QKALSISGA
KFFTISLDLFASVLGAVVTYFMVLVQLK
>LdecOR49b
MIFPKNEHLKVCMYANALLGVWPYIFENNPTLRKLYNIYSKATFTYYLLYILTAIIQLVILLT
RQEINVK
EVFANLAITLLYSVTIMRVYAIKSTRMRNIIKEIMDLEDKTMASGDKQIIEIYEFHTTQSKVS
NVIFLVN
IFIGKTFSKYCLLKNIQIRLVNIPPLFVEDIIKFDVEKNETISIKALPLSSWFPYDEQKHYLAS

YLWHIL
DGSIGASFVMYTDIFTFSLIIFPLGQIHILKHILRHFNEYALKIKHQLGVTEEEASFITIRECIL
KHNSI
IRYINDFNKEMRNIMVLDFLQSSLQLASVVIQLLVTQMNLINFIYSAQFTVSMLIRLLVYY
WYGNEIIVE
SSNIAVAIWESEWYEESKKVKNLMLIMMMRCNRELCLEIGPFNTMSLNTLIGILKATYSYM
MVIYRR

>PmacOR21
MLEWNWYSKRSVENFLLIEMDVKMTENDDPFLDVRKLIYLDVLENRWFKAVLISFTFAHI
ISALKFLSQV
EGQAAICNTTLQLPFIINNFLILLIRIHYCNAVYQYCYSTRYCDDKLKKKIAKENRDAVFYL
KMWMGILQ
VCVAVATINFLTGNYKILRIDNFGVFGEKFKYLSNFITIVYILGTMWLHSIILNNFTYIVLHC
KIQFLLI
IEQIENLKRFKNGDISKENEGYQQAVRVTLKKAVIHHIQIKQMIKYMSHTNKTMVVFLSFE
GFLIMIGSL
WIVIVEGKTAFILAFTVVTITTTTLYRFCTRGETLISSSMDFYITLCNTDWIHWNQSNAKSL
LILLQATA
TPIAVSFFGIITVDCNLIKQFYKWSHQAISLIVKFQRT
>PmacOR13
MYLNFLQSFKTELAIVKSCGVWDYVFKCRYQFLYVLYFIIVNIGLAMYNLMKFNDMLQS
NTLETAVAAGF
VLPIALMGNIRSLCFFMNRKEFFELLTSMDDEIFRPKNTAQMVMAQKMLKYYNNFKLGM
YAFSILPSFGC
PIGRIIFGESGQKYCEAVITSSRGTAIYLFQAVSLGMISVINVVTNYFMVGFSLFIALQCDQL
CHHLEHI
DVTKNFKIKEFVQHHRRILRFAENTEKLFSFIYFSFIIMCLLAFCTTLFMISIIEDRYSFQCLH
LIFYQL
SIFIMLFIPCWFATQVNIKSEKIPLAAYSCAWTENPRSFKNDLIIFMNNSQKPIQFKAWNLVD
LSLETYM
AVIKTSFSYYTVLNSLIFEED
>PmacOR12
MKIKRGYLKAFNREKRVLQMFGVYPVKSSQIKYSLLRFIICFAVNSIQLYLMIMLLVTKDIS
NFLEAWHF
IIITIIVLFKISFLVLASTDMEDVEDYLNNFDTVDIPHGIVNYVIGEAYFRNRLYLPQWTTVV
SAVTFQF
SACLIFHNLGLVLFISWSPFNLQQPTYYYITLIFQVVVFFSNGMPNVTIDTMYYILVDVACC
EIDVLMYK
LSNLDPLKNPNETLKELKEYVVCHQKILRYITLIQRNVYSKLMFLQCIGSIMVICVLGFQFT
MTVNWFLF
IQHATYFSSMIFQIFGYTWFGQKFMGKSQEITQACYMSRWYECDIRIQKMLLNIMTRTKKP
LVLSSYIFD
LTLETFIGVLKSSYSYMALLRTMYTK

>PmacOR10
MSEMDNIDYRKFFTFNVRVFKICGVWKPDSNSNNKILITLYNSFCISLWTSFLFSQFMFLYN
SPSIFDEL
LSTSHITIPEYVCSLMFIVIYRKLDLIKLILDKLNEPMFQPKCEEHLQLAKSLEHFYKTYNYS
VTYLAIQ
TSLLFLIIPLWQDEQTLPAKAWFPFEWEPSPNFEMIWVFQSVSVCIVTVNVACLTIYTTGIFM
LIGLQCD
YLCITFNNLTQFHVENGILLNRGEKHLQISNPKFSDVMLENLVVCIRHYTEIRKMALEIERI
HEMGVFIL
FLGGATMISTILFSLSTVEIGSPQSLMLMSDTMCMLVMQFVYCWFGGEITNKSANVFMTS
YNTPWTDCNI
RYRKVLLQFMTMTQNPIEMRVGGLLVMSNAVFTSIVKSSYSVYALLHDIEE
>PmacOR22
MNTENVKVIKIMEHPIRILQIPYLFPSATEINDPKANIYFKYLLVNFSSIYYSVGALLHMIFN
LKRGIAD
HIDKDIGNILSYHGASYFLFRYLLNLKFIIILFKQFSDFETFGKPNNFDERNKSINKWSRVYL
CYQTTIL
VTMLLPAFLYIPQCKQENLEKGLQEVCGLPIPTWLPFRFDYFPVKQIVYLYECYSAFVVYQ
TAGMLSYTM
FASCEHLILRLEHVKHQLVNALNEKDVVIRRQKFNKAVQYHQAVIQMGTLLNKCFSPCM
MVHISLTGPFI
GVVGYTFLTNIPLDSTALLVGWLVSTYIVCYGGQRLMEASISVGNIMNRIHWYDLETDLQ
RDLILIIIRS
RRPIFLTAGPFGPITYSTVVTILKTSYSYVTLLNQTM
>PmacOR18
MSMIYPKQGEYRISYEPLSTLKKCIQLHDFKGTKKYFWHAAALLKVFILLGRTVYAGQTL
NQPKRLAEIV
ATYPVRFMAITKIYMLYFDRKRVNYFYNTVSQEFWDFHIAGPELEKQIKKRFFIINFAVACQ
FVAAMLVV
TLFLIFPLVDMPEGKRPLPNIIWTPFDTDPSPLYEIIYVIMIWNLTLSVLGNAFYDVLFTYSLQ
HLFVQF
MLLKKLIKNITKGILEETSDCDKFNSEYFQNKVYERFRICAEHHAKLLKFGKNLKMFSSRA
LLPQLIMSY
AVLVINGYILSIDHKDVMKTTGLLNLTGSCLVQLAVFSLQGSEIKFQSLSLLDAISNSEWYL
FRAPVKRA
FTFMMLNSKVPLTVSAGGMANVDNEILLEIVQKAFSAITLLRALTAEAEMKG
>PmacOR5
MEGNVKVNSLNGYPDNFFYTNSVCLKLVGMWIPSKEYSLLFRIIYGIYVTLIYSEGFVFIIC
ELLIFRET
MKKVSNFITYIEMLFTHIVGIIKYFVLILGRHKIRNLMNTLQDDKYFYEPINGISPGKIFSNG
KETNAKI
SKLTFVMYICVGVSAHISSELILNNEIKGQSFENTNKTCADYFPYFFKIPFDVTMKWRCEL
ALALMDMGL
IFHAAIIACYDGVFVALLNCLRCQLSIVCHVFRTLRPRSLKSVGLSETHMELLDTQNPILEK

EMYKQLSH
ATEHLKILLRVGEEIESMFTFVTLCQTLASLLIFAACLYMASSVPMNSPEFFSQMEYFSCVL
LQLMLFCY
FGNEITSASEEIRTALFDCDWLNSSKRLKQSIILTMIRMQRPLYLTIGKFSPLTLSTIVAVCRG
SFSYFA
LFKSIQ
>PmacOR2
MILRFSIFDYFRPNSHTTDYGKSMLWFVKFLLEAIFIWPDTERPELLIISCIIIWLAGIFSWWC
VFSYII
VNIEDFNKAVYGMCLLSIPTMIVMKMPFVLFKFKALKNLLKQIETEFWPYDVVDSETKKE
LKEVYSISIG
FMFFCFFIVQIYHGATLVIPVFLEERVLPLPCSLPFEWTDGIPYALIYIVHFICLELGITFGVLG
LDILI
LCLCICTSNQYRILRKCFLIYNTVDMRETNDLLRTITKRSMDNYSLEKEYLVRLIDHHNLL
LRFTTNLNE
VLSPLELGQLVISIMALCLGTYVLSMEELSTFHRTFAFIYLIGFNLQLVIDCTVGAEIYHQAT
FLPHCVF
HSNWMSLEDTNLKKDILFVLQSSQRFPQLMAYNLYSMDMVTFLKIQKFTLSVYTLLSNMS
AIKQNVT
>PmacOR1
METLSGLEPHRLPNYSAIFNNIFNTFGVWTEGKRLANSLIITTFYSTTVILLFIITPQFCHFIY
MYKARD
NVIAFADEFYVSIASLMIVCKDYCLIKHRLEIKELMKSMDSEIFRPKNTGQYRRIFKIIMDE
YKKLFWMI
TFVNVSFCVCVVIGPFFDLIKDGAKLLPLVDCYPFKVNESPVYEIMYVYQILVCFYLMMH
NFPFDLFILD
LIGFCIAECDVLCENMSELKKEESAIEYDRKLINCIKHHEEISRFVRNVERIFSFPILQQYFC
NLISSCT
TMFKLSIAEPMSVDFFKTLSYQCCLLLQMFLYCWTASTLTEKVC

>TcasOR92a
MSKSEKIHTLATYFDSNIAFLKLTAFWIYDDETTRRKKYLQHAYNIFWIFYLFVAYQPAELL
YVYYSFND
LSVFLRALRDIGNHVSLAYKAFNYFIMRRDILKLMETLQHGNYHYEDCGDFQPKLIVDEE
KKEALKWTKY
FLNFCNAICLSMFANGVFTFIFLSDKQYVERNGQRVYHQEQPVNTVSPFGSGTKLRFFVTF
IYTMIALTF
YAWTIVALDSLFITIMSCISSHLKILQGAFKTVRARCIMRTRAERLLKEETLHDPPFLENCVN
KEMIRCI
KHLQTVLSVSGKLESIYSTQTFVQTFISLGEMCFSLYLLSETADQNIGNEITYLIATGFELLM
YCWFGNR
ITEASLKISYALYESDWFPTSLSFKKQIIFTMTRMQKPINVTIGKITPLAFSTFLTIARGAYSFF
TFLKQ

RHGINH
>TcasORco
MMKFKVTGLVADLMPNIRLIQASGHFMLNYHADNSGALHTLRLGYCCMHLVFVLVQYG
CNFVNLVLERGD
VNDLAANTITVLFFTHCVTKFVYFAVRSKLFYRTLGIWNQPNSHPLFVESNNRYHGIALKK
MRRLLYIII
IWTSFSAIAWTGITFVGDSVHNIKDPENENLTITEPIPRLLVKAWYPWDAMSGMPYYITLVF
QIYYVFFS
LAHANLLDSLFCSWLIFACEQLQHLKEIMKPLMELSATLDTYVPKSADLFRAPSATSQDQL
IENDYNEKN
EDLKGVYSTRQELGGHFRGGALQNFGSGGVGPNGLTKKQELMVRSAIKYWVERHKHVV
RLVTAIGDAYGV
ALLLHMLTSTIMLTLLAYQATKITGVDKYAATVLGYLLFALAQVFHFCIFGNRLIEESSSVM
EAAYSCHW
YDGSEEAKTFVQIVCQQCQKAMSISGAKFFTISLDLFASVLGAVVTYFMVLVQLK

>TcasOR60
MIIQSVIKRNDLMVLSETLYFLTTHLTFVCKLANLEYHKKLLLDIEDMLKTTRFQKTLSLD
LIEKTGMNE
KIRKFNLVAKTFRIVCVWCVVLYVLVPYFDPGKSKTLPTPGWFPFNWTDKYYYGTYFFEV
AGISITAHMD
SSIDILSWLLVTIASFQCDILKENLKNIYYNYDKEHDIRETFKDCIRHHEEIIKFTTKVEQSFS
QGILLQ
FLCSALVICFTGFLMLVVPVLTFQFANTIMYFCCMMIQLGMYCWYGHEIMTTSDEIGQYF
YLANWYDSSL
TLRKDFAIFLERAKRPITLTAGGFVVLSLNTFTRILRSSYSYFAVLKHLYNKS
>TcasOR48
MVVQKIDLLEPFDNVTRLLKILGLWYSPNETIVYKIYKNFVMATCFLYTLTCTVYGFKFMS
FETLEIAFG
AVEGVLKSLMFRLKFQKIAESWQQIRQQEFQPRNEHQRTVLKWYIEVTKSLFLVYFFGVYI
GCISALTVS
SWLRHKDFPTDHWFPFNYRRPFLYQYIYVHITVGFYLTAFLNCASDSCFYLSLLHITAQCEI
LADTLKNV
HDLHKLNAAKKNSGQKGEDEVMNQILIECMKHYNLIKKYTSLVADCFKEIITLQFVPTIV
MICIAMYKIS
TLEPSNTQFWFFAFTELGAITQIFIYCFVGNLVTSTSQKLFYATFESQWYNASQKFKKNLIT
VMMAVQRP
VIFYGWNIFAINYATFKSIVQTSWSMCVAFRSTQDL
>TcasOR234
MQQAALRNFPWHYIKRIFIDFGYHRTMKIFTIVYFILYSGSLLLDLYYLFNNFSIAAMVRYG
CMIMLISY
VIAGMLFCFIFEKQLLNLLSEAETIFWPPEMITSELPKFIHRTNVLNYFIIAWFGLLGVILFPV
WGDQSE

WFLNVWAYKAYFGSWWYIPYNLFYYSQPMAAWTCVRLPFIMMYFSLQIKLQIFLLNQQIL
EIPKGHNTNS
ETAPDDLSYQEAVSQKMCLCISHNVKIKRWTKSFLRKVIQAMPVFVLLGILGSIFVTFSVLY
SFESTSTI
LKIRLVVVVGCTILSVYMFVEGSQRLCDESSQMFEMLAYSPWYLYNKNNRRILLTFMTNT
LEPITITWGG
IILNYNFGLTVRLQTIDL
>TcasOR50
MFTDSDKAYETLEIAVGSAEGVLKGIIFRTKFQKITESWQQIQQPEFQPRNEKQKSVLRRYI
EVTKTFFK
VYFSLVYVGCVTGIVVSSWLRHKDLPTDHWLPFDFRRPFLYPYVYVHVTVGLYLNSFTNC
VLDSCFYLSL
LHITAQCDVLADTLKNIHDLDKLNGKNVPERENVDEVMNKILTECMKHFKLIQKFTNVIT
DSFKEILTLQ
FVPTIAMICISMYKISTLHPSNTQFWFFIFTDIGATTQIFIYCFVGNLVTTTSEKLFYAAFESQ
WYNASQ
KFKKNVITVMMAVQQPIIFYGWNVFAINYETFKSIMRTSWSICVALKSTQDL
>TcasOR40
MIISVTGFVTKPFLFEERGFPVDVWLPTSLKDRLDVYWGFYIYVSIGVAYPVIASGVLDPLI
PSLLCLAT
GHLKVLNDNLEHLDEYSSEENGSKDSNLYKNIQKCIKHHIEILNFVYNHQKCFSLMVFSQF
LGSPMILCF
TCWNVSMREPFSLEWFQSLAYFLGLLLQLFFYCYYGTRLSEEFEHVTTAVYMGKWYKYD
VKSRKALIILM
ERSKKPTIVTAGKILDLSLETFTIILKRSYSLLAVLKNQN
>TcasOR68
MLAFVLYMLFVVSVPILEVLNLVLQEKITFKQIIDNAFMIAELGCLIPKYWPFVRNNDRLV
KCIHYFDSP
AFQPTKKKHREILQNCVKVCRAITIFFVAAVSSGYVSWSSRPISWKNHIFPTDLWLPYDPKV
APKLYNFL
VYTYLIKILKDNLQHLGEDTEAEFNQQSVINRLPKSEIMYRKIVKCVEHHNLILDFVKEFQ
YCFAQCAFS
QIAGSVVVLCVSCLQLTIVDLLSFDCLAMILFLVSMLSEVYFFCHFGTLLYEESSTISDAIYM
GSWYDYD
KKSKQALTILMERTKRPVIVIAGKLVQLSLITFSMILRRSYSLLAVLENYNIEIN
>TcasOR6
MWSLLPVLNGWTWQKKLPFPARYPLDVTKSPYYELAYVYQFICIWYITVANLNLDTIIIAL
MMYTSCQCD
LLCDDLKNLTETRFFDKKLIECIKHHKAILVFAEKSNSLFNMIVLGQIATSTVVLALTMFQL
SMVSPLSS
EGLNHLFYIGGIIMQILLYCWFGNEVEAKSSNILYAIYESTWSEASKNSKKNLIIFSIRCQRPI
KATAVK
LFALSLRTFITIVRSGWSYFAVLYNVGSE
>TcasOR153

MCRKYMHGSGLGPTSPKIKRFISLWLLFPVSLLLDVLVIYDFHFLDNDIFKTAELLESVSSF
GQLPIRKF
ILTYHSKLIQNLLEDRKKFWSYEMFGETYGKFLRRKMVLATRLIQTMIFFGASVATLMFVS
TLADDRKTV
PLECWIPEFKHSTHVVLVMQFCSLCEIIYLVGAVDCLYVLTCVDIKIQFLLLQKKLKTIQVG
VKPMEECL
NELTICVKHHNLLLRSHKSLNRIFSEYFFVQYFVSVLAACVQLYILMYITASLEDIMKSIVY
LSAVVFQV
AIFFMPASDIEEEAEQFAVEIYNVNWECTSGTKFRKQLLFMLMKAQKPLYMLGGGMIHAN
RNEYIVLFRL
AFSISTLLGGMNENGRTDK
>TcasOR53
MGFNFQSRTNKILRRLRYVGTWFENDSYDLYFLYAVLLNIYYNLHNIAQTMNVFYHLDDI
EEWSSSGLLT
LTTLLTNFKAYCVLTNKKRILKLNQILTRSVFQPRSDHQVKMATDKFKIFDTMYSLHSSGPT
LTVVFFSL
YSLAELENRKLPFNAWYPYDFKKTPNFELTYLFQFTACMVQALIHVNTDSLAFNFIAILVIQ
LDFLADNL
RNMCQKAESMEQSLLDCIRHHKEILACRNELYHILNVNLFGQFILSTTALCMTFLQMTVV
NPTSTHFIAI
LVYGMALLVELLMFCWWGNELIIKSQLIPQAAFESNWMEGSIFFQKNLVFFICRAQKEMM
LYAVGFFRIS
LNTFILVSTSVSENVVY
>TcasOR171
MKAQDSDNPYIVLRRVFVDFAFTSHMIIYTKITFVFHFLTLLLETYYMITNFNVELFSRYGC
MMCLMTYI
VLAKLLEILFARHIKFLEEERLSHFWKLEESSEETQKVVNAESSKIRKKTFFVLSWFVALGF
VLFPIFGD
LNDLFMFGRVYRNYFGSWAIIPFCIYVSTFPSIAYNSICLPAVVSYFIFHLNLQISLINDKLGK
ISEKSR
QSEIYQKLCSCVAHHVRLRRWTNIFQNELESALPFYLFLGAINSIAVSFFILYNLQNMTLIFEI
RLVVIS
VCNVLILWIFAEAGQEFSDNSDSIFDAVVACPWYSWNAQNRKIMLIFMLNCLKPMTFSWG
GVKLDYQFTV
TIVKMSYSYALVLYNWRYEK
>TcasOR173
MQSHLQILILSQKIGQISQNNNHMDDVSKFHDVGYQKKIRTSLHVCMCRHVTLKQWISKI
LQIVQKAIPV
YFSLAIIVLVTVMFCILYNVESASTTTIFKIRLVLVGICGAVVLFTFSETGQLLSDDTSQVFDT
LAASPW
HEWDPKNRKTLLMFLLNSLKPVKIYWGGFALDYQLGGSVIKTTFSYALVLFNLRKD
>TcasOR130
MSSKNYLSFPTGFLLGTGLLPESKFSRKIVSIGVFTPMTLFLVYLIVKKARNGENKDVLLW
AELFESLTT

CAHILSRKYVMYVHGGLISQIIKERGCYFWNYDIFGPKFGQTLERKMNICTKIVKIVVSGG
VVTIVLFCL
TTVFDKSKVVPLVCWTPEDSLQTGIIYAMEVLIMFEIMWALLSIDCFYLLICTDLRIQFILLQ
RMIKSIK
FGSNHDEKSFAKMVHCTQHHKFLLHFHAKLNTIFSSYFIVLYLVTVACASMHTYIILFKQV
ANLDISDWL
KVRFRSPGFGDSIKSACYLSGMLFQVGLYFVITSNVEIEVVEIYNLNWENTGSVKIRKHVL
FMLMKSQEE
LSVTGGGMLHVKRNEYVGLVRLAYTIATILGGMT
>TcasOR73
MSQVQHLKFNVLTLKLIMLWPPNGHMTLNYHLKSLSLLIIVYLSAWTCIYGILRPLFTESD
GYDVLIQRA
IATIDFIGCIYMRYCFLDKIQNVTSLIRELPTFEKFCGKVEIETTEKKVQKYSKAVTVYWFFG
NLMNCLA
PLHERTKCENLRKSQLYIERDPCGLMARCFYPFDVSKKLFPMAYFIQVYTCVVISYYVIVL
TMTLVGIMM
HILTQLKNCRNLIHNLHEEITERESLKKKIQRIIVYHIKIINLTMTRKHIFLNFTVTILKLSFLW
PSNDN
YDQWRLVKDASLIVSLMPCALPILAHFVLQITGDVYNMVTITENLIALICIIGMIYMTICFV
KNRKLVKT
LVKNLPAFTKYSKTTDIILTDKKANLYTKIFVFYGVIGNVVYMIMPYLNIEKCQQRQNNDV
PCGLVTRCW
FPFKFDYSPVFEIVFVHQFYTCLMVSVIILDLTMLICGFLMHITNQLKHLRGFIKRFDCSSQ
KIAEDVIY
CVKFHTAIITYSEKTNEAFGTMMMLHITLTSLVISALGFEILIVDNFNDSLRFTLHLLGWLV
LLLLICYY
GQLLIDESIAVAEDIYYVPWHLAPVDVQKDIYMILMRSQKPLTLNAANIGVMSFPTFLRVIS
SAYSYFTL
LLNIKS
>TcasOR72
MAKLEYLTGATFTLKCAVLYPIDSNNPKIKKILYAVWAIFFILTFVTGFIQCFVFVCINPFDLV
QEAMII
MSLVFYSTTFFYFIVFYKNWQNMVALVTNINKNFHRATDNVIEKISMDQASELSDKLAYV
WTSSLAVGSV
VPVVLAIATGNLEMPMPAWFPYDYNKSPVFEITYLWQVFCLITLAIIYGASDMFFPCITIIIG
QQFKILA
SNFKNNFYTSLIKLGAEESIVQNFSKDIKTHEFRSFYIKYGNIFKILNNAKFQTLNRAFLKR
NIKHHKLL
LRFCEDLNKILNTFLLIRVSAIVFNLIFIGFNIIISTDRTLMLGFCNYFCFGSTELFIHTYSGQIL
TENA
DFLWTLYECPWYLCDVTYQKMLILVQMRVKRMVSTKAGNFFTMIAPSFIAFQRAVFSYIT
LLKEVTDLGK
D
>TcasOR14

MLLKWSSVIEFNLFLLKWIGLWPGEDYQLNMYSFYGFSVIILILCGHTLSTGLTLILDSGDI
DTFTETMF
ILNIEFMTAWKALNFALNRKKFMQLLDAIDKTTFQPRNGKQVTLVLRNIDGWKVMFKMF
GISLGLSFIFT
GLLPIFSKTYKDRKLPMEAWYPFDSTKSPFYQLCYVYQMAAVAVAVMVILNVDTLVAAM
NICIGLQCDLL
CDNLRNLHTNTSKMQVFIYCWFGNEVIVKSSKIPYALFESDWTQDSLEMKKNMIVFILRT
QKTLKITVCH
VFDLSLPTFLTILKTGWSYFAFMNRVTSPH
>TcasOR108
MEKALKLVNILGLDPRKNDTFSKFRSIFCFTILISASFSSHLEFFLNFKGLETCERAAESIIPQ
YQTMCK
MATFLLYKTEMLDLIKKSERFWKLDRFGDLQAKNLHSTYPIFQIFFYVYVVILFLTCAMFA
LVNWIFDTG
KPISLCYGESEGLETPWVEFYIVLQSVEVTIIFLGITGYDMVFLYYAGSVCIQFQMLKMAFA
ERKMNERQ
FLKAVKHHEFLLQYVEQLGDIYSMWFLLQYFSSLFGICFGLFLISKEGLPTEPERLSKYFPYI
FSFTMQS
FTFCMTGTMLSDWSSEISDEIFHSDWSDDQVYKNKTARLIVMNRAQRPAKISIGKFLDLNL
RSFILLMRS
VFSFLAFVNNILNRIN
>TcasOR24
MRRLKKLLLVDIRQKLFKPRNRQQVVMVQSRVNFWKKIYFMFTGMGVATMFFWALFPIM
DGTVKEHRLPF
LAWYPFSVNKSPFYEITYIYQIVSVFFIVIVNMNSDMLLVALMNILGVQCDLLCDNLKNIQF
RERINEEF
LRCVNHHMQILSYASDCNKFFNTIVLAQFFTTVVSLGLTMYQLTIVTPFTSEFYSFIVYGGA
VLMEIFLY
CWFGNEVEFKSLNIPFASFGFDWTIGSVGLQKNLIIFIAKSQRPIRMSALNLFHLSLETFVKI
LRTAYSY
FALLNNVNSLN
>TcasOR340
MPYVQKDDPFWVLKKLSIDIVSHTVMKKLNFAICSFNSTLLLLQTIHIITSTNKILLMSAYG
PMMGFSCF
VIFQALAQTVYEKTVLELLCEFYSLYWPLDNVNPGKFLKQFRFLYVISFVTGGMFITSVLL
VSPVFKNEK
DIFLIREMFHNWGQILEVVFWAGLFFQTVWAILIACVLAYAIFGIKFQLSLLLYQIKGMKGL
RHQSMVKE
KLHSVIRRHVCLTGFVRKVVKTYWGLLQVEVCMFLVVNISMLFFFINSFSRSDWQHNLRP
LCITVTSFML
TTCLIVLCLQIPDMTGRIFDTTLELPWHLWNSKNRRTLLIFMTNSVQPIYINILGLGRLNSSS
VSEYVKM
IYSTTTVLCSLREGKK
>TcasOR94

MAIKICKFTRKNMQISLIWPREFEEINPGKWYYIRIVIFLITYGVFPFCTFLHAVVVIHNNLDI
RISEDI
GAVVSNIGISYMAIIYVQQQNQIAYLLKDLSDFKDFGKPPFFEEENKRLNFWSICTFIYPTC
GASLYNLS
KILEKSECNKINEENGLPATCGFIFPIWVPFNINYFPLFHIMLISTWFCTTMFVRLHLSISYNA
FEIAHH
IILRIKHLNGMIITCFDCQDYKISRQKFTTCVLYYKQILDLSNRLNQSFSSIMFVHFTMTSAV
CGCLEKQ
FVDGEYVGGFIHLVGWIISLFIASVGGQDLVNASQSISEAIWSSKWYLADIRLKKDVLFML
MRSQKDLHM
SVGSFGVLSYAFFVSVLKMSYSILAMLTS
>TcasOR78
MGHAIMTEILTYLTLMGFWPRSPKSSKASAFLIILSTSFLFFGILFYLIVNRQFGSSEIDSIETI
TSQFG
VLYYLILFTWKRNDIVEIVELLSDFSKFGKPPFFDQRSTRLNYRLSCIVLILIVANIVVAALPV
IYIDSC
HKANEQLNLTKTCGLIAPVWLPFDYNEYPRKHLVFAWEVYCCVMNYVGSGIGALTMVGT
MEHVIIRIEQL
KYIFPKILDQPNPRIREQMLKNWVRYHLALFEIGRLMNDAYKWSLSVIVLCVGALFACIGI
SMLQSTASQ
INSICLFFGWFPSIAFLCMWGQRLLDSSLSVGTAVYSSRWYDMDVAFQKSVLMILIRSQKPI
RISVGPFT
HLSMLLLLGVFQSAYSYINLLNATS
>TcasOR110
MDKVEFSDPLFFLNVIGMHPFKADKFSKFRLAFSIAVYFAVIFSGVLELIVNSQGLETYARA
SDTLIPQC
QLVCKIFVLAKYKKQIARLLNGSQRFWDLGQFGARYGNSFGKTHKYLKSFFLLYKVMLTF
TCLQFLAVKI
IFKIPKPIAISFGETKGLEPLYDHLYLVLHAMITLVTINLVNGFDGLFFYFIGHVLTELKMVK
VAFGDSP
IETNWSEEKRFKFAVRHHRFVLDFIEQFNIVYCTMLLVQHLTCLFGICFGVFLMTKDGVPP
DLDRASKYL
PYIVTFIFQTFTFCFAGNLLLSWSLEIPNEIFYHDWAKKTTYENKLAKIISMKRGQRAARLT
LGGFANLD
LDSFRMVLKNALSFFTFVNAMMNKKAVTSV
>TcasOR15
MLVKWSSVIKINIFLLKWVGLWPGEKYQLNVYSFYAFTVIILILCGQTLSTGLTLILGSGDV
DTFTETLF
VVNIEFMTAWKALNFALNRKKFIQLLNAIDKPMFQPRNDKQVTLVLRNIDGWRVMFKMF
AISLALSLIFT
GLLPIFTKTYKQRKFPYEAWYPFDSSKFPIYQLCYMYQMASASTLVVVILNVDTLVAAMNI
CIGLQCDLL
CDNLRNLHFDTSKSMNQKLIECIKHHKSIISFAEKFRQAFNWSIFLQFFISSTSLAIVMFKISR
TTNYGS

EYYRFISFACSVLVQVFIYCWFGNEVIVKSEKIPYALFECDWTPEPLEVKRSMIIFIIRTQRIL
KITVSY
MFDLSLPTFLSILKTGWSYFAFMNQVTEVNTSK
>TcasOR1
MMKFKVTGLVADLMPNIRLIQASGHFMLNYHADNSGALHTLRLGYCCMHLVFVLVQYG
CNFVNLVLERGD
VNDLAANTITVLFFTHCVTKFVYFAVRSKLFYRTLGIWNQPNSHPLFVESNNRYHGIALKK
MRRLLYIII
IWTSFSAIAWTGITFVGDSVHNIKDPENENLTITEPIPRLLVKAWYPWDAMSGMPYYITLVF
QIYYVFFS
LAHANLLDSLFCSWLIFACEQLQHLKEIMKPLMELSATLDTYVPKSADLFRAPSATSQDQL
IENDYNEKN
EDLKGVYSTRQELGGHFRGGALQNFGSGGVGPNGLTKKQELMVRSAIKYWVERHKHVV
RLVTAIGDAYGV
ALLLHMLTSTIMLTLLAYQATKITGVDKYAATVLGYLLFALAQVFHFCIFGNRLIEESSSVM
EAAYSCHW
YDGSEEAKTFVQIVCQQCQKAMSISGAKFFTISLDLFASVLGAVVTYFMVLVQLK
>TcasOR150
MAFDASQHNYLHLCLILYDLSGLRVSSNSFLKFLSLYVLYPLLLIMFVMVHLNVWFKHAN
IFEITEVFTS
ICIVASMCIRKTVLIQYGSTFEDVIQKHSQFWDYGLFGTKTESRLRKNMEFCFLLLKCFIISG
IASIIVR
CFSPLFMKELLLPQDCWIPGNQPVAKKIIYVLQIIFYIESMTYTPLFDGLYIIMTGNLKSQLIL
LQKAIE
SIDLKRQDDETSWRRVKECCQHHKFLLSILKKINKMYSNFFVCTYLLTIIGICIPLFVIFDKS
SNLTQIV
ESILVAIVMNTLLIMICIPGSEIEIEADRLITQIYNLNWYETRSLKIRKFILFWLMQVQVPVQI
KGGGVL
AVNRALILQIQRIAYSASTLLTGLTS
>TcasOR93
MTNLEIKICRATLKILKYSLIWPNEADEMNPGKWYYIRVATFLLITSLWVLSVFMHIVMSII
HDADVHLS
EDVAFCVAFCGLYYMTMIYVKNQPKVALLLRDLSKFQFGKPPGFEEKERILGFLSQFFFYY
CVMAVMVYN
LVKLLQKPDCEKMNEIKGLKENCGLLTPTWLPFDINYFPAFHLTFLYVFISTQILMKLALIIS
FNALEMA
YHVILRIDHLKIMITECLDQRNYEVSRRKLKTCILYHLEILSLSNRLNDCFSNIMFAHLTITA
AICGCLE
KQFVDGDNRLGALLHVCGWISALFVACIGGQHLLNASLSIPDAIWSSKWYEADVRIRKDL
LFMMAKSQVG
LHLNVGSFGVLSFSVFFSVLKMSYSILAMLTS
>TcasOR131
MEYLKYCQTYIRGCGLASNSSPFRKILAIYFFLPPCFLIIAFSVYELWDANNNDIFLVIEVLE
CISSYTQ

LTIRKYMIFTQNELMVEIINDCDQLWSFDTCGPELGKKFKQRMKNCWIMVKALVTCGFTT
FILMCISARA
DRDNLLPFLCWIPDFPYATEVLFLSQFMLLMELLYYVMATDGFYLLVCMDIHIQFEMMQE
MLKTIQFDVI
SEKESWEKLTELAKHHNRMLHQKLNQVFSKYYVVQYFMTVAAMTVQTYTLKYRMVNI
QTALKSIMYTFSL
MFQSAYYLFPASNIEIEAENFSTEIYFLNWQDHEDVKIRKHILFMLMKSQENLELMGEGM
VHINRNEYLL
MFRLAFTIATLLDGLNQL
>TcasOR135
MEYLRYCQSFVVGTGLVTNSPSFRKFLGWCFLLPLSLIVFAFSIYKIRDTDNDIFLMIEVME
SISSFTQL
MIRKYIIFIQGELMLEIFNECENLWSFDLFGPQLSEKFKQQMKNCWTLAKVLITSGFITIVL
MCISALTD
KTKSLPFICWVPNFSYAHELIFLSQFILLIELLYYVVATDGFYLLICMDIQIQYKMMGKMLK
SVQFGVIS
EEESWEKLVELANHHNKMLHEKLNKVFSKYYIIQYAISVSAMSAQVYTLMYSKVVIETAL
KSICYTISLL
LQVAYYFFPASNMEIEAEKFSTKIYFLNWQDNADAKIRKHILFMLLKSQKSLEMWGEGML
HINRNEYLLI
FRLGFTIATLLSGFK
>TcasOR92
MKNQEIKICRATLTVLKYSLIWPSEADEMNPGKWYYIRVVTFILFTCPWVLSVFMHLIVSIR
NNADIHLS
EDVALMVAFTGVYYMTIIYVKKQPKVAFLLRDLSYFQFGKPPGFDETERILGFLSKLTFCY
SVMAVVIYN
YIKYRQKPECERMNKLKGLKENCGMLTPTWWPFEINYSPAFQLIFLYIFTSTQVMMKLSL
MISFNVLEMA
HHIILRINHLKTMILESLDEQDYEASKRKIKTCILYHLEILGFAERMDDCFSNGMFAHLTITA
AICGCLE
KQFVDGDNQLGSLLHIFGWILALFLACLGGQHLINASETISDAIWSSKWYDADLRLRKDLI
FMMARSQVG
LYLNVGGFGILSYALFLSVIKMSYSILAMLTS
>TcasOR47
MYLDLNKGIADNTVLFRIMGHWPFGNPKLYRVYTHFVLINMYLYNLTSLINMLKNLDDTE
EVTATIYNLL
STVAVIIKANIFHYHFNHVKTIVTMFESEAFQPKNKQQEKILKNGIFWARFIFYYFFLTADLT
LVMWILF
PIMDGERRFPSNAWFPYDYLSGRNYTLTYIWQSIFIIYHALSNVCMDTFFAMLMVQTGAQ
CDVLNNQVSL
LGKESVDSDTVRGELGKCIHHHKLILKLAETIGLVFRNIVLVQFATSVSVLCETMFLLSLVK
TLNATFVM
LLFYQVAIFTQIFLYCWFGNEVVLKSAKLYYSAYESRWYECPQSFKKDLLFFMQRTQKPIV
LFVGKMFPI

TVITFTSILRSSWAYFMALRKVHDKS

>TcasOR17

MDDFNWISTVKTNLLLLHIGGIWPRGDGTHKLNLYTIYAIFITFTFTTYHCFSQIINFFFVDD
LQALTES
IFISLIQSMALVKAFYILKNMRILKNILKNLETNKMLQPRNLKQIKMVQPSLTQWRLLSQM
FWISAVFAM
CLFGAFPIVESTYKEFRLPYLAWYPFDTKSSPFYEIMYLHQFVSSYTIAIVDIGADTLIAALN
VFVATQC
EILCDNIRNINGSVEEMDSKWKECFTHHKEILKVARHCQKFFNWIVLMQFCASVICIGLTM
FQLTLVVSF
SSEFFSSLFYFGAITVQIFMYCWFGNEVELKSSKILYATFEANWVEAPHQVKKNILIFAIRCQ
NPIKMSS
LNVFYLTLETFMAIFRTSWSYFAVLRQIQNRISEE

>TcasOR25

MEKFDWRCPIRINLLLLRSVGLWPRGYGVYKRNYYLFYSIFTTITIVGGHNLSQVINIFYVY
SDLEALTG
TIFVATTNILALVKRYVFVRNLPLIKEILQTLNTYQFHPKTRQQLKIIQAPLRRWKLAYLCFS
IIVYFNV
AMWTLEPLLDKMIKNRRLPFEAWYPFNSKQSPNYEIAYCYQFICIWNITIANLNLDTLIFAF
MMFISAQC
EILCDDLRSLDVGFGPKLIQCIKHHKEILRLAKVTNNIFNFIILGQIATSTAALALTMFQLSLI
SSINTT
ALTHFAYMMGMLSEILLYCWFGNEIEVKSYLIPNAAYESQWMHQDRSVAKNLLILGCRCR
KPIKITAINL
FTLSLPTFIAILRSAWSYFALLSTINGK

>TcasOR325

MCNLHDRYSQFGCFIVITFRQDSKVFFLTNMYSKLFGERGEFSMSVVYFVATGLFQIKLIRL
MVYLLLIA
NTLGFLAILYQFILDAELVYIIQYGPIISGSTYALGSLYGIIFLRDAEEFQHGFQFWNEHEGSK
ETQNRI
KQHINSLTVSVILNTALAFVTGTSLILPNKDEIHYHYFIKILMDLETVPRRLSQALYYLYKLN
FVMMFPI
MTINSYRVLYFSRKFKFQVMLLLEHIETLTKNYNVDDINLFYNTRYQDYIKQKLIFFIRRHS
YIAQYVAK
INNSIGPFVVLYAISATLLGVSVLLIVATGTIYYNTYQIILCGAIYLSTIFCAVDGTETVEMES
VEIYNK
LLGQPWYTWNNENKRIFVIFLMNCKKPLQITKFSDTFYVNYDWGIAVLKKVYSLGSVFFN
LRRYIIDK

>TcasOR70

MPSIIDISFKININVLCLAGLYLPDKFKSLYRVYTYLVYVFIVIPVPTLGCVYLLAQEKITFRQ
IADNLF
LIAELGCFIPKYWPLVRHAERIKRCIHYFSAPIFKTDRKEHQEILDDCIKVCHQWSAFYFAS
VTAGFVSW
SIRPISWENHILPTDIWLPFDPHTASSAKVASVYFYLVLGKGFGLGIKILKNNLQHLGEYVD

EELASLEP
CRKAQLTYQKIRQCVIHHEHILAFVEEYEECFSQVALSQFVGAVVIFCVSCLQLTIVEVVSL
DFLAMMMY
FIAMLCQVYLYCHFGTILYDESDTISDAIYLSKWYEFDKRSKKALCILMERLKRPMTVTCG
KIFTMSLVT
FTMILRRAYSLLAVLENYNIELN
