# Supplementary material for: Antennal transcriptome analysis of olfactory genes and tissue expression profiling of odorant binding proteins in Semanotus bifasciatus (cerambycidae: coleoptera)
Source: BMC Genomics. 2022 Jun 22;23:461. doi: 10.1186/s12864-022-08655-w (PMC9219211; doi:10.1186/s12864-022-08655-w)
Supplement: Supplementary file 6 — Additional file 6: Table S1. Primer s for fluorescence quantitative real time PCR. [file 12864_2022_8655_MOESM6_ESM.pdf]

Table S1. Primer s for fluorescence quantitative real time PCR

Name	Forward primer	Reverse primer
SbifOBP1	TGCAGCTCTAACTGGCATTG	TGCTGTGCTGGAGACCAATAG
SbifOBP2	CAGTTGCTTCATGGTACTTGCG	ACACCCGCTTCTCTCATAACAC
SbifOBP3	ATGGGCCTGTTCAATGAAGG	TTTGCACCAACCAGAACACC
SbifOBP4	TTGCCGTTTCGAATGCACTG	TGGGTTGTTGGCATTTGGTG
SbifOBP5	AGCCGCCAGAGAGATAATTCC	AAGCCAAGCAGTTTCGCATG
SbifOBP6	AGAAGCCACAATTGCTGCTG	TGTTTGTCAGCCAAGCGTTC
SbifOBP7	CGCGATGACGGAAAAACAAC	ACTGCCCTTTTTGCAAACCG
SbifOBP9	ATGCTCTGCGAATCCAAAGG	TTTGGAACGGCGCATTCTTC
SbifOBP10	AGAAACCAGACGGCGAATTG	TGGCGCATTCCTTAACCAAG
SbifOBP11	TTTGCTGCTTCTCGCTTACG	TTTGCCTTCGTGGGTTTCTG
SbifOBP12	AGCTGGACGATATTGTGGAAGG	ATGGGCATGGATGATGTTGG
SbifOBP14	TGGCGCTCATTCTCTTTGTG	AGTGGCTCTTTGAACTGCAC
SbifOBP15	ACTTGCCAAGGCTATCAACG	TGTCATGATGCTCACCATGC
SbifOBP16	AGGCGATTGGGATGTAGATCAC	TTTGTCGTCCAACGTCACAG
SbifOBP17	TCAACAAGCAGTGCATGGAG	AAATCTCCAGCGGCGTTTTG
SbifOBP18	ATGCGGACGGTGAAATTCAG	AAGCAGTCTCCAACGGACTATC
SbifOBP19	TCTGCTGCTGGTTTTGTTGG	TGATGAGGCTGAGTCTTCCTTG
SbifOBP20	GGCTGAAGAAAATTCGCAGACG	TTCCAGCCTCATCTTGCATG
SbifOBP21	TTGCTGGTCAACTTCCTGAC	TGCACGAAACAGTTGGACAC
SbifOBP22	TTGGCATGCAAGATGACGAG	TGCCCATTTGCACCAAACAC
SbifOBP24	AACACGTGCTTTGCTTCTCC	TTTTTGGACAGCGCACTTGG
SbifOBP25	TCCGGGAATGCATATCTAGCAG	TGGCATGTTCATCAGCATGG
SbifOBP26	AAGCACACACGCACCAAAAG	AACGTTGACTGCTTGTACGG
SbifOBP27	GCAGGCGAAATACAAAGAAGGG	CAGCAGTCTGTTCAGGATCATC
SbifOBP28	AAATTCCCGCCAACATCGAC	AACACTTGTGCACCAGGTAC
SbifOBP29	AAATTGGAGAGCCACGCAAG	ACCGATCTTCTTGCTCATGC
SbifOBP30	AACTGGCAGATGCTTTCTCC	TCCTTCTCGCAAACATCACC
UBC	ACATGGCAAGGTCTCATAGTGC	TTTTTGGCGGCTTGAAAGGG
