# Supplementary material for: Antennal transcriptome analysis of olfactory genes and tissue expression profiling of odorant binding proteins in Semanotus bifasciatus (cerambycidae: coleoptera)
Source: BMC Genomics. 2022 Jun 22;23:461. doi: 10.1186/s12864-022-08655-w (PMC9219211; doi:10.1186/s12864-022-08655-w)
Supplement: Supplementary file 7 — Additional file 7. The version information or download links of the different software packages used for trimming/assembly/annotation. [file 12864_2022_8655_MOESM7_ESM.pdf]

Additional file 7：The version information or download links of the different software packages

Soft/Database	Version	Source
fastx_toolkit	Version 0.0.14	http://hannonlab.cshl.edu/fastx_toolkit/
Sickle	--	https://github.com/najoshi/sickle
SeqPrep	--	https://github.com/jstjohn/SeqPrep
fastp	Version 0.19.5	https://github.com/OpenGene/fastp
TGICL	Version 2.1	https://sourceforge.net/projects/tgicl/files/latest/download
Trinity	Version v2.8.5	https://github.com/trinityrnaseq/trinityrnaseq
SPAdes	Version 3.13.1	https://github.com/ablab/spades
BUSCO	Version 3.0.2	https://busco.ezlab.org/
cd-hit	Version v4.5.7	https://github.com/weizhongli/cdhit
hisat2	Version 2.1.0	http://ccb.jhu.edu/software/hisat2/index.shtml
samtools	Version 1.9	https://github.com/samtools/samtools.git
transrate	Version v1.0.3	http://hibberdlab.com/transrate/index.html
RSEM	Version 1.3.1	http://deweylab.biostat.wisc.edu/rsem/

kallisto	Version 0.46.0	https://pachterlab.github.io/kallisto/download
Salmon	Version 0.14.1	https://github.com/COMBINE-lab/salmon
bowtie2	Version 2.3.5.1	https://sourceforge.net/projects/bowtie-bio/files/bowtie2/2.3.5.1/
DESeq2	Version 1.24.0	http://bioconductor.org/packages/stats/bioc/DESeq2/
edgeR	Version 3.24.3	http://bioconductor.org/packages/stats/bioc/edgeR/
DEGSeq	Version 1.38.0	http://bioconductor.org/packages/stats/bioc/DEGSeq/
misa	version 2.3.6	http://pgrc.ipk-gatersleben.de/misa/misa.html
TransDecoder	Version 5.5.0	http://transdecoder.github.io/
HMMER	Version 3.2.1	http://www.hmmer.org/download.html
bwa	version 0.7.9a	https://sourceforge.net/projects/bio-bwa/files/
bcftools	version 1.9	https://github.com/samtools/samtools.git

GATK	Version 3.8	https://software.broadinstitute.org/gatk/download/
BLAST+	Version2.9.0	ftp://ftp.ncbi.nlm.nih.gov/blast/executables/blast+/2.9.0/
Diamond	Version 0.9.24	https://github.com/bbuchfink/diamond
WGCNA	Version 1.63	https://horvath.genetics.ucla.edu/html/CoexpressionNetwork/Rpackages/WGCNA/
STEM	Version 1.3.11	http://www.cs.cmu.edu/~jernst/stem/
maSigPro	Version 1.56.0	http://www.bioconductor.org/packages/release/bioc/html/maSigPro.html
GSEA	Version 3.0	http://software.broadinstitute.org/gsea/index.jsp
Rfam database	Version 14.6	http://rfam.janelia.org/

goatools	Version 0.6.5	https://files.pythonhosted.org/packages/bb/7b/0c76e3511a79879606672e0741095a891dfb98cd63b1530ed8c51d406cda/goatools-0.8.9.tar.gz
MSigDB database	Version 6.2	http://software.broadinstitute.org/gsea/downloads.jsp
STRING database	Version 11.5	https://string-db.org/
AnimalTFDB database	Version 3.0	http://bioinfo.life.hust.edu.cn/AnimalTFDB/
PlantTFDB database	Version 4.0	http://planttfdb.cbi.pku.edu.cn/
Pfam database	Version 32	http://pfam.xfam.org/
KEGG database	Version 2018.	http://www.genome.jp/kegg/
NCBI database	Version 2020.	ftp://ftp.ncbi.nlm.nih.gov/pub/taxonomy/accession2taxid/nucl_gb.accession2taxid.gz
PIR idmapping database	Version 2020.	ftp://ftp.pir.georgetown.edu/databases/idmapping/idmapping.tb.gz
